# Backbone Alterations in Cyclic Peptides Influence Both Membrane Permeability and Biological Activity

**DOI:** 10.1021/acs.jmedchem.5c01901

**Published:** 2025-11-06

**Authors:** Joseph Openy, Sabela Vega-Ces, Gulshan Amrahova, Emeline Mestdach, Celestine Chi, Benjamin Kissel, Peter ‘t Hart

**Affiliations:** † 28268 Max Planck Institute of Molecular Physiology, Otto-Hahn-Strasse 11, Dortmund 44227, Germany; ‡ Fakultät für Chemie Und Chemische Biologie, 14311 Technische Universität Dortmund, Otto-Hahn-Strasse 6, Dortmund 44227, Germany; § Biophysics, Discovery Sciences, 33367 AstraZeneca, Gothenburg SE-431 83, Sweden; ∥ Institute of Biochemistry, University of Münster, Corrensstrasse 36, Münster 48149, Germany

## Abstract

Cyclic peptides hold significant potential as disruptors of challenging targets such as protein–protein interactions, but their poor passive membrane permeability limits their use. In this study, we used a combination of cell-permeability assays and a target-agnostic cell-painting assay to observe how minimal structural changes affect membrane permeability and the biological activity of Sanguinamide A analogues. Significant differences in permeability were observed among the analogues, and for those that were permeable, NMR spectroscopy revealed conformational changes in response to environmental polarity. In the cell-painting assay, we observed that even though permeability generally correlated with overall morphological changes, distinct differences in specific activities were observed depending on the nature of the backbone modification. These findings highlight the importance of selecting the correct backbone modification when trying to balance both membrane permeability and biological activity, offering valuable insights for the development of cyclic peptides as therapeutic agents.

## Graphical abstract

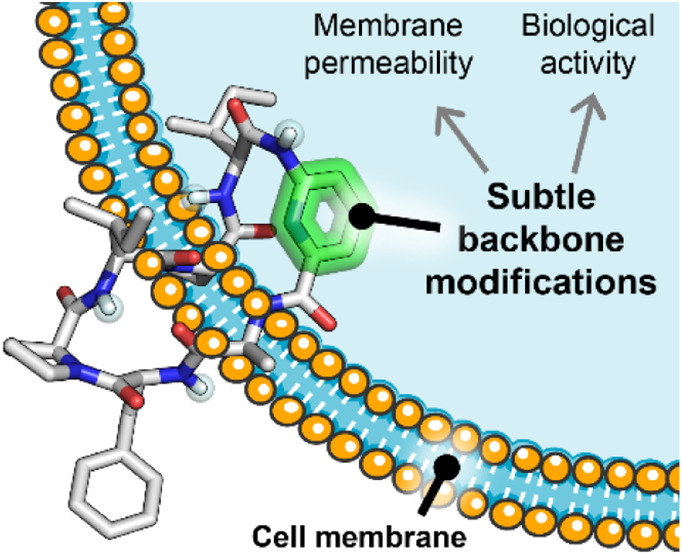

## Introduction

Macrocyclic peptides have the potential to bind to biomolecular surfaces that are challenging to target with small molecules, due to the lack of well-defined binding pockets.^1–3^ Pocketless surfaces are commonly found in protein–protein and protein-nucleotide interactions and therefore make attractive therapeutic targets.^4–6^ However, although macrocyclic peptides are interesting for therapeutic development, they are in the “beyond-rule-of-5” (bRo5) space, typically making them too large (>500 Da) and polar for passive membrane permeability which limits their use for intracellular targets.^7^ Nonetheless, several exceptions to these limitations are known especially among natural products.^8,9^ One of the most well studied natural product cyclic peptides is Cyclosporin A (1202.6 Da) which has a surprisingly high passive membrane permeability.^10,11^ The backbone nitrogens of Cyclosporin A are methylated at various amino acid positions (Figure 1), which is believed to induce intramolecular hydrogen bonds (IMHBs) so that hydrophobic amino acid side chains are forced to the periphery.^11^ In this conformation, the polar backbone amides are shielded effectively reducing the polar surface area (PSA) of the macrocycle during the transition through the hydrophobic parts of the cellular membrane. In an aqueous environment, the conformation changes exposing the polar groups to increase solubility, and this solvent dependent conformational change is referred to as chameleonicity.^12^

To promote passive permeability of cyclic peptides in the bRo5 space, various strategies have been developed to either increase the general hydrophobicity or to (simultaneously) promote chameleonicity. The most well described methods are backbone *N*-methylation (as inspired by Cyclosporin A)^13–18^ and the introduction of nonpeptidic backbone fragments^19–23^ (Figure 1).

**Figure 1:**
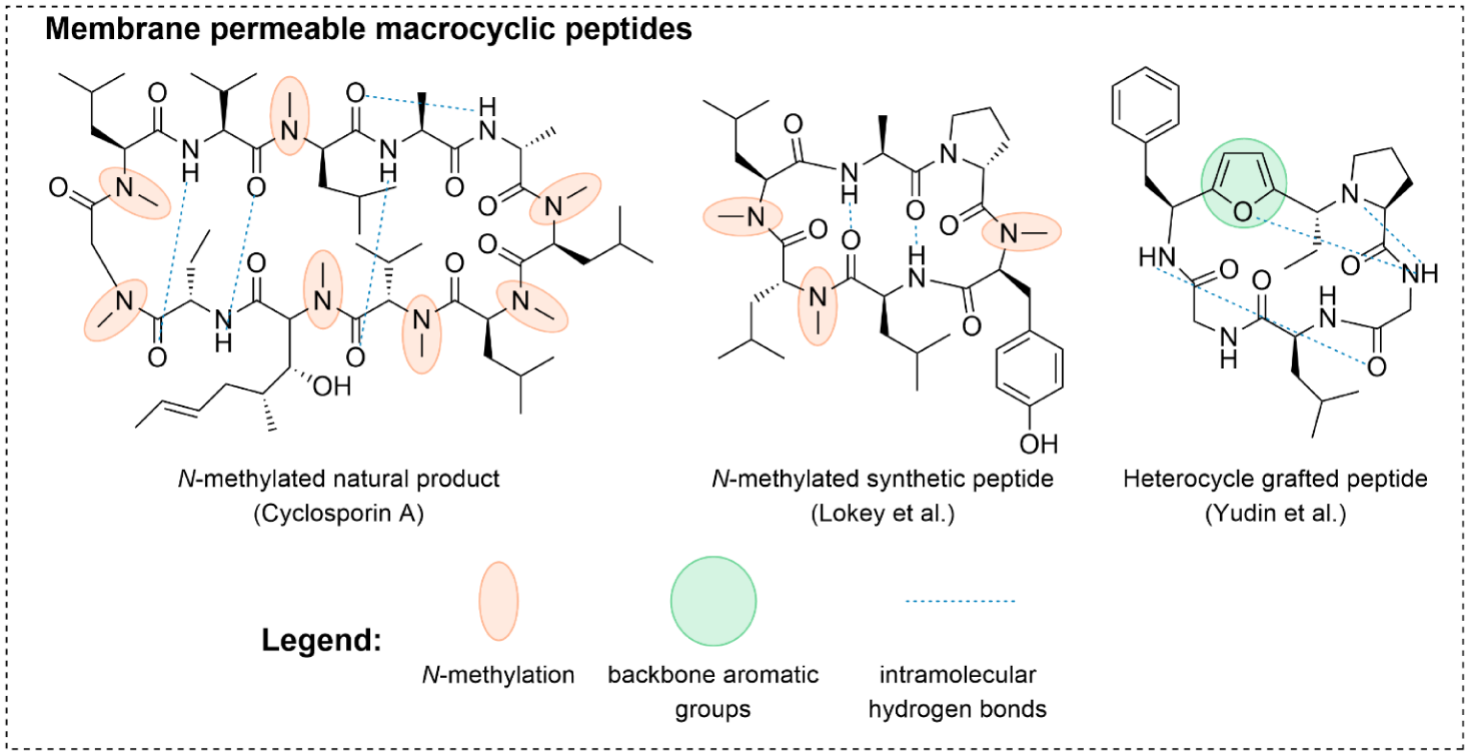
Structures of the cell permeable cyclic peptides in bRo5 space with either backbone *N*-methylation or backbone aromatic groups.^18,23^

Although these strategies have been shown to be effective at increasing passive permeability, they are typically used on model peptides or synthetic constructs without known biological activity. For such strategies to be implemented into the design process of a cyclic peptide with a biological target, the effect of these structural changes on the biological activity should be considered as well. A powerful method to overcome this issue is to evaluate the biological activity of a given compound using a target agnostic assay. In such an experiment, one does not observe a target specific read-out but rather analyzes phenotypic changes that could be caused by a wide variety of targets.

An assay that reports on virtually any target is the cell-painting assay, where changes in cell morphology are observed.^24–27^ First, cells are treated with the compound of interest, followed by staining of various cellular elements with selective dyeing agents. These dyes are fluorescently orthogonal to each other, allowing imaging of the cells in various channels simultaneously. Next, high content image analysis is performed to analyze a wide range of morphological changes of the stained cellular elements. The change in these parameters is then compared to a control group (i.e.: DMSO treated cells) and allows for a morphological profile to be generated facilitating the comparison between different compounds.^26^ Thus, the cell-painting assay allows the comparison of the biological activity even of model compounds for which no specific target has been described.

In this study, we describe the systematic modification of the natural product peptide Sanguinamide A by exchanging its backbone thiazole for well-defined aromatic groups. The backbone modifications were subtly altered by adjusting their polarity or adding methylene groups, thereby tuning the macrocycle’s flexibility. The resulting library of macrocycles was analyzed for membrane permeability using immobilized artificial membrane chromatography and the Caco-2 assay. To gain insight into the conformational changes in environments with varying polarity, the peptides were analyzed using variable temperature NMR and 2D-NMR. Finally, to investigate how structural changes affected their biological activity, the peptides were assessed using the cell painting assay. The observed levels of cell-permeability correlated with the intensity of the observed morphological changes in this assay; however, the nature of the biological activity was also influenced by the chemical modifications in the macrocycles.

## Results and Discussion

### Selection of a Model Peptide

To investigate the effect of minor backbone modifications on both passive membrane permeability and biological activity, we looked for a model peptide that had several desirable properties. First, a peptide with reasonable passive membrane permeability was required, allowing for tuning via subtle chemical changes. Second, we looked for a peptide for which a robust total synthesis had been described. Various natural product peptides could satisfy these criteria, but we found Sanguinamide A (Figure 2A) to be particularly suitable.^3,28,29^

**Figure 2:**
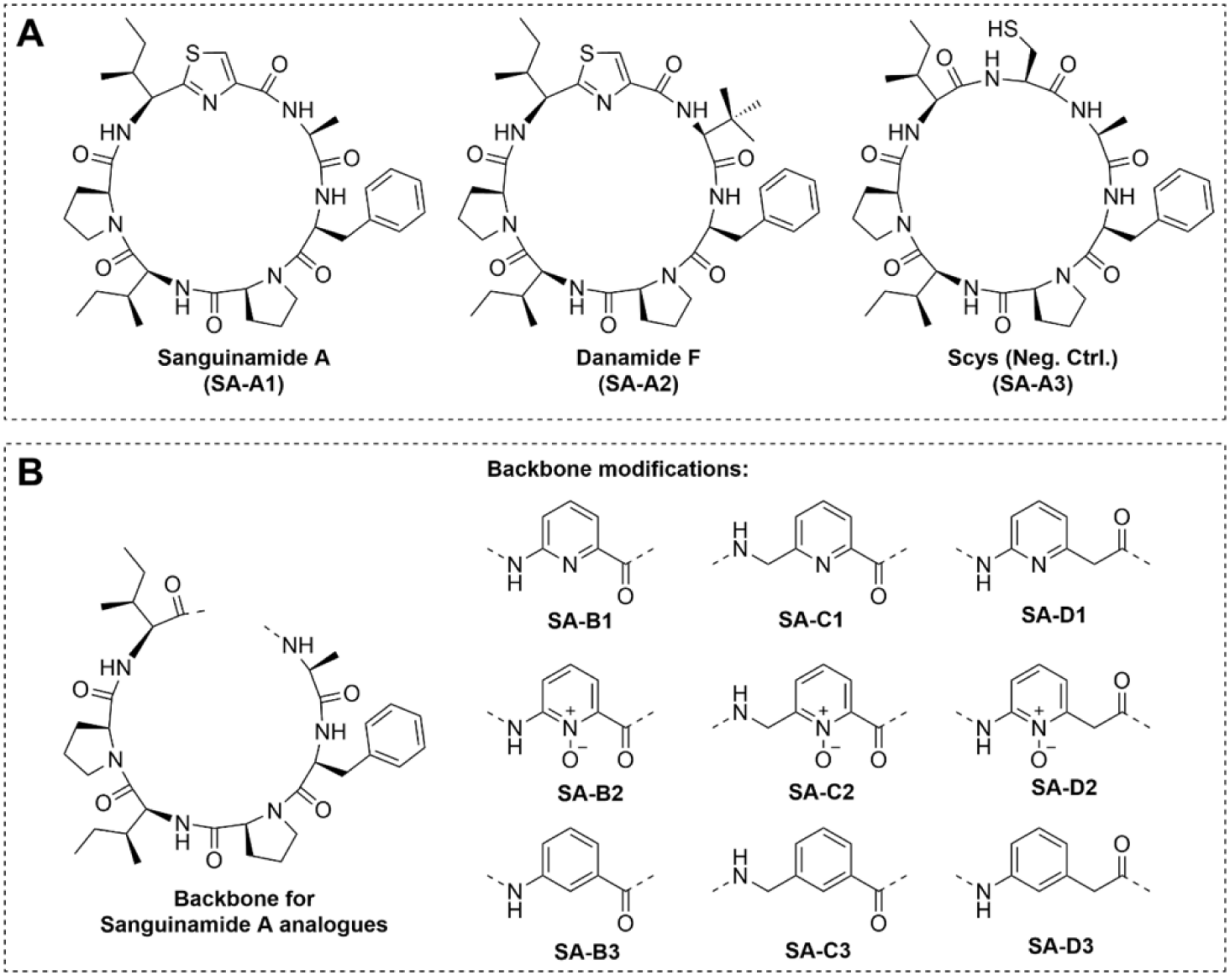
(A) Structure of Sanguinamide A, Danamide F, and a homodetic analog of Sanguinamide A. (B) Sanguinamide A analogues used in this study.

Although no biological function has been described, the peptide has been shown to have a medium degree of cell permeability and even bioavailability (F = 7%).^29^

The total synthesis was described by the Fairlie group and consists of the assembly of an isoleucine modified thiazole building block, which is installed into the peptide using standard Fmoc-based solid phase peptide synthesis (SPPS).^29^ After linear assembly, the peptide is cyclized in solution in good yields. However, what attracted our attention the most about Sanguinamide A, was a second report by the Fairlie group, where it was demonstrated that the cellular uptake as well as bioavailability could be improved by exchanging the alanine residue for a *tert*-butyl glycine (Danamide F, *F* = 51%, Figure 2A).^30^ Further work by the Lokey group also demonstrated that subtle modifications to side chains or backbone *N*-methylation lead to significant changes in the membrane permeability.^31^ These reports clearly indicated that cellular uptake of this scaffold could be modulated.

### Design of Sanguinamide A Analogues

Four sets of compounds were designed (Figure 2B), with the first being a set (set A) of control compounds including Sanguinamide A itself and the improved Danamide F (Figure 2A).^30^ A negative control homodetic peptide was added, where the thiazole was replaced with the cysteine it is derived from during biosynthesis. In the other sets, we replaced the thiazole with more simple aromatic groups to facilitate straightforward synthesis and therefore the introduction of minor modifications. We chose to only modify this part of the peptide, so that observed changes in permeability or biological activity would not be caused by alterations in the sequence, but rather by the chemical modifications made in one specific location. To this end, we chose either pyridine, pyridine *N*-oxide, or benzene, to study the effect of the polarity of the aromatic group (Figure 2B). In Sanguinamide A, the thiazole group is directly connected to the α-carbon of the isoleucine residue next to it. To simplify the synthesis, we decided to introduce an extra amide in this place, so that the aromatic group could be easily coupled to the isoleucine residue. Set B has the amine and carbonyl groups directly connected to the aryl functionality, but in the other sets an extra methylene was added to the amine side of the ring (set C) or the carbonyl side (set D). Sets C and D are more flexible than set B and therefore serve to study the effect of this property. Although the amide facilitates synthesis, it does introduce an extra hydrogen bond donor which could affect the membrane permeability.

### Synthesis of Control Peptides

The synthetic routes for peptide SA-A1 and SA-A2 followed the previously described total synthesis of Sanguinamide A.^29^ Peptide SA-A3 was assembled completely in linear form, followed by mild cleavage from the resin using hexafluoroisopropanol as a weak acid to maintain trityl protection of the cysteine residue. The peptide was then cyclized using PyBOP followed by treatment with TFA and scavengers to provide the final deprotected peptide.

### Synthesis of Pyridine-Based Building Blocks

Since we expected the nucleophilicity of the pyridine amine to be low, we decided to prepare the backbone building block as a dipeptide. By doing so, we could employ the same synthesis strategy as the Fairlie group used for Sanguinamide A. The synthesis of the dipeptide fragment for series B started from 6-aminopyridine-2-carboxylic acid which was first converted to methyl ester **1** using thionyl chloride in methanol (Scheme S1). We attempted to directly couple the aminopyridine **1** to Boc protected isoleucine, but the poor nucleophilicity of the amine did not yield the desired product under a variety of conditions. Stronger carboxylic acid activation conditions were not desirable, as they could lead to racemization at the isoleucine α-carbon, and we instead attempted to improve the nucleophilicity of the amine. This was efficiently achieved by converting the pyridine to *N*-oxide **2** using *m*-CPBA. The amide bond formation now proceeded smoothly, and subsequent saponification directly yielded the dipeptide fragment **6** for peptide SA-B2 (Scheme S1). Reduction of **3** in the presence of B_2_pin_2_, followed by saponification yielded fragment **5** for peptide SA-B1 (Scheme S1).

The fragments for the SA-C series were synthesized starting from 2,6-pyridinedicarboxylic acid (Scheme S2). Both carboxylic acids were esterified using thionyl chloride in methanol followed by a short reduction to selectively convert one of the esters to alcohol **8**. The alcohol was then converted to chloride **9** using thionyl chloride followed by quantitative azidation (**10**) and hydrogenation over palladium on carbon to provide amine **11** in very good yields. The amine was coupled to Boc-l-Ile-OH and either directly saponified to yield dipeptide **13** (for peptide SA-C1, Scheme S2), or oxidized using *m*-CPBA, followed by saponification to yield fragment **15** (for peptide SA-C2, Scheme S2).

The synthesis of the fragments for the SA-D peptides started from 2-amino-6-methylpyridine by first Boc-protecting the amine (Scheme S3). Next, the methyl group was carboxylated using diethylcarbonate and LDA to form compound **17**. After oxidation of the pyridine nitrogen using *m*-CPBA (**18**), the Boc group was removed using TFA to yield the free amine which could then be coupled to Boc-l-Ile-OH. Compound 20 was then either directly saponified or reduced before saponification to yield compounds **22** and **23** respectively (Scheme S3).

### Synthesis of Benzene-Based Building Blocks

The peptides that carried a benzene equivalent of the backbone fragment (SA-B3, C3, and D3) were synthesized using a slightly altered strategy. With a benzene group, a dipeptide fragment was not required since the nucleophilicity of the aniline was sufficient for regular amide bond formation. Therefore, the fragments could be installed in Fmoc-protected form on solid phase, followed by regular coupling of Boc-l-Ile-OH. To prepare the Fmoc-protected building blocks we used commercially available amines except for the building block required for peptide SA-C3 (Scheme S4). Instead, the intermediate **24** was first prepared by hydrogenation of the commercially available 3-cyanobenzoic acid. Next, all free amines were protected using Fmoc-OSu under aqueous basic conditions to yield compounds **25**–**27**.

### Synthesis of Cyclic Peptides

With all the required fragments in hand we assembled the peptides on chlorotrityl chloride resin as was done for the total synthesis of Sanguinamide A (Scheme 1).^29^

**Scheme 1:**
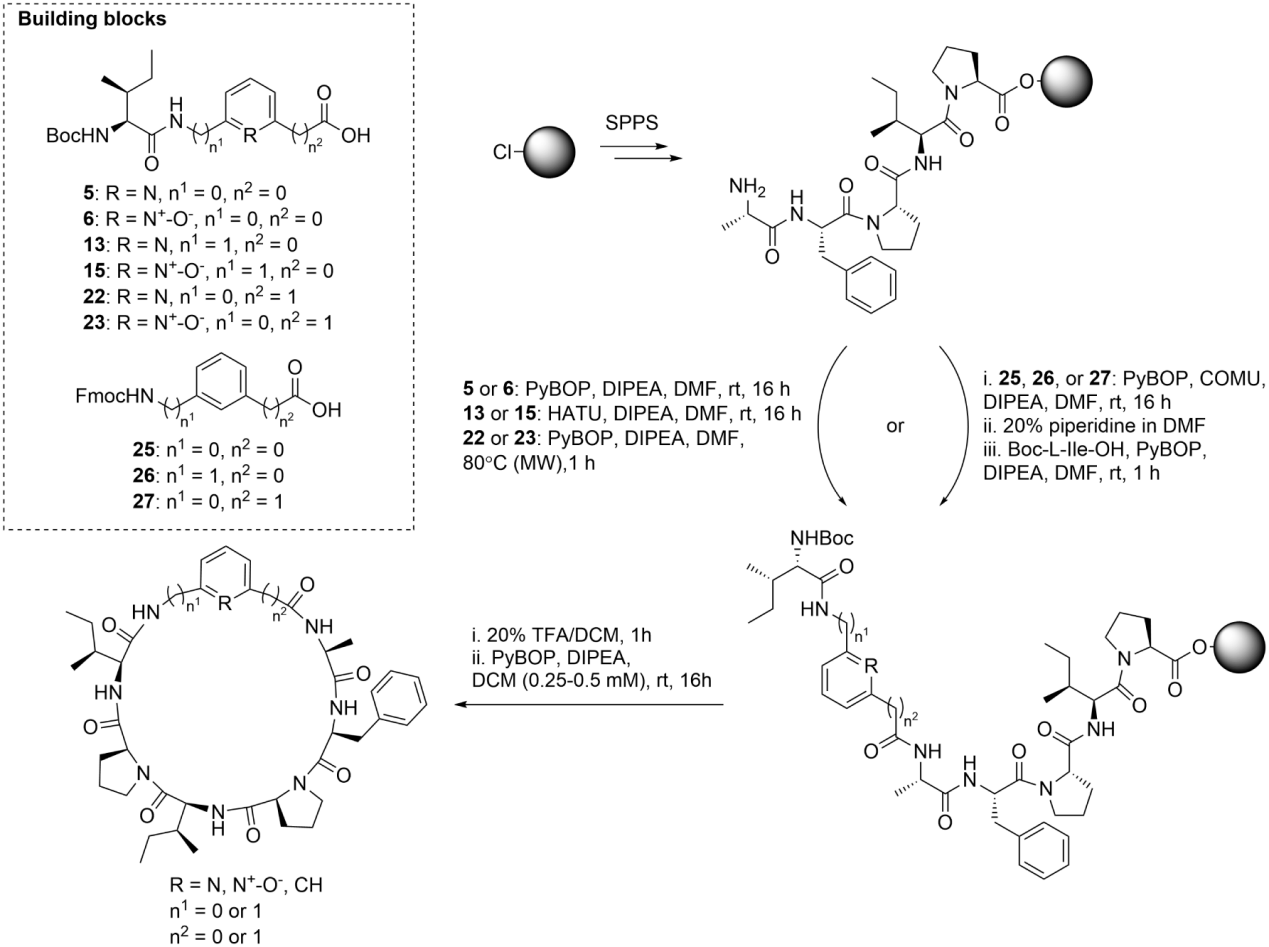
Synthetic Scheme for the Synthesis of the Cyclic Peptides SA-B1–3, SA-C1–3, and SA-D1–3^a^

a The structures of all used building blocks are shown in the box.

The precursor pentapeptide sequence was assembled using regular Fmoc-SPPS, and the dipeptide fragments were then coupled using COMU as the coupling reagent. For peptides SA-B3, SA-C3, and SA-D3, the Fmoc protected benzene amino acids **25**–**27** were coupled using PyBOP, followed by Fmoc removal and coupling of Boc-l-Ile-OH again using PyBOP. The *N*-terminal amine was always Boc protected so that treatment with TFA both liberated the *N*-terminus, while simultaneously cleaving the peptide from the resin to provide the free *C*-terminal carboxylic acid. The peptides were cyclized in solution using PyBOP under high dilution conditions (0.25–0.5 mM) to avoid dimerization.

### Cell-Permeability Analysis of Sanguinamide Analogues

With all peptides in hand we first set out to estimate their passive membrane permeability using the immobilized artificial membrane (IAM) chromatography technique.^32,33^ An IAM column is packed with a reverse phase like material modified with phospholipids to mimic a cellular membrane. After analysis of a compound its retention time is compared to a set of calibration compounds to yield a CHI-IAM value.^33^ A higher value indicates a stronger membrane interaction, which can be correlated to passive membrane permeability. Previously, the technique has been applied to both peptidic and nonpeptidic macrocycles.^34–36^ The CHI-IAM values indicated that SA-B1 maintained the same capacity to diffuse into a phospholipid membrane as Sanguinamide A (SA-A1) itself (Table 1 and Figures S35–S58). This is interesting especially since the design introduced an extra HBD as well as increased the macrocycle size, both of which are often detrimental for membrane permeability.^37^ Surprisingly, the *N*-oxide variant (SA-B2) had a similar CHI-IAM value as the benzene analogue (SA-B3) in this experiment. Although the values were not as high as SA-B1, the compounds still performed better than the negative control SA-A3. For all extended analogues (SA-C and SA-D series) the membrane affinity was strongly decreased indicating that the increased flexibility had a negative impact.^15,38^

**Table 1:** Parameters for All Compounds

Compound	CHI-IAM	Caco-2 A to B P_app_ (e^–6^ cm/s)^a^	cLogP^b^	tPSA (Å)^b^	Macrocycle size	HBD count
SA-A1 (San A)	39.71 ± 0.16	30.8	2.72	169.9	21	4
SA-A2 (Dan F)	45.23 ± 0.18	28.5	3.75	169.9	21	4
SA-A3	33.97 ± 0.13	0.900	0.69	186.6	21	5
SA-B1	39.88 ± 0.16	11.7	1.92	199	23	5
SA-B2	37.47 ± 0.10	1.210	1.16	213.1	23	5
SA-B3	37.94 ± 0.10	11.5	2.53	186.1	23	5
SA-C1	34.44 ± 0.13	0.580	1.60	199	24	5
SA-C2	31.64 ± 0.04	0.226	0.84	213.1	24	5
SA-C3	34.71 ± 0.08	0.214	2.21	186.1	24	5
SA-D1	34.95 ± 0.08	0.343	1.85	199	24	5
SA-D2	33.73 ± 0.02	0.194	1.09	213.1	24	5
SA-D3	33.89 ± 0.02	0.218	2.46	186.1	24	5

a Measured in the presence of transporter inhibitors.

b Values were calculated using RDKit.

To further evaluate the passive membrane permeability of our compounds, we used the Caco-2 assay in the presence of a cocktail of transporter inhibitors. The permeability was measured in A to B direction and the values correlated well with the CHI-IAM values (Figure 3A). One exception was compound SA-B2 which shows significantly lower membrane permeability in Caco-2 cells in comparison to compound SA-B3, even though the CHI-IAM values were similar. It has previously been described that the membrane interaction of charged molecules could be overestimated by IAM chromatography as they do not only interact with the lipid part of the stationary phase, but also the charged phosphatidylcholine group which carries both a positive and a negative charge.^39^ Such an effect could explain the discrepancy between the CHI-IAM and Caco-2 values for SA-B2. Besides this difference, it is also evident that Sanguinamide A (SA-A1) has a higher permeability in the Caco-2 assay in comparison to the IAM chromatography. The analysis did confirm the findings of the IAM analysis that larger and more flexible macrocycles indeed have low permeability. We evaluated whether calculated parameters could predict membrane permeability and although cLogP showed some correlation with both assays (Figure 3B,C), topological polar surface area (tPSA) was found to be a poor predictor of permeability for these compounds (Figure S1).

**Figure 3:**
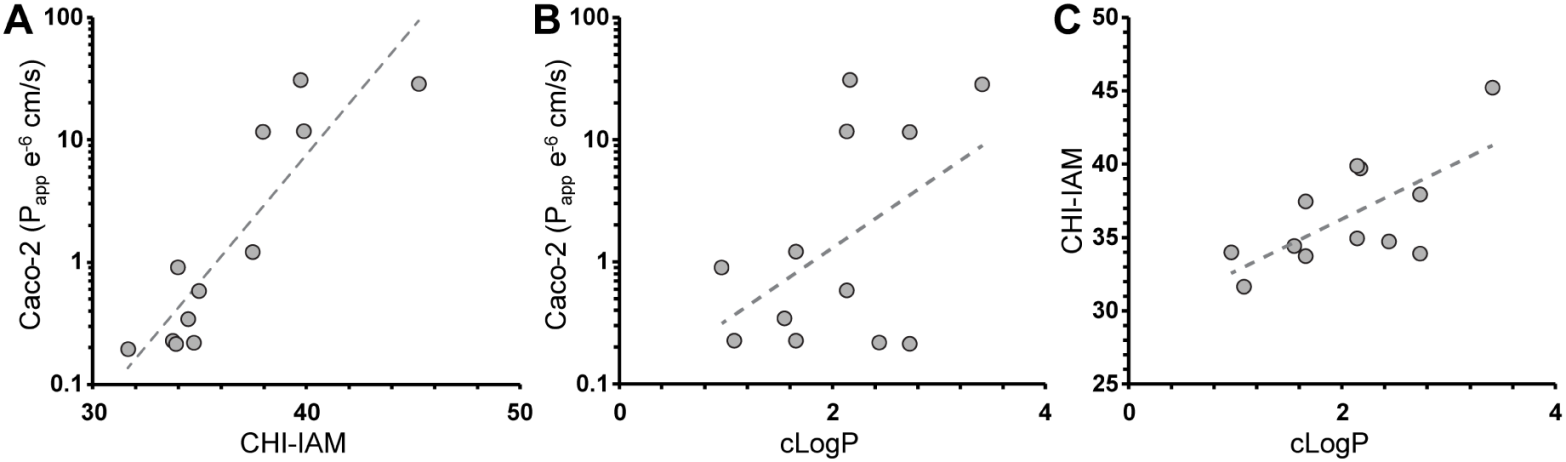
(A) Correlation between CHI-IAM and Caco-2 measurements. *R* ^2^ = 0.43 (B) Correlation between cLogP and Caco-2. *R* ^2^ = 0.36 (C) Correlation between cLogP and CHI-IAM. *R* ^2^ = 0.46.

### Variable-Temperature NMR Analysis

The solvent exposure of peptide backbone NH groups can be studied using variable temperature ^1^H-NMR experiments, where the change in chemical shift of the amide protons is observed upon increasing temperature.^40^ A large shift (ΔδNH/Δ*T* < −4.6 ppb/K) upon increasing temperature indicates that the amide proton is solvent exposed, while the opposite is true when the amide proton is shielded (ΔδNH/ΔT ≥ −4.6 ppb/K).^41^ These experiments were performed for compounds SA-A1, SA-B1, SA-B2, and SA-B3 in both DMSO-d_6_, as a mimic of a polar environment, and CDCl_3_, as a mimic of the hydrophobic membrane environment (Figures S2–S9, and Tables 2 and 3). In DMSO-d_6_, the observed shifts for Sanguinamide A (SA-A1) were similar to those observed previously by Nielsen et al.^29^ The SA-B peptides were mostly similar to SA-A1 although slight changes were observed in SA-B1 where Phe-2 seemed to be more shielded and Ile-6 more exposed. For SA-B3 the Ile-6 amide was also more exposed to solvent, but the change in Phe-2 shift was much smaller.

**Table 2:** VT-NMR Analysis of SA-A1, SA-B1, SA-B2, and SA-B3 in DMSO-d_6_ ^b^

Compound	Ala-1	Phe-2	Ile-4	Ile-6	Ar group
SA-A1^a^	–0.4 (0.4)	–4.2 (−4.2)	–1.7 (−1.5)	–3.8 (−4.0)	-
SA-B1	–0.5	–1.7	–1.7	–6.6	–1.5
SA-B2	–0.3	–4.2	–1.5	–3.6	1.3
SA-B3	–0.5	–3.7	–1.7	–6.5	–1.8

a Δδ_NH_/Δ*T* values reported by Nielsen et al. as measured in DMSO-*d*_6_ are reported in brackets.^29^

b Reported are the ΔδNH/ΔT in ppb/K.

**Table 3:** VT-NMR Analysis of SA-A1, SA-B1, SA-B2, and SA-B3 in CDCl_3_ ^b^

Compound	Ala-1	Phe-2	Ile-4	Ile-6	Ar group
SA-A1^a^	–0.1 (0.1)	–10.2 (−9.1)	–3.5 (−3.1)	–3.6 (−3.1)	-
SA-B1	–0.6	–10.4	–4.0	0.0	–1.0
SA-B2	–1	–6.4	–2.6	0.6	0
SA-B3	3.8	11.6	0	–0.4	0.6

a ΔδNH/Δ*T* values reported by Bockus et al. as measured in CDCl_3_ are reported in brackets.^31^

b Reported are the ΔδNH/Δ*T* in ppb/K.

Overall, these results suggest that only minor conformational changes occurred between all compounds in DMSO-*d*_6_. It has to be noted that a minor second conformer was observed for the SA-B compounds, which was not the case for SA-A1. Indicating that the extra backbone amide that was introduced during synthesis, led to increased flexibility in these compounds. In CDCl_3_, again only minor differences were observed for all SA-B peptides in comparison to SA-A1. Although various values changed somewhat, they never crossed the 4.6 ppb/K barrier in either direction. The biggest changes were observed for SA-B2, where increased shielding of Phe-2 and Ile-6 was observed. When comparing the data sets between the two used solvents, it is apparent that only one major change occurs for all compounds, which is an increased solvent accessibility of the Phe-2 NH. These results indicate that there is a relatively minor conformational change upon a change in environment polarity.

To further confirm the solvent accessibility of the amides we performed H-D exchange experiments using SA-A1, SA-B1, and SA-B3 by adding 20% D_2_O to samples dissolved in DMSO-*d*_6_ (Table 4 and Figures S10–S12). The rate of exchange of the amide proton for deuterium is an indicator of solvent accessibility. The analysis of SA-A1 showed similar trends in the exchange rates as observed by the Fairlie group.^30^ In line with their observations, the Ala-1 NH exchanges rather fast in SA-A1, which is in stark contrast to Ala-1 in SA-B1 and SA-B3 where it is fully stable. The Phe-2 amide is solvent exposed in all compounds, although the exchange is significantly slower for SA-B1. The Ile-4 amide is always strongly shielded which is in line with it being involved in a stable hydrogen bond with the Ala-1 carbonyl group as for SA-A1. A larger difference was observed for Ile-6 between SA-A1 and SA-B1 which seems to be much more accessible in the latter. Unfortunately, for SA-B3 this peak overlapped with aromatic peaks prohibiting quantification.

**Table 4:** H-D Exchange Rates in Minutes Observed by 1H-NMR Measured in 20% D_2_O and 80% DMSO-d6

Compound	Ala-1	Phe-2	Ile-4	Ile-6	Ar group
SA-A1^a^	57 (188)	48 (380)	>240 (6000)	341 (1500)	-
SA-B1	>240	156	>240	28	105
SA-B3	>240	20	>240	s.o.	267

a ΔδNH/Δ*T* values reported by Nielsen et al. as measured in D_2_O (10%)/DMSO-d_6_ (90%) are reported in brackets.^30^ s.o. = spectral overlap.

### NMR Structures

Although VT and H-D exchange-NMR provides some information on the conformational changes between different peptides, it does not provide information about their absolute spatial orientation or where actual IMHBs are formed. To gain more insight into the structures, we generated 3D models of the compounds SA-A1, SA-B1, and SA-B3 based on 2D-NMR experiments. The NMR solution structures were determined in DMSO-d_6_ and CDCl_3_ at 298 K, using a combination of 2D NMR spectra including ^1^H, ^13^C-HSQC, COSY, TOCSY, and ^1^H,^1^H-ROESY spectra (Figures S21–S26).^42^ ROE distance restraints (ranging from 49 to 64 per structure, as detailed in Table S2) were combined with torsion angle restraints (2–3 per structure) derived from ^3^J_H-Hα_ coupling constants, along with the respective proline conformations (cis for 2Phe-3Pro and trans for 4Ile-5Pro, as shown in Figures S27–S32).^29^ The structures were calculated using the CYANA software and the 20 lowest-energy structures from a total of 100 were selected and had no distance violations >0.2 Å (Figures 4A–E and S13–S20). The structures are in adequation with the observations made from VT NMR and the H-D exchange experiments. Indeed, the Ala-1 NH is involved in an IMHB with Ile-4 in DMSO-d_6_ for all three peptides correlating with the low VT-NMR values (Figures 4A–E and S19). It is much more stable for SA-B1 and SA-B3 according to the H-D exchange experiments, and this unexpected high rate of exchange for this NH group in SA-A1 was also observed by Nielsen et al.^30^ In CDCl_3_, SA-B3 displays the strongest VT-NMR change for the Ala-1 NH, as it is released from the hydrogen bond to be exposed to the solvent (Figures 4C and S18). It is possible that the exposure of this NH group in the apolar environment contributes to the somewhat lower cell permeability of SA-B3. Even though the aromatic NH group seems to be fully solvent exposed for both SA-B1 and SA-B3 it exchanges surprisingly slow (especially for SA-B3).

**Figure 4:**
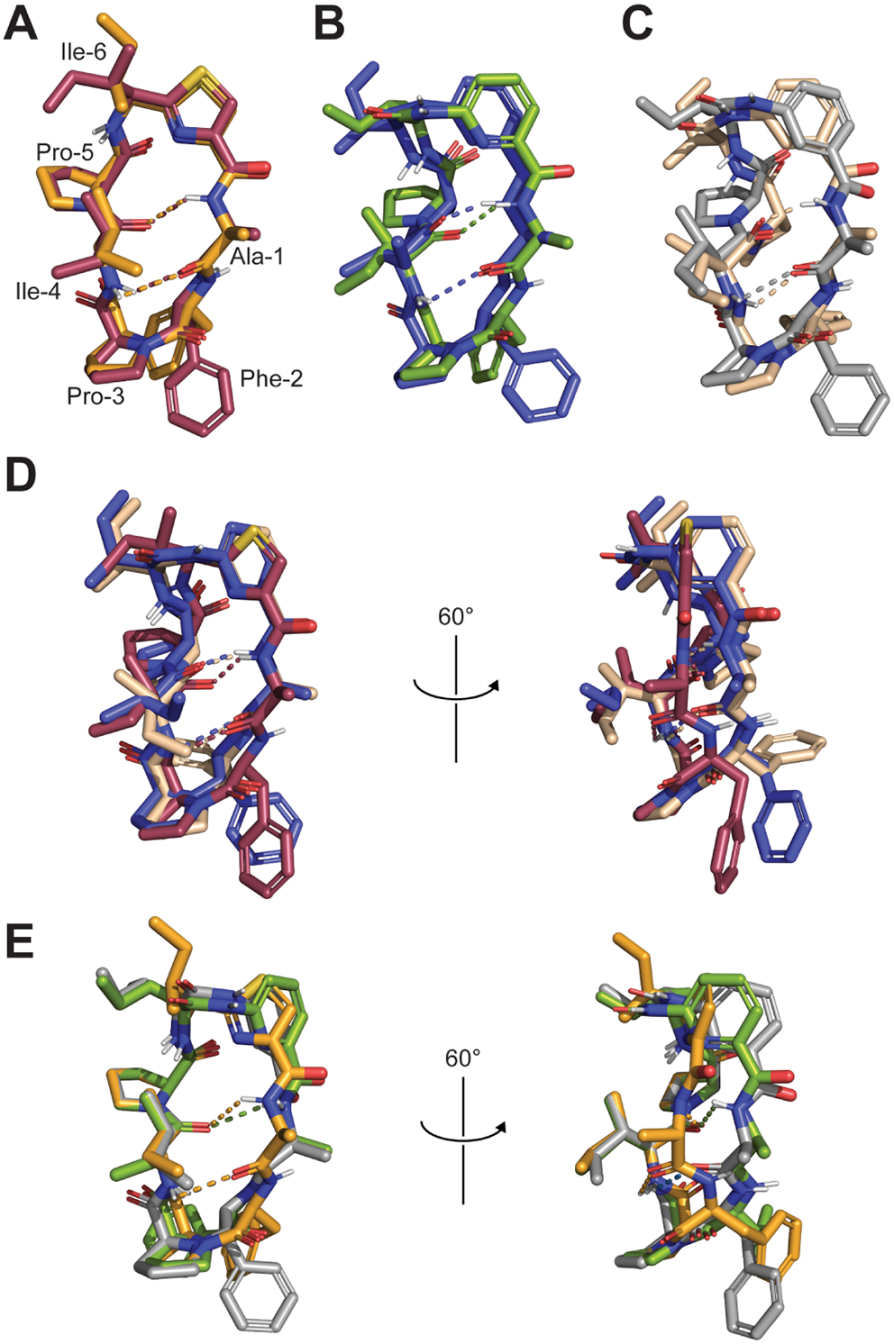
(A) Overlay of the structures of SA-A1 in DMSO-d_6_ (red) and CDCl_3_ (orange). (B) Overlay of the structures of SA-B1 in DMSO-d_6_ (blue) and CDCl_3_ (green). (C) Overlay of the structures of SA-B3 in DMSO-d6 (beige) and CDCl_3_ (gray). (D) Overlay of SA-A1 (red), SA-B1 (blue), and SA-B3 (beige) in DMSO-d_6_. (E) Overlay of SA-A1 (orange), SA-B1 (green), and SA-B3 (gray) in CDCl_3_.

A change in orientation of the Phe-2 side chain between the different solvents is observed for all compounds and shields the NH more in DMSO-d_6_ (low VT-NMR values) than in CDCl_3_ (high VT-NMR values). The significant difference in the Phe-2 H-D exchange rate for SA-B1 could be caused by this reorientation of the Phe-2 side chain (Figure 4D) although this is not observed for SA-B3. For SA-B1 and SA-B3, the shielding of this NH in DMSO-d_6_ is not only regulated by the Phe-2 side chain, but also the Pro-5 side chain shielding it from the back (Figure S19). The Ile-4 NH undergoes relatively minor transitions, with the exception of SA-B1 where the IMHB is lost in CDCl_3_ leading to the strongest change in VT-NMR. Similar to the change in Ala-1 exposure for SA-B3, this could be contributing to the lower Caco-2 values for SA-B1. For the Ile-6 NH, there is an interesting difference between SA-A1 and the SA-B peptides. Although virtually no change in VT-NMR values is observed for SA-A1, there is a quite strong shielding effect for both SA-B1 and SA-B3 in CDCl_3_. The effect is caused by a reorientation of the Ile-6 side chain, but also by an increased proximity to the Pro-5 side. We hypothesize that the increased shielding of this NH compensates somewhat for the lost shielding of the Ala-1 and Phe-2 NH groups in CDCl_3_ and therefore allows these peptides to still be membrane permeable (albeit less than SA-A1). The H-D exchange experiments confirmed that the Ile-6 NH is much more solvent accessible for SA-B1, in comparison to SA-A1 further supporting this hypothesis. Since the newly introduced fragments in SA-B1 and SA-B3 add an extra amide to the macrocycle, the overall conformation of their backbones is shifted in comparison to SA-A1 (Figure 4D,E). However, this shift is only apparent in the right side of the macrocycle (Ala-1 and Phe-2), while the left side is quite similar (Ile-4 and Pro-5), and it does not change the conformation of the turn made by Phe-2 and Pro-3 (mostly resembling a type VIa1 turn, Table S3).^43^ To accommodate this extra amide it is forced into the less stable cis conformation for both SA-B peptides in both solvents (Figures 4, S19 and S20). The lower stability is likely compensated by the ability to form intramolecular hydrogen bonds at other positions which would be hard to form when the amide would be in trans as it would force the peptide to take a more open ring conformation. Indeed, the H-D exchange experiment shows that the Ala-1 NH is now strongly stabilized when comparing SA-B1 and B3 to SA-A1. In general, it is clear this causes SA-B1 and SA-B3 to undergo much stronger conformational changes in the different environments. The energetic penalty that these conformational changes require, in combination with the less efficient shielding of NH groups, likely influences their cell permeability as highlighted in the lower Caco-2 values. These results also correlate with the lack of membrane permeability of the even larger macrocycles. For these compounds an even stronger conformational change would be required, increasing the energetic penalty, or the distance between polar and prospective shielding groups is now simply too large. To investigate whether chameleonicity plays a role in the membrane permeability of SA-A1, SA-B1, and SA-B3, we calculated their polar surface area (PSA) in the two different solvents. Interestingly, there was no significant difference in PSA between the polar or apolar environments for SA-A1 (93.2 Å in DMSO-d_6_ vs 92.0 Å in CDCl_3_) and SA-B1 (99.8 Å in DMSO-d_6_ vs 100.2 Å in CDCl_3_). For SA-B3 there was a decrease in PSA of approximately 8% (103.0 Å in DMSO-d_6_ vs 95.0 Å in CDCl_3_).

### Biological Activity Evaluation of the Sanguinamide A Analogues using Cell Painting

Since its discovery no biological activity has been reported for Sanguinamide A, making it hard to identify a cellular read-out that could potentially be connected to its membrane permeability.^28^ Therefore, we subjected our set of compounds to the cell painting assay that is agnostic toward specific biological targets. In our cell-painting setup, the compounds were tested at different concentrations (10, 30, and 50 μM) in U2OS cells followed by treatment with dyes for mitochondria (Mito Tracker Deep Red), endoplasmic reticulum (concanavalin-Alexa488 conjugate), actin, (Phalloidin-Alexa594 conjugate), DNA (Hoechst 33342), nucleoli and cytoplasmic RNA (SYTO 14), and Golgi and plasma membranes (WGA-Alexa594 conjugate). Next, the cells were imaged in various channels at 9 sites per well and a total of 579 morphological parameters were extracted.^44^ An induction score was calculated from this analysis, which represents the number of parameters for which the change was significant in comparison to the DMSO control. Compounds that generated induction levels greater than a threshold of 5% were considered to be active, while those below were considered inactive. Interestingly, all compounds that were observed to have high membrane permeability according to CHI-IAM and Caco-2 were found to be active (Table 5 and Figure 5A,B). Compound SA-B2, which was only poorly cell permeable, was only found to be active at the highest concentration tested. Both cLogP and tPSA did not correlate with either the induction values measured at either the 30 or 50 μM compound concentration (Figures S33 and S34).

**Table 5:** Cell Painting Induction and Cell Viability Values Measured using U2OS Cells for All Compounds

Compound	Induction at 50 μM (%)	Induction at 30 μM (%)	Induction at 10 μM (%)	Cell count at 50 μM (%)	Cell count at 30 μM (%)	Cell count at 10 μM (%)
SA-A1 (San A)	29.7	32.6	1.4	88	97	103
SA-A2 (Dan F)	95.7	41.1	12.8	15	66	100
SA-A3	1	1	0.7	98	102	102
SA-B1	23.1	15.4	2.2	82	87	101
SA-B2	7.8	0.2	0.5	103	98	99
SA-B3	32	15.5	2.6	94	99	96
SA-C1	1	0.2	0.7	99	96	98
SA-C2	1.4	0.5	0.7	109	104	102
SA-C3	0.2	0.7	0.3	99	97	98
SA-D1	0.9	0.2	0.7	100	100	97
SA-D2	1	0.7	0.2	94	99	100
SA-D3	0.7	0	0	96	100	105

**Figure 5:**
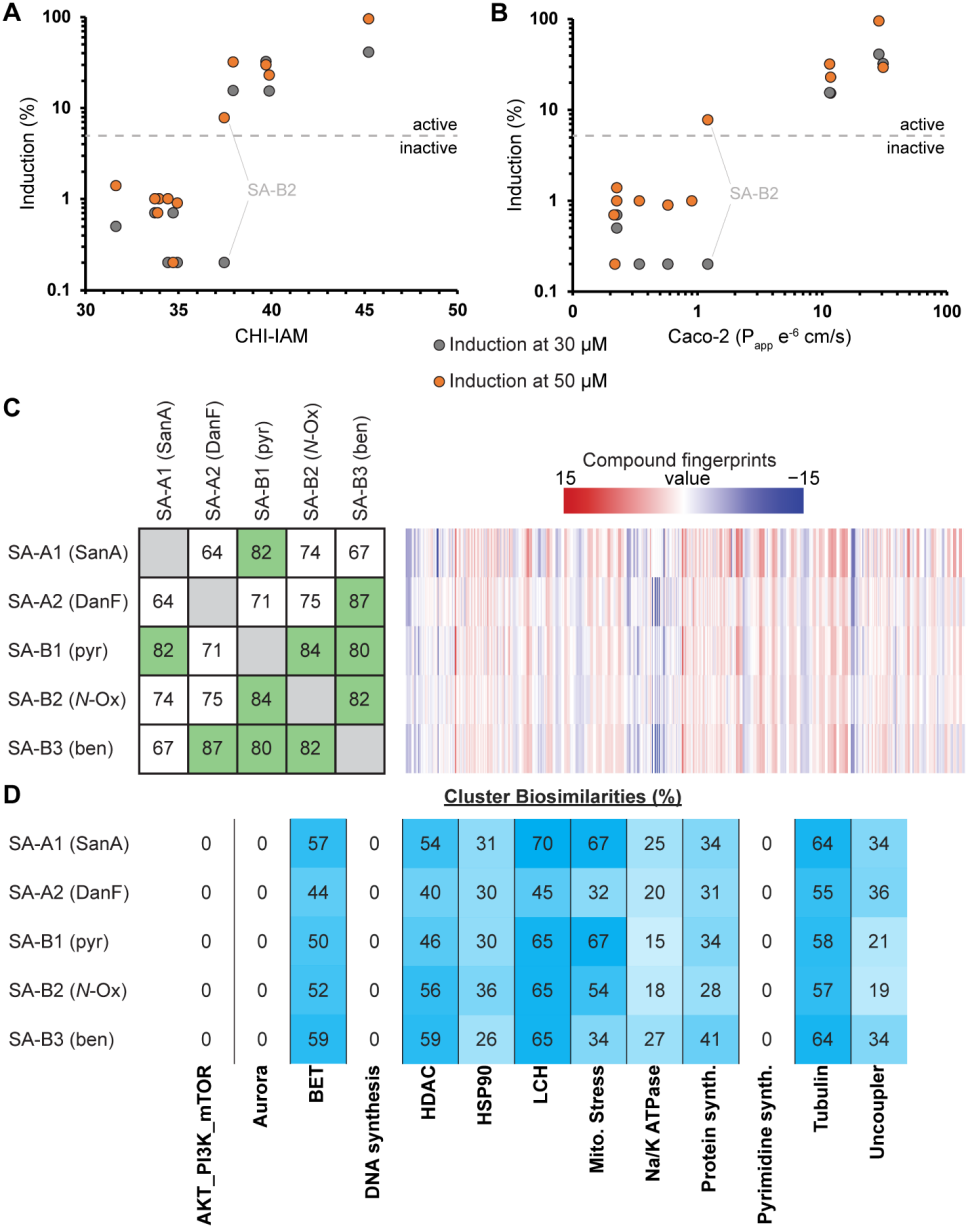
(A) Correlation between CHI-IAM and induction values at 30 μM and 50 μM of all peptides. (B) Correlation between Caco-2 and induction values at 30 μM of all peptides. (C) Comparison between the cell painting profiles of peptides. The profiles were determined at the lowest compound concentration where an induction threshold of a minimum of 5% was reached. The concentrations were 10 μM for SA-A2, 30 μM for SA-A1, SA-B1, and SA-B3, and 50 μM for SA-B2. (D) Comparison of all active compounds with previously determined profiles of clusters of compounds with similar biological activities. The same concentrations were used as for C.

Next, we compared the profiles of all active compounds to analyze their similarity. We selected the profiles of each compound at the lowest concentration that reached the activity threshold to avoid conditions that led to significant decreases in cell viability. The direction of the change of each parameter is compared between the different compounds to derive a biosimilarity score (in %) and the profiles are considered to be similar when this is >75%.^45^

By using this threshold, the only compound that could be considered similar to Sanguinamide A, was SA-B1 with a biosimilarity of 82% (see Figure 5C). The more hydrophobic SA-A2 and SA-B3 had the lowest similarity (64 and 67%, respectively), while SA-B2 was close at 74%. When using the other compounds as the starting point for comparison, it became clear that the hydrophobic compounds SA-A2 and SA-B3 were highly similar, as well as all the new analogues within the SA-B series. To streamline the interpretation of the data, subprofiles have been defined for clusters of reference compounds with similar biological activity (from a total of 4256 reference compounds that were analyzed).^45^ Comparison of the active compounds to these subclusters highlighted again that there were differences in biological activity (Figure 5D). In general, SA-A2 deviated the most from SA-A1 again confirming the previous observation when comparing their profiles directly. However, compounds that compare very well in the direct comparison, are not always similar in the subprofile analysis. An example is the difference between SA-B1 and SA-B3 in the mitochondrial stress and Na/K ATPase profiles. Also, SA-A2 and SA-B3, which are highly similar when compared directly, do not compare very strongly in the BET (bromodomain and extra-terminal motif proteins), HDAC (histone deacetylase) and LCH (lysosomotropism/cholesterol homeostasis regulation) profiles. Even for SA-A1 and SA-B1, which have the highest similarity when compared head-to-head, differences can be noted, especially in the Na/K ATPase and uncoupler profiles.

## Conclusion

By studying model compounds, we can improve our understanding of how chemical modifications affect the passive membrane permeability of cyclic peptides in bRo5 space. However, for a complete insight into which modification to introduce as well as where to introduce them in the structure, its biological activity should be considered as well. To provide a platform to study the effects on permeability and biological effect simultaneously we chose to use a combination of permeability assays and a target agnostic biological activity assay known as the cell painting assay.

We used two previously synthesized peptides, Sanguinamide A and Danamide F, and designed a series of analogues based on the first. Our aim was to only alter a small part of the backbone so that both permeability and biological activity changes could be attributed to these differences. The two used membrane permeability assays (CHI-IAM and Caco-2) correlated well and highlighted that the modifications have a pronounced effect. In all our analogues, the macrocycle size was increased by the introduction of an extra amide group to facilitate installation of the backbone aromatic group. The increase in macrocycle size (and therefore flexibility) led to a moderate reduction in uptake in both assays for the SA-B peptides. For the larger SA-C and SA-D series, the uptake was strongly decreased even though the size increase was only a single atom. The replacement of the thiazole with the larger alternative rings (and the addition of an extra amide) will influence the local electrostatic surface potential and dipole moment making direct comparison challenging. In contrast, the more modest changes within the SA-B series, and between the SA-B, SA-C, and SA-D series are more straightforward to interpret. For example, the exchange of an N to a CH (between SA-B1 and SA-B3) does not influence the conformation or permeability significantly, but influences the biological activity in various clusters as shown by cell painting. Furthermore, a slight extension of the peptides with a CH_2_ group on either side of the aromatic group leads to complete loss of permeability. These results highlight how sensitive bRo5 peptides are to how chemical modifications affect their passive membrane permeability.

To investigate whether conformational changes occurred during membrane transition we measured NMR spectra in two different solvents. Until now, the structure of Sanguinamide A (SA-A1) was only determined in DMSO-*d*_6_, and it was unclear if a more apolar environment would lead to large conformational changes. Interestingly, hardly any changes in the backbone conformation were observed, and the change of polarity of the environment only led to reorientation of various side-chains. In contrast, both SA-B1 and SA-B3 underwent rather strong changes in backbone conformation combined with side chain reorientation of Phe-2 and Ile-6. The large changes led to the breakage of an IMHB in CDCl_3_ for both peptides, which was also observed by Bockus et al. in certain Sanguinamide A analogues with higher flexibility.^31^ Furthermore, a lack of reduction in PSA for SA-A1 and SA-B1 indicates that chameleonicity does not play a role in the membrane permeability. In contrast, the change in PSA for SA-B3 does indicate that chameleonicity is at least somewhat involved.

Since no biological activity has been reported for Sanguinamide A, we turned to the target agnostic cell painting assay to see whether morphological changes could be observed upon treatment. Indeed, Sanguinamide A induced significant changes in U2OS cells indicating it is not biologically silent. All other compounds were evaluated and the degree of morphological change strongly correlated with cell permeability. Interestingly, the pyridine containing SA-B2 was the only compound that could be considered similar to the parent compound Sanguinamide A. All other modifications, including the *tert*-butyl glycine in Danamide F, led to compounds with more significantly changed profiles. Similarly, when compared to Danamide F, only the benzene containing SA-B3 was found to reach the similarity threshold. These results might be driven by the local polarity of the backbone aromatic group, although the cLogP values of these compounds do not explain these observations as they consider the entire peptide. Nonetheless, it is clear that very subtle single atom changes lead to compounds with different biological activity profiles even when their membrane permeability is similar. The differences became clearer when the compounds were compared to clusters of reference compounds with known biological activities. All compounds showed the highest similarity to compounds in the LCH cluster. However, the least similar was Danamide F, while the entire exchange of the aromatic group for any of the variants had a much smaller effect. In other clusters, the results were opposite where Sanguinamide A and Danamide F had similar values, but the values for SA-B1 and SA-B2 were reduced (Na/K ATPase and Uncoupler clusters). Perhaps even more interesting are the cell painting differences between SA-B1 and SA-B3, which are highly similar in structure with only a single exchange of N to CH. These two molecules have identical Caco-2 permeabilities, and their structures as determined in DMSO-*d*_6_ overlap very well (apart from the Phe-2 side chain orientation). While the direct comparison of these two compounds results in a high similarity (80% biosimilarity), differences can be observed in the subprofile analysis, especially evident for the mitochondrial stress cluster. These results highlight that a deeper analysis of the cell painting data is required beyond simply directly comparing compounds head-to-head. Nonetheless, since cell painting uses images of only a subset of cellular organelles, it is limited in the changes it can detect in terms of specific targets or pathways. Only when the engagement with a specific target leads to a morphological change in any of the detected elements, can a change in biological activity be observed. Furthermore, the biological activity clusters can only be defined specifically when a suitable set of well annotated molecules is available that targets relevant elements. For a more complete profile, the results would ideally be combined with proteomic and/or transcriptomic studies, to define further biological changes. This is also reflected in the fact that the cell painting assay did not yet lead to a conclusion on the biological activity of Sanguinamide A, even though this assay has the potential to do so. For this, a clear set of highly similar reference compounds would be required, or potentially the further definition of biological clusters. Alternatively, the references for the cell painting assay could be generated using a library of reagents for genetic perturbation of genes (e.g.: siRNA or CRISPR guide RNAs).

In conclusion, we have demonstrated that the cell painting assay is a powerful technique to identify how chemical changes in model cyclic peptides affect their biological activity even if a specific mode of action has not been described. The information obtained allows the design of cell permeability and biological activity to go hand in hand. Furthermore, we highlight that even minor chemical changes to the structure of a cyclic peptide, while improving permeability, can alter biological activity and that these can be derived from an in-depth analysis of the cell-painting data. Even though the signal generated by the series of compounds in the cell painting assay is moderate, significant changes in the biological activity were observed. Our findings suggest that minor chemical changes to the structure of a highly potent compound, in order to improve its permeability, could easily lead to changes in its biological targets and therefore induce off-target effects. These points should be taken into account during such medicinal chemistry campaigns.

## Experimental Section

### General Synthetic Methods

All solvents and reagents were of analytical grade and obtained from commercial sources unless stated otherwise.

Manual solid phase peptide synthesis was carried out on a promega vacuum manifold while automatic solid phase synthesis was carried using a Syro I peptide synthesizer.

Silica flash chromatography was carried out on a Büchi Pure C-850 Flash Prep using a flash column (RediSep, 24 g). Thin layer chromatography was performed using silica gel coated aluminum plates (Merk 60 F254) and visualized under UV irradiation at 254 nm. The purity of all compounds was ≥95% as determined by HPLC using the following methods. Analytical UHPLC was performed using 2.1 mm × 50 mm, 1.8 μm Zorbax Eclipse C18 Rapid Resolution columns equipped on either an Agilent 1290 or Agilent 1260 Infinity system. Analytical LCMS was performed using an Agilent 1260 Infinity system equipped with a 2.1 mm × 150 mm, 2.7 μm InfinityLab Poroshell 120 EC-C18 column. Preparatory HPLC purification was carried out using 125 mm × 21 mm, 5 μm, Macherey-Nagel C18 Gravity columns (Macherey-Nagel GmbH & Co. KG, Germany) on an Infinity II LC-MS system (Agilent Technologies, USA). High resolution mass spectra were recorded on an LTQ Orbitrap in tandem with an HPLC-System fitted with a 50 mm × 1 mm, 1.9 μm Hypersyl GOLD using electrospray ionization method.

Nuclear magnetic resonance (NMR) spectra of small molecules were recorded using Bruker DRX500 (500 MHz), and Bruker DRX700 spectrometers and chemical shifts are reported with reference to deuterated solvent peaks.

The synthetic schemes for compounds **1**-**27** can be found in the (Supporting Information Schemes S1–S4).

### Methyl 6-Aminopicolinate (**1**)

SOCl_2_ (5.25 mL, 72.4 mmol, 2.00 equiv) was added dropwise to a suspension of 6-aminopicolinic acid (5.00 g, 36.2 mmol, 1.00 equiv) in MeOH (100 mL) at 0 °C. The reaction was heated at reflux until everything dissolved and stirred overnight. The solution was allowed to cool to room temperature, and the excess solvent was removed under reduced pressure. The residue was dissolved in CH_2_Cl_2_ and washed with NaHCO_3_. The aqueous layer was extracted with CH_2_Cl_2_. The combined organic layers were dried over MgSO_4_, filtered and concentrated under reduced pressure to afford the title compound. Yield: 87% (4.79 g) as a white solid. ^1^H NMR (400 MHz, DMSO-*d*_6_) δ 7.51 (t, 1H), 7.33 – 7.13 (m, 1H), 6.65 (d, *J* = 8.3 Hz, 1H), 6.28 (s, 2H), 3.79 (d, *J* = 0.6 Hz, 3H). ^13^C NMR (101 MHz, DMSO-*d*_6_) δ 159.68, 145.70, 137.66, 113.18, 112.20, 51.85. HRMS (ESI): *m*/*z* calculated for C_7_H_8_N_2_O_2_ [M + H]^+^ = 153.0664, measured = 153.0659.

### 2-Amino-6-(methoxycarbonyl)­pyridine 1-Oxide (**2**)

*m*-Chloroperoxybenzoic acid (5.48 g, 31.73 mmol, 1.01 equiv) was added to a solution of pyridine **1** (4.78 g, 31.42 mmol, 1.00 equiv) in a minimum amount of CH_2_Cl_2_ required for dissolution at 0 °C. The reaction temperature was allowed to rise to room temperature and the solution was stirred overnight. The solution was purified by silica column chromatography (0–15% CH_2_Cl_2_/MeOH) to afford the title compound as a white solid. Yield: 52% (2.77 g) ^1^H NMR (400 MHz, DMSO-*d*_6_) δ 7.28 (dd, *J* = 8.5, 7.5 Hz, 1H), 6.98 (dd, *J* = 8.5, 1.7 Hz, 1H), 6.86 (dd, *J* = 7.5, 1.8 Hz, 1H), 3.85 (d, *J* = 0.6 Hz, 3H). ^13^C NMR (101 MHz, DMSO-*d*_6_) δ 162.28, 151.32, 139.63, 128.51, 111.29, 111.16, 52.84. HRMS (ESI): *m*/*z* calculated for C_7_H_9_N_2_O_3_ [M + H]^+^ = 169.0613, measured = 169.0609.

### 2-((2*S*,3*S*)-2-((*tert*-Butoxycarbonyl)­amino)-3-methylpentanamido)-6-(methoxycarbonyl)­pyridine 1-Oxide (**3**)

DIPEA (11.1 mL g, 64.23 mmol, 4.00 equiv), was added dropwise to a solution of **2** (2.70 g, 16.06 mmol, 1.00 equiv) and HATU (16.71 g, 32.11 mmol, 2.00 equiv) in CH_2_Cl_2_ (100 mL) at 0 °C. The reaction mixture was allowed to warm up to room temperature while stirring. After 16 h, the crude mixture was washed with NaHCO_3_, brine and dried over MgSO_4_. The solvent was removed under reduced pressure and the crude mixture was purified by silica column chromatography (0–30% EtOAc/petroleum ether) to afford the title compound. Yield: 90% (5.52 g) as a white solid. ^1^H NMR (500 MHz, DMSO-*d*_6_) δ 10.79 (s, 1H), 8.32 (d, *J* = 8.3 Hz, 1H), 7.98 (t, *J* = 8.0 Hz, 1H), 7.78 (dd, *J* = 7.6, 0.9 Hz, 1H), 7.01 (d, *J* = 8.6 Hz, 1H), 4.13 (t, *J* = 8.1 Hz, 1H), 3.88 (s, 3H), 1.85–1.73 (m, 1H), 1.54–1.43 (m, 1H), 1.37 (s, 9H), 1.29 (s, 1H), 1.15 (dt, *J* = 13.3, 8.1 Hz, 1H), 0.86 (d, *J* = 6.9 Hz, 3H), 0.82 (t, *J* = 7.4 Hz, 3H). ^13^C NMR (126 MHz, DMSO) δ 164.85, 155.48, 151.81, 145.81, 139.71, 120.47, 117.40, 78.10, 59.37, 52.40, 36.22, 28.21, 24.51, 15.33, 10.86. HRMS (ESI): *m*/*z* calculated for C_18_H_28_N_3_O_6_ [M + H]^+^ = 382.1978, measured = 382.1966

### Methyl 6-((2*S*,3*S*)-2-((*tert*-Butoxycarbonyl)­amino)-3-methylpentanamido)­picolinate (**4**)

Bis­(pinacolato)­diboron (2.50 g, 9.83 mmol, 1.50 equiv) was added to a cooled solution of **3** (2.50 g, 6.55 mmol, 1.0 equiv) in CH_2_Cl_2_ (100 mL) at 0 °C while stirring. After 7 h, the excess solvent was removed in vacuo and the residue was dissolved in EtOAc, washed with brine then dried over MgSO_4_. The solvent was removed under reduced pressure and the crude mixture was purified by silica column chromatography (0–30% EtOAc/petroleum ether) to afford the title compound. Yield: 99% (2.39 g) as a white solid. ^1^H NMR (500 MHz, DMSO-*d*_6_) δ 10.76 (s, 1H), 8.32 (d, *J* = 8.3 Hz, 1H), 7.98 (t, *J* = 8.0 Hz, 1H), 7.78 (dd, *J* = 7.6, 0.9 Hz, 1H), 6.98 (d, *J* = 8.6 Hz, 1H), 4.13 (t, *J* = 8.3 Hz, 1H), 3.88 (s, 3H), 1.77 (q, *J* = 7.9 Hz, 1H), 1.56–1.42 (m, 1H), 1.37 (s, 9H), 1.19–1.13 (m, 1H), 0.86 (d, *J* = 6.8 Hz, 3H), 0.82 (t, *J* = 7.4 Hz, 3H). ^13^C NMR (126 MHz, DMSO) δ 172.88, 165.29, 155.92, 152.25, 146.28, 140.12, 120.90, 117.85, 81.83, 78.57, 73.98, 59.84, 52.82, 36.70, 28.65, 25.41, 24.95, 15.77, 11.29. HRMS (ESI): *m*/*z* calculated for C_18_H_27_N_3_O_5_ [M + H]^+^ = 366.2023, measured = 366.2026

### 6-((2*S*,3*S*)-2-((*tert*-Butoxycarbonyl)­amino)-3-methylpentanamido)­picolinic Acid (**5**)

To a solution ester **4** (2.390 g, 6.54 mmol, 1.00 equiv) was added a 0.5 M solution of LiOH in THF:MeOH:H_2_O (4:1:1; 30 mL) in an ice bath. The reaction temperature was allowed to rise to room temperature while stirring. After 2 h, the excess solvent was removed in vacuo, and the reaction pH was lowered to 5 using 0.1 M HCl followed by extraction with CH_2_Cl_2_ (3 × 30 mL). The combined organic layers were dried over MgSO_4_, filtered and dried under high vacuum to afford the title compound as a white solid. Yield: 84% (1.94 g) ^1^H NMR (500 MHz, DMSO-*d*_6_) δ 13.26 (s, 1H), 10.71 (s, 1H), 8.28 (d, *J* = 8.3 Hz, 1H), 7.95 (t, *J* = 7.9 Hz, 1H), 7.75 (d, *J* = 7.4 Hz, 1H), 7.01 (d, *J* = 8.5 Hz, 1H), 4.12 (t, *J* = 8.3 Hz, 1H), 1.77 (p, *J* = 7.9, 6.8 Hz, 1H), 1.56–1.41 (m, 1H), 1.37 (s, 9H), 1.23–1.12 (m, 1H), 0.86 (d, *J* = 6.8 Hz, 3H), 0.81 (t, *J* = 7.4 Hz, 3H). ^13^C NMR (126 MHz, DMSO) δ 172.28, 165.80, 155.39, 151.54, 146.87, 139.41, 120.26, 116.86, 78.02, 59.29, 36.11, 28.09, 24.41, 15.22, 10.74. HRMS (ESI): *m*/*z* calculated for C_17_H_26_N_3_O_5_ [M + H]^+^ = 352.1872, measured = 352.1865

### 2-((2*S*,3*S*)-2-((*tert*-Butoxycarbonyl)­amino)-3-methylpentanamido)-6-carboxypyridine 1-Oxide (**6**)

Compound **3** (3.20 g, 8.39 mmol, 1.00 equiv) was added to a 0.5 M solution of LiOH in THF:MeOH:H_2_O (4:1:1; 30 mL) in an ice bath. The reaction temperature was allowed to rise to room temperature while stirring. After 2 h, the excess solvent was removed in vacuo, and the reaction pH was lowered to 5 using 0.1 M HCl followed by extraction with CH_2_Cl_2_ (3 × 30 mL). The combined organic layers were dried over MgSO_4_, filtered and dried over high vacuum to afford the title compound as a white solid. Yield: 91% (2.80 g) ^1^H NMR (500 MHz, DMSO-*d*_6_) δ 10.74 (s, 1H), 8.61 (dd, *J* = 8.7, 1.9 Hz, 1H), 7.99 (dd, *J* = 7.9, 1.9 Hz, 1H), 7.87 (t, *J* = 8.2 Hz, 1H), 7.44 (d, *J* = 7.6 Hz, 1H), 4.31 (t, *J* = 7.4 Hz, 1H), 1.87 (dtt, *J* = 14.6, 11.0, 5.4 Hz, 1H), 1.57 – 1.42 (m, 1H), 1.39 (s, 9H), 1.36 – 1.18 (m, 1H), 0.89 (d, *J* = 6.8 Hz, 1H), 0.82 (t, *J* = 7.4 Hz, 3H). ^13^C NMR (126 MHz, DMSO) δ 172.97, 161.29, 156.33, 143.01, 135.50, 132.94, 122.26, 119.43, 79.11, 60.40, 36.30, 28.62, 24.81, 15.78, 11.51. HRMS (ESI): *m*/*z* calculated for C_17_H_25_N_3_O_6_ [M + H]^+^ = 368.1822, measured = 368.1816

### Dimethylpyridine-2,6-dicarboxylate (**7**)

Thionyl chloride (5.52 mL, 76.1 mmol, 1.2 equiv) was added dropwise to a suspension of isophthalic acid (10.6 g, 63.4 mmol, 1.00 equiv) in MeOH (100 mL) at 0 °C and the reaction was heated at reflux while stirring. After 16 h, the excess solvent was removed in vacuo. The residue was dissolved in CH_2_Cl_2_ (50 mL), washed with NaHCO_3_ and brine, then dried over MgSO_4_ followed by concentration in vacuo. The product was used in the subsequent step without any further purification. Yield: 84% (10.4 g) as a white solid. ^1^H NMR (500 MHz, DMSO-*d*_6_) δ 8.78 – 8.71 (m, 2H), 8.65 (dd, *J* = 8.4, 7.1 Hz, 1H), 4.40 (s, 6H). ^13^C NMR (126 MHz, DMSO) δ 215.89, 175.44, 158.95, 149.15, 138.27, 62.59. HRMS (ESI): *m*/*z* calculated for C_9_H_9_NO_4_ [M + H]^+^ = 196.0610, measured = 195.0604.

### Methyl 6-(Hydroxymethyl)­picolinate (**8**)

Sodium borohydride (2.71 g, 71.7 mmol, 1.4 equiv) was added portion wise to a solution of **7** (10.0 g, 51.2 mmol, 1.00 equiv) in MeOH (100 mL) in a flame-dried round-bottomed flask under argon atmosphere at 0 °C. The ice bath was removed, and the temperature was allowed to rise. After 30 min following observation of effervescence, the reaction pH was lowered to 6 by dropwise addition of 0.1 M HCl and the excess MeOH was removed under reduced pressure. The resulting aqueous solution was extracted with CH_2_Cl_2_ (50 mL × 3) and the combined organic layers were dried over MgSO_4_, followed by concentration in vacuo. The compound was used without further purification.

### Methyl 6-(Chloromethyl)­picolinate (**9**)

The intermediate **8** was suspended in MeOH (100 mL) at 0 °C followed by dropwise addition of SOCl_2_ (5.52 mL, 76.1 mmol, 1.2 equiv). The reaction mixture was heated at reflux and stirred overnight. The excess solvent was removed in vacuo, and the residue was dissolved in CH_2_Cl_2_ (50 mL), washed with NaHCO_3_ and brine, then dried over MgSO_4_ followed by concentration in vacuo. The crude product was purified by silica column chromatography (0–30% EtOAc/Petroleum ether) to afford the title compound as a white solid. Yield: 66% (6.30 g) over 2 steps. ^1^H NMR (500 MHz, DMSO-*d*_6_) δ 8.03–7.98 (m, 1H), 7.92 (dd, *J* = 7.8, 1.1 Hz, 1H), 7.73 (dd, *J* = 7.8, 1.1 Hz, 1H), 4.61 (s, 2H), 3.87 (s, 3H). ^13^C NMR (126 MHz, DMSO) δ = 165.23, 162.55, 146.32, 138.21, 123.95, 123.09, 63.89, 52.41. HRMS (ESI): *m*/*z* calculated for C_8_H_8_ClNO_2_ [M + H]^+^ = 186.0316, measured = 186.0316

### Methyl 6-(Azidomethyl)­picolinate (**10**)

To a solution of **9** (6.6 g, 35.5 mmol, 1.00 equiv) in DMF (50 mL) in a flame-dried flask under argon atmosphere was added NaN_3_ (6.6 g, 35.5 mmol, 1.00 equiv) portion wise while stirring and the reaction temperature was raised to 90 °C. After 16 h, the reaction was quenched by adding H_2_O (20 mL) and stirred for a further 10 min. The excess DMF was removed under high vacuum, and the crude product was dissolved in CH_2_Cl_2_ (50 mL), washed with brine and dried over MgSO_4_ and concentrated in vacuo followed by purification by silica column chromatography (0–30% EtOAc/Petroleum ether) to afford the title compound. Yield: 95% (6.50 g) as a white solid. ^1^H NMR (700 MHz, DMSO-*d*_6_) δ 8.04 (t, *J* = 7.7 Hz, 1H), 8.01 (dd, *J* = 7.8, 1.3 Hz, 1H), 7.69 (dd, *J* = 7.5, 1.3 Hz, 1H), 4.63 (s, 2H), 3.89 (s, 3H). ^13^C NMR (176 MHz, DMSO) δ = 165.00, 156.27, 147.31, 138.73, 125.93, 124.09, 54.17, 52.52. HRMS (ESI): *m*/*z* calculated for C_8_H_8_N_4_O_2_ [M + H]^+^ = 193.0720, measured = 193.0719

### (6-(Methoxycarbonyl)­pyridin-2-yl)­methanaminium Chloride (**11**)

A 100 mL round bottomed flask under argon atmosphere was charged with 10% Pd/C (3.60 g, 3.38 mmol, 0.1 equiv) followed by 3 evacuation and argon refill cycles. The catalyst was then suspended in MeOH (30 mL) and charged with a solution of 10 (6.50 g, 33.8 mmol, 1.0 equiv) in a minimum amount of MeOH required for complete dissolution followed by 3 evacuation and H_2_ refill cycles. The reaction mixture was charged with 36% HCl (8.70 mL, 101 mmol, 3.0 equiv) while stirring under H_2_. After 48 h, the suspension was filtered using a bed of Celite, concentrated in vacuo and the residue dissolved in CH_2_Cl_2_ and used in the next reaction without further purification.

### Methyl 6-(((2*S*,3*S*)-2-((*tert*-Butoxycarbonyl)­amino)-3-methylpentanamido)­methyl)­picolinate (**12**)

The flask with the solution of **11** was charged with Boc-Ile-OH (11.7 g, 50.6 mmol, 1.50 equiv), PyBOP (26.4 g, 50.74 mmol, 1.5 equiv) and DIPEA (17.7 mL, 101 mmol, 3.0 equiv). Extra CH_2_Cl_2_ was added until complete dissolution occurred while stirring. After 16h, the organic phase was washed with citric acid, saturated NaHCO_3_ and brine then dried over dried over MgSO_4_ and concentrated in vacuo. The crude product was purified by silica column chromatography (0–30% EtOAc in petroleum ether) and provided an off-white solid after evaporation of the solvents. Yield: 41% (5.63 g) over two steps. ^1^H NMR (500 MHz, DMSO-*d*_6_) δ 8.59 (t, *J* = 6.0 Hz, 1H), 7.92 (d, *J* = 4.4 Hz, 2H), 7.55 (t, *J* = 4.5 Hz, 1H), 6.83 (d, *J* = 8.6 Hz, 1H), 4.42 (t, *J* = 5.7 Hz, 1H), 3.88 (s, 3H), 1.79–1.61 (m, 1H), 1.47–1.41 (m, 1H), 1.40 (s, 9H), 1.22–1.03 (m, 1H), 0.82 (dd, *J* = 8.3, 6.7 Hz, 6H). ^13^C NMR (126 MHz, DMSO) δ 172.49, 165.69, 159.94, 156.03, 147.23, 138.36, 124.95, 123.60, 78.51, 59.53, 52.84, 44.51, 36.46, 28.66, 25.01, 15.93, 11.38. HRMS (ESI): *m*/*z* calculated for C_19_H_29_N_3_O_5_ [M + H]^+^ = 380.2185, measured = 380.2177.

### 6-(((2*S*,3*S*)-2-((*tert*-Butoxycarbonyl)­amino)-3-methylpentanamido)­methyl)­picolinic Acid (**13**)

To a solution of ester **12** (2.40 g, 6.32 mmol, 1.00 equiv) was added a 0.5 M solution of LiOH in THF:MeOH:H_2_O (4:1:1; 30 mL) in an ice bath. The reaction temperature was allowed to rise to room temperature while stirring. After 2 h, the excess solvent was removed in vacuo, and the reaction pH was lowered to 5 using 0.1 M HCl followed by extraction with CH_2_Cl_2_ (3 × 30 mL). The combined organic layers was dried over MgSO_4_, filtered and dried over high vacuum to afford the title compound. Yield: 95% (2.20 g). ^1^H NMR (500 MHz, DMSO-*d*_6_) δ 8.96 (s, 1H), 8.11–7.78 (m, 2H), 7.46 (dd, *J* = 7.1, 1.6 Hz, 1H), 7.17 (s, 1H), 4.52–4.28 (m, 2H), 3.82 (t, *J* = 8.3 Hz, 1H), 1.78–1.67 (m, 1H), 1.51–1.40 (m, 1H), 1.34 (s, 9H), 1.19–1.04 (m, 1H), 0.79 (dd, *J* = 8.7, 6.6 Hz, 6H). ^13^C NMR (126 MHz, DMSO) δ = 173.57, 172.12, 166.82, 155.68, 137.95, 137.91, 123.79, 123.79, 122.52, 122.52, 77.92, 28.18, 24.67, 15.51, 10.93. HRMS (ESI): *m*/*z* calculated for C_19_H_29_N_3_O_6_ [M + H]^+^ = 366.2023, measured = 366.2023.

### 2-(((2*S*,3*S*)-2-((*tert*-Butoxycarbonyl)­amino)-3-methylpentanamido)­methyl)-6-(methoxycarbonyl)­pyridine 1-Oxide (**14**)

*m*-Chloroperoxybenzoic acid (0.510 g, 2.96 mmol, 1.02 equiv) was added to a solution of pyridine **12** (1.10 g, 2.90 mmol, 1.00 equiv) in a minimum amount of CH_2_Cl_2_ required for dissolution at 0 °C. The reaction temperature was allowed to rise to room temperature and the solution was stirred overnight. The solution was purified by silica column chromatography (0–15% CH_2_Cl_2_/MeOH) to afford the title compound as a white solid. Yield: 82% (0.945 g). ^1^H NMR (500 MHz, DMSO-*d*_6_) δ 8.54 (t, *J* = 6.0 Hz, 1H), 7.63 (dd, *J* = 7.8, 2.0 Hz, 1H), 7.50 (d, *J* = 5.9 Hz, 1H), 7.37 (t, *J* = 7.9 Hz, 1H), 7.00 (d, *J* = 8.1 Hz, 1H), 4.41 (dd, *J* = 17.6, 6.2 Hz, 1H), 4.29 (dd, *J* = 17.7, 5.8 Hz, 1H), 3.87 (s, 3H), 3.81 (t, *J* = 8.0 Hz, 1H), 1.77–1.67 (m, 1H), 1.55–1.41 (m, 1H), 1.40 (s, 9H), 1.20–1.06 (m, 1H), 0.82 (t, *J* = 7.2 Hz, 6H). ^13^C NMR (126 MHz, DMSO) δ = 172.59, 162.33, 155.81, 149.27, 141.61, 124.85, 124.71, 123.43, 78.20, 59.28, 53.00, 37.92, 35.57, 29.61, 28.20, 24.96, 24.68, 15.48, 10.87. HRMS (ESI): *m*/*z* calculated for C_19_H_29_N_3_O_6_ [M + H]^+^ = 396.2135, measured = 396.2129.

### 2-(((2*S*,3*S*)-2-((*tert*-Butoxycarbonyl)­amino)-3-methylpentanamido)­methyl)-6-carboxypyridine 1-Oxide (**15**)

To a solution of ester **14** (0.900 g, 2.28 mmol, 1.00 equiv) was added a 0.5 M solution of LiOH in THF:MeOH:H_2_O (4:1:1; 30 mL) in an ice bath. The reaction temperature was allowed to rise to room temperature while stirring. After 2 h, the excess solvent was removed in vacuo, and the reaction pH was lowered to 5 using 0.1 M HCl followed by extraction with CH_2_Cl_2_ (3 × 30 mL). The combined organic layers were dried over MgSO_4_, filtered and dried over high vacuum to afford the title compound as a white solid. Yield: 52% (0.451 mg) ^1^H NMR (500 MHz, DMSO-*d*_6_) δ 12.91 (s, 1H), 8.46 (t, J = 6.0 Hz, 1H), 7.80 (dt, J = 7.7, 1.5 Hz, 1H), 7.49 (d, J = 7.6 Hz, 1H), 7.41 (t, J = 7.6 Hz, 1H), 6.74 (d, J = 8.9 Hz, 1H), 4.48 – 4.22 (m, 2H), 3.82 (t, J = 8.2 Hz, 1H), 1.73 – 1.63 (m, 1H), 1.37 (s, 9H), 1.08 (dtd, J = 13.4, 8.6, 8.1, 5.7 Hz, 1H), 0.79 (t, J = 7.5 Hz, 6H). ^13^C NMR (126 MHz, DMSO) δ = 172.11, 167.74, 155.87, 140.50, 132.17, 128.88, 128.67, 128.19, 78.44, 59.36, 42.19, 28.64, 24.90, 15.91, 11.41. HRMS (ESI): *m*/*z* calculated for C_18_H_27_N_3_O_6_ [M + H]^+^ = 382.1973, measured = 382.1973.

### *tert*-Butyl (6-Methylpyridin-2-yl)­carbamate (**16**)

*N*,*N*-Dimethylpyridin-4-amine (5.65 g, 46.2 mmol, 1.00 equiv) was added a solution of 6-methylpyridin-2-amine (5.00 g, 46.2 mmol, 1.00 equiv) and Boc_2_O in *t*-BuOH (50 mL) at 0 °C while stirring and the reaction temperature was raised to 50 °C. After 16 h, the reaction temperature was lowered to room temperature and the solvent was removed under reduced pressure. The residue was dissolved in EtOAc (50 mL) and washed with a saturated solution of NaHCO_3_ and brine. The organic layer was dried over MgSO_4_ and concentrated in vacuo. The crude oil was purified by silica column chromatography (0–5% EtOAc/Petroleum ether) to afford the title compound as a white solid. Yield: 46% (4.47 g) ^1^H NMR (500 MHz, DMSO-*d*_6_) δ 9.57 (s, 1H), 7.82–7.13 (m, 1H), 7.07–6.70 (m, 1H), 2.35 (s, 3H), 1.45 (s, 9H). ^13^C NMR (126 MHz, DMSO) δ 156.30, 152.78, 151.80, 138.20, 117.50, 109.29, 79.36, 28.03, 23.65. HRMS (ESI): *m*/*z* calculated for C_11_H_16_N_2_O_2_ [M + H]^+^ = 209.1285, measured = 209.1285.

### Ethyl 2-(6-((*tert*-Butoxycarbonyl)­amino)­pyridin-2-yl)­acetate (**17**)

LDA (2M, 23.6 mL, 47.3 mmol, 4.00 equiv) was added dropwise to cooled solution of **16** (2.50 g, 11.8 mmol, 1.00 equiv) in anh. THF (20 mL) at −78 °C while stirring. After 30 min, diethyl carbonate (2.86 mL, 23.6 mmol, 2.00 equiv) was added dropwise and stirred for a further 10 min. The temperature was raised to 0 °C. After 2 h the reaction was quenched with a saturated NH_4_Cl solution and extracted with EtOAc (30 mL × 3) and the combined organic layers were dried over MgSO_4_. After concentration in vacuo the crude product was purified by silica column chromatography (0–30% EtOAc/petroleum ether) to afford the title compound as a yellow oil. Yield: 74% (2.50 g)^1^H NMR (500 MHz, DMSO-*d*_6_) δ 9.71 (s, 1H), 7.91–7.50 (m, 2H), 6.97 (dd, *J* = 4.8, 3.5 Hz, 1H), 4.07 (q, *J* = 7.1 Hz, 2H), 3.71 (s, 2H), 1.45 (s, 9H), 1.17 (t, *J* = 7.1 Hz, 3H). ^13^C NMR (126 MHz, DMSO) δ 170.28, 152.97, 152.80, 152.02, 138.54, 118.35, 110.68, 79.45, 60.36, 42.87, 28.02, 14.09. HRMS (ESI): *m*/*z* calculated for C_14_H_20_N_2_O_4_ [M + H]^+^ = 281.1496, measured = 281.1495.

### 2-Amino-6-(2-ethoxy-2-oxoethyl)­pyridine 1-Oxide (**18**)

*m*-CPBA (1.82 g, 10.53 mmol, 1.00 equiv) was added to a solution of **17** (2.46 g, 8.78 mmol, 1.00 equiv) in a minimum amount of CH_2_Cl_2_ required for dissolution and cooled in an ice bath. The reaction temperature was allowed to rise to room temperature. After 7 h, excess CH_2_Cl_2_ was blown off under a stream of argon and the solution was purified by silica column chromatography (0–30% EtOAc/petroleum ether) to afford the title compound as a yellow oil. Yield: 94% (2.44 g) ^1^H NMR (500 MHz, DMSO-*d*_6_) δ 9.34 (s, 1H), 7.96 (dd, *J* = 8.5, 1.9 Hz, 1H), 7.41 (t, *J* = 8.1 Hz, 1H), 7.24 (dd, *J* = 7.8, 1.9 Hz, 1H), 4.07 (q, *J* = 7.0 Hz, 2H), 3.89 (s, 2H), 1.50 (s, 9H), 1.17 (t, *J* = 7.1 Hz, 3H).^13^C NMR (126 MHz, DMSO) δ 168.86, 151.52, 144.20, 143.83, 127.08, 120.13, 111.91, 82.19, 60.87, 37.34, 28.18, 14.53. HRMS (ESI): *m*/*z* calculated for C_14_H_20_N_2_O_5_ [M + H]^+^ = 297.1445, measured = 297.1447.

### Ethyl 2-(6-Aminopyridin-2-yl)­acetate (**19**)

A round-bottom flask containing **18** (2.44 g, 8.23 mmol, 1.00 equiv) in an ice bath was charged with a solution of 30% TFA/CH_2_Cl_2_ (30 mL, 119.34 mmol, 15.0 equiv) dropwise. The reaction temperature was allowed to rise to room temperature while stirring. After 1 h, the volatiles were removed under a stream of argon and subsequently, in vacuo. The product was used in the next reaction without further purification.

### 2-((2*S*,3*S*)-2-((*tert*-Butoxycarbonyl)­amino)-3-methylpentanamido)-6-(2-ethoxy-2-oxoethyl)­pyridine 1-Oxide (**20**)

Compound **19** was dissolved in CH_2_Cl_2_ (30 mL) and the flask was placed in an ice bath followed by the addition of DIPEA (8.60 mL, 49.4 mmol, 6.00 equiv), Boc-Ile-OH (3.81 g, 16.46 mmol, 2.00 equiv), and PyBOP (8.57 g, 16.46 mmol, 2.00 equiv) while stirring and the reaction temperature was allowed to rise to room temperature. After 16 h, the solution was washed with NaHCO_3_, NH_4_Cl and brine. The organic phase was dried over MgSO_4_, and concentrated in vacuo to give the crude product as a yellow oil which was purified by silica column chromatography (0–30% EtOAc/petroleum ether) to afford the title compound as a yellow oil. Yield: 74% (2.50 g) over 2 steps. ^1^H NMR (500 MHz, DMSO-*d*_6_) δ 10.61 (s, 1H), 8.27 (dd, *J* = 8.5, 1.9 Hz, 1H), 7.54 (d, *J* = 7.6 Hz, 1H), 7.41 (t, *J* = 8.1 Hz, 1H), 7.29 (dd, *J* = 7.8, 1.9 Hz, 1H), 4.16–4.00 (m, 3H), 3.89 (s, 2H), 1.54–1.40 (m, 1H), 1.38 (s, 9H), 1.33–1.24 (m, 1H), 1.22 (ddd, *J* = 13.7, 6.8, 2.5 Hz, 1H), 1.16 (t, *J* = 7.1 Hz, 3H), 0.87 (d, *J* = 6.9 Hz, 3H), 0.82 (t, *J* = 7.4 Hz, 3H). ^13^C NMR (126 MHz, DMSO) δ 172.17, 168.86, 156.32, 144.36, 143.42, 126.87, 121.15, 113.26, 79.09, 60.84, 37.43, 36.32, 28.67, 28.62, 24.97, 15.88, 14.56, 14.51, 11.73, 11.62. HRMS (ESI): *m*/*z* calculated for C_20_H_31_N_3_O_6_ [M + H]^+^ = 410.2291, measured = 410.2284.

### Ethyl 2-(6-((2*S*,3*S*)-2-((*tert*-Butoxycarbonyl)­amino)-3-methylpentanamido)­pyridin-2-yl)­acetate (**21**)

Synthesized according to the method for compound **4** using **20** (3.25 g, 7.93 mmol, 1.00 equiv) and bis­(pinacolato)­diboron (3.02 g, 11.9 mmol, 1.50 equiv) to provide a yellow oil. Yield: 86% (6.85 g) ^1^H NMR (500 MHz, DMSO-*d*_6_) δ 10.37 (s, 1H), 7.98 (d, *J* = 8.4 Hz, 1H), 7.74 (t, *J* = 7.9 Hz, 1H), 7.07 (dd, *J* = 7.5, 0.8 Hz, 1H), 6.98 (d, *J* = 8.6 Hz, 1H), 4.09 (qd, *J* = 7.1, 1.3 Hz, 3H), 3.76 (s, 2H), 1.85 – 1.63 (m, 1H), 1.55 – 1.41 (m, 1H), 1.37 (s, 9H), 1.33 – 1.23 (m, 1H), 1.17 (d, *J* = 6.4 Hz, 3H), 0.84 (d, *J* = 6.8 Hz, 3H), 0.81 (t, *J* = 7.4 Hz, 3H). ^13^C NMR (126 MHz, DMSO) δ 172.40, 170.69, 155.91, 153.66, 151.74, 139.35, 120.06, 112.20, 83.32, 78.53, 60.86, 43.27, 36.69, 28.65, 25.30, 24.96, 15.78, 14.54, 11.32. HRMS (ESI): *m*/*z* calculated for C_20_H_31_N_3_O_5_ [M + H]^+^ = 394.2336, measured = 394.2335.

### 2-(6-((2*S*,3*S*)-2-((*tert*-Butoxycarbonyl)­amino)-3-methylpentanamido)­pyridin-2-yl)­acetic Acid (**22**)

To a solution of ester **21** (2.90 g, 6.84 mmol, 1.00 equiv) was added a 0.5 M solution of LiOH in THF:MeOH:H_2_O (4:1:1; 30 mL) in an ice bath. The reaction temperature was allowed to rise to room temperature while stirring. After 2 h, the excess solvent was removed in vacuo, and the reaction pH was lowered to 5 using 0.1 M HCl followed by extraction with CH_2_Cl_2_ (3 × 30 mL). The combined organic layer was dried over MgSO_4_, filtered and dried over high vacuum to afford the title compound as a white solid. Yield: 96% (2.40 g) ^1^H NMR (700 MHz, DMSO-*d*_6_) δ 10.31 (s, 1H), 7.88 (d, *J* = 8.3 Hz, 1H), 7.68 (t, *J* = 7.9 Hz, 1H), 7.01 – 6.93 (m, 2H), 4.11 – 4.04 (m, 1H), 3.69 (s, 2H), 1.76 (d, *J* = 8.3 Hz, 1H), 1.56 – 1.42 (m, 1H), 1.38 (s, 9H), 1.15 (dt, *J* = 15.0, 7.7 Hz, 1H), 0.85 (d, *J* = 6.8 Hz, 3H), 0.82 (t, *J* = 7.4 Hz, 3H). ^13^C NMR (176 MHz, DMSO) δ = 172.18, 156.86, 155.89, 151.39, 139.21, 119.22, 110.92, 78.55, 59.94, 43.50, 36.76, 28.66, 24.98, 15.79, 11.35. HRMS (ESI): *m*/*z* calculated for C_18_H_27_N_3_O_5_ [M + H]^+^ = 366.2029, measured = 366.2024.

### 2-((2*S*,3*S*)-2-((*tert*-Butoxycarbonyl)­amino)-3-methylpentanamido)-6-(carboxymethyl)­pyridine 1-Oxide (**23**)

To a solution of ester **20** (3.24 g, 7.93 mmol, 1.00 equiv) was added a 0.5 M solution of LiOH in THF:MeOH:H_2_O (4:1:1; 50 mL) in an ice bath. The reaction temperature was allowed to rise to room temperature while stirring. After 2 h, the excess solvent was removed in vacuo, and the reaction pH was lowered to 5 using 0.1 M HCl followed by extraction with CH_2_Cl_2_ (3 × 30 mL). The combined organic layers were dried over MgSO_4_, filtered and dried over high vacuum to afford the title compound as a white solid. Yield: 43% (1.31 g) ^1^H NMR (500 MHz, DMSO-*d*_6_) δ 12.53 (s, 1H), 10.63 (s, 1H), 8.25 (dd, *J* = 8.4, 1.9 Hz, 1H), 7.54 (d, *J* = 7.8 Hz, 1H), 7.40 (t, *J* = 8.1 Hz, 1H), 7.28 (dd, *J* = 7.9, 1.9 Hz, 1H), 4.11 (t, *J* = 7.3 Hz, 1H), 3.83 (s, 2H), 1.96–1.82 (m, 1H), 1.50–1.40 (m, 1H), 1.38 (s, 9H), 1.31–1.16 (m, 1H), 0.87 (d, *J* = 6.8 Hz, 3H), 0.85–0.79 (m, 3H). ^13^C NMR (126 MHz, DMSO) δ = 173.61, 171.68, 169.87, 155.72, 144.37, 126.41, 120.71, 112.66, 78.00, 58.12, 37.09, 35.91, 28.24, 24.52, 15.46, 11.18. HRMS (ESI): *m*/*z* calculated for C_18_H_27_N_3_O_6_ [M + H]^+^ = 382.1978, measured = 382.1973.

### (3-Carboxyphenyl)­methanaminium 2,2,2-Trifluoroacetate (**24**)

A 100 mL round bottomed flask under argon atmosphere was charged with 10% Pd/C (0.72 g, 0.68 mmol, 0.1 equiv) followed by 3 evacuation and argon refill cycles. The catalyst was then suspended in MeOH (30 mL) and charged with a solution of 3-cyanobenzoic acid (1.00 g, 6.80 mmol, 1.00 equiv) in a minimum amount of MeOH required for complete dissolution followed by 3 evacuation and H_2_ refill cycles. The reaction mixture was charged with 37% HCl (2.00 mL, 20.4 mmol, 3.0 equiv) while stirring under H_2_. After 16 h, the suspension was filtered using a bed of Celite, concentrated in vacuo and the intermediate was purified by reverse phase flash column chromatography (0–95% H_2_O in methanol with both eluents containing 0.1% TFA). Yield: 42% (5.36 g) as a white solid. ^1^H NMR (700 MHz, DMSO-*d*_6_) δ 13.10 (s, 1H), 8.55 (s, 2H), 8.09 (s, 1H), 7.93 (d, *J* = 7.8 Hz, 1H), 7.76 (d, *J* = 7.7 Hz, 2H), 7.54 (t, *J* = 7.7 Hz, 1H), 4.09 (s, 2H). ^13^C NMR (176 MHz, DMSO) δ 166.97, 134.61, 133.54, 131.08, 129.97, 129.26, 128.87, 41.78. HRMS (ESI): *m*/*z* calculated for C_8_H_9_NO_2_ [M + H]^+^ = 152.0712, measured = 152.0704.

### 3-((((9H-Fluoren-9-yl)­methoxy)­carbonyl)­amino)­benzoic Acid (**25**)

To a solution of 3-aminobenzoic acid (2.00 g, 14.6 mmol, 1.00 equiv) and NaHCO_3_ (2.45 g, 29.16 mmol, 2.00 equiv) in a minimum amount of water required for complete dissolution and cooled in an ice bath, was added a solution of Fmoc-OSu (5.90 g, 17.5 mmol, 1.20 equiv) in MeCN dropwise while stirring. After 20 min, the ice bath was removed, and additional MeCN was added if precipitation was observed. After 4 h, the reaction was extracted with EtOAc (30 mL × 3). The combined organic phase was extracted with a saturated solution of NaHCO_3_ (30 mL × 1). The combined aqueous layer was acidified with 2 M HCl to a pH range of 1–3; extracted with EtOAc (30 mL × 3), washed with saturated NaHCO_3_ and brine. The combined organic layer was dried over MgSO_4_, filtered, and concentrated in vacuo to afford the title compound as a white solid. Yield: 62% (3.23 g). Compound characterization matched previously reported values.^46^

### 3-(((((9H-Fluoren-9-yl)­methoxy)­carbonyl)­amino)­methyl)­benzoic Acid (**26**)

To a solution of benzoic acid **24** (0.985 mg, 6.65 mmol, 1.00 equiv) and NaHCO_3_ (1.10 g, 13.0 mmol, 2.00 equiv) in a minimum amount of water required for complete dissolution and cooled in an ice bath, was added a solution of Fmoc-OSu (2.64 g, 7.82 mmol, 1.2 equiv) in MeCN dropwise while stirring. After 20 min, the ice bath was removed, and additional MeCN was added if precipitation was observed. After 4 h, the reaction was extracted with EtOAc (30 mL × 3). The combined organic phase was extracted with a saturated solution of NaHCO_3_ (30 mL × 1). The combined aqueous layer was acidified with 2 M HCl to a pH range of 1–3; extracted with EtOAc (30 mL × 3), washed with saturated NaHCO_3_ and brine. The combined organic layer was dried over MgSO_4_, filtered, and concentrated in vacuo to afford the title compound as a white solid. Yield: 21% (500 mg) HRMS (ESI): *m*/*z* calculated for C_23_H_19_NO_4_ [M + H]^+^ = 374.1392, measured = 374.1384.

### 2-(3-((((9H-Fluoren-9-yl)­methoxy)­carbonyl)­amino)­phenyl)­acetic Acid (**27**)

To a solution of 2-(3-aminophenyl)­acetic acid (570 mg, 3.77 mmol, 1.00 equiv) and NaHCO_3_ (634 mg, 7.54 mmol, 2.00 equiv) in a minimum amount of water required for complete dissolution and cooled in an ice bath, was added a solution of Fmoc-OSu (4.53 g, 4.52 mmol, 1.2 equiv) in MeCN dropwise while stirring. After 20 min, the ice bath was removed, and additional MeCN was added if precipitation was observed. After 4 h, the reaction was extracted with EtOAc (30 mL × 3). The combined organic phase was extracted with a saturated solution of NaHCO_3_ (30 mL × 1). The combined aqueous layer was acidified with 2 M HCl to a pH range of 1 – 3; extracted with EtOAc (30 mL × 3), washed with saturated NaHCO_3_ and brine. The combined organic layer was dried over MgSO_4_, filtered, and concentrated in vacuo to afford the title compound as a white solid. Yield: 96% (1.35 g) ^1^H NMR (700 MHz, DMSO-*d*_6_) δ 9.71 (s, 1H), 7.90 (d, *J* = 7.5 Hz, 2H), 7.75 (d, *J* = 7.5 Hz, 2H), 7.43 (t, *J* = 7.4 Hz, 2H), 7.39 (s, 1H), 7.35 (td, *J* = 7.4, 1.1 Hz, 3H), 7.20 (t, *J* = 8.0 Hz, 1H), 6.88 (d, *J* = 7.5 Hz, 1H), 4.45 (d, *J* = 6.9 Hz, 2H), 4.30 (t, *J* = 6.8 Hz, 1H), 3.49 (s, 2H). ^13^C NMR (176 MHz, DMSO) δ 172.65, 153.48, 143.84, 140.86, 139.05, 135.62, 128.71, 127.79, 127.22, 125.23, 123.68, 120.25, 119.24, 118.17, 116.75, 65.67, 46.68. LRMS (ESI): *m*/*z* calculated for C_23_H_19_NO_4_ [M + H]^+^ = 374.1392, measured = 209.1285.

### Ethyl 2-((1*S*,2*S*)-1-((*tert*-Butoxycarbonyl)­amino)-2-methylbutyl)­thiazole-4-carboxylate (**28**)

Synthesis was performed in accordance to the method described by Nielsen and coworkers.^29^

**Yield**: 3.91 g (10.6 mmol, 48%) yellow solid. ^1^H NMR (600 MHz, DMSO-*d*_6_) δ 8.41 (s, 1H), 7.73 (d, *J* = 8.4 Hz, 1H), 4.65 (t, *J* = 7.7 Hz, 1H), 4.29 (dtt, *J* = 10.8, 7.1, 3.8 Hz, 2H), 1.99 – 1.88 (m, 1H), 1.55 – 1.42 (m, 1H), 1.38 (s, 9H), 1.30 (t, *J* = 7.1 Hz, 3H), 1.28 – 1.19 (m, 1H), 0.84 – 0.79 (m, 3H), 0.77 (d, *J* = 6.8 Hz, 3H). ^13^C NMR (151 MHz, DMSO) δ 174.66, 160.70, 155.65, 145.48, 128.74, 78.39, 60.61, 57.36, 38.32, 28.11, 24.50, 15.52, 14.14, 11.28. HRMS (ESI): *m*/*z* calculated for C_16_H_26_N_2_O_4_S [M + H]^+^ = 343.1692, measured = 343.1686.

### 2-((1*S*,2*S*)-1-((tert-Butoxycarbonyl)­amino)-2-methylbutyl)­thiazole-4-carboxylic acid (**29**)

Synthesis was performed in accordance to the method described by Nielsen and coworkers.^29^

**Yield**: 220 mg (0.56 mmol, 35%) yellow solid. ^1^H NMR (600 MHz, DMSO-*d*_6_) δ 12.97 (s, 1H), 8.33 (s, 1H), 7.71 (d, *J* = 8.4 Hz, 1H), 4.65 (t, *J* = 7.7 Hz, 1H), 2.03 – 1.79 (m, 1H), 1.65 – 1.43 (m, 1H), 1.38 (s, 9H), 1.31 – 1.20 (m, 1H), 0.83 (t, *J* = 7.2 Hz, 3H), 0.78 (d, *J* = 6.8 Hz, 3H).^13^C NMR (151 MHz, DMSO) δ 174.24, 162.05, 155.39, 146.60, 128.22, 57.31, 38.35, 28.10, 24.51, 15.51, 10.93. HRMS (ESI): *m*/*z* calculated for C_14_H_22_N_2_O_4_S [M + H]^+^ = 315.1379, measured = 315.1374.

### Synthesis of Linear Precursor NH_2_-Ala-Phe-Pro-Ile-Pro-CTC (**30**)

Dry peptidyl resin (3.0 mmol) was swollen in CH_2_Cl_2_ for 20 min in a 20 mL fritted syringe reactor. The solvent was drained and a solution of Fmoc-Proline–OH (4.05 g, 12.00 mmol, 4.0 equiv) and DIPEA (4.180 mL, 24.00 mmol, 8.0 equiv) in anhydrous CH_2_Cl_2_ (10 mL) was drawn into the syringe and shaken. After 2 h, the reaction mixture was drained and the resin was washed with CH_2_Cl_2_ (2 × 30 s), DMF (4 × 30 s). The resin was then washed with CH_2_Cl_2_ (10 mL × 4 × 30 s) followed by addition of CH_2_Cl_2_/MeOH/DIPEA (80:15:15, 10 mL). After 30 min of shaking, the capped resin was washed with DMF (10 mL × 4 × 30 s), CH_2_Cl_2_ (10 mL × 4 × 30 s), Et_2_O (10 mL × 1 × 1 min) and dried under high vacuum followed by spectroscopic quantification of the actual resin loading by Fmoc release. Fmoc protected amino acids (4 equiv) were coupled using PyBOP (4 equiv) and DIPEA (8 equiv) in DMF for 30 min at room temperature. Deprotection of the Fmoc protecting group was carried out using a 20% solution of piperidine in DMF for 5 min followed by addition of fresh reagents and further reaction for 10 min.

### Synthesis of Linear Precursor NH_2_ **-**tBuGly-Phe-Pro-Ile-Pro-CTC (31)

Synthesis performed as for linear peptide **30**.

### Synthesis of Peptides SA-A1, SA-A2, SA-B1, and SA-B2

A portion of the peptidyl resin containing the linear precursor **30** described above was shaken with the respective linker fragments **29**, **31**, **5**, or **6** (2.00 equiv), PyBOP (2.00 equiv), and DIPEA (4.00 equiv) in DMF at room temperature for 16 h. Peptidyl resin was then washed with DMF (10 mL × 4 × 30 s) and CH_2_Cl_2_ (10 mL × 4 × 30 s). Next, the peptides were cleaved using 20% TFA in CH_2_Cl_2_ (10 mL) for 1 h, and the volatiles were removed under a stream of Argon followed by coevaporation to dryness with CHCl_3_ (10 mL × 3). The linear peptides were dissolved in CH_2_Cl_2_ (to a 500 μM concentration) followed by the addition of PyBOP (2.00 equiv) and DIPEA (4.00 equiv) while stirring at rt. After 16 h, the solvent was removed in vacuo and the residue dissolved in a minimum amount of 50% MeCN in H_2_O required for dissolution. Peptides were purified by preparative HPLC on a Büchi Pure C-850 Flash Prep system equipped with a 50 mm × 10 mm, 5 μm, Macherey-Nagel C18 Gravity column, eluting at 6 mL/min with a binary mixture of MeCN and H_2_O with both containing 0.1% TFA using a gradient of 5% – 95% MeCN over 60 min.

### Synthesis of Peptides SA-C1 and SA-C2

A portion of the peptidyl resin containing the linear precursor **30** described above was shaken with the respective linker fragments **13**, or **15** (2.00 equiv), HATU (2.00 equiv) and DIPEA (4.00 equiv) in DMF at room temperature for 16 h. Peptidyl resin was then washed with DMF (10 mL × 4 × 30 s) and CH_2_Cl_2_ (10 mL × 4 × 30 s). Next, the peptides were cleaved using 20% TFA in CH_2_Cl_2_ (10 mL) for 1 h, and the volatiles were removed under a stream of Argon followed by coevaporation to dryness with CHCl_3_ (10 mL × 3). The linear peptides were dissolved in CH_2_Cl_2_ (to a 500 μM concentration) followed by the addition of PyBOP (2.00 equiv) and DIPEA (4.00 equiv) while stirring at rt. After 16 h, the solvent was removed in vacuo and the residue dissolved in a minimum amount of 50% MeCN in H_2_O required for dissolution. Peptides were purified by preparative HPLC on a Büchi Pure C-850 Flash Prep system equipped with a 50 mm × 10 mm, 5 μm, Macherey-Nagel C18 Gravity column, eluting at 6 mL/min with a binary mixture of MeCN and H_2_O with both containing 0.1% TFA using a gradient of 5–95% MeCN over 60 min.

### Synthesis of Peptide SA-D1 and SA-D2

A portion of the peptidyl resin containing the linear precursor **30** described above was treated with the respective linker fragments **22**, or **23** (2.00 equiv), PyBOP (2.00 equiv) and DIPEA (4.00 equiv) in DMF at 80 °C for 1 h in a CEM Discover SP microwave reactor in a 10 mL glass vial fitted with an ActiVent cap. Peptidyl resin was then washed with DMF (10 mL × 4 × 30 s) and CH_2_Cl_2_ (10 mL × 4 × 30 s). Next, the peptides were cleaved using 20% TFA in CH_2_Cl_2_ (10 mL) for 1 h, and the volatiles were removed under a stream of Argon followed by coevaporation to dryness with CHCl_3_ (10 mL × 3). The linear peptides were dissolved in CH_2_Cl_2_ (to a 500 μM concentration) followed by the addition of PyBOP (2.00 equiv) and DIPEA (4.00 equiv) while stirring at rt. After 16 h, the solvent was removed in vacuo and the residue dissolved in a minimum amount of 50% MeCN in H_2_O required for dissolution. Peptides were purified by preparative HPLC on a Büchi Pure C-850 Flash Prep system equipped with a 50 mm × 10 mm, 5 μm, Macherey-Nagel C18 Gravity column, eluting at 6 mL/min with a binary mixture of MeCN and H_2_O with both containing 0.1% TFA using a gradient of 5% – 95% MeCN over 60 min.

### Synthesis of Peptide SA-A3

Resin loading and peptide synthesis were performed as for peptide **30** to afford the linear heptapeptide NH_2_–Ile-Cys­(Trt)-Ala-Phe-Pro-Ile-Pro-CTC. Peptide cleavage was performed by shaking a suspension of the peptidyl resin in HFIP/CH_2_Cl_2_ (1/4 v/v, 6 mL) for 1 h, followed by filtration and concentration under reduced pressure. The resulting oil was suspended in CH_2_Cl_2_ at a 0.25 mM concentration, followed by the addition of PyBOP (2.00 equiv) and DIPEA (4.00 equiv) and stirred overnight. The reaction mixture was then concentrated in vacuo and global cleavage was performed by treatment with TFA/ODT/TIPS/H_2_O (90/2.5/2.5/5 v/v) for 1 h. The peptide was then precipitated by adding the solution to cold Et_2_O and pelleted by centrifugation. The supernatant was removed, and the pellet resuspended in cold Et_2_O followed by centrifugation. This procedure was repeated twice. The resulting pellet was dissolved in H_2_O/MeCN (1:1) and lyophilized. The resulting solid was dissolved in a minimum amount of 50% MeCN in H_2_O required for dissolution. The peptide was purified by preparative HPLC on an Infinity II LC-MS system (Agilent Technologies, USA) equipped with a 50 mm × 10 mm, 5 μm, Macherey-Nagel C18 Gravity column, eluting at 6 mL/min with a binary mixture of MeCN and H_2_O with both containing 0.1% TFA using a gradient of 5% – 95% MeCN over 60 min.

### Synthesis of Peptides SA-B3, SA-C3, and SA-D3

A portion of the peptidyl resin containing the linear precursor **30** described above was shaken with the respective linker fragments **25**, **26**, or **27** (2.00 equiv), PyBOP (2.00 equiv), COMU (1.00 equiv) and DIPEA (4.00 equiv) in DMF at room temperature for 16 h. After Fmoc deprotection using 20% piperidine in DMF (1 × 5 min, 1 × 10 min). Boc-Ile-OH (2.00 equiv) was then coupled using PyBOP (2.00 equiv) and DIPEA (4.00 equiv) in DMF. Peptidyl resin was then washed with DMF (10 mL × 4 × 30 s) and CH_2_Cl_2_ (10 mL × 4 × 30 s). Next, the peptides were cleaved using 20% TFA in CH_2_Cl_2_ (10 mL) for 1 h, and the volatiles were removed under a stream of Argon followed by coevaporation to dryness with CHCl_3_ (10 mL × 3). The linear peptides were dissolved in CH_2_Cl_2_ (to a 500 μM concentration) followed by the addition of PyBOP (2.00 equiv) and DIPEA (4.00 equiv) while stirring at rt. After 16 h, the solvent was removed in vacuo and the residue was dissolved in a minimum amount of 50% MeCN in H_2_O as required for dissolution. Peptides were then purified by preparative HPLC on a Büchi Pure C-850 Flash Prep system equipped with a 50 mm × 10 mm, 5 μm, Macherey-Nagel C18 Gravity column, eluting at 6 mL/min with a binary mixture of MeCN and H_2_O with both containing 0.1% TFA using a gradient of 5% – 95% MeCN over 60 min.

### CHI-IAM Chromatography

CHI-IAM chromatography was carried out on an Agilent 1200 Series LC (Agilent Technologies, USA) equipped with a 4.6 mm × 100 mm to 150 mm IAM.PC.DD2 column from Regis Technologies, Inc., eluting at 1.5 mL/min, eluting over a gradient of 0% – 85% MeCN with a 50 mM ammonium acetate buffer adjusted to pH 7.4 over 4.75 min.

### Caco-2 AB Permeability

Transepithelial transport of the compounds through a Caco-2 monolayer was determined in an automated fashion using a Tecan Freedom EVO 200 equipped with a Te-MO 96 and a TEER Station. Caco-2 cells were grown in DMEM supplemented with 10% FCS for 15–19 days on Costar 24-well cell culture cluster plates (polycarbonate membrane, 0.4 μm pore size). Chamber volumes were 288 and 950 μL on the apical and basolateral sides of the cell monolayers, respectively, and all incubations were performed with prewarmed buffers in a shaking incubator at 480 rpm and 37 °C. Prior to assay, cells were washed with HBSS supplemented with 25 mM HEPES (HBSS-HEPES), pH 7.4, to remove the culture medium. After 15 min equilibration time the transepithelial electrical resistance (TEER) was determined to assess acceptance of the cell plates into the assay. A second measurement and a lucifer yellow leakage determination was carried out after performing all the transport experiments to monitor integrity of the cell monolayers throughout the study. Permeability in the absorptive direction (A–B, apical-to-basolateral) was studied over 120 min at 10 μM test compound. The compound solutions were freshly prepared from DMSO stock solutions diluted into HBSS supplemented with 25 mM MES, pH 6.5, or into HBSS-HEPES, pH 7.4; the final solvent concentration was always 1%. Samples from the donor side (2 μL) were drawn immediately after addition of test compound and after 45 and 120 min. The donor samples were diluted 1:100 with 198 μL in HBSS. From the receiver compartment an amount of 200 μL is withdrawn after 45 and 120 min and replaced with fresh HBSS-HEPES, pH 7.4. Upon completion of the study all samples were quenched with 67 μL of acetonitrile and analyzed subsequently using UPLC/MS/MS. The permeability was determined as the appearance rate of compound on the receiver side, in relation to donor concentration, according to the following equation:

$$P_{app}=\frac{dQ}{\left(dt\right)\left(A\right)\left(D\right)\left(T\right)}$$

where d*Q*/[(d*t*)­(*T*)] is the slope of the permeation profile across the Caco-2 cell monolayers, *A* is the surface area of the Transwell insert (0.33 cm^2^), and *D* is the concentration on the donor side. Following the permeability experiments, recovery from the donor and receiver compartments was calculated from the following equation:

$$\%\mathrm{mass}\;\mathrm{recovery}=\frac{\left(D_{\mathrm{end}}V_{d}\right)+\left(R_{\mathrm{end}}V_{t}\right)}{D_{0}V_{d}}\times 100$$

where *D*_0_ and *D*_end_ are the donor sample concentrations at the beginning and last time points, respectively, *V*_d_ is the donor side volume (0.288 mL for A–B and 0.95 mL for B–A), *R*_end_ is the receiver sample concentration at the last time point, and *V*_r_ is the receiver volume (0.95 mL for A–B and 0.285 mL for B–A). All measurements were performed in duplicate, and only minor standard deviations ((0.08–1.14) × 10^–6^ cm/s) and on average moderate to good mass recovery values (82%) were observed. The experiment was performed in the presence of a cocktail of transporter inhibitors (Zosuquidar 10 μM, Benzbromarone 30 μM, KO-143 2 μM) to measure passive permeability.

### VT-NMR

Variable-temperature nuclear magnetic resonance (VT-NMR) measurements were conducted using a Bruker Avance Neo 600 (av600) spectrometer equipped with a CP2.1 TMO 600 S3 cryoprobe. The ^1^H NMR data was collected over a temperature range from 293.15 to 323.15 K, with temperature increments of 5 °C.

### H-D Exchange NMR

The samples were dissolved in DMSO-*d*_6_ and 20% D_2_O was added. Immediately after ^1^H NMR data was collected at 0, 10, 20, 30, and 240 min. The peak area was normalized to a peak of constant height.

### NMR Spectroscopy for Structural Modeling

NMR samples of SA-A1, SA-B1 and SA-B3 were prepared by dissolving the peptide (5–9 mg) in 500 μL of either *d*_6_-DMSO or CDCl_3_. NMR spectra were recorded on a 600 MHz Bruker spectrometer equipped with a cryoprobe. 2D NMR spectra were based on standard Bruker pulse programs: ROESY (derivated from roesyph), TOCSY (dipsi2esgpph) and COSY (cosyqf). ROESY spectra were acquired with 8192 complex data points over 8403–10823 Hz, with 1024 increments in F1 and 8 scans. Mixing times were comprised between 350 and 500 ms. TOCSY spectra were acquired with 4096 complex data points over 8196–9615 Hz, with 512 increments in F1, 16 scans and a mixing time of 80 ms.

Spectra were processed using NMRPipe software. Assignment and ROE intensities were obtained using CCPNMR software.

### Structure Calculations

Distance restraints used in calculating SA-A1, SA-B1, and SA-B3 structures in CDCl_3_ and DMSO were derived from ROESY spectra recorded at 298 K. Data is summarized in Table S2.

The amide bond conformations between 2Phe-3Pro and 4Ile-5Pro were determined by ROESY cross-peaks inspection. Strong 2PheHα-3ProΗα correlations were observable in all solutions thus asserting a cis conformation. On the contrary, strong 4IleHα-5ProΗδ were noticeable, thus upholding a trans conformation. Noticeably, 4IleHα-5ProΗα were also distinguishable in SA-B1 and SA-B3 solutions in DMSO. Additionally, multiple series of resonances observable in these solutions attest of a mixture of conformers. CYANA predictions, based on chemical shift differences between prolines Cβ and Cγ, were mostly in accordance with our spectral observations. Backbone φ torsion angles restraints were based on ^3^J_NHCH_ obtained from 1D NMR: −65 ± 30° for J_NHCH_ < 6 Hz, and −120 ± 30° for J_NHCH_ > 8 Hz. The calculations were performed using CYANA v3.98.1. Modifications of the macrocycle were adapted for CYANA from thiazole (SA-A1) and modified 3-(methylamino)­benzoic acid (SA-B1 and SA-B3) using the PDBeChem converting tool cylib. Final structures were visualized with Pymol and contained no distance violations >0.2 Å nor angle violations >8°. The statistics of the structure determination are reported in Table S2.

### Cell Painting

The described assay follows closely the method described by Bray et al.^25^

Initially, 5 μL U2OS medium were added to each well of a 384-well plate (Revvity PhenoPlate 384). Subsequently, U2OS cell were seeded with a density of 1600 cells per well in 20 μL medium. The plate was incubated for 10 min at ambient temperature, followed by an additional 4 h incubation (37 °C, 5% CO_2_). Compound treatment was performed with the Echo 520 acoustic dispenser (Beckman-Coulter) at final concentrations of 10 μM, 3 μM or 1 μM. Incubation with compound was performed for 20 h (37 °C, 5% CO_2_). Subsequently, mitochondria were stained with Mito Tracker Deep Red (Thermo Fisher Scientific, Cat. No. M22426). The Mito Tracker Deep Red stock solution (1 mM) was diluted to a final concentration of 100 nM in prewarmed medium. The medium was removed from the plate leaving 10 μL residual volume and 25 μL of the Mito Tracker solution were added to each well. The plate was incubated for 30 min in darkness (37 °C, 5% CO2). To fix the cells 7 μL of 18.5% formaldehyde in PBS were added, resulting in a final formaldehyde concentration of 3.7%. Subsequently, the plate was incubated for another 20 min in darkness (RT) and washed three times with 70 μL of PBS. (Agilent Washer Elx405). Cells were permeabilized by addition of 25 μL 0.1% Triton X-100 to each well, followed by 15 min incubation (RT) in darkness. The cells were washed three times with PBS leaving a final volume of 10 μL. To each well 25 μL of a staining solution were added, which contains 1% BSA, 5 μL/ml Phalloidin (Alexa594 conjugate, Thermo Fisher Scientific, A12381), 25 μg/mL Concanavalin A (Alexa488 conjugate, Thermo Fisher Scientific, Cat. No. C11252), 5 μg/mL Hoechst 33342 (Sigma, Cat. No. B2261–25 mg), 1.5 μg/mL WGA-Alexa594 conjugate (Thermo Fisher Scientific, Cat. No. W11262) and 1.5 μM SYTO 14 solution (Thermo Fisher Scientific, Cat. No. S7576). The plate is incubated for 30 min (RT) in darkness and washed three times with 70 μL PBS. After the final washing step, the PBS was not aspirated. The plates were sealed and centrifuged for 1 min at 50*g*.

The plates were prepared in triplicates with shifted layouts to reduce plate effects and imaged using a Micro XL High-Content Screening System (Molecular Devices) in 5 channels (DAPI: Ex350–400/Em410–480; FITC: Ex470–500/Em510–540; Spectrum Gold: Ex520–545/Em560–585; TxRed: Ex535–585/Em600–650; Cy5: Ex605–650/Em670–715) with 9 sites per well and 20× magnification (binning 2).

The generated images were processed with the *CellProfiler* package (https://cellprofiler.org/, version 3.0.0) on a computing cluster of the Max Planck Society to extract 1716 cell features per microscope site. The data was then further aggregated as medians per well (9 sites → 1 well), then over the three replicates.

Further analysis was performed with custom *Python* (https://www.python.org/) scripts using the *Pandas* (https://pandas.pydata.org/) and *Dask* (https://dask.org/) data processing libraries as well as the *Scientific Python* (https://scipy.org/) package (separate publication to follow).

From the total set of 1716 features, a subset of highly reproducible and robust features was determined using the procedure described by Woehrmann et al.^47^ in the following way: Two biological repeats of one plate containing reference compounds were analyzed. For every feature, its full profile over each whole plate was calculated. If the profiles from the two repeats showed a similarity ≥0.8 (see below), the feature was added to the set.

This procedure was only performed once and resulted in a set of 579 robust features out of the total of 1716 that was used for all further analyses.

The phenotypic profiles were compiled from the *Z*-scores of all individual cellular features, where the *Z*-score is a measure of how far away a data point is from a median value.

Specifically, *Z*-scores of test compounds were calculated relative to the Median of DMSO controls. Thus, the *Z*-score of a test compound defines how many MADs (Median Absolute Deviations) the measured value is away from the Median of the controls as illustrated by the following formula:

$$z-\mathrm{score}=\frac{value_{meas.}-Median_{\mathrm{Controls}}}{MAD_{\mathrm{Controls}}}$$

The phenotypic compound profile is then determined as the list of *Z*-scores of all features for one compound.

In addition to the phenotypic profile, an induction value was determined for each compound as the fraction of significantly changed features, in percent:

$$\mathrm{Induction}\left[\%\right]=\frac{\mathrm{number}\;\mathrm{of}\;\mathrm{features}\;\mathrm{with}\;\mathrm{abs}.\mathrm{values}>3}{\mathrm{total}\;\mathrm{number}\;\mathrm{of}\;\mathrm{features}}$$

Similarities of phenotypic profiles (termed *Biosimilarity*) were calculated from the correlation distances (CD) between two profiles (https://docs.scipy.org/doc/scipy/reference/generated/scipy.spatial.distance.correlation.html):

$$CD=1-\frac{\left(u-\mathrm{u̅}\right)\cdot \left(v-\mathrm{v̅}\right)}{{∥(u-\mathrm{u̅})∥}_{2}{∥(v-\mathrm{v̅})∥}_{2}}$$

where x̅ is the mean of the elements of *x*, *x*·*y* is the dot product of *x* and *y*, and *x*∥_2_ is the Euclidean norm of *x*:

$${∥x∥}_{2}=\sqrt{x_{1}^{2}+x_{2}^{2}+...+x_{n}^{2}}$$

The biosimilarity is then defined as

Biosimilarity=1−CD

Biosimilarity values smaller than 0 are set to 0 and the biosimilarity is expressed in percent (0–100).

## Supplementary Material





## Notes

Supplemental figures, VT-NMR spectra, 2D-NMR spectra for structural determination of peptides, CHI-IAM chromatograms, HPLC chromatograms of peptides, and ^1^H and ^13^C NMR spectra for all nonpeptide compounds (PDF)
Molecular formula strings (CSV)

## ⊥

Department of Organic Chemistry, Währinger Straße 38, 1090 Wien

## Abbreviations

2D-NMR: 2-dimensional nuclear magnetic resonance spectroscopy
Ala: alanine
Ar: aryl
B_2_pin_2_: bis­(pinacolato)­diboron
BET: bromodomain and extraterminal motif
Boc: *tert*-butyloxycarbonyl
bRo5: beyond-rule-of-5
Caco-2: Cancer coli 2 cells
CHI-IAM: chromatographic hydrophobicity index – immobilized artificial membrane
cLogP: calculated logP
COMU: 1-[1-(Cyano-2-ethoxy-2-oxoethylideneaminooxy)-dimethylamino-morpholino]-uronium hexafluorophosphate
COSY: correlation spectroscopy
Da: Dalton
DCM: dichloromethane
DIPEA: diisopropylethylamine
DMSO: dimethyl sulfoxide
DNA: DNA
Fmoc: 9-fluorenylmethoxycarbonyl
Fmoc-OSu: *N*-(9-Fluorenylmethoxycarbonyloxy)­succinimid
HATU: hexafluorophosphate azabenzotriazole tetramethyl uronium
HBD: hydrogen bond donor
HDAC: histone deacetylase
HSP: heat shock protein
HSQC: heteronuclear single quantum correlation
IAM: immobilized artificial membrane
IMHB: intramolecular hydrogen bond
Ile: isoleucine
K: kelvin(s) (absolute temperature)
LCH: lysosomotropism/cholestrerol homeostasis regulation
LDA: lithium diisopropylamide
*m*-CPBA: meta-chloroperoxybenzoic acid
mTOR: mammalian target of rapamycin
MW: microwave
NMR: nuclear magnetic resonance
Phe: phenylalanine
PI3K: phosphoinositide 3-kinase
Pro: proline
PSA: polar surface area
PyBOP: benzotriazol-1-yloxytripyrrolidinophosphonium hexafluorophosphate
rt: room temperature
RNA: ribonucleic acid
ROESY: rotating frame overhauser enhancement spectroscopy
SPPS: solid phase peptide synthesis
T: absolute temperature in units of kelvins (K)
TFA: trifluoroacetic acid
TOCSY: total correlation spectroscopy
tPSA: topological polar surface area
