# Supplementary material for: Backbone Alterations in Cyclic Peptides Influence Both Membrane Permeability and Biological Activity
Source: J Med Chem. 2025 Nov 6;68(22):24108–26. doi: 10.1021/acs.jmedchem.5c01901 (PMC12670408; doi:10.1021/acs.jmedchem.5c01901)

Supplemental information for “Backbone Alterations in Cyclic Peptides Influence Both Membrane Permeability and Biological Activity”

Joseph Openy,[a,b] Sabela Vega-Ces,[a] Gulshan Amrahova,[a] Emeline Mestdach,[c] Celestine Chi,[c] Benjamin Kissel,[b] and Peter ‘t Hart[a,d]*

[a]Max Planck Institute of Molecular Physiology, Otto-Hahn-Strasse 11, 44227, Dortmund, Germany

[b]Fakultät für Chemie und Chemische Biologie, Technische Universität Dortmund, Otto-Hahn-Strasse 6, 44227, Dortmund, Germany

[c]Biophysics, Discovery Sciences, AstraZeneca, SE-431 83, Gothenburg, Sweden

[d]Institute of Biochemistry, University of Münster, Correnssstrasse 36, 48149, Münster, Germany

email: pthart@uni-muenster.de

Contents

1. Synthetic Schemes 3
2. Supplemental tables 5
3. Supplemental Figures 7
   tPSA correlation 7
   VT-NMR spectra 7
   H-D exchange spectra 11
4. NMR structure data 14
5. Cell Painting 30
6. IAM chromatograms 31
7. Compound characterization 39
   HPLC chromatograms peptides 39
   NMR spectra small molecules 43

1. Synthetic Schemes

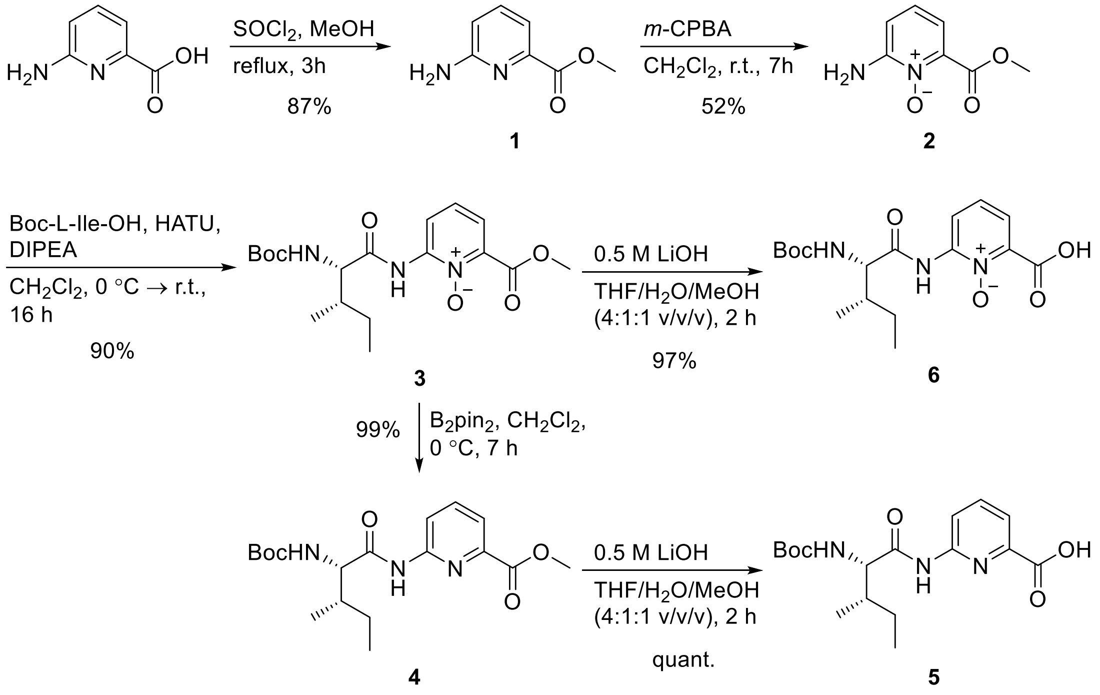

Scheme S1. Synthesis of dipeptides **5** and **6** for SA-B1 and SA-B2.

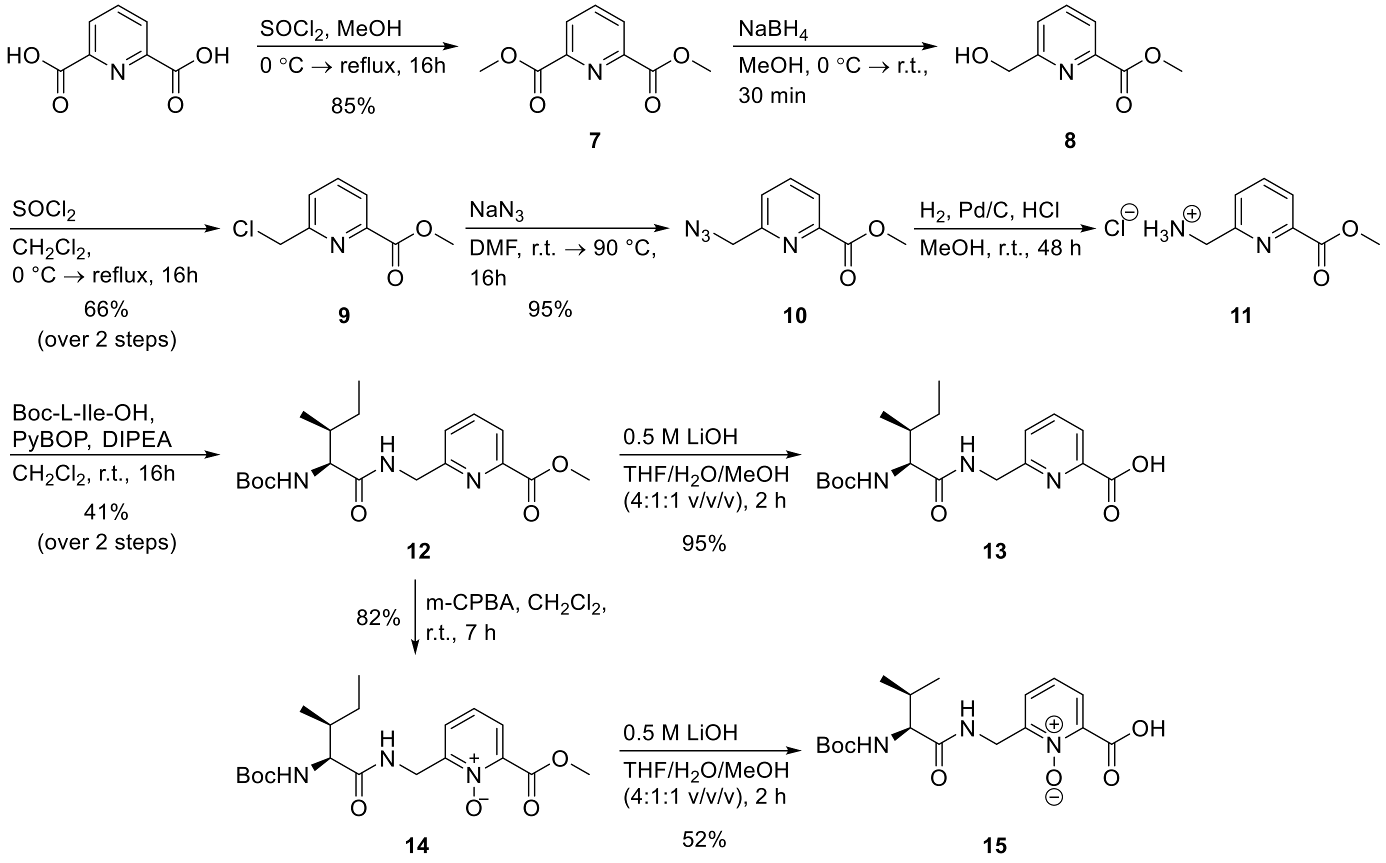

Scheme S2. Synthesis of dipeptides **13** and **15** for SA-C1 and SA-C2.

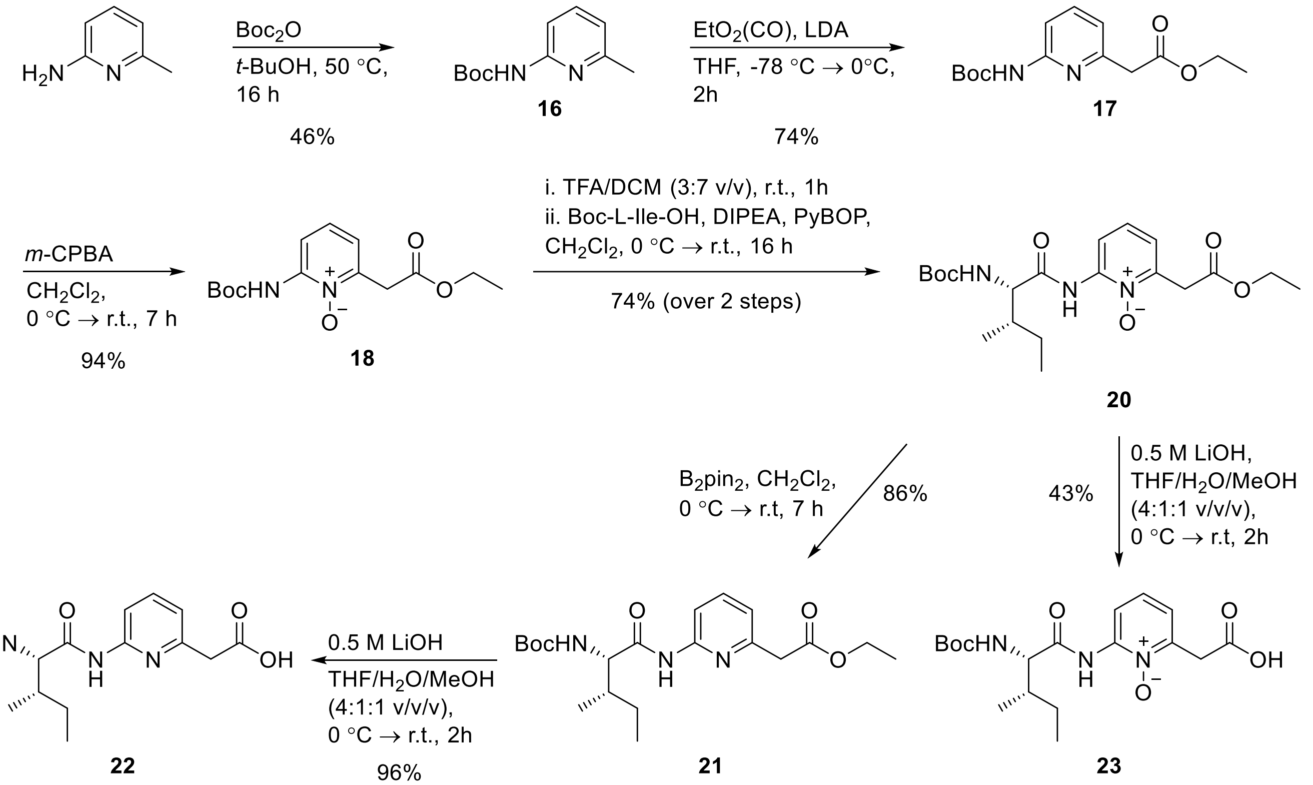

Scheme S3. Synthesis of dipeptides **22** and **23** for SA-D1 and 2.

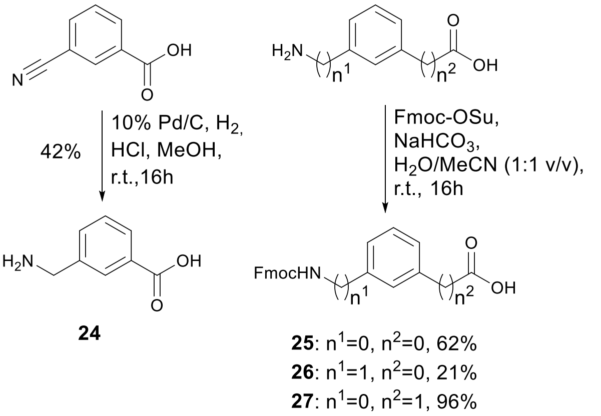

Scheme S4. Synthesis of benzene backbone building blocks for peptides SA-B3, -C3, and -D3.

2. Supplemental tables

Table S1: Peptides yields and HRMS results

Peptide	Yield (%)	Exact mass calc. $[M+H]^+$	Exact mass meas. $[M+H]^+$
SA-A1	86	722.3694	722.3691
SA-A2	28	764.4164	764.4161
SA-A3	42	742.3956	742.3956
SA-B1	9	759.4188	759.4201
SA-B2	9	775.4137	775.4136
SA-B3	9	758.4236	758.4235
SA-C1	8	773.4345	773.4345
SA-C2	17	789.4294	789.4294
SA-C3	17	773.4345	773.4344
SA-D1	11	773.4345	773.4345
SA-D2	3	789.4294	789.4293
SA-D3	16	772.4392	772.4391

Table S2: Structural statistics of the 20 NMR structures of SA-A1, SA-B1 and SA-B3 in both solvents.

Experimental restraints	SA-A1<br>$CDCl_3$	SA-A1<br>DMSO	SA-B1<br>$CDCl_3$	SA-B1<br>DMSO	SA-B3<br>$CDCl_3$	SA-B3<br>DMSO
Upper distance limits: total	64	60	53	51	62	49
short-range, |i-j|<=1	46	46	43	40	46	36
medium-range, 1<|i-j|<5	14	8	5	6	10	10
long-range, |i-j|>=5	4	6	5	5	6	3
Average target function value	0.89	1.08	0.79	0.70	0.64	0.75
$^{3}J_{HNCH}$ (Hz) used for torsion angles restrictions	1Ala: 8.1<br>2Phe: <2.6	2Phe: <2.6<br>4Ile: 8.5	2Phe: <2.6<br>6Ile: 8.6	2Phe: 3.7<br>4Ile: 8.6<br>6Ile: 5.1	2Phe: <2.6<br>4Ile: 8.1<br>6Ile: 9.8	2Phe: <3.2<br>6Ile: 5.3

3. Supplemental Figures

tPSA correlation

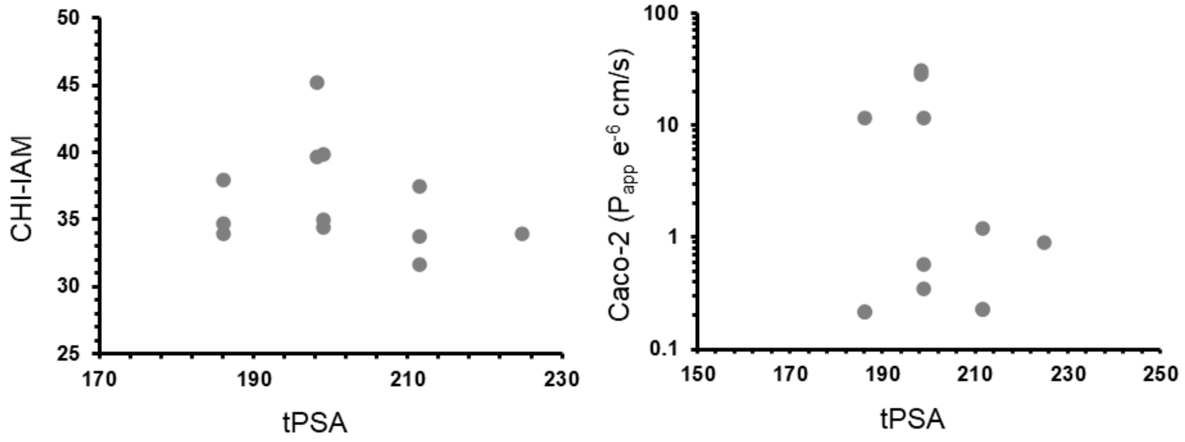

Figure S1: Correlation plot of tPSA and CHI-IAM values (left) and tPSA and Caco-2 values (right).

VT-NMR spectra

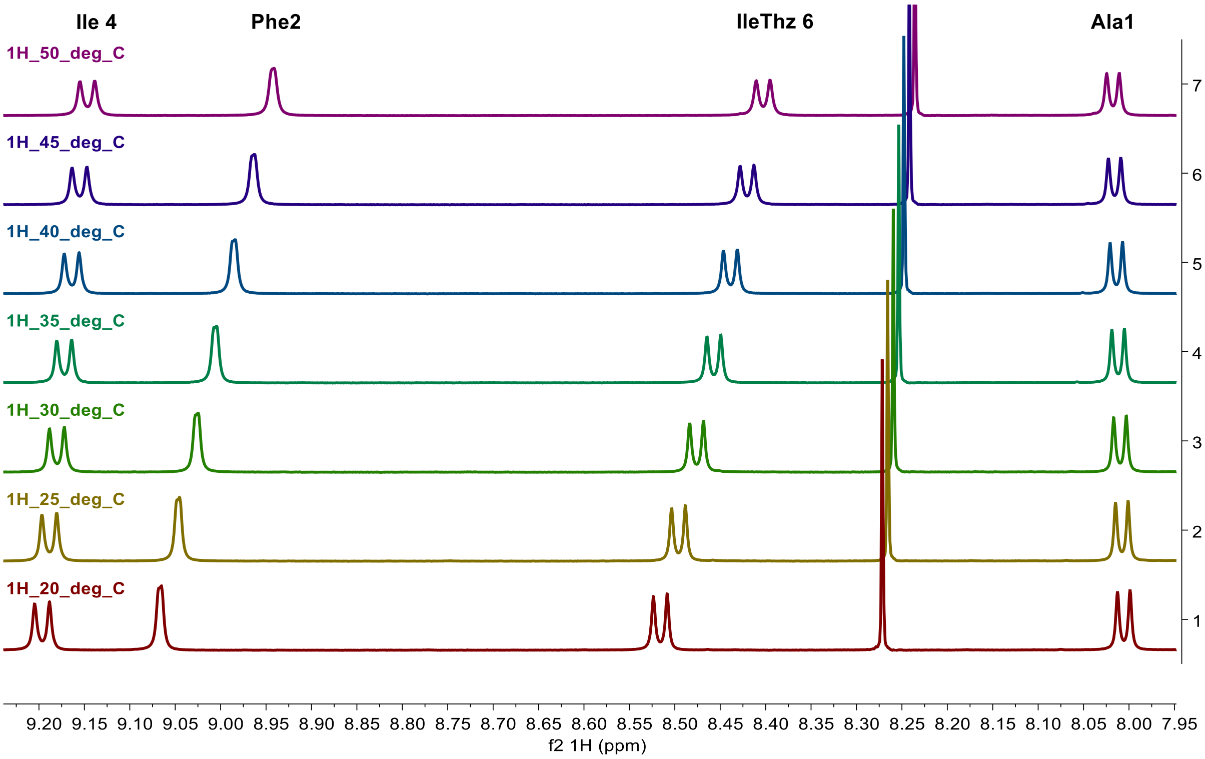

Figure S2: Amide proton shifts for peptide SA-A1 ranging from 20°C - 50°C (bottom to top) in 5°C steps measured in DMSO-d_6.

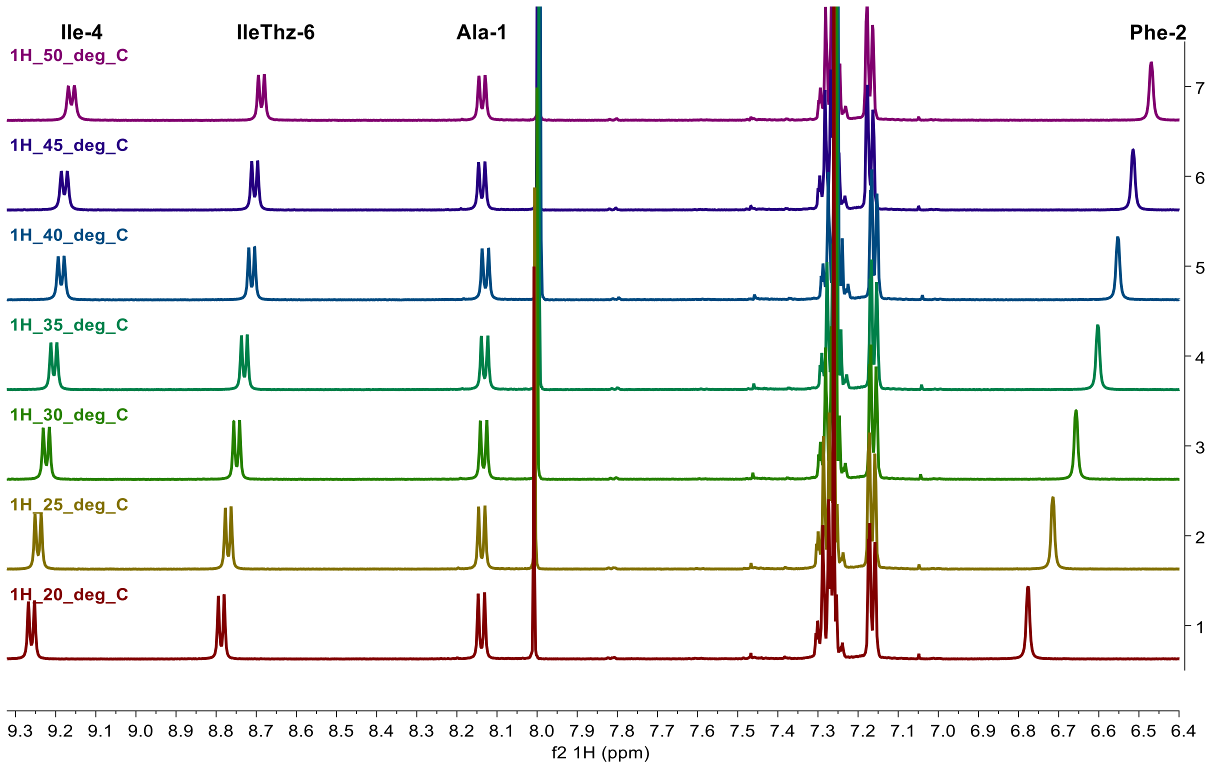

Figure S3: Amide proton shifts for peptide SA-A1 ranging from 20°C - 50°C (bottom to top) in 5°C steps measured in $CDCl_3$.

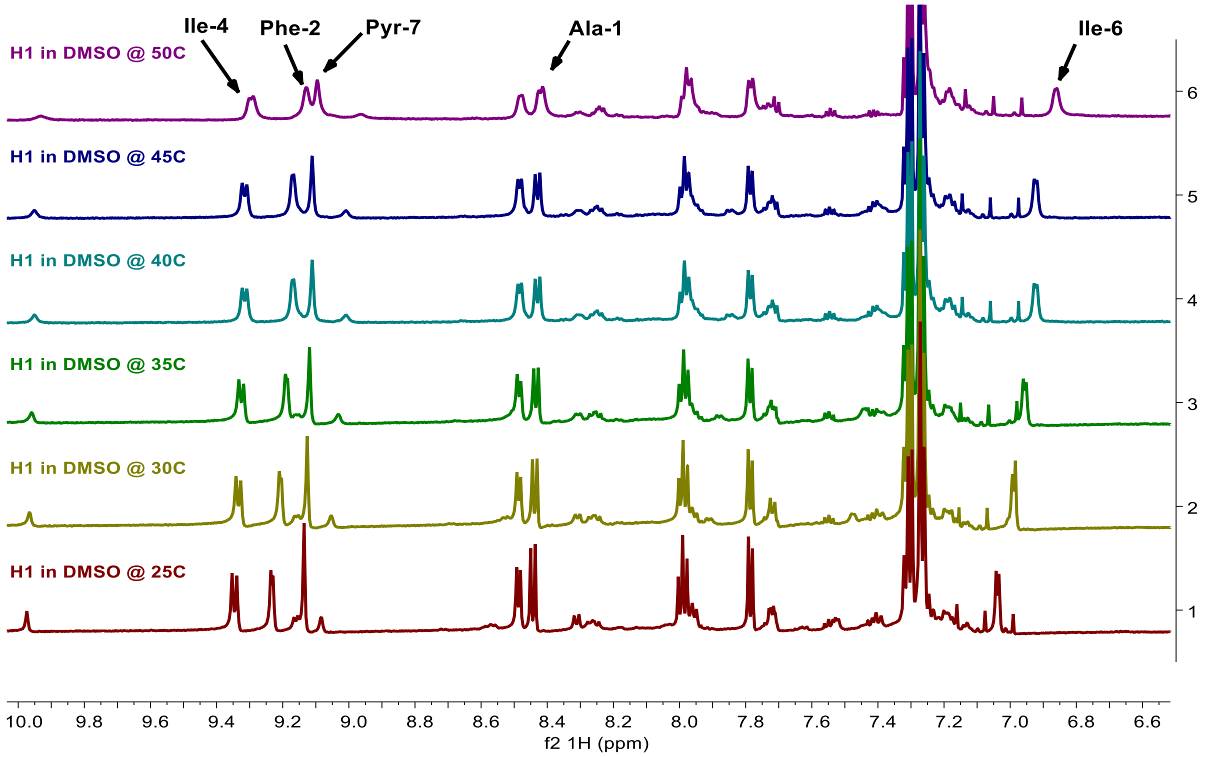

Figure S4: Amide proton shifts for peptide SA-B1 ranging from 20°C - 50°C (bottom to top) in 5°C steps measured in DMSO-d_6.

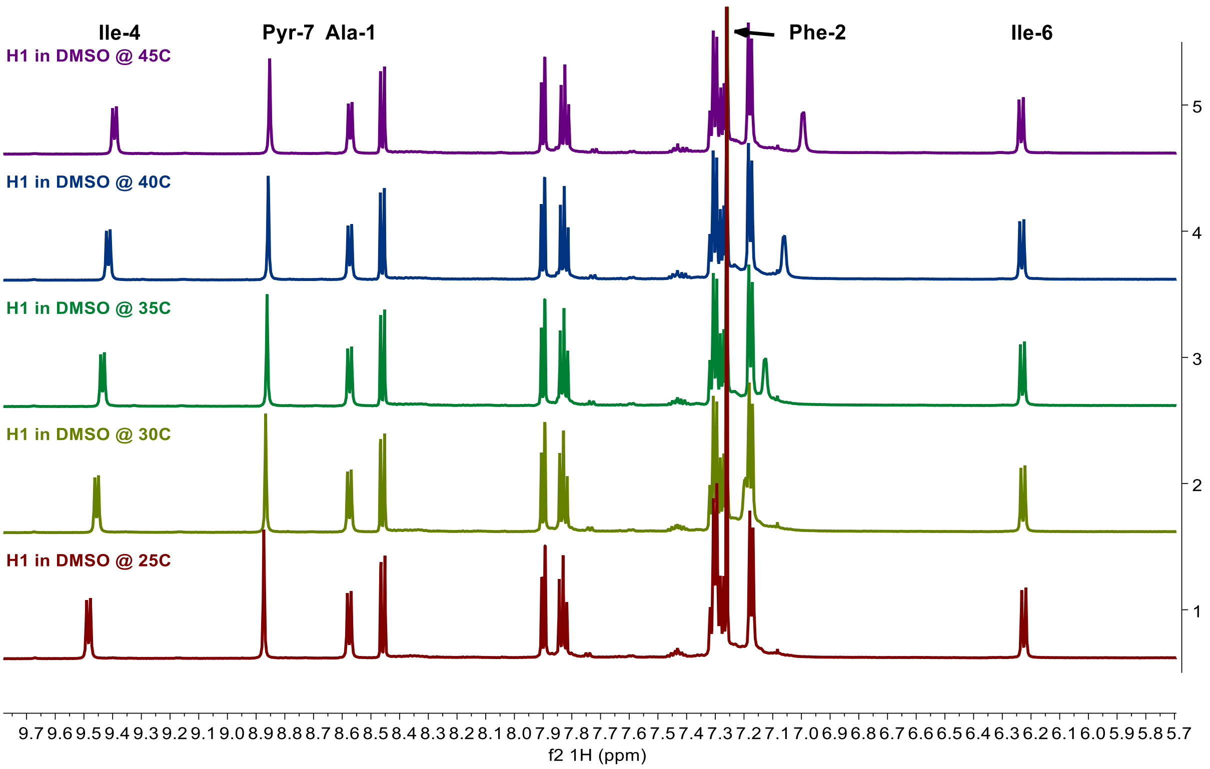

Figure S5: Amide proton shifts for peptide SA-B1 ranging from 20°C - 45°C (bottom to top) in 5°C steps measured in $CDCl_3$.

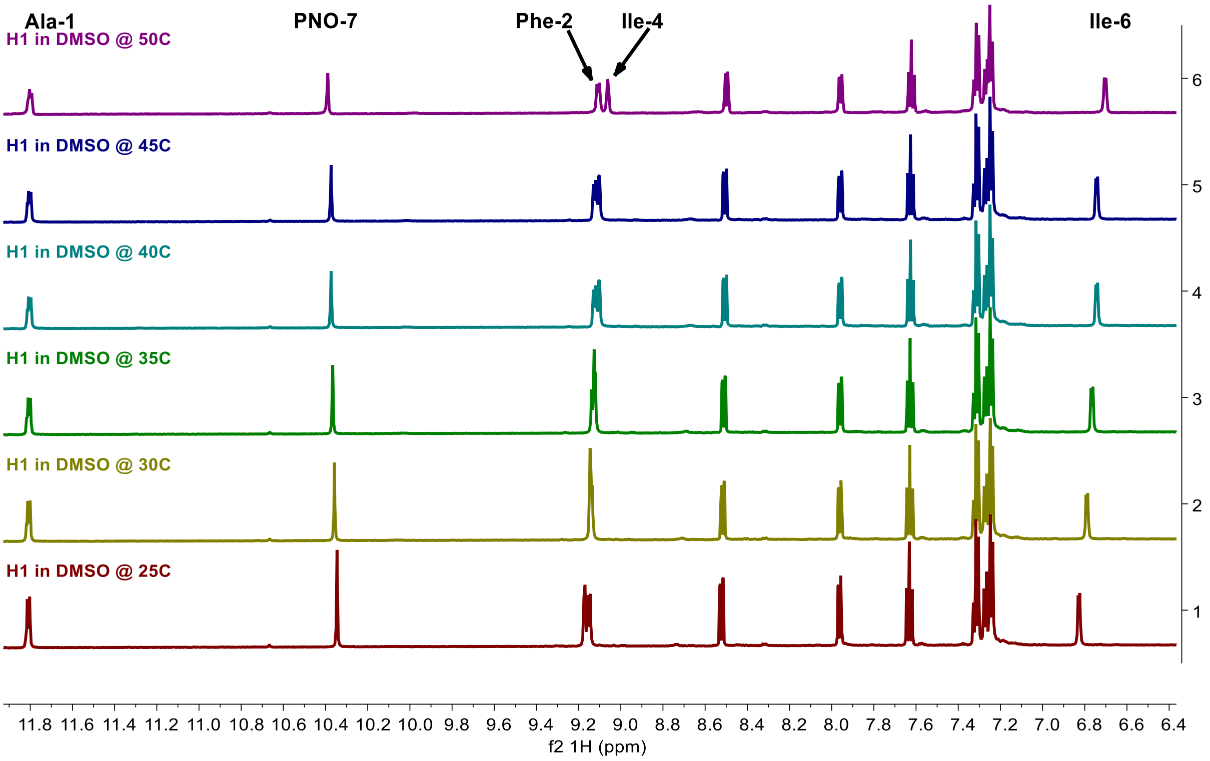

Figure S6: Amide proton shifts for peptide SA-B2 ranging from 20°C - 50°C (bottom to top) in 5°C steps measured in DMSO-d_6.

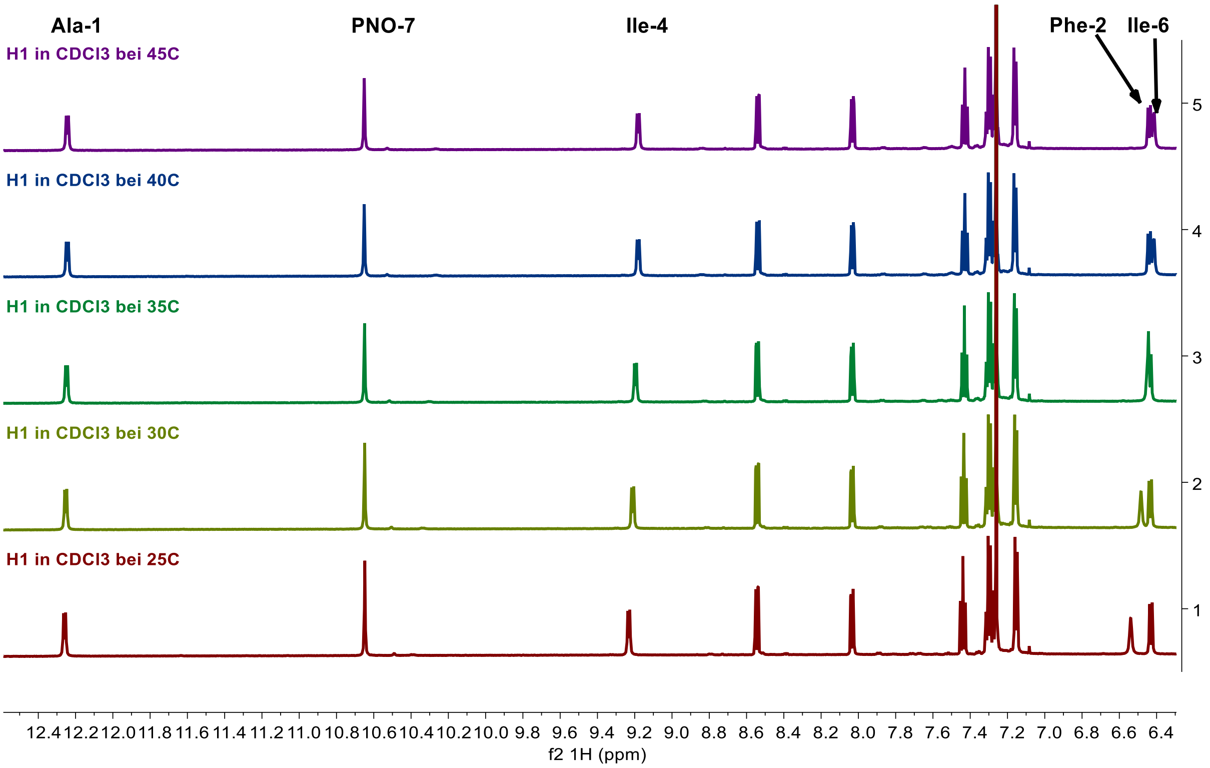

Figure S7: Amide proton shifts for peptide SA-B2 ranging from 20°C - 45°C (bottom to top) in 5°C steps measured in $CDCl_3$.

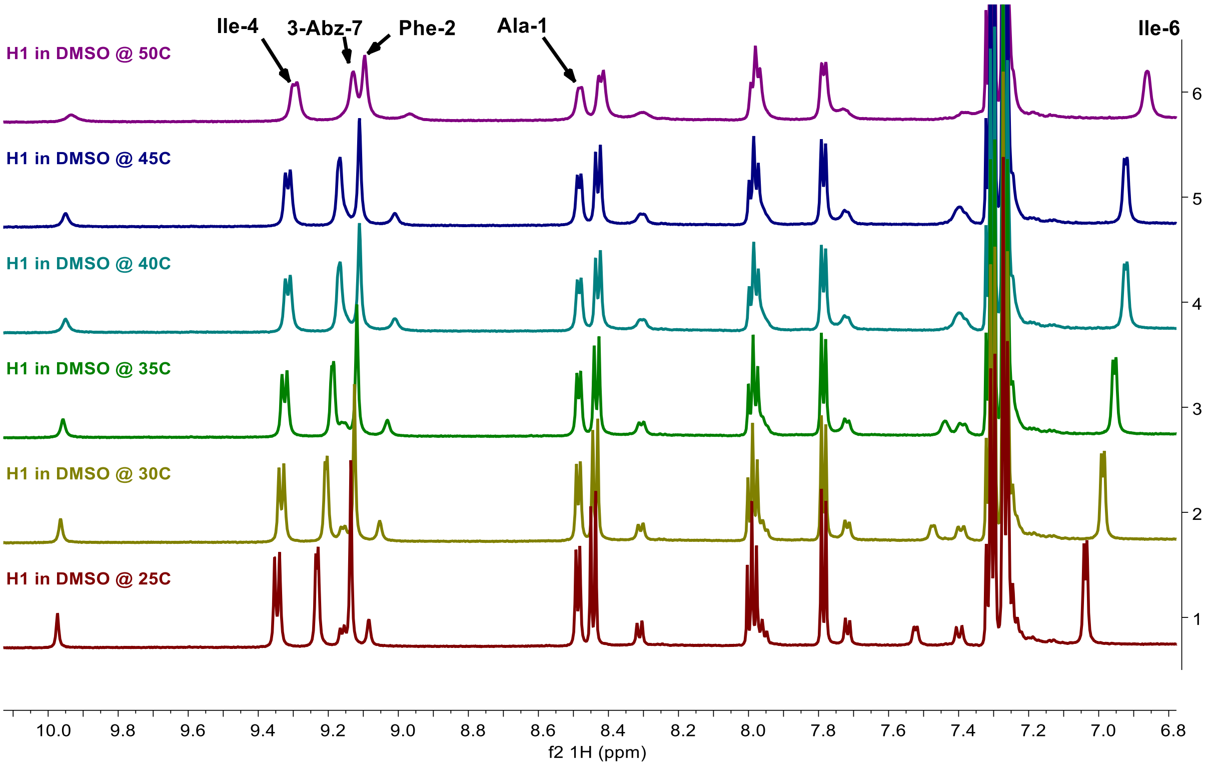

Figure S8: Amide proton shifts for peptide SA-B3 ranging from 20°C - 50°C (bottom to top) in 5°C steps measured in DMSO-d_6.

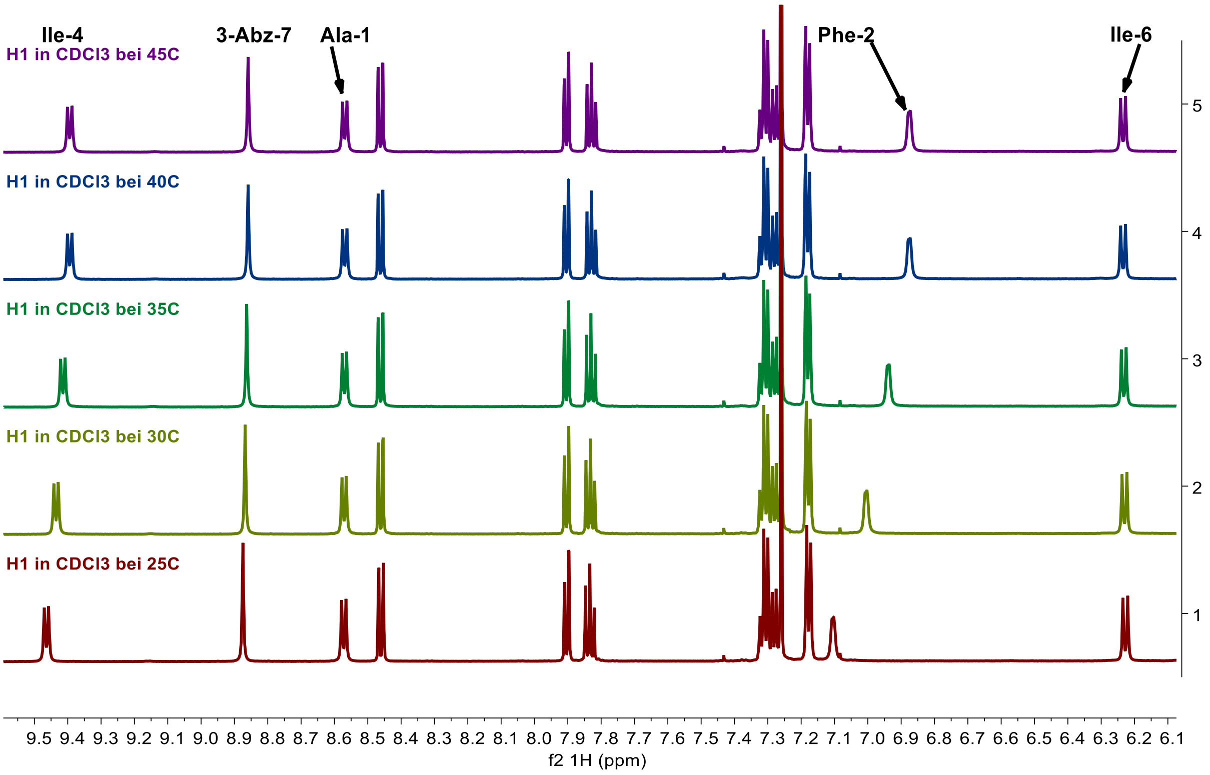

Figure S9: Amide proton shifts for peptide SA-B3 ranging from 20°C - 45°C (bottom to top) in 5°C steps measured in $CDCl_3$.

H-D exchange spectra

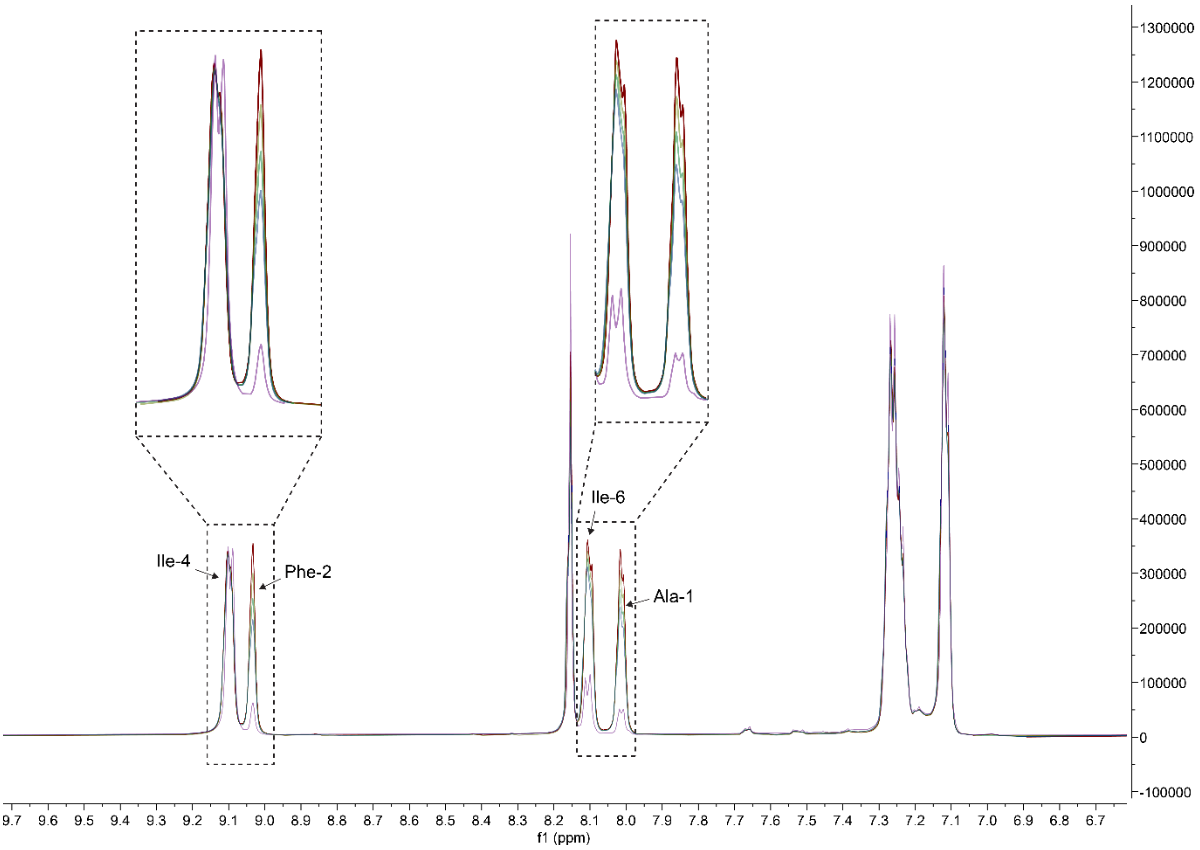

Figure S10: Overlay of amide proton H-D exchange spectra for peptide SA-A1 in DMSO-d_6/D_2O (80%/20%). Spectra were recorded at 0, 10, 20, 30, and 240 minutes.

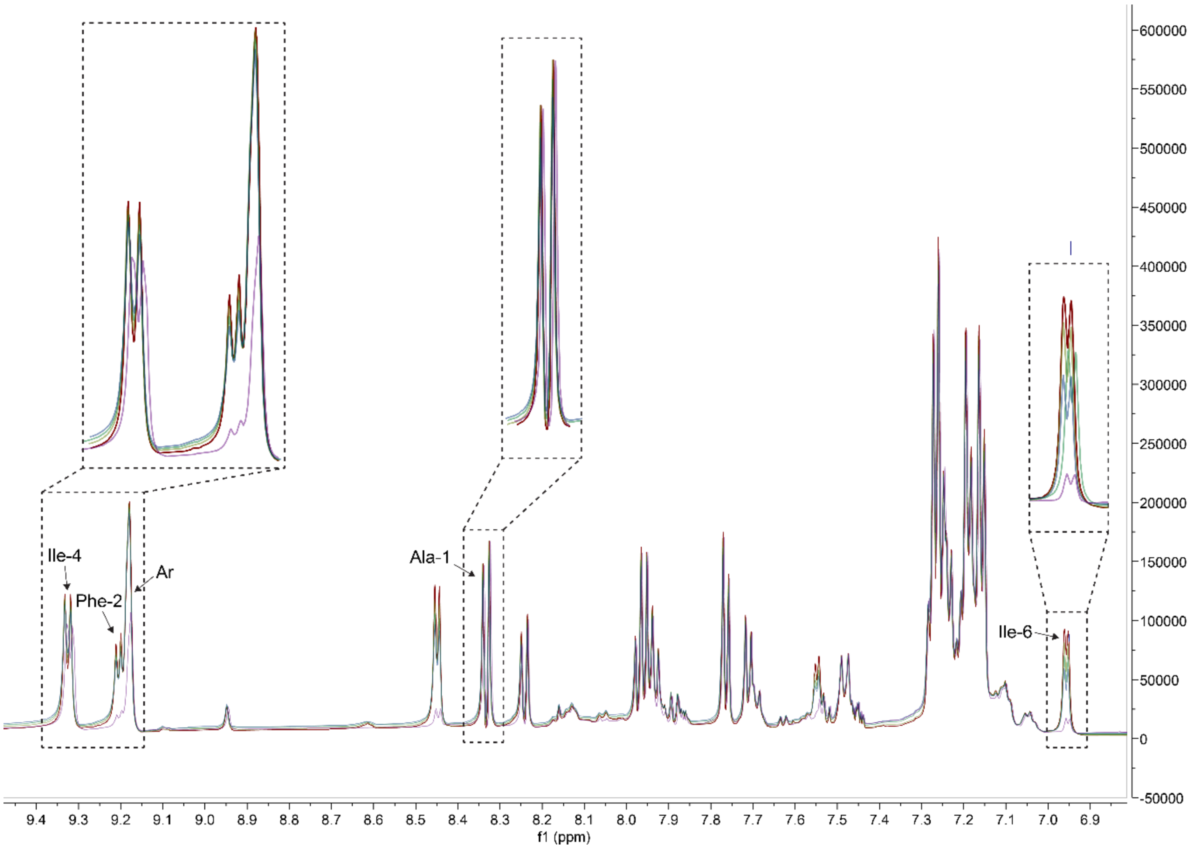

Figure S11: Overlay of amide proton H-D exchange spectra for peptide SA-B1 in DMSO-d_6/D_2O (80%/20%). Spectra were recorded at 0, 10, 20, 30, and 240 minutes.

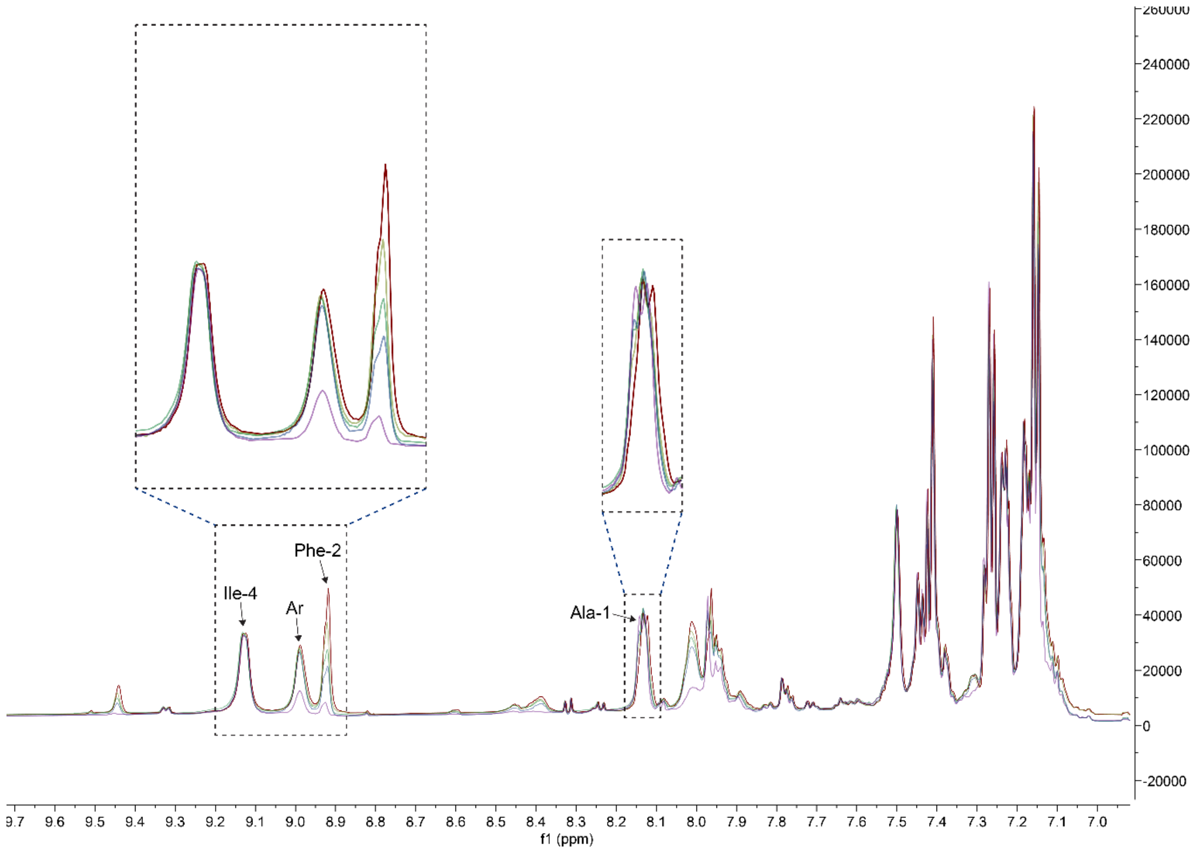

Figure S12: Overlay of amide proton H-D exchange spectra for peptide SA-B3 in DMSO-d_6/D_2O (80%/20%). Spectra were recorded at 0, 10, 20, 30, and 240 minutes.

4. NMR structure data

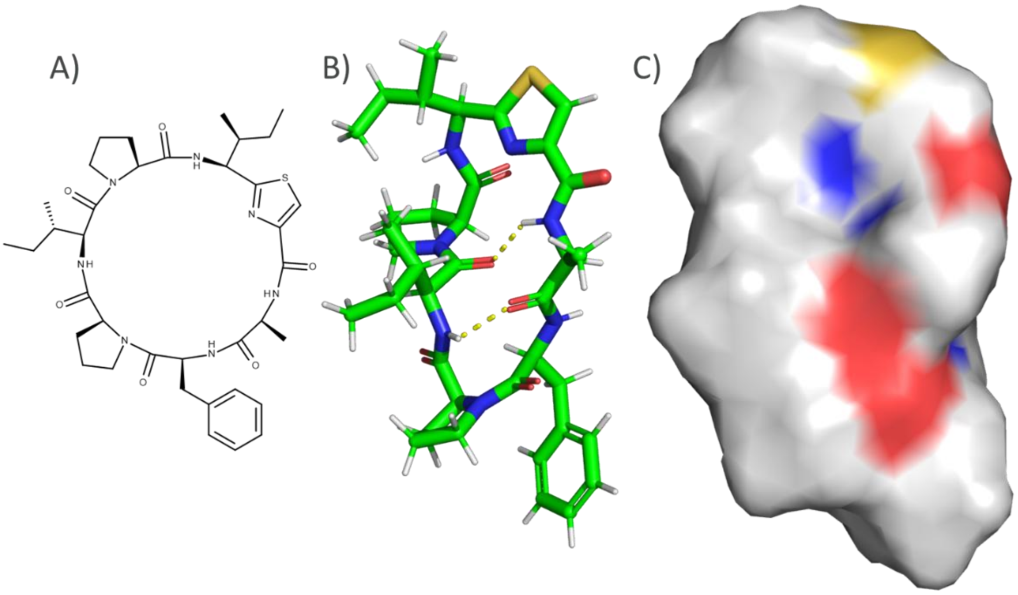

Figure S13: SA-A1 (in DMSO): A) molecular structure, B) NMR-derived solution structure, and C) solvent-exposed surface depicting polar groups (nitrogen: blue, oxygen: red) amongst hydrophobic surface (grey).

Thiazole (yellow), 1AlaNH (slightly) and 2PheNH are solvent exposed while 4IleNH and 6IleNH are shielded from the solvent. Hydrogen bonds are formed between 1AlaNH—4IleCO and 1AlaCO—4IleNH.

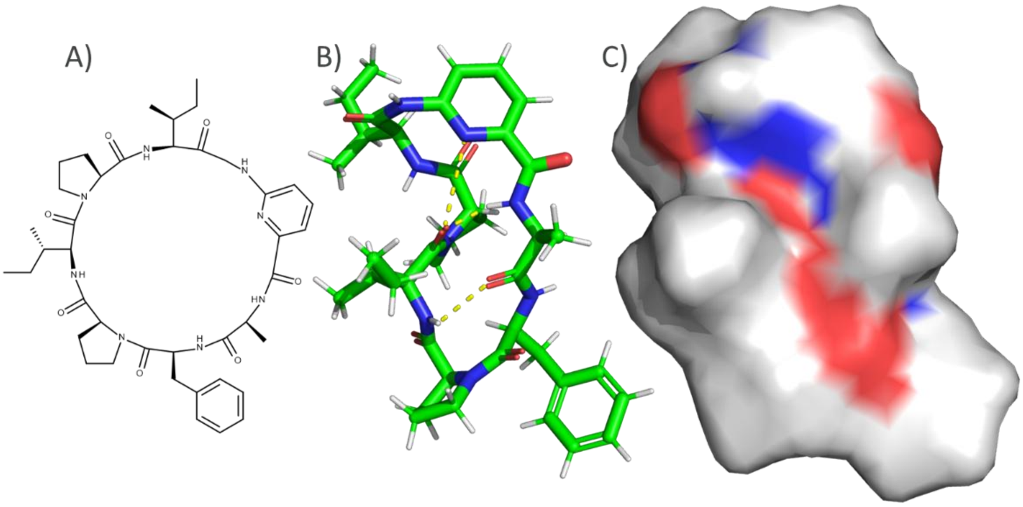

Figure S14: SA-B1 (in DMSO): A) molecular structure, B) NMR-derived solution structure, and C) solvent-exposed surface depicting polar groups (nitrogen: blue, oxygen: red) amongst hydrophobic surface (grey).

1AlaNH (slightly) and 2PheNH are still solvent exposed while 4IleNH remains shielded from the solvent. Hydrogen bonds are formed between 1AlaNH—4IleCO and 1AlaCO—4IleNH. Since 6Ile side chain is leaning towards the modified macrocycle part, 6IleNH is more accessible to the solvent.

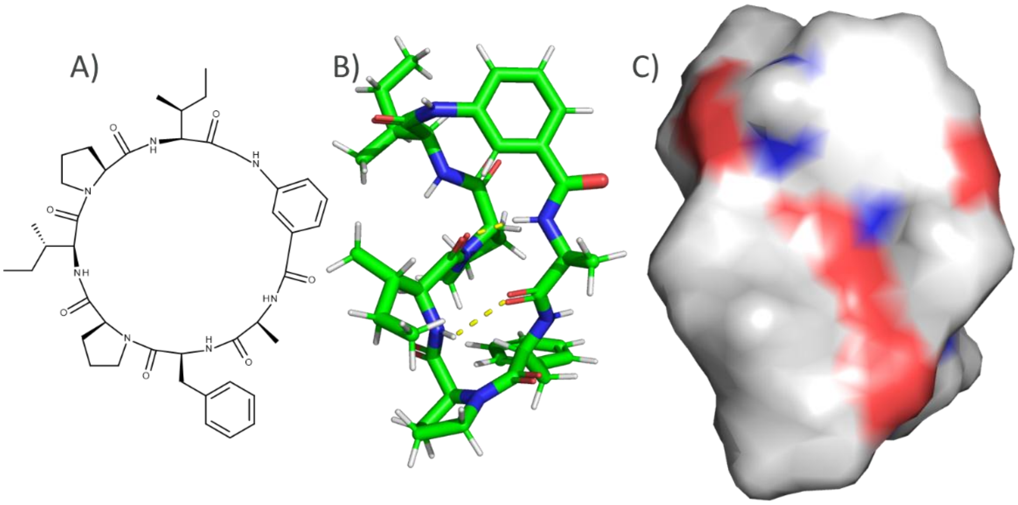

Figure S15: SA-B3 (in DMSO): A) molecular structure, B) NMR-derived solution structure, and C) solvent-exposed surface depicting polar groups (nitrogen: blue, oxygen: red) amongst hydrophobic surface (grey).

1AlaNH (slightly) and 2PheNH are still solvent exposed while 4IleNH remains shielded from the solvent. Hydrogen bonds are formed between 1AlaNH—4IleCO and 1AlaCO—4IleNH. As for SA-B1, 6IleNH is more accessible to the solvent due to Ile6 side chain facing the modified macrocycle part.

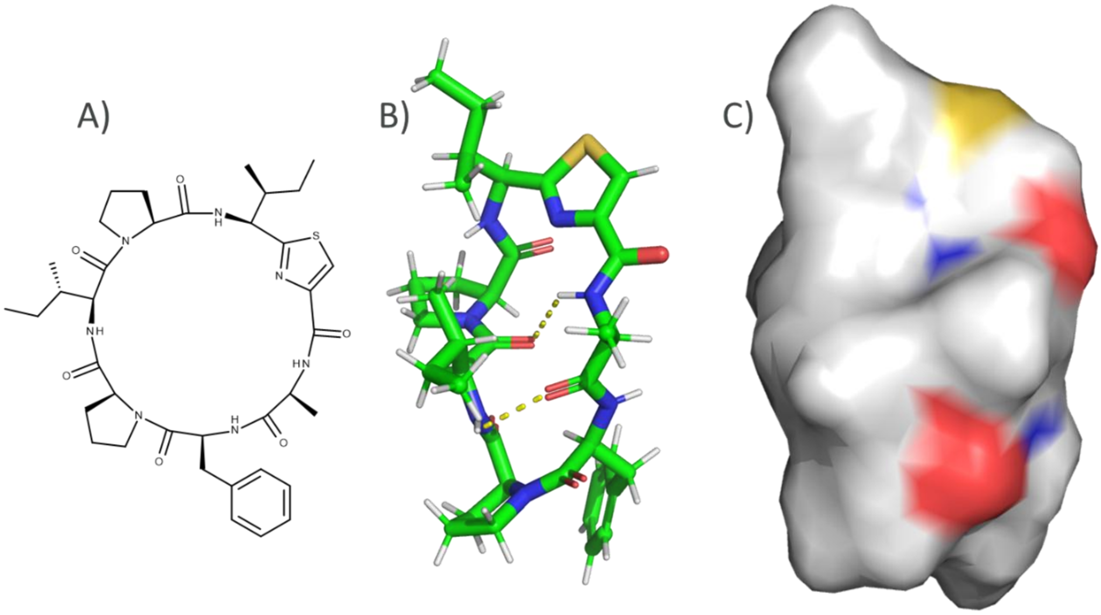

Figure S16: SA-A1 (in $CDCl_3$): A) molecular structure, B) NMR-derived solution structure, and C) solvent-exposed surface depicting polar groups (nitrogen: blue, oxygen: red) amongst hydrophobic surface (grey).

Thiazole (yellow), 1AlaNH (slightly) and 2PheNH are solvent exposed. Hydrogen bonds are formed between 1AlaNH—4IleCO and 1AlaCO—4IleNH, while 4IleNH still tends to face the solvent environment. 6IleNH is not shielded by its side chain, thus more accessible to the solvent.

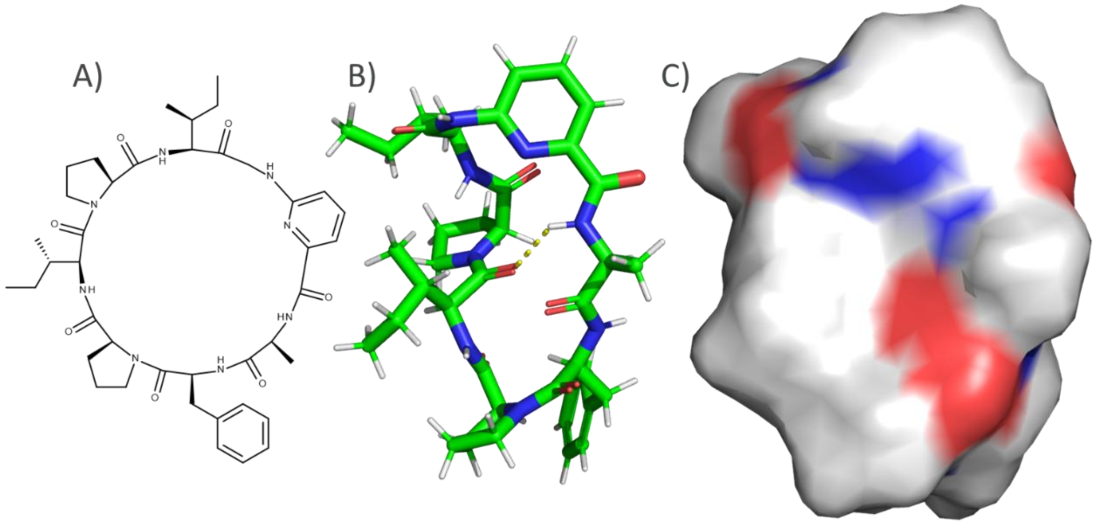

Figure S17: SA-B1 (in $CDCl_3$): A) molecular structure, B) NMR-derived solution structure, and C) solvent-exposed surface depicting polar groups (nitrogen: blue, oxygen: red) amongst hydrophobic surface (grey).

1AlaNH (slightly) and 2PheNH are still solvent exposed. Hydrogen bond is formed between 1AlaNH—4IleCO. 4IleNH is heading towards the solvent environment, thus facilitating exchange, compared to the DMSO-based structure. 6IleNH becomes more shielded from solvent due to 6Ile side chain closing in towards 5Pro.

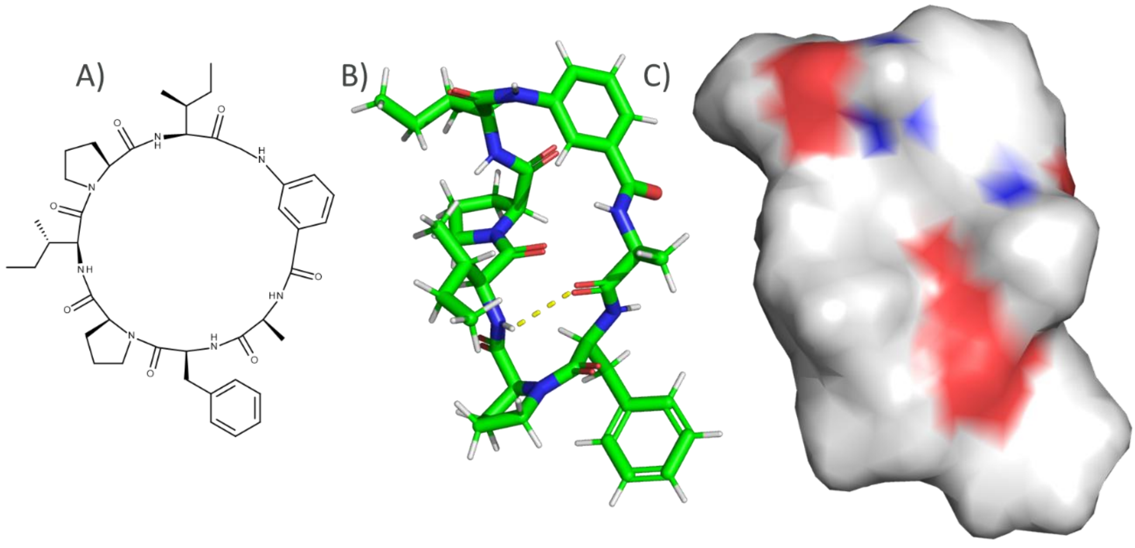

Figure S18: SA-B3 (in $CDCl_3$): A) molecular structure, B) NMR-derived solution structure, and C) solvent-exposed surface depicting polar groups (nitrogen: blue, oxygen: red) amongst hydrophobic surface (grey).

2PheNH is still solvent exposed, and 1AlaNH is not involved in hydrogen bond anymore, facilitating the solvent access greatly. Intramolecular hydrogen bond is formed between 1AlaCO—4IleNH. Moreover, 4IleNH is further heading towards the macrocycle, and covered by 4Ile side chain, which shields the amide from solvent. 6IleNH is more shielded from solvent due to 6Ile side chain closing in with Pro5, as in SA-B1.

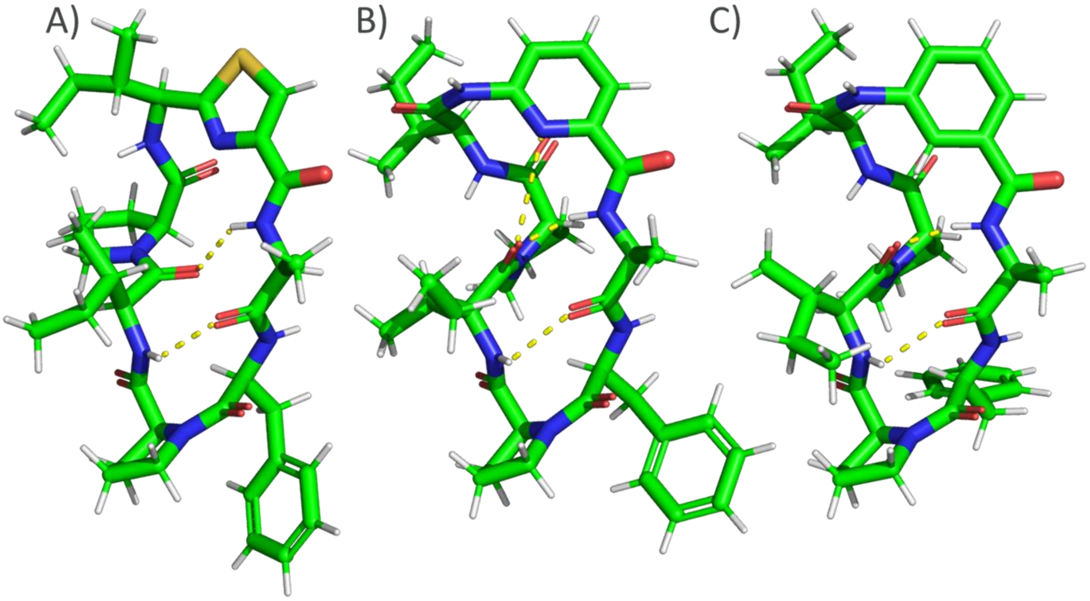

Figure S19: In DMSO: A) SA-A1, B) SA-B1 and C) SA-B3.

1AlaNH/CO and 4IleCO/NH remains in similar hydrogen bonds throughout the various peptides, as well as exposure of 2PheNH. 6IleNH is shielded by the closeness of 6Ile side chain and 5Pro in SA-A1, and becomes less protected when adding an amide group and a benzene-type ring to the macrocycle.

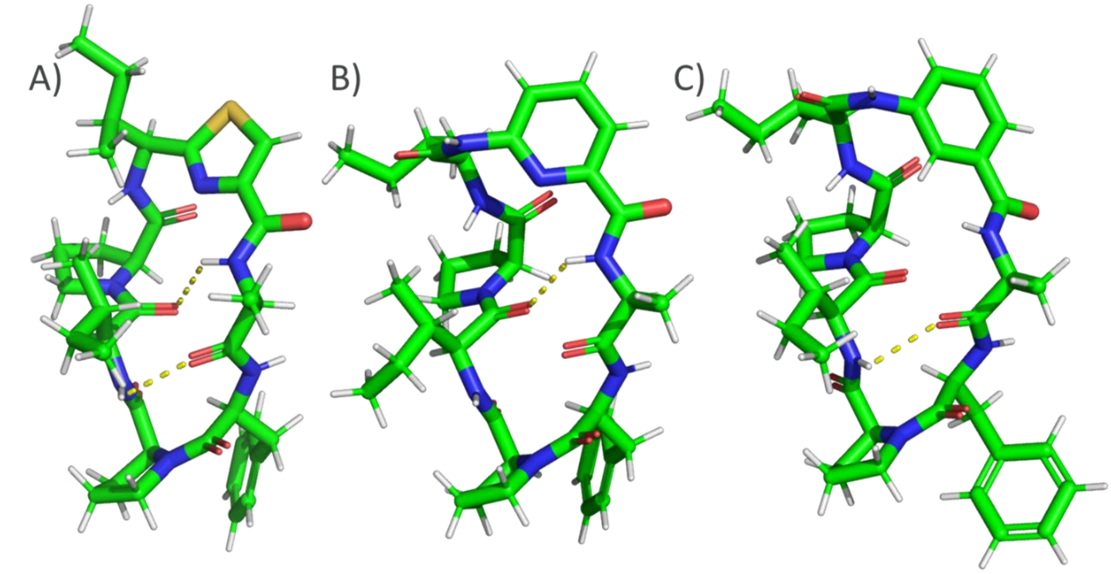

Figure S20: In $CDCl_3$: A) SA-A1, B) SA-B1 and C) SA-B3.

In all structures, 2PheNH remains exposed to the solvent. While involved in 1AlaNH--4IleCO hydrogen bond in SA-A1 and SA-B1, 1AlaNH faces the solvent in SA-B3. In SA-B3, 4IleNH is heading towards 2AlaCO to form an intramolecular hydrogen bond while being shielded by 4Ile side chain, thus being less accessible to solvent. 6IleNH is shielded by the closeness of 6Ile side chain and 5Pro in SA-B1 and SA-B3, while it is more exposed in the initial sanguinamide SA-A1.

Table S3: Dihedral angle analysis of 2Phe and 3Pro

Measurement	Φ 2Phe (°)	Ψ 2Phe (°)	Φ 3Pro (°)	Ψ 3Pro (°)
SA-A1 $CDCl_3$	-37	140.5	-69.8	-25.7
SA-A1 DMSO	-39	150.5	-69.8	-0.8
SA-A1 $CDCl_3$	-39.4	148.5	-69.8	-33.3
SA-A1 DMSO	-43	151.1	-69.8	-9.1
SA-A1 $CDCl_3$	-47.1	157.9	-69.8	-8
SA-A1 DMSO	-47	151.7	-69.8	-6.5

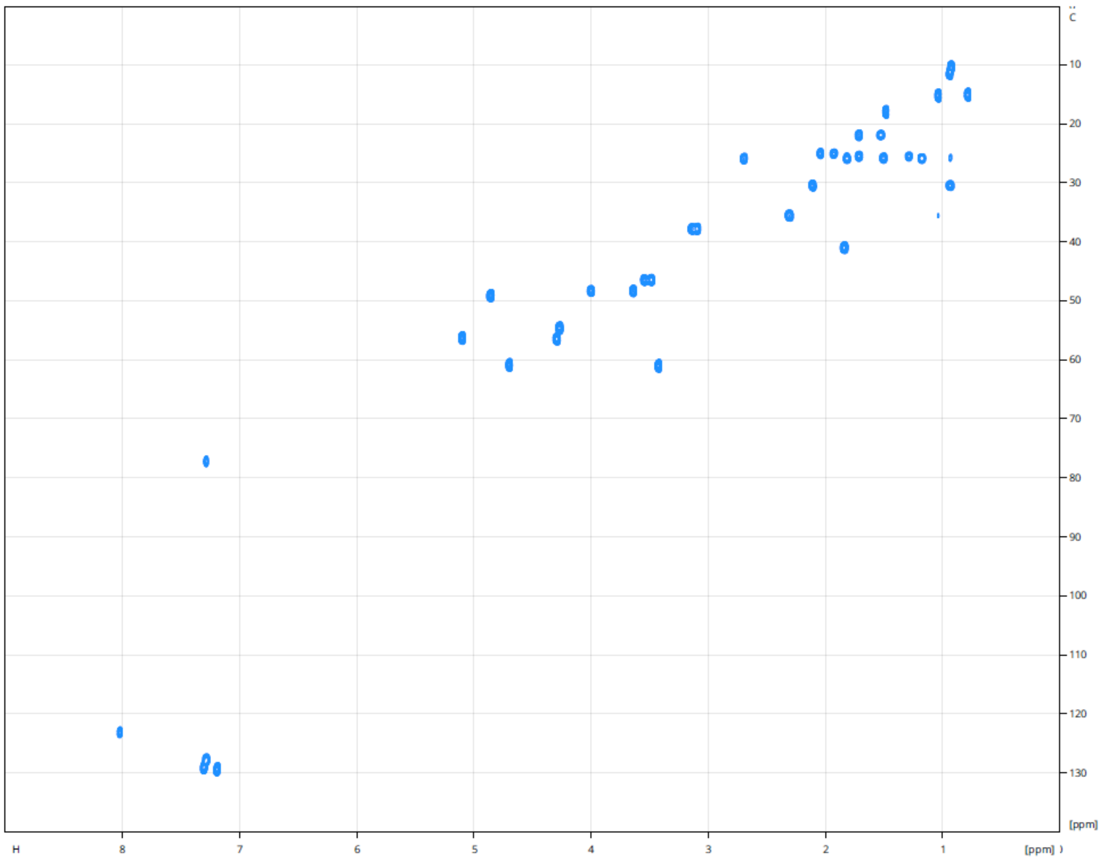

Figure S21: 600MHz ^{1}H,^{13}C-HSQC spectrum of sanguinamide A (SA-A1) in $CDCl_3$.

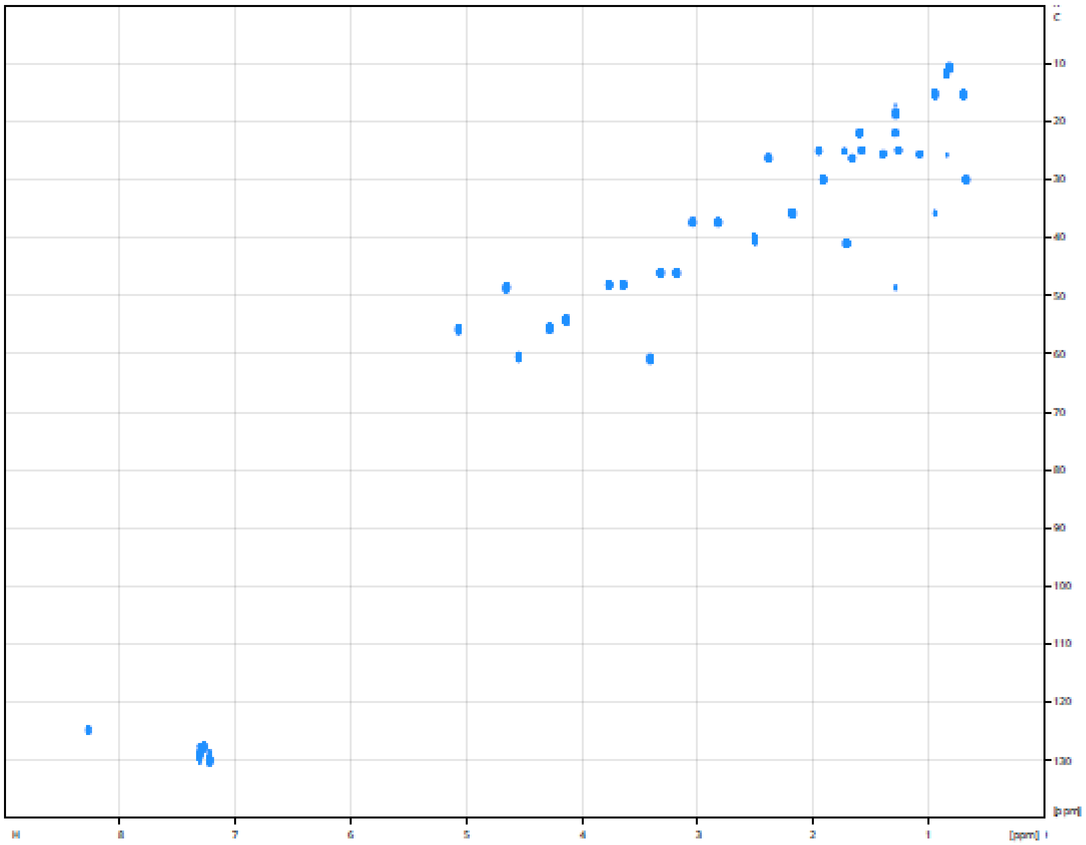

Figure S22: 600MHz $^{1}H,^{13}C$-HSQC spectrum of sanguinamide SA-A1 in DMSO.

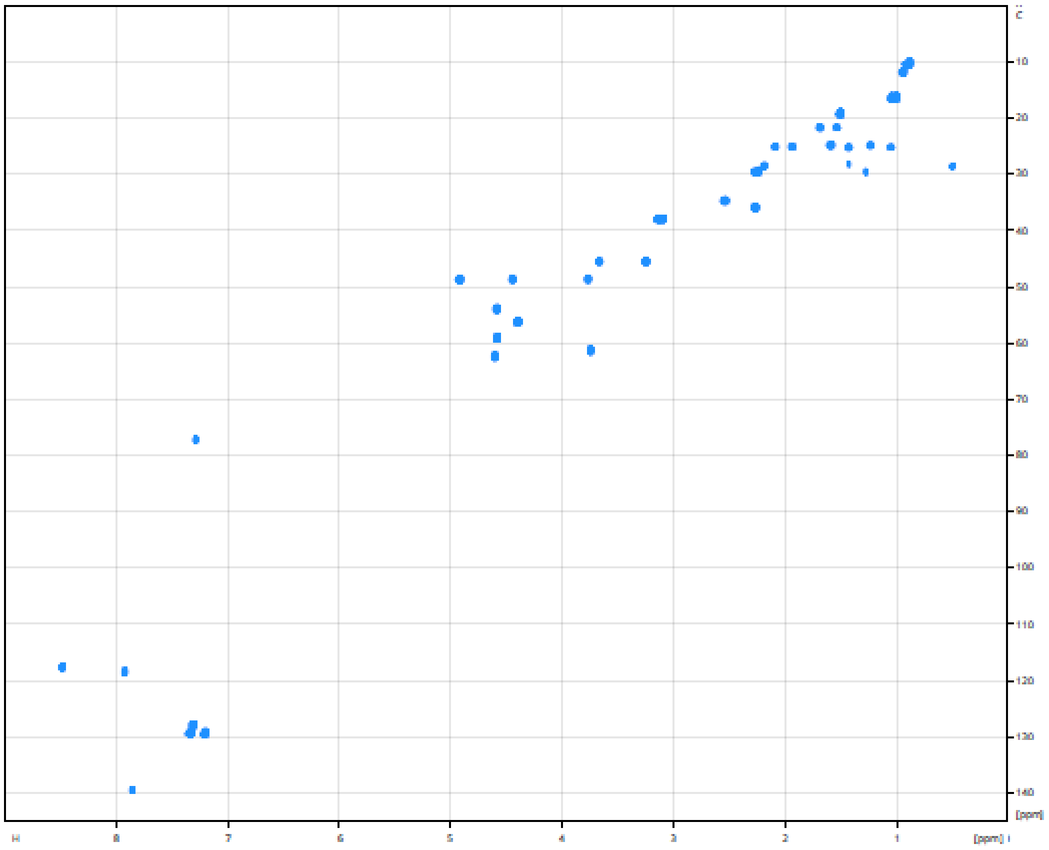

Figure S23: 600MHz ^{1}H,^{13}C-HSQC spectrum of SA-B1 in $CDCl_3$.

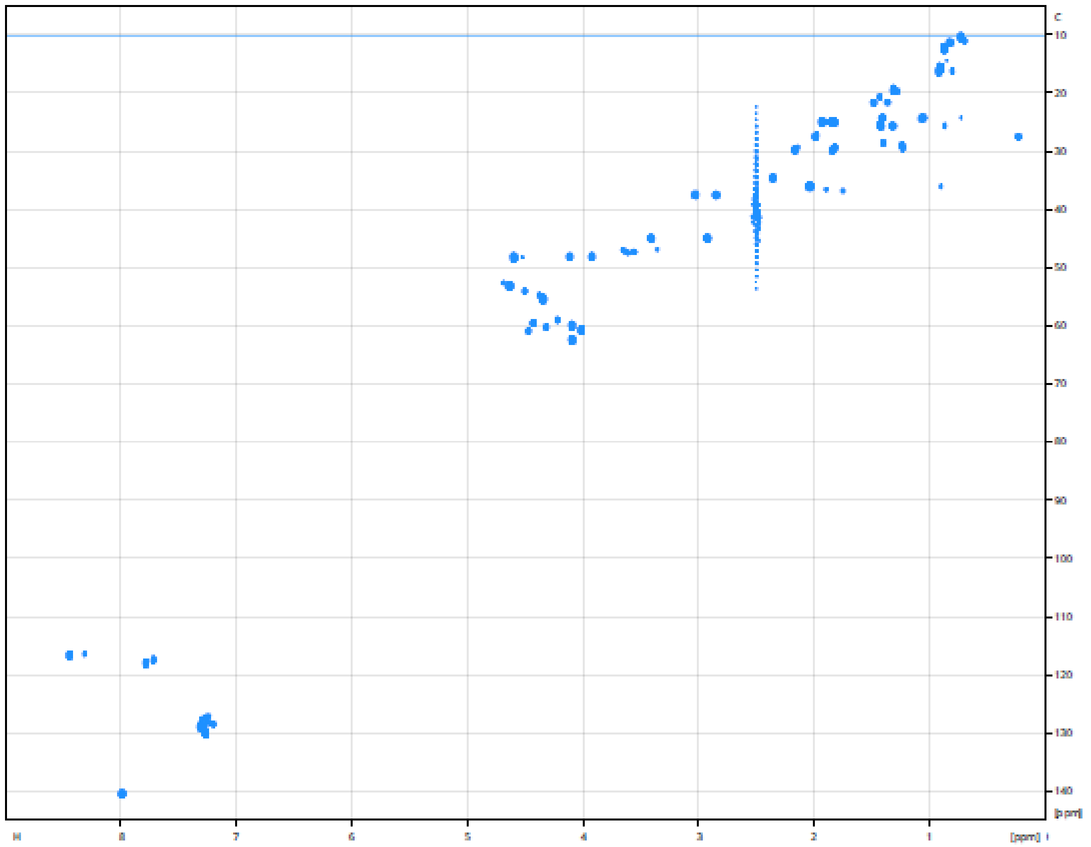

Figure S24: 600MHz $^{1}H,^{13}C$-HSQC spectrum of SA-B1 in DMSO.

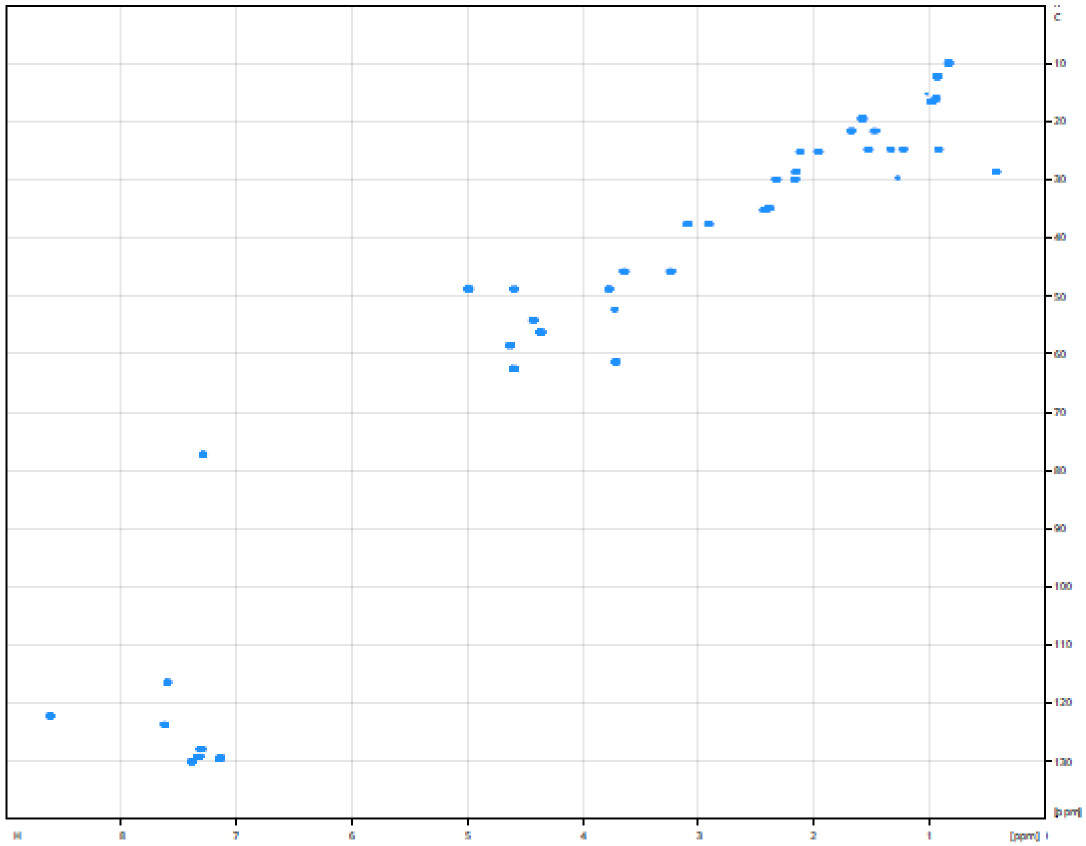

Figure S25: 600MHz $^{1}H,^{13}C$-HSQC spectrum of SA-B3 in $CDCl_3$.

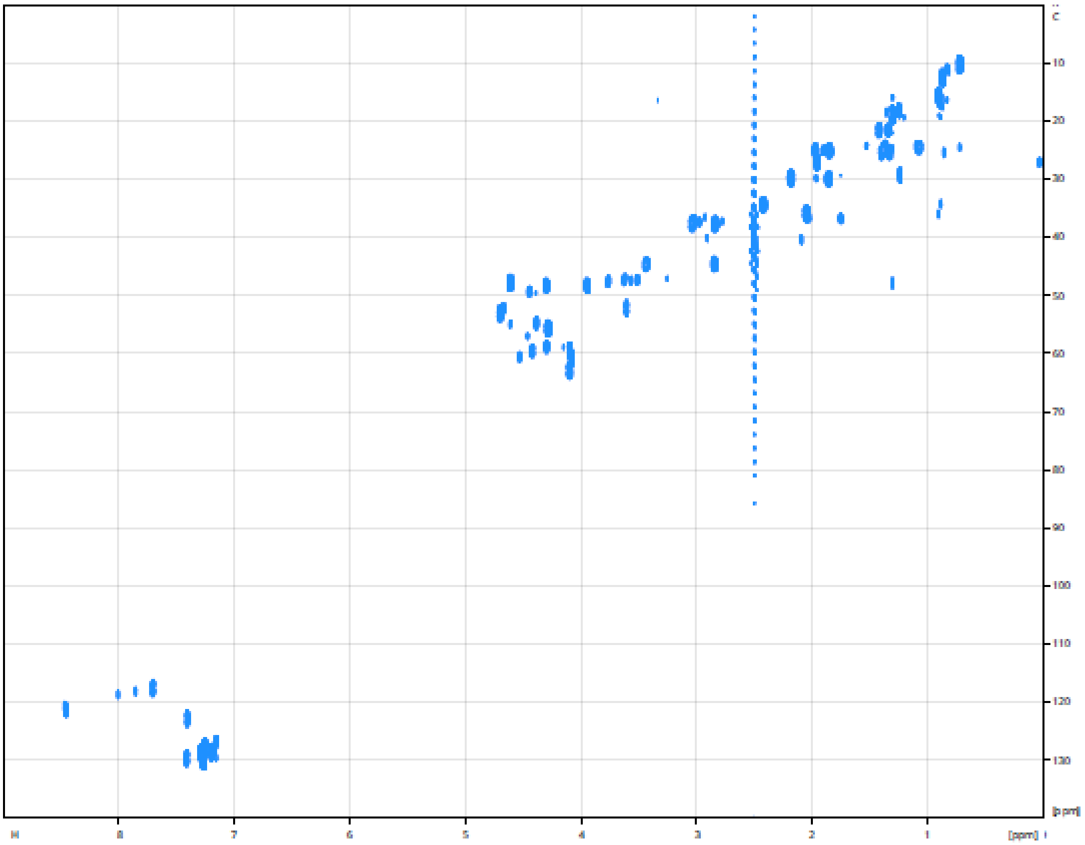

Figure S26: 600MHz ^{1}H,^{13}C-HSQC spectrum of SA-B3 in DMSO.

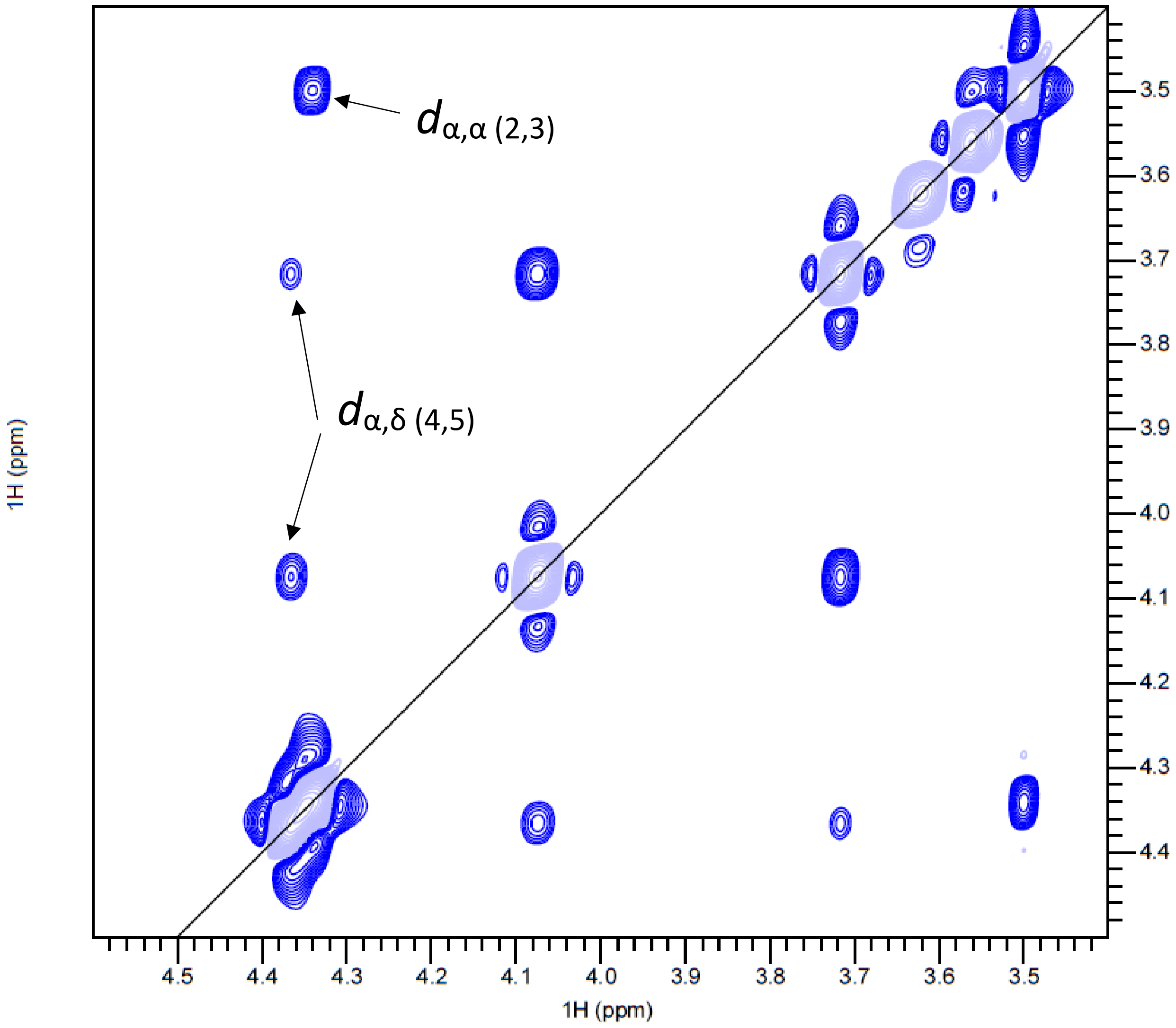

Figure S27: Selected region of 600MHz ROESY spectrum of SA-A1 in $CDCl_3$ at 298K showing ROE between alpha/delta protons indicating conformation for 3Pro and 5Pro.

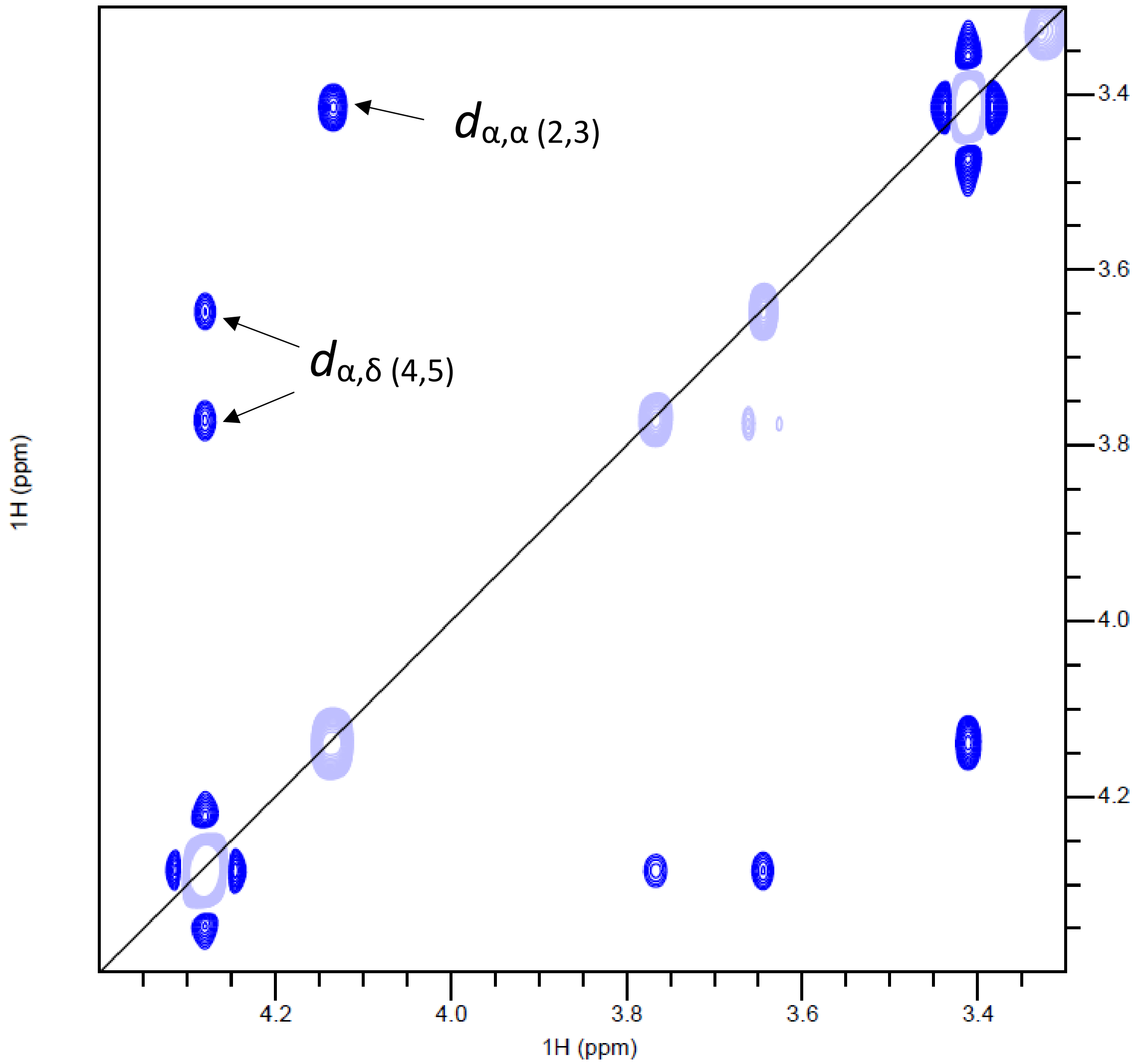

Figure S28: Selected region of 600MHz ROESY spectrum of SA-A1 in DMSO at 298K showing ROE between alpha/delta protons indicating conformation for 3Pro and 5Pro.

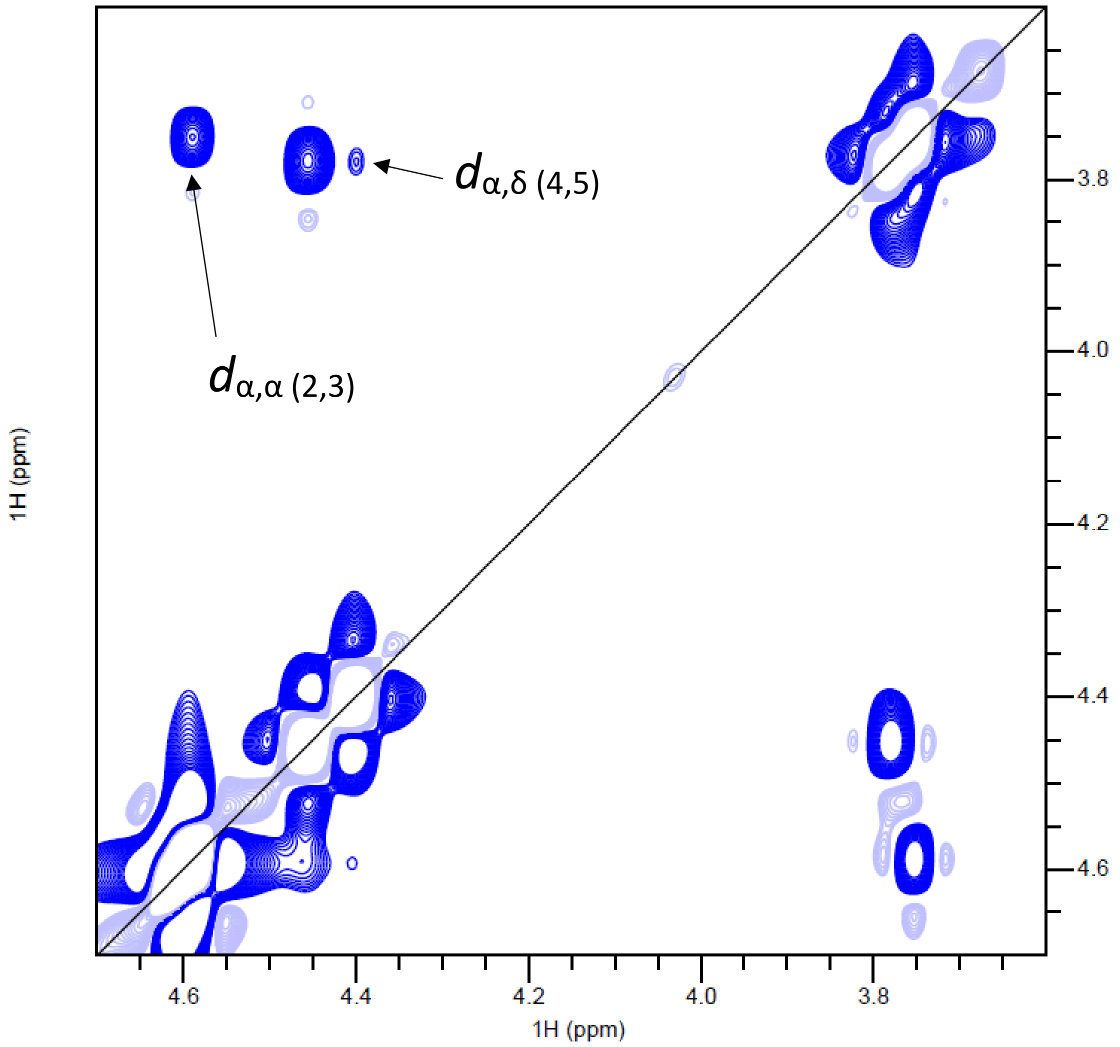

Figure S29: Selected region of 600MHz ROESY spectrum of SA-B1 in $CDCl_3$ at 298K showing ROE between alpha/delta protons indicating conformation for 3Pro and 5Pro.

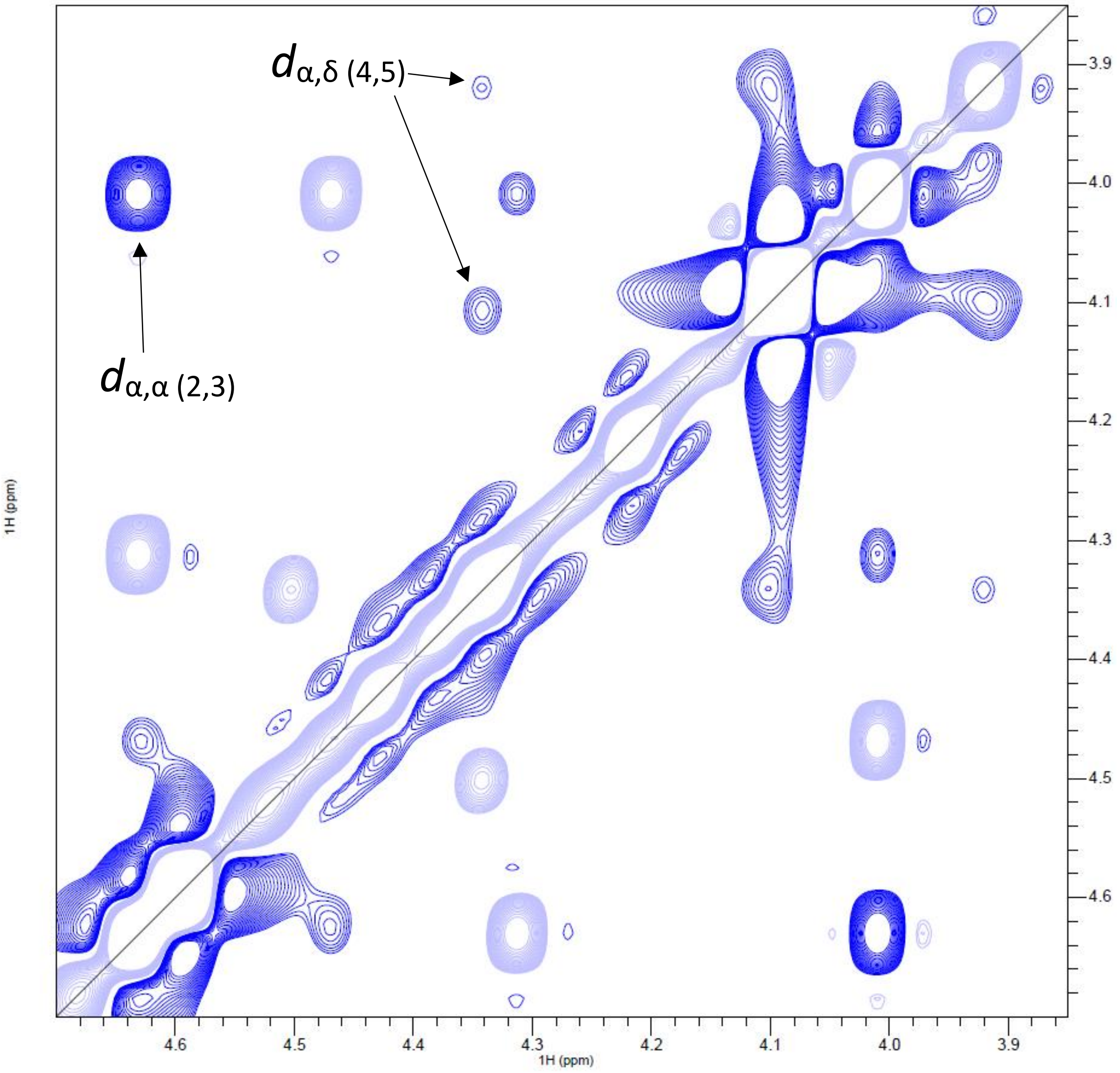

Figure S30: Selected region of 600MHz ROESY spectrum of SA-B1 in DMSO at 298K showing ROE between alpha/delta protons indicating conformation for 3Pro and 5Pro.

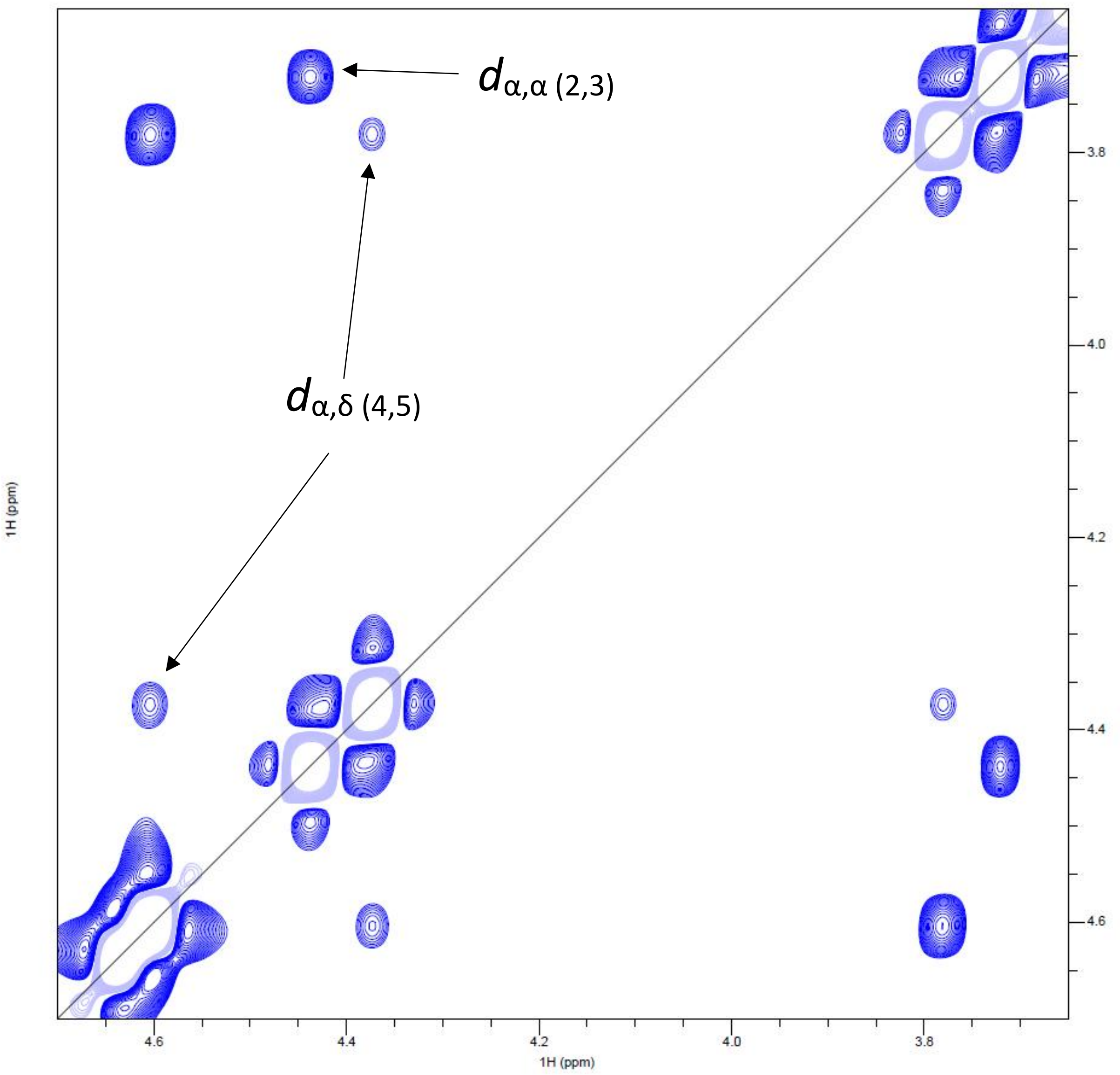

Figure S31: Selected region of 600MHz ROESY spectrum of SA-B3 in $CDCl_3$ at 298K showing ROE between alpha/delta protons indicating conformation for 3Pro and 5Pro.

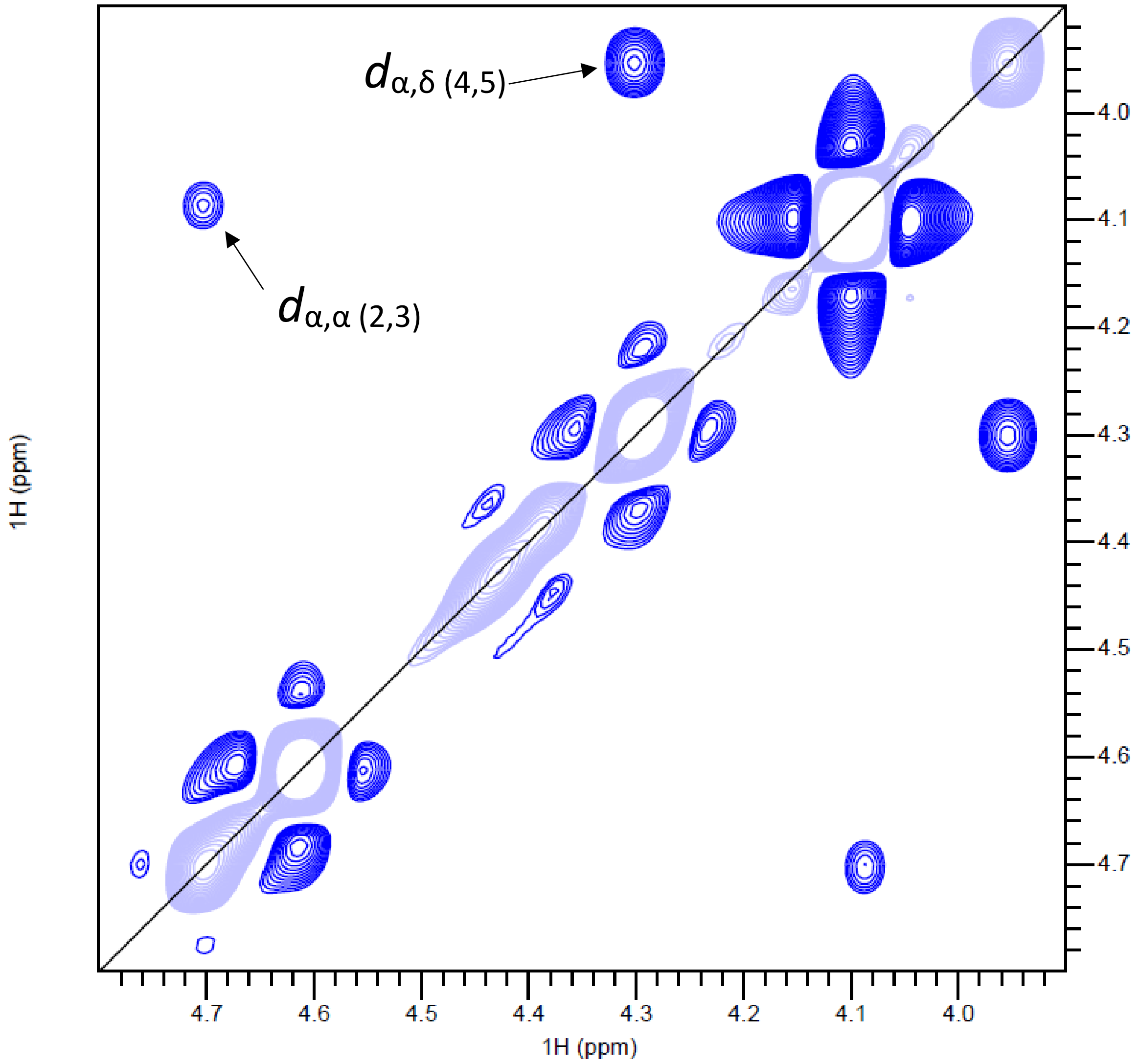

Figure S32: Selected region of 600MHz ROESY spectrum of SA-B3 in DMSO at 298K showing ROE between alpha/delta protons indicating conformation for 3Pro and 5Pro.

5. Cell Painting

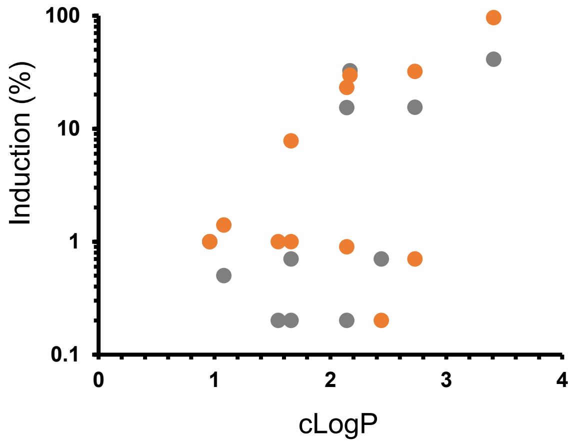

Figure S33: Correlation between cLogP and the induction value of Caco-2 cells treated with compounds at 30 µM (grey) or 50 µM (orange).

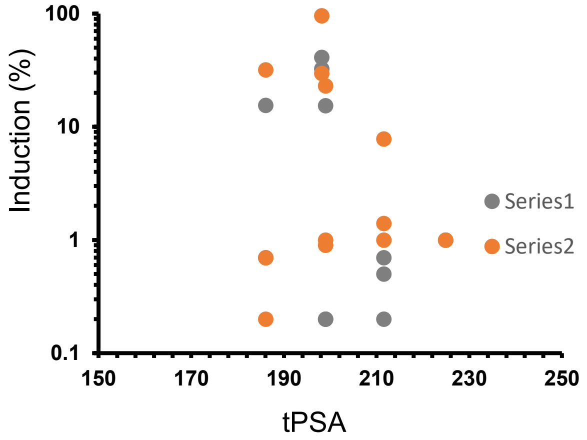

Figure S34: Correlation between tPSA and the induction value of Caco-2 cells treated with compounds at 30 µM (grey) or 50 µM (orange).

6. IAM chromatograms

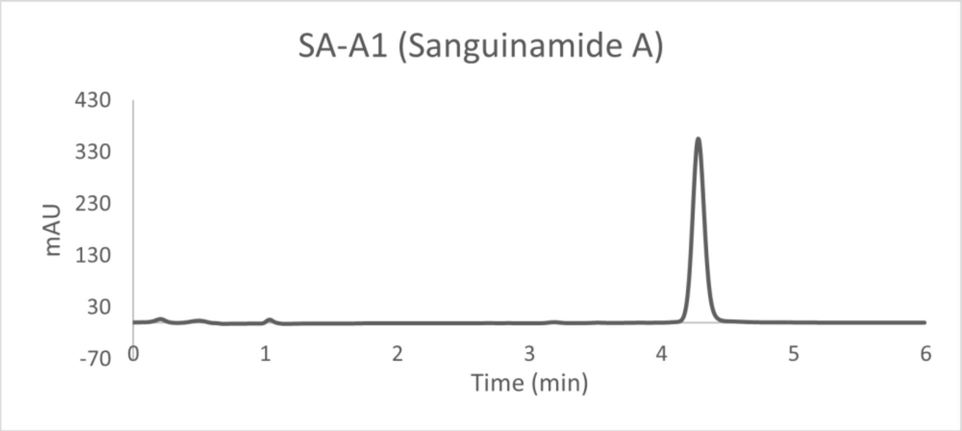

Figure S35: IAM chromatogram of SA-A1 (replicate 1)

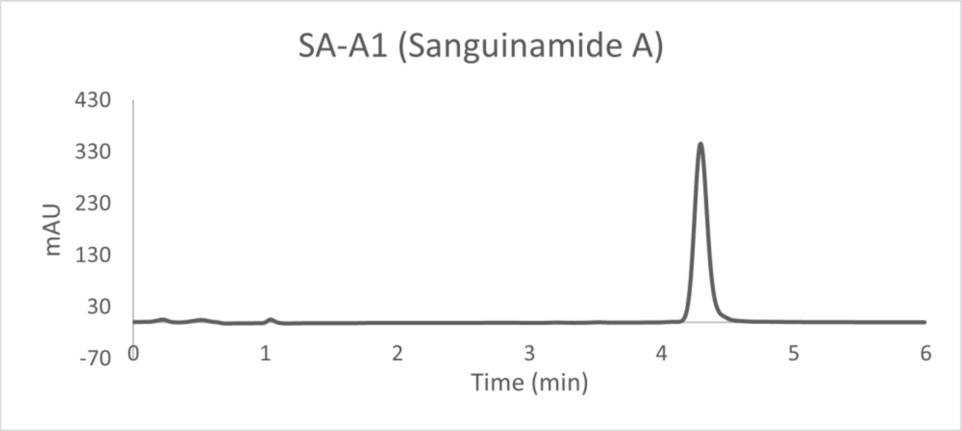

Figure S36: IAM chromatogram of SA-A1 (replicate 2)

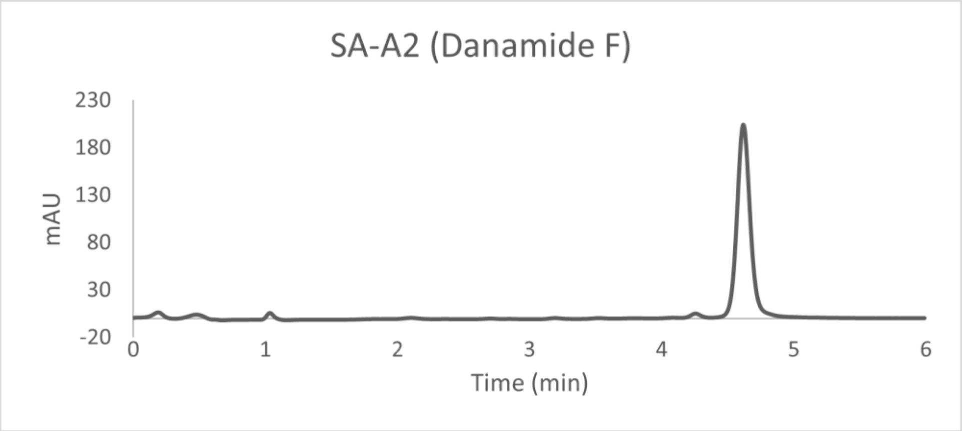

Figure S37: IAM chromatogram of SA-A2 (replicate 1)

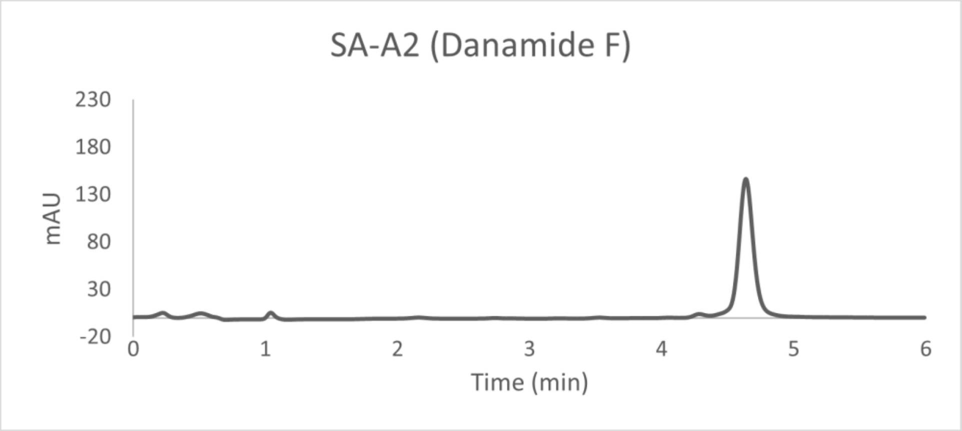

Figure S38: IAM chromatogram of SA-A2 (replicate 2)

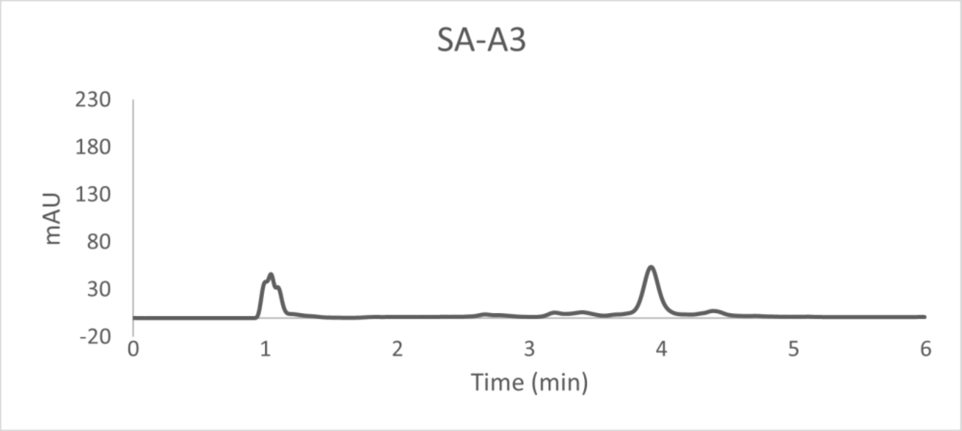

Figure S39: IAM chromatogram of SA-A3 (replicate 1)

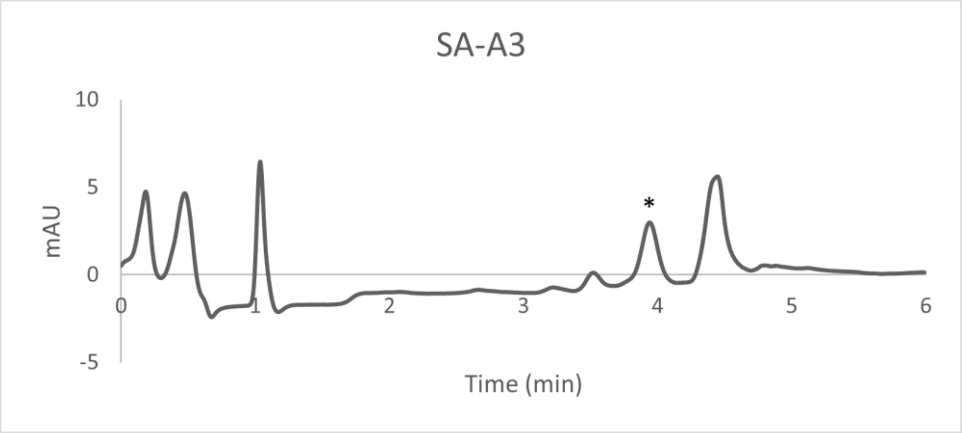

Figure S40: IAM chromatogram of SA-A3 (replicate 2). * = SA-A3 as determined by MS.

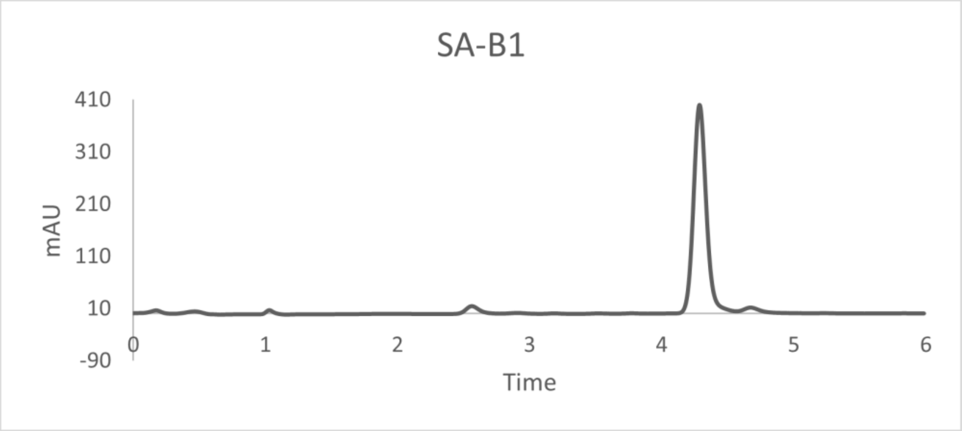

Figure S41: IAM chromatogram of SA-B1 (replicate 1)

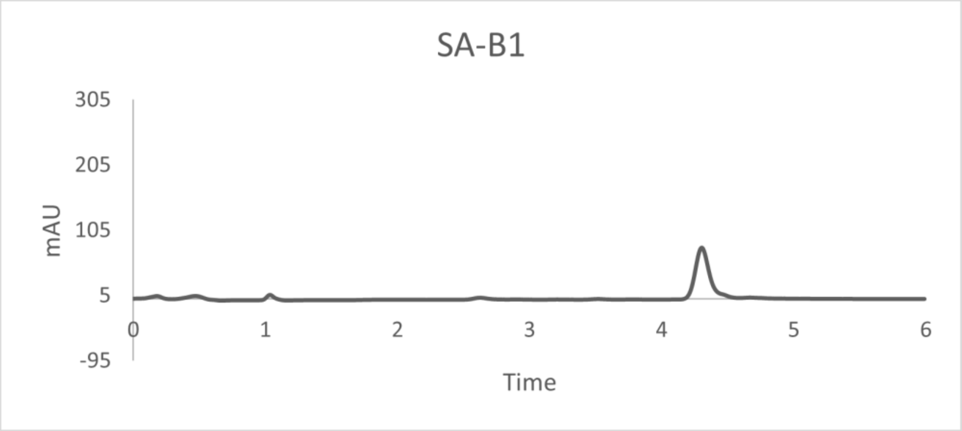

Figure S42: IAM chromatogram of SA-B1 (replicate 2)

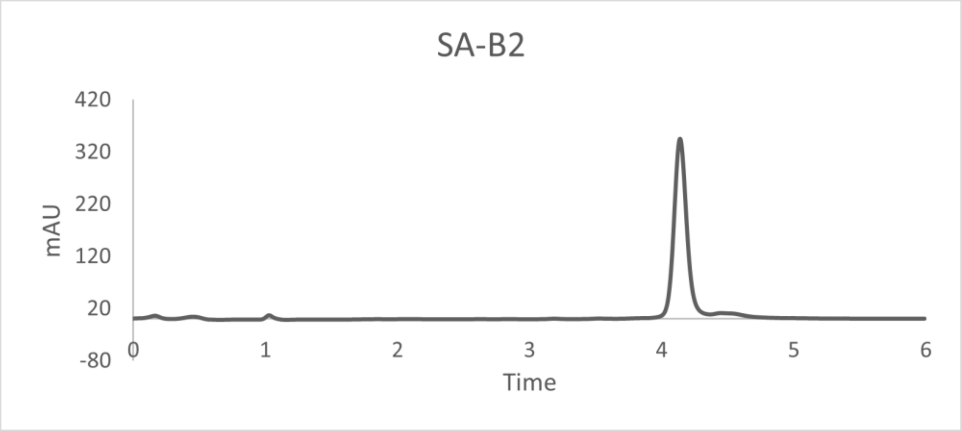

Figure S43: IAM chromatogram of SA-B2 (replicate 1)

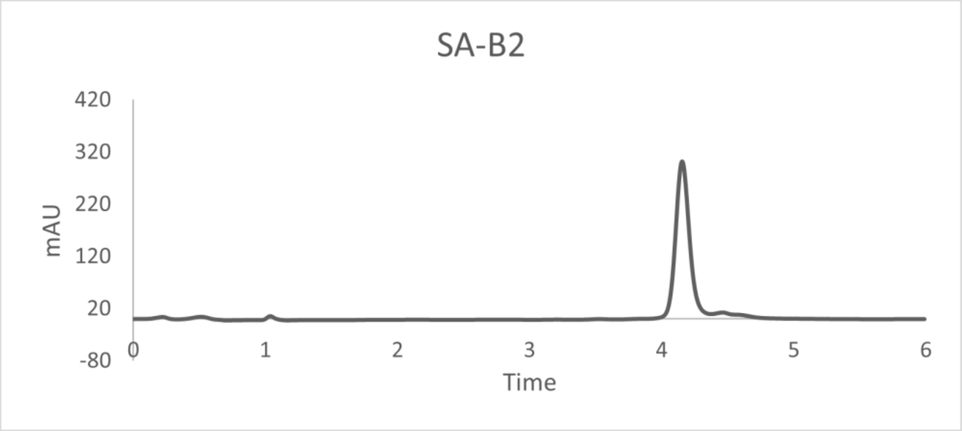

Figure S44: IAM chromatogram of SA-B2 (replicate 2)

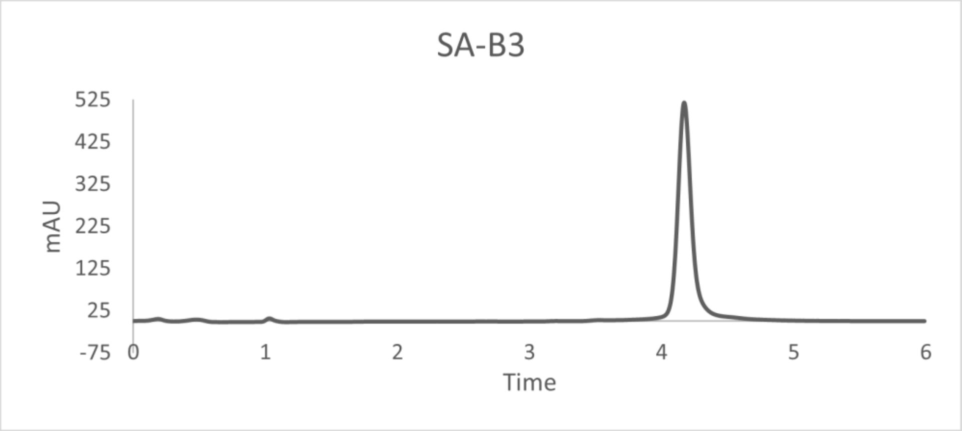

Figure S45: IAM chromatogram of SA-B3 (replicate 1)

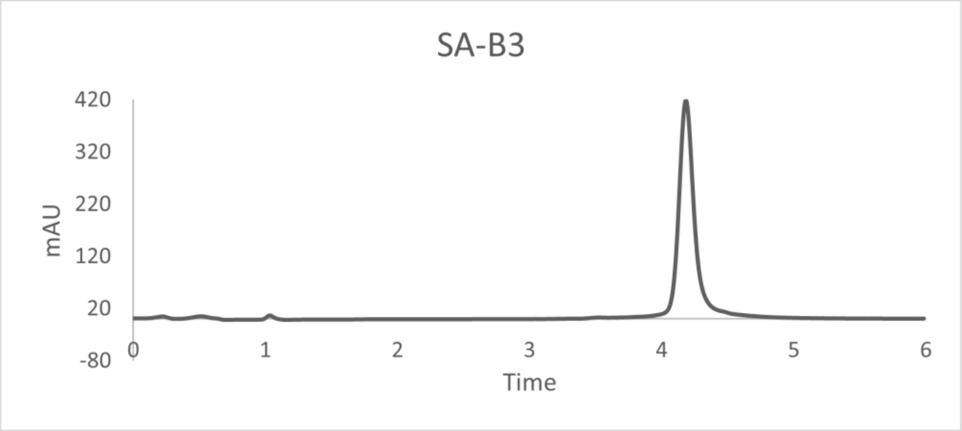

Figure S46: IAM chromatogram of SA-B3 (replicate 2)

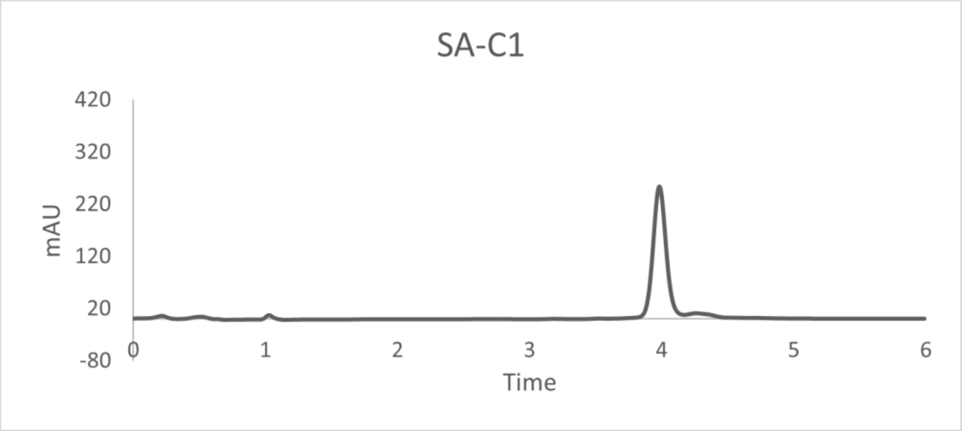

Figure S47: IAM chromatogram of SA-C1 (replicate 1)

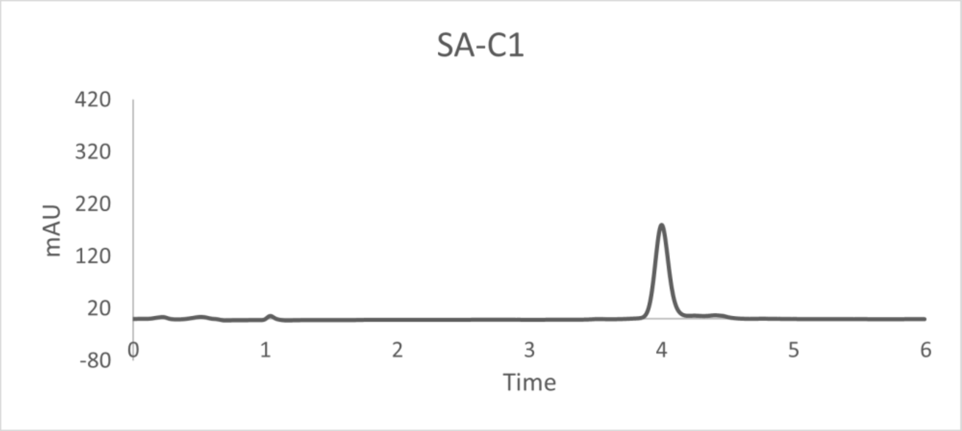

Figure S48: IAM chromatogram of SA-C1 (replicate 2)

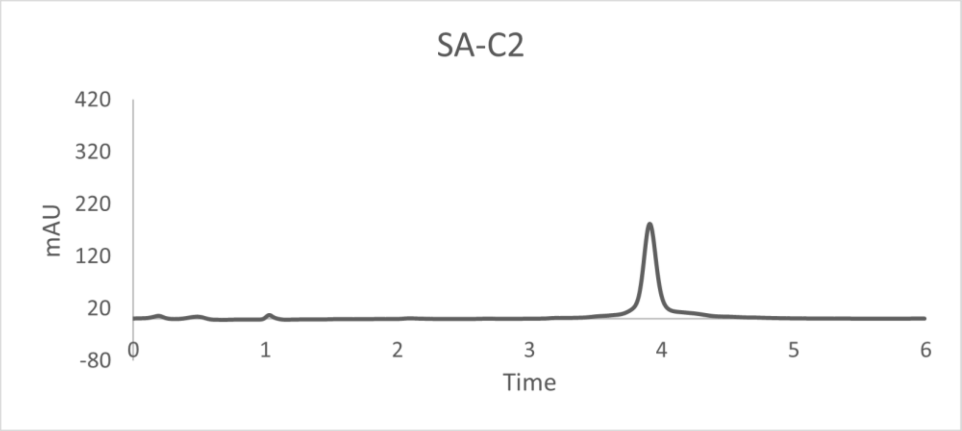

Figure S49: IAM chromatogram of SA-C2 (replicate 1)

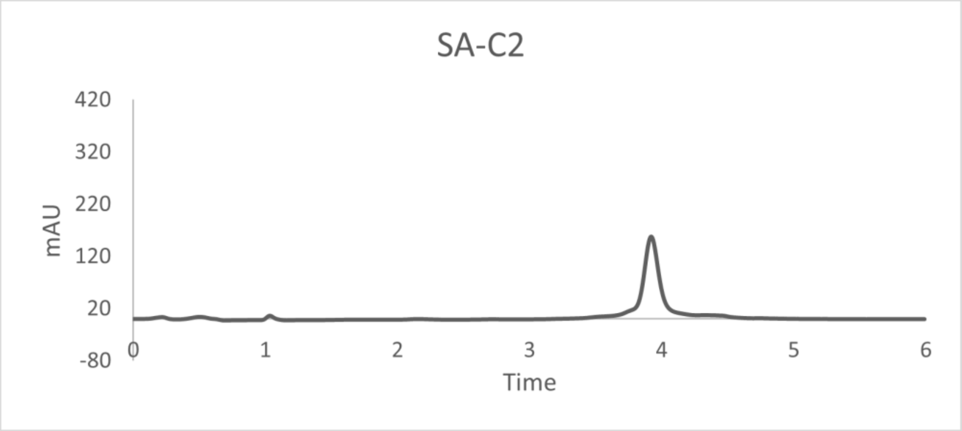

Figure S50: IAM chromatogram of SA-C2 (replicate 2)

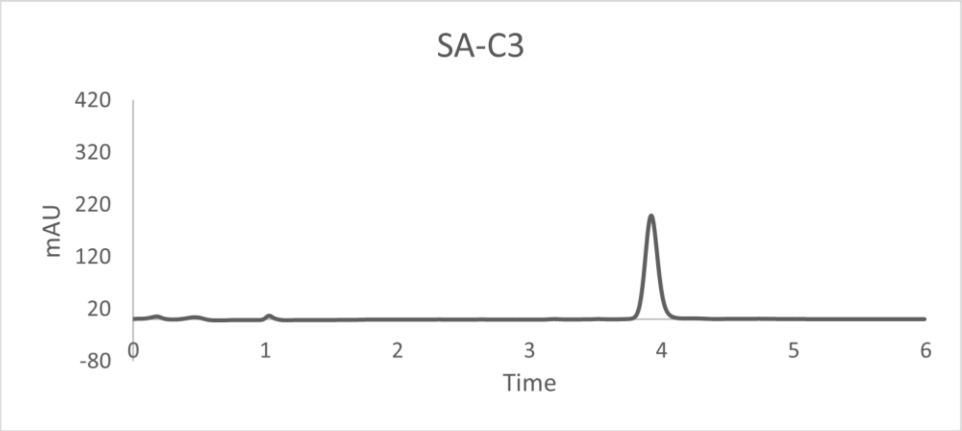

Figure S51: IAM chromatogram of SA-C3 (replicate 1)

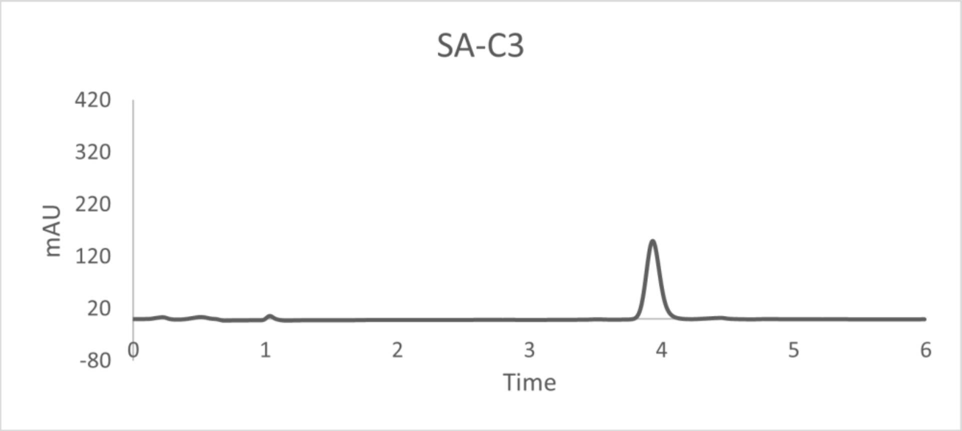

Figure S52: IAM chromatogram of SA-C3 (replicate 2)

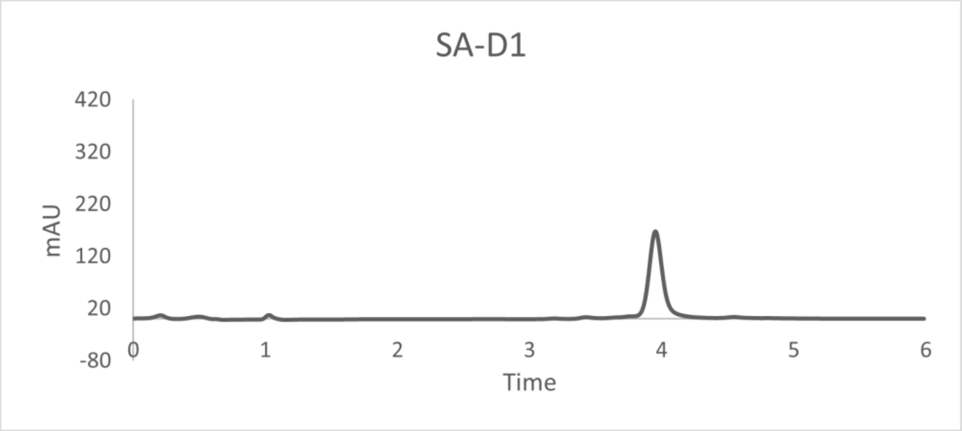

Figure S53: IAM chromatogram of SA-D1 (replicate 1)

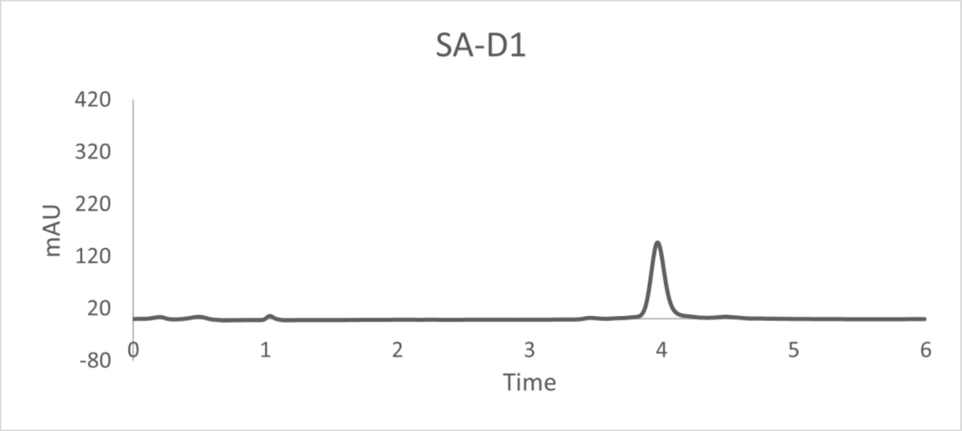

Figure S54: IAM chromatogram of SA-D1 (replicate 2)

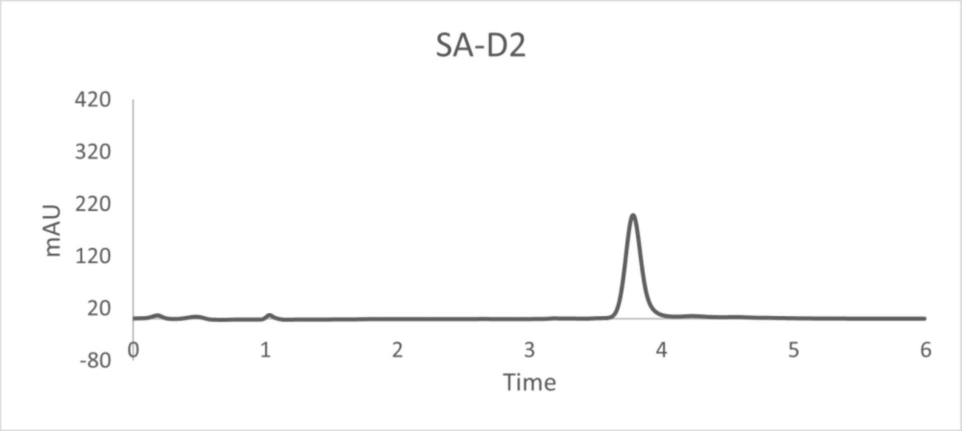

Figure S55: IAM chromatogram of SA-D2 (replicate 1)

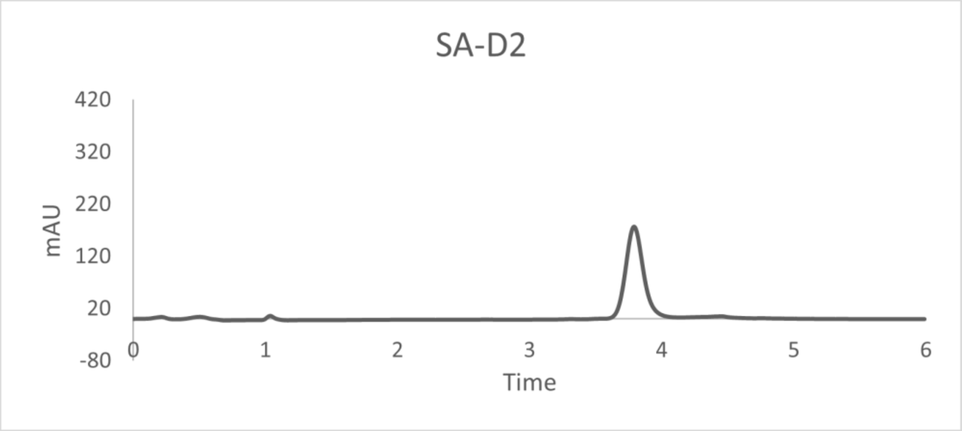

Figure S56: IAM chromatogram of SA-D2 (replicate 2)

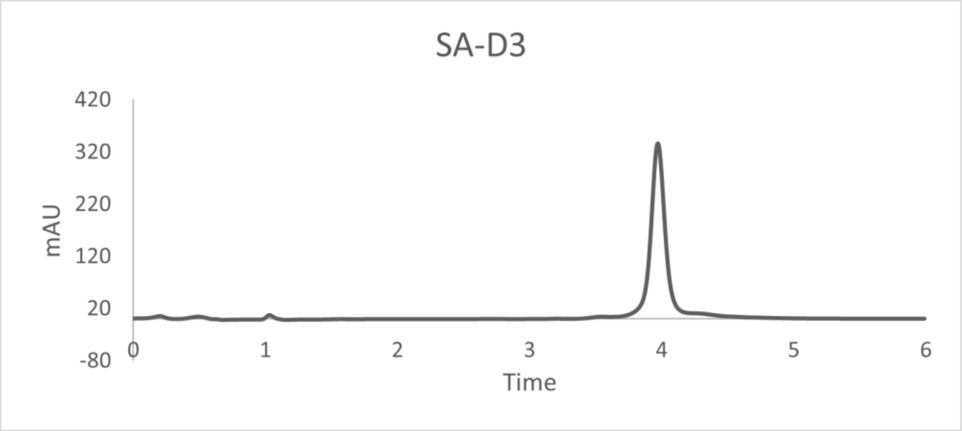

Figure S57: IAM chromatogram of SA-D3 (replicate 1)

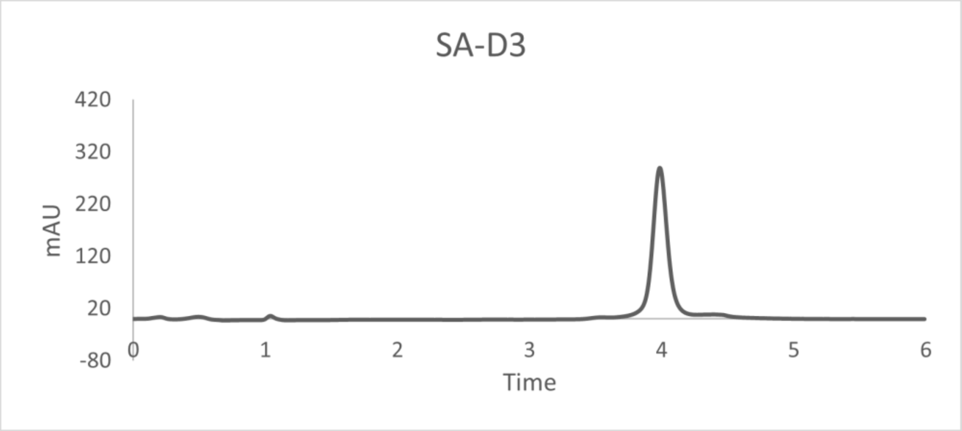

Figure S58: IAM chromatogram of SA-D3 (replicate 2)

7. Compound characterization

HPLC chromatograms peptides

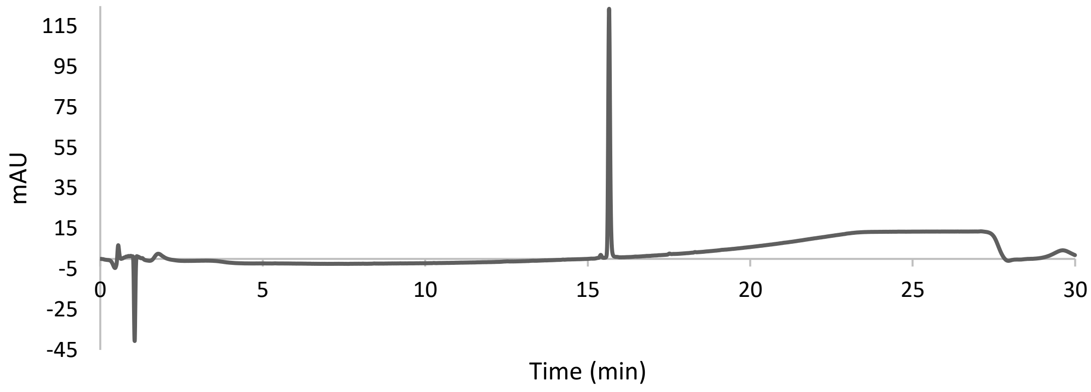

Figure S59: UPLC trace for peptide SA-A1

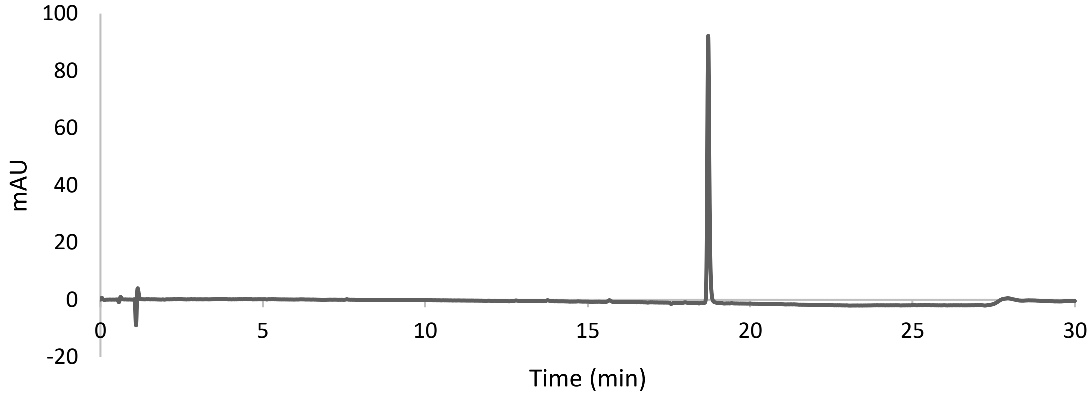

Figure S60: UPLC trace for peptide SA-A2

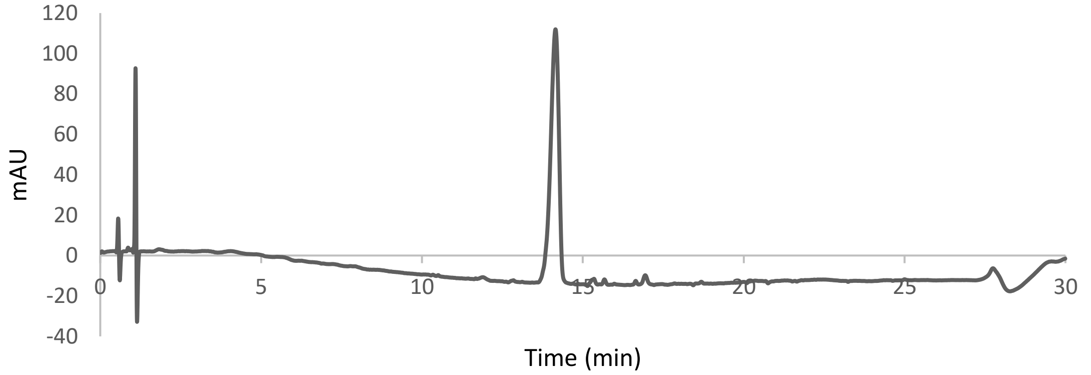

Figure S61: UPLC trace for peptide SA-A3

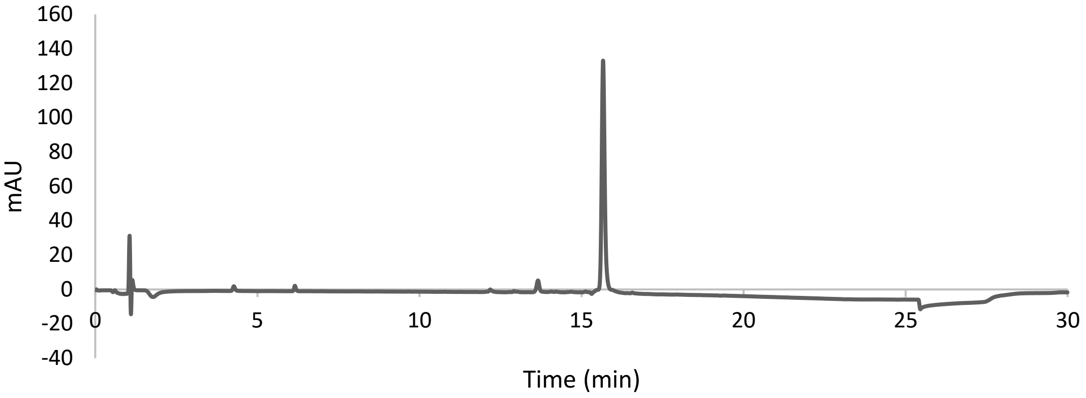

Figure S62: UPLC trace for peptide SA-B1

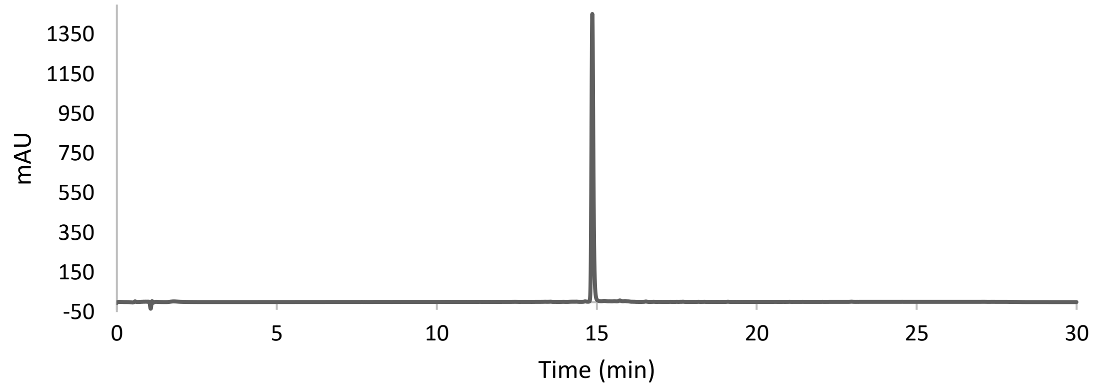

Figure S63: UPLC trace for peptide SA-B2

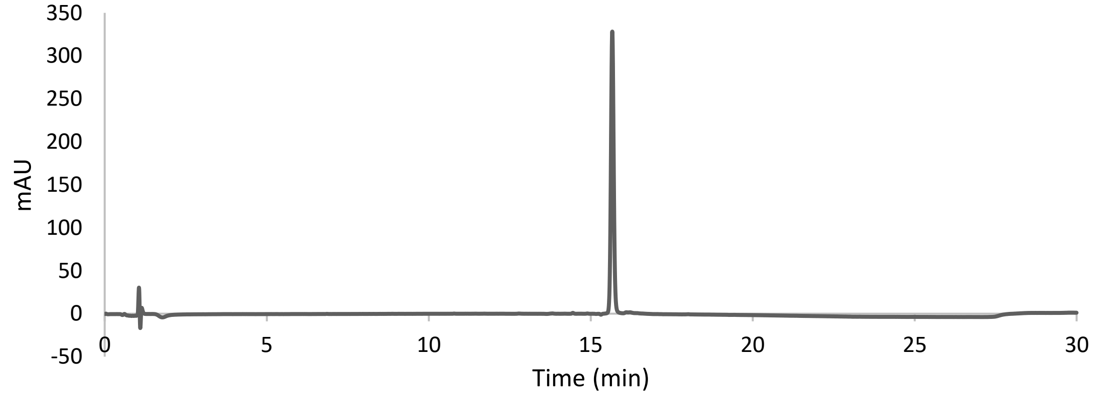

Figure S64: UPLC trace for peptide SA-B3

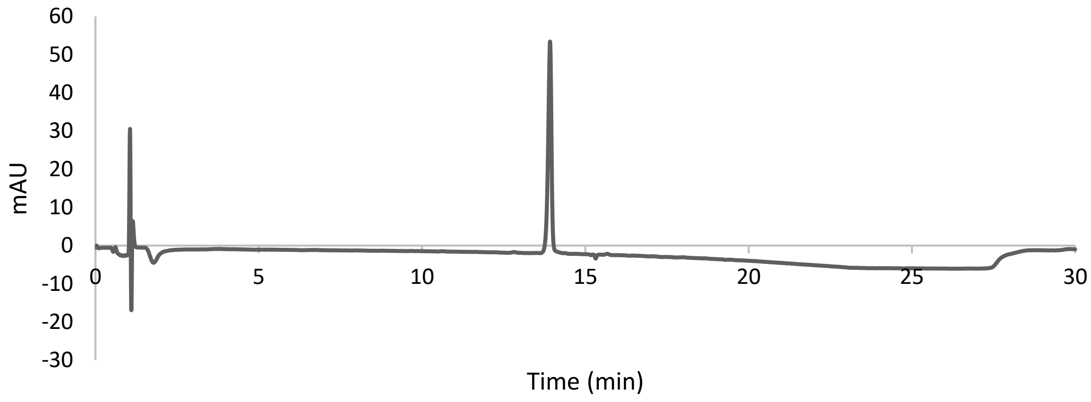

Figure S65: UPLC trace for peptide SA-C1

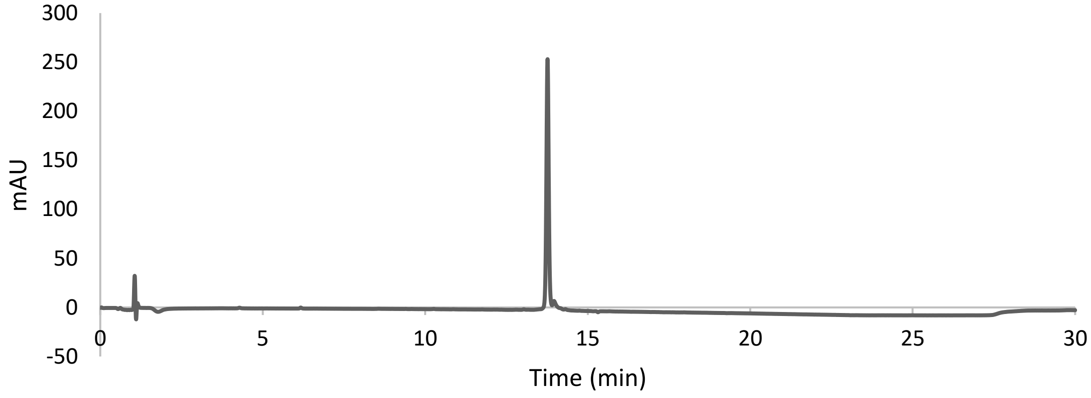

Figure S66: UPLC trace for peptide SA-C2

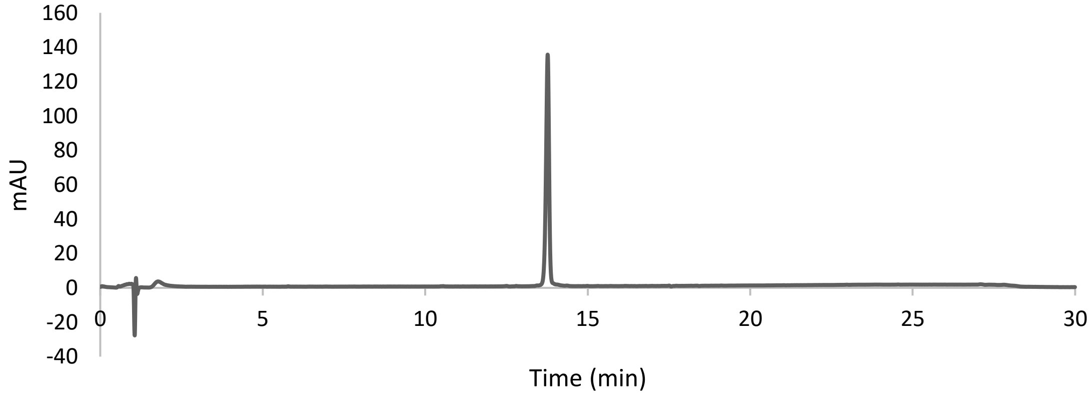

Figure S67: UPLC trace for peptide SA-C3

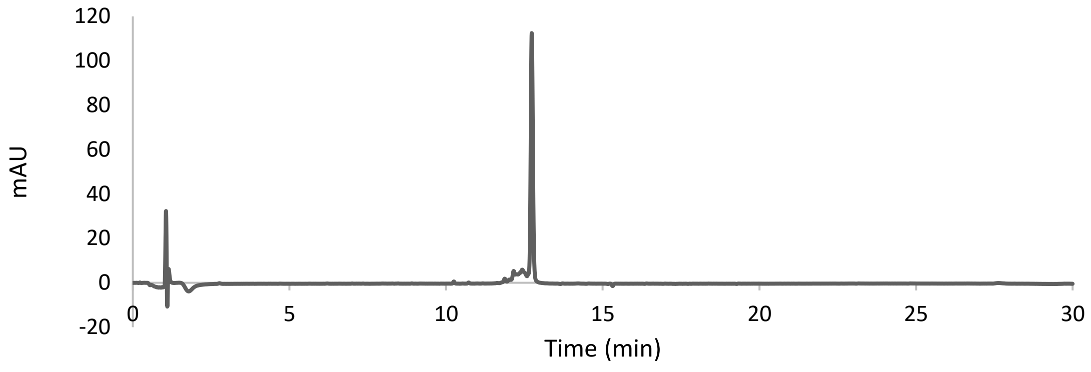

Figure S68: UPLC trace for peptide SA-D1

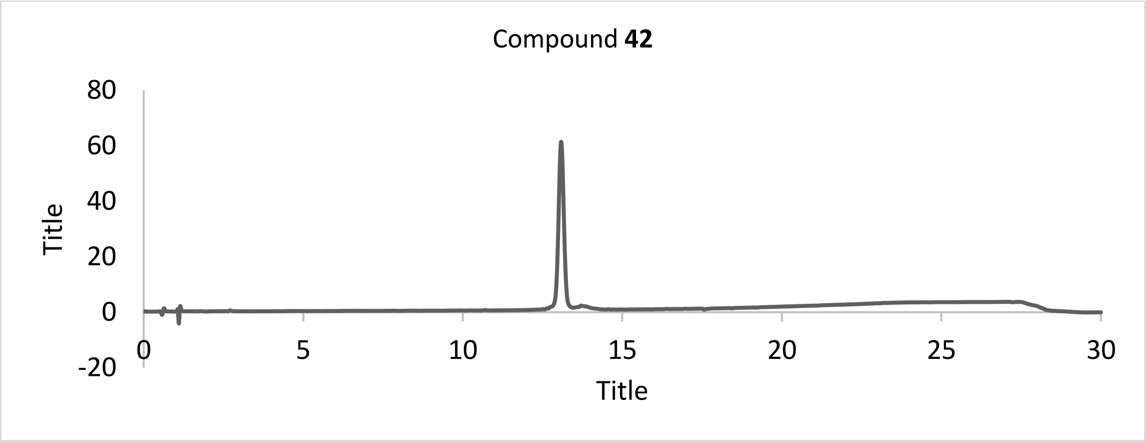

Figure S69: UPLC trace for peptide SA-D2

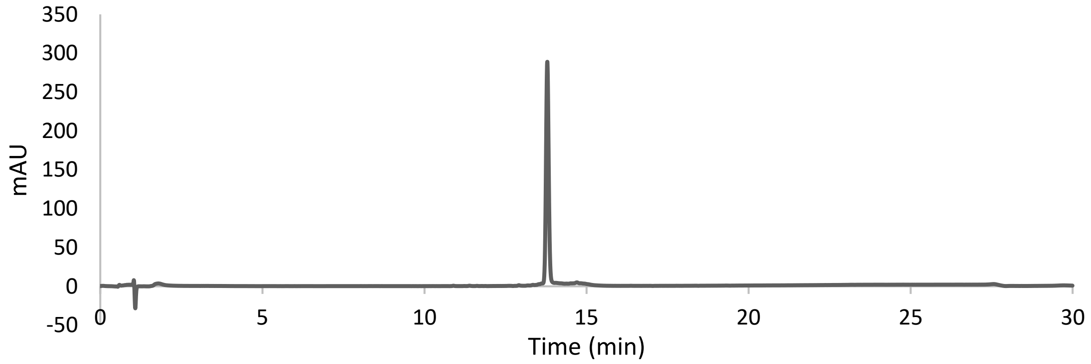

Figure S70: UPLC trace for peptide SA-D3

NMR spectra small molecules

1 ^{1}H NMR

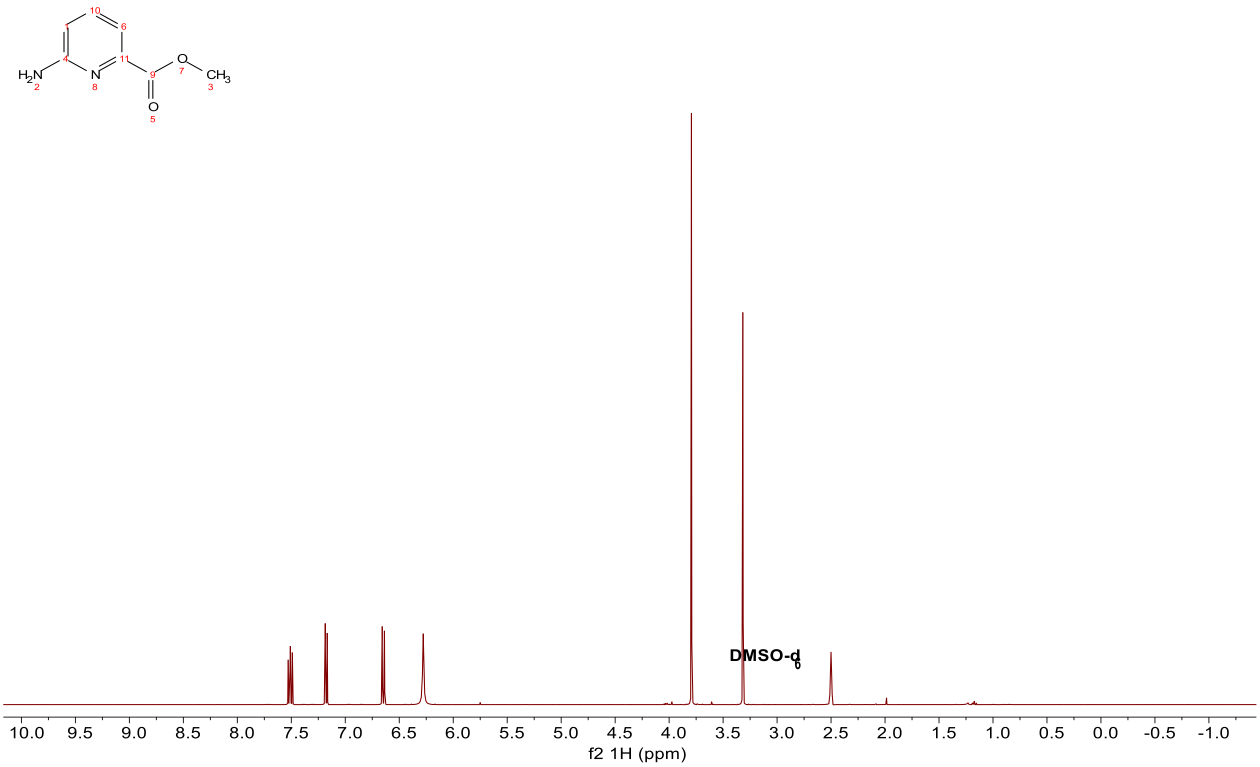

1 ^{13}C NMR

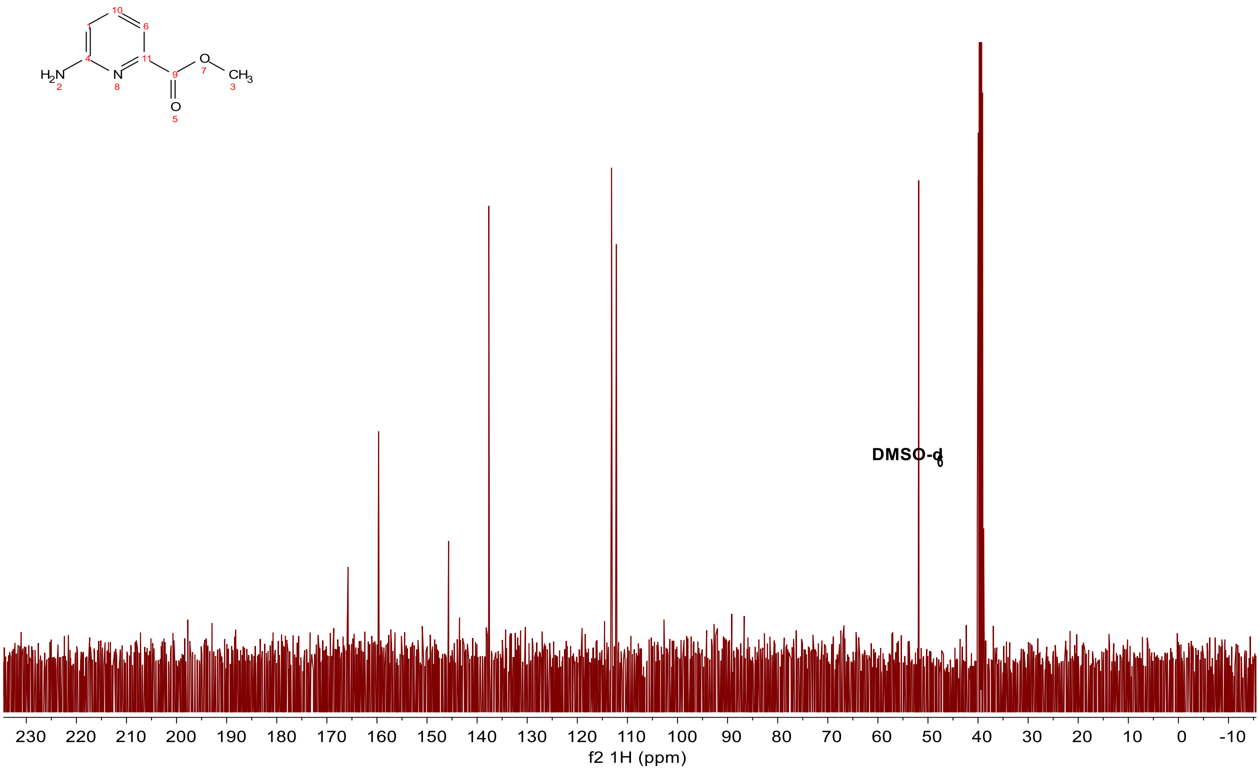

2 ^{1}H NMR

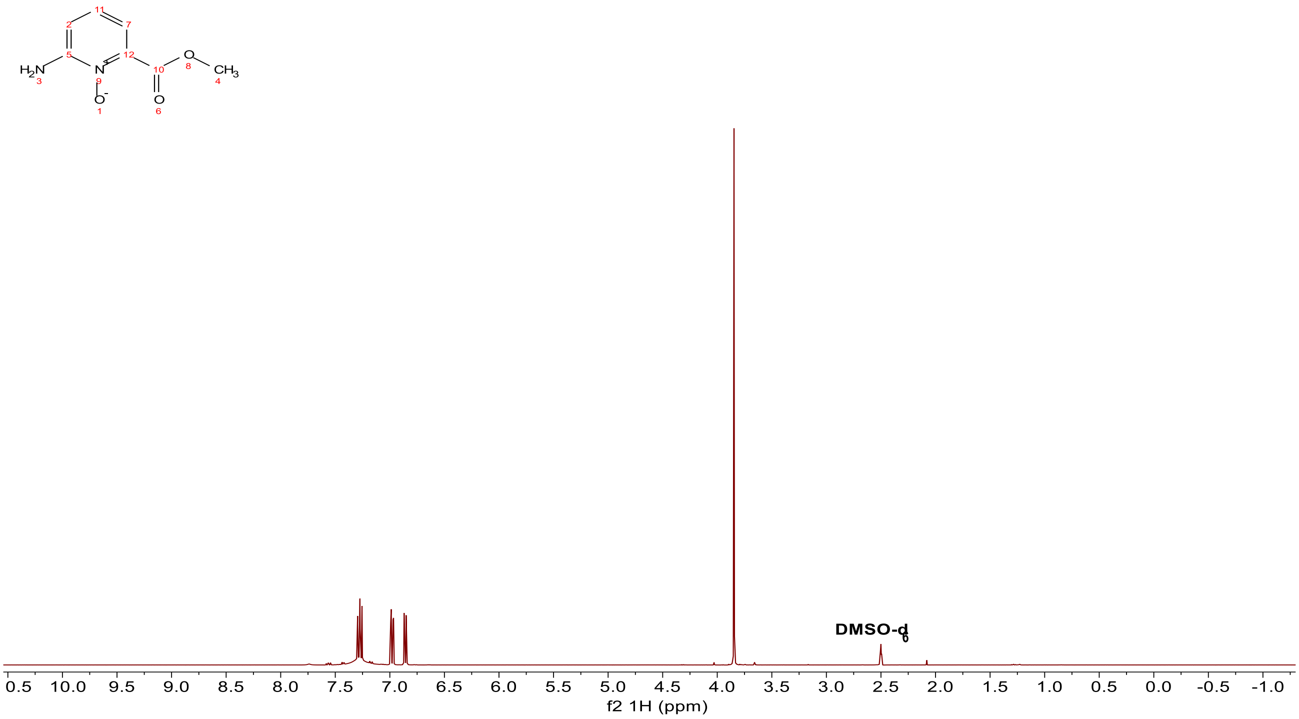

2 ^{13}C NMR

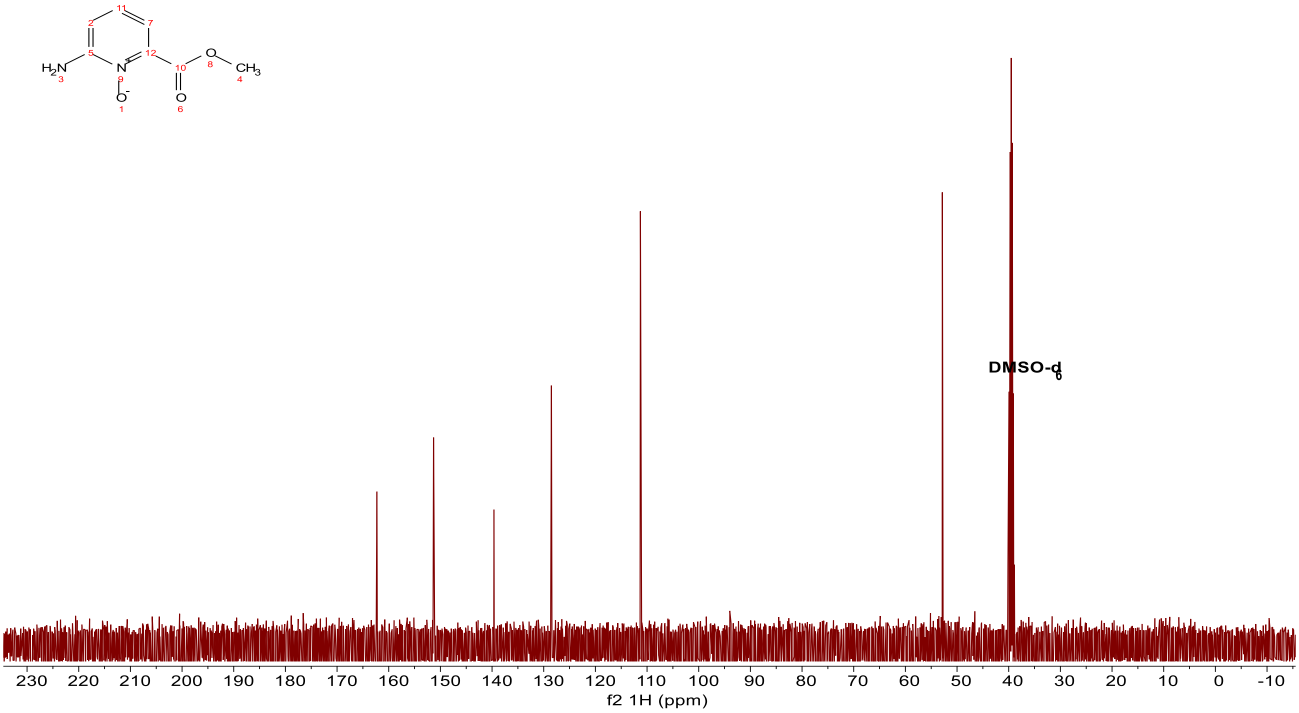

3 ^{1}H NMR

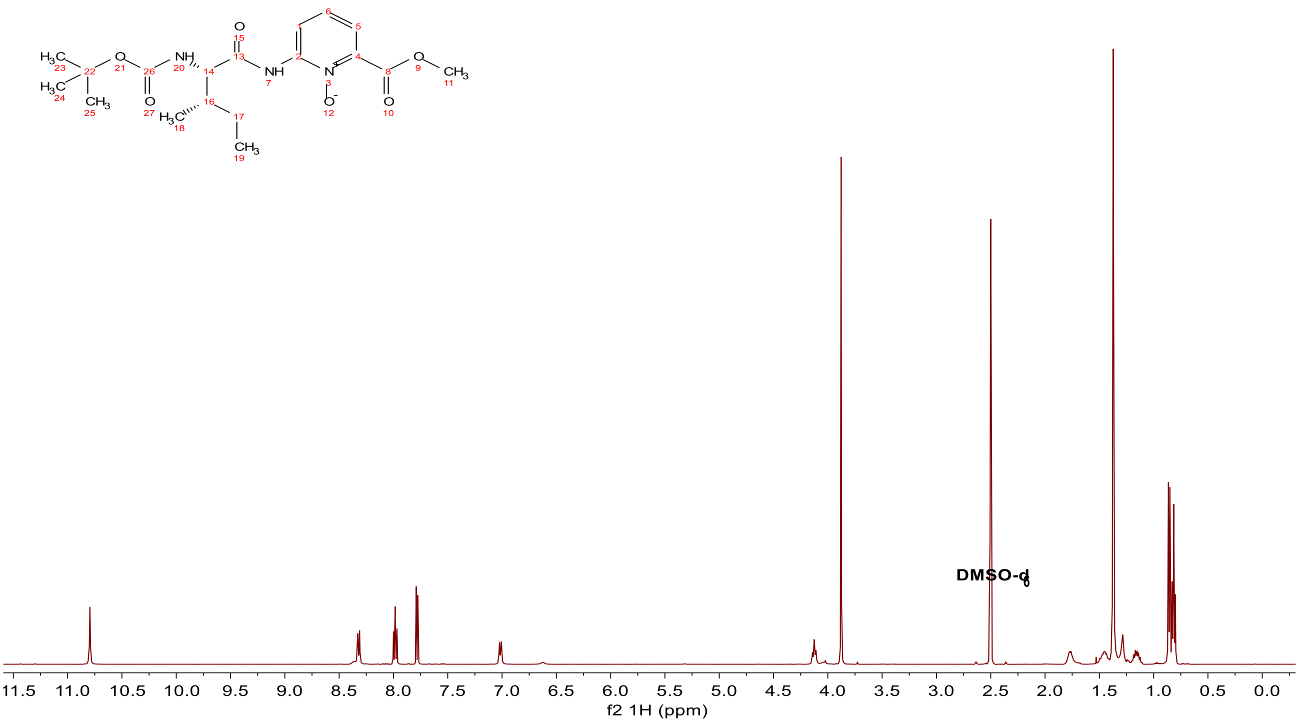

3 ^{13}C NMR

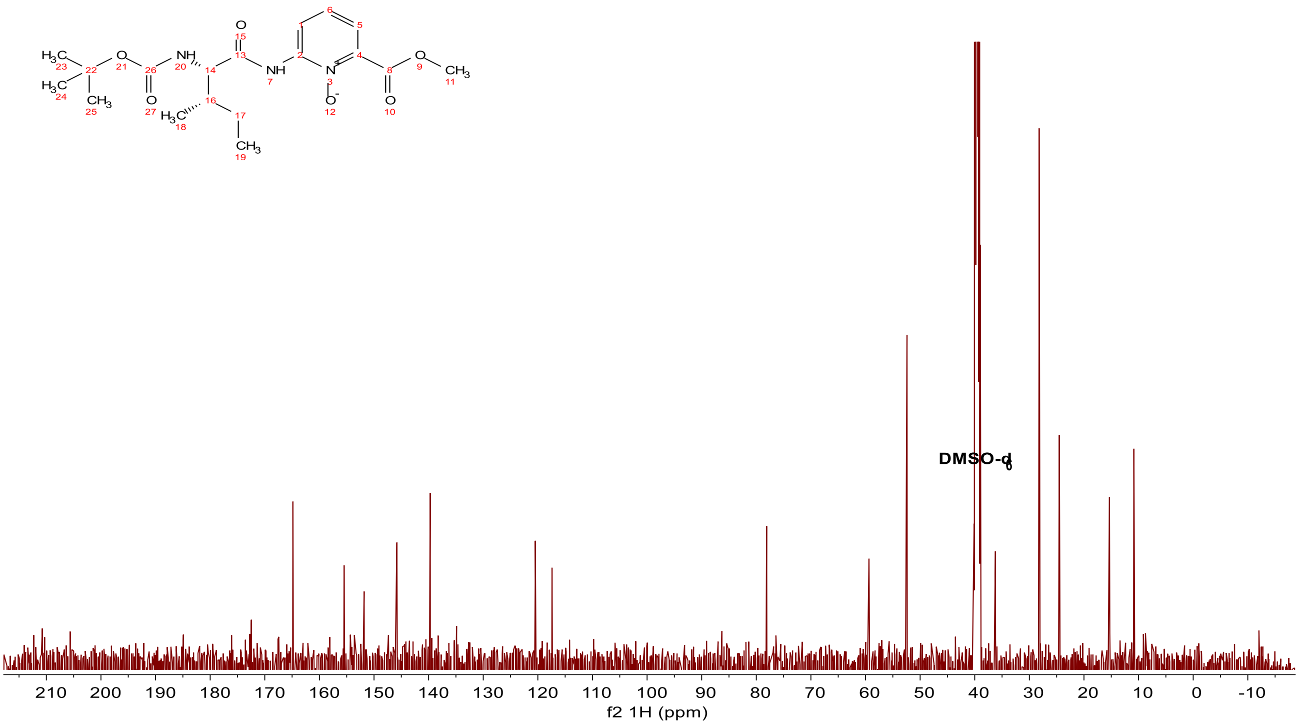

4 ^{1}H NMR

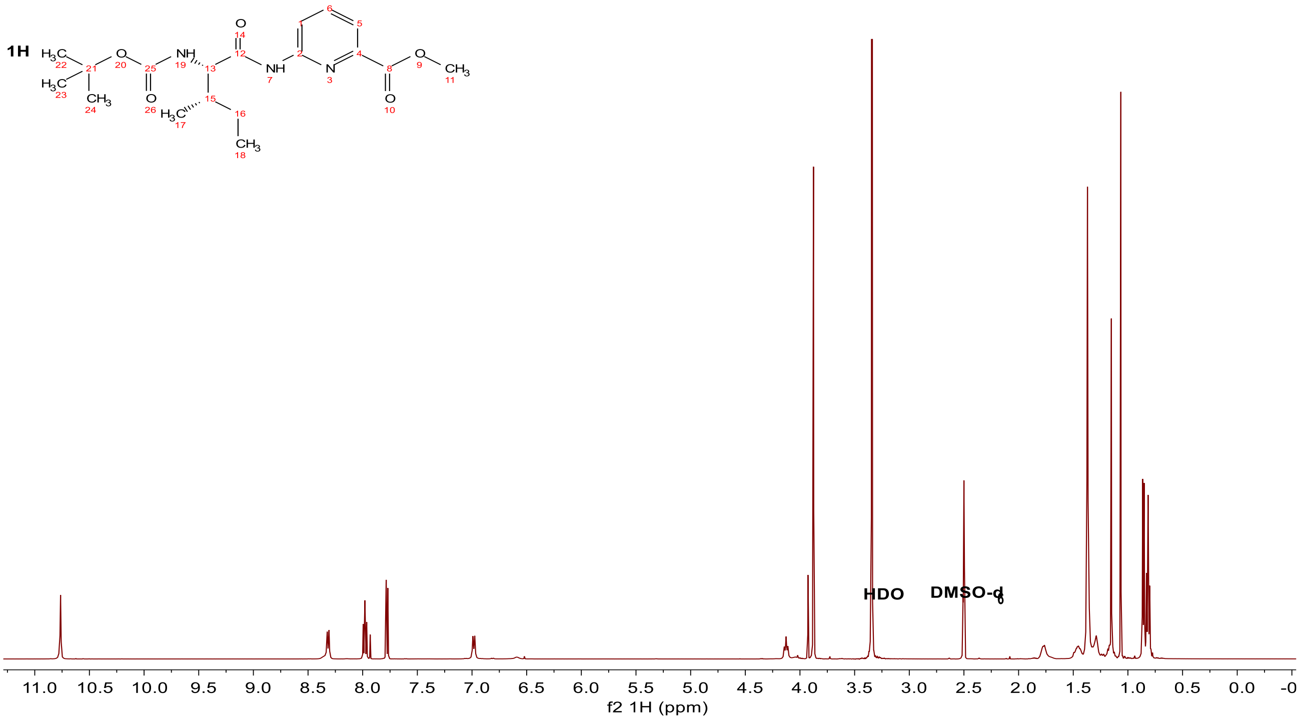

4 ^{13}C NMR

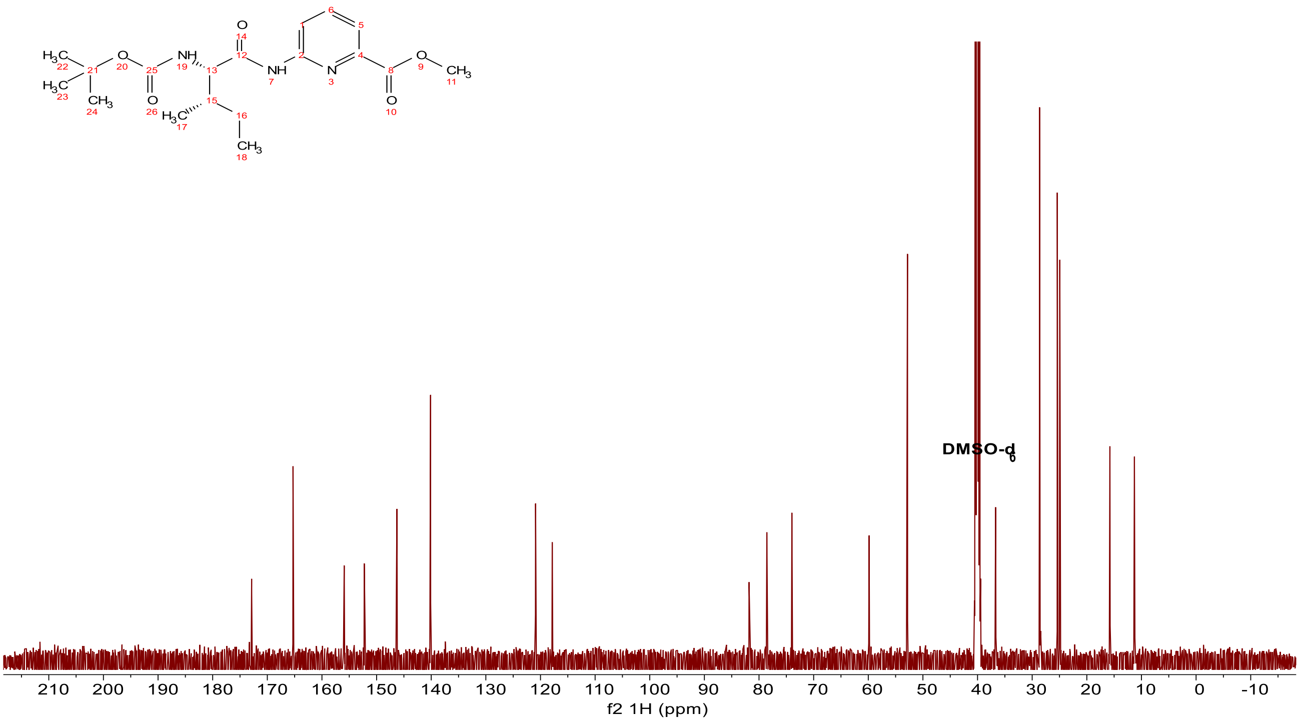

5 ^{1}H NMR

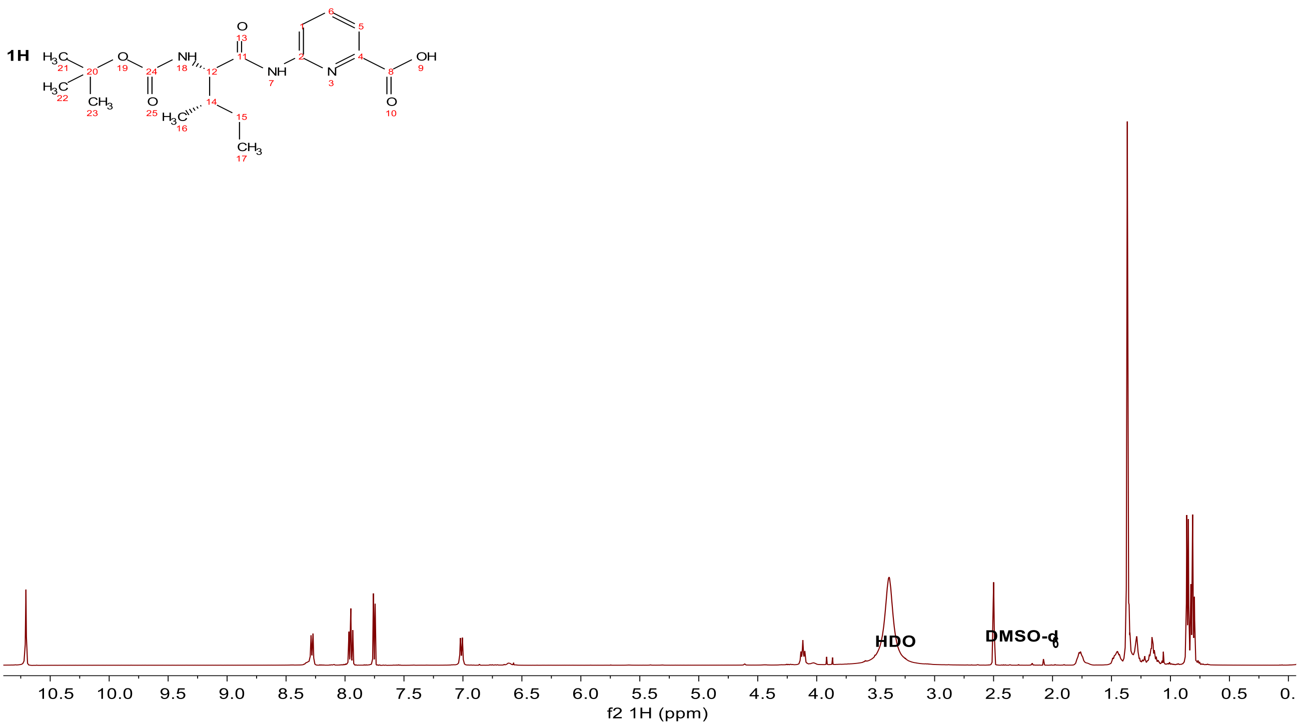

5 ^{13}C NMR

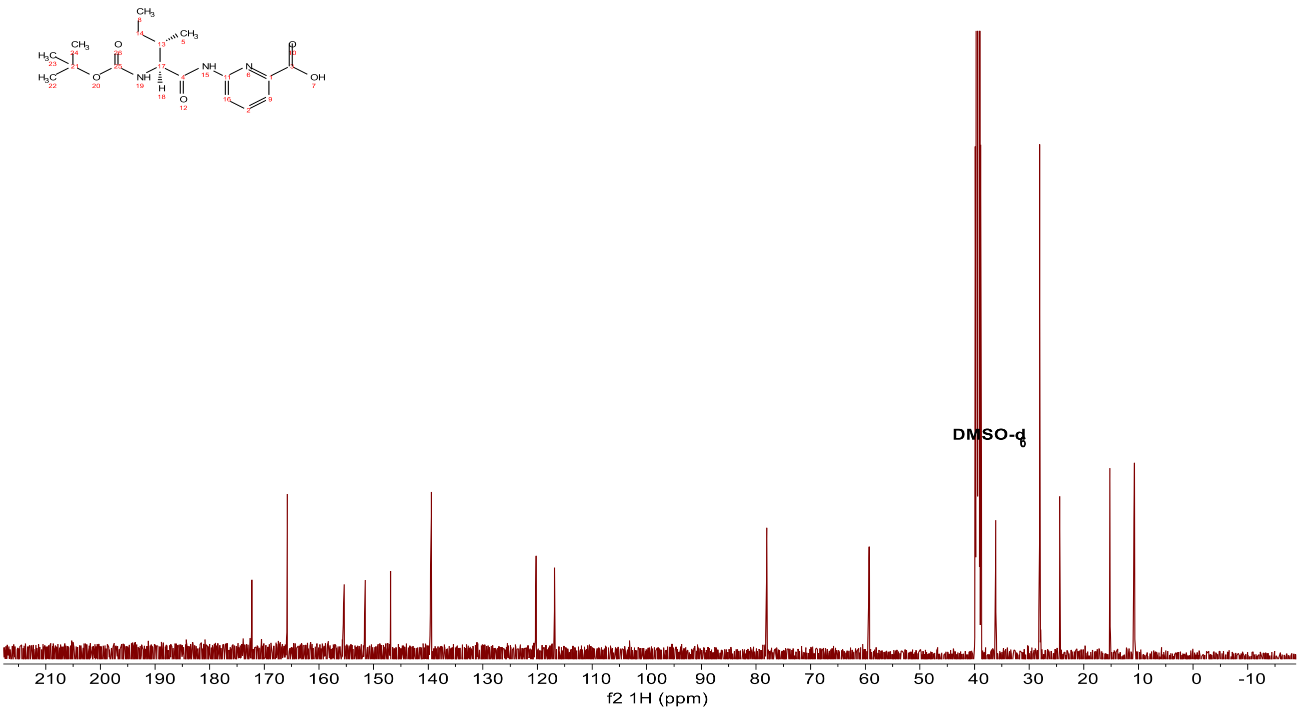

6 ^{1}H NMR

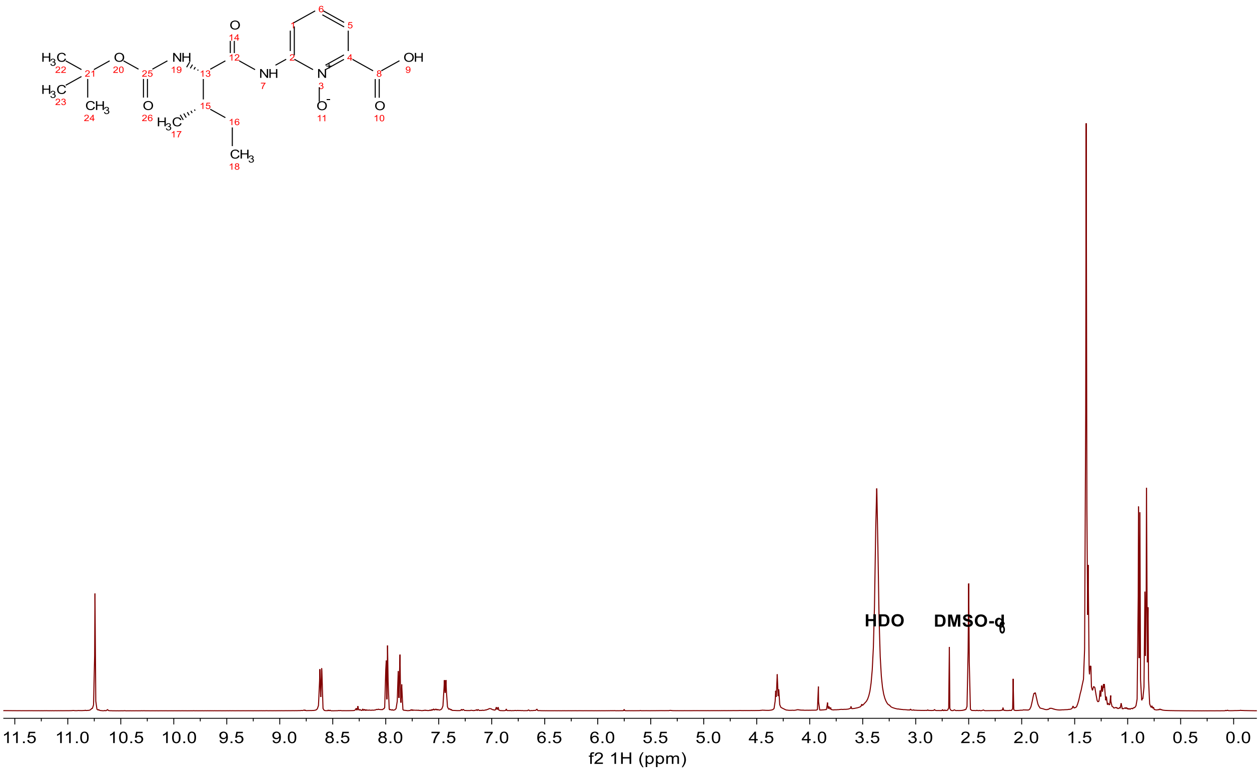

6 ^{13}C NMR

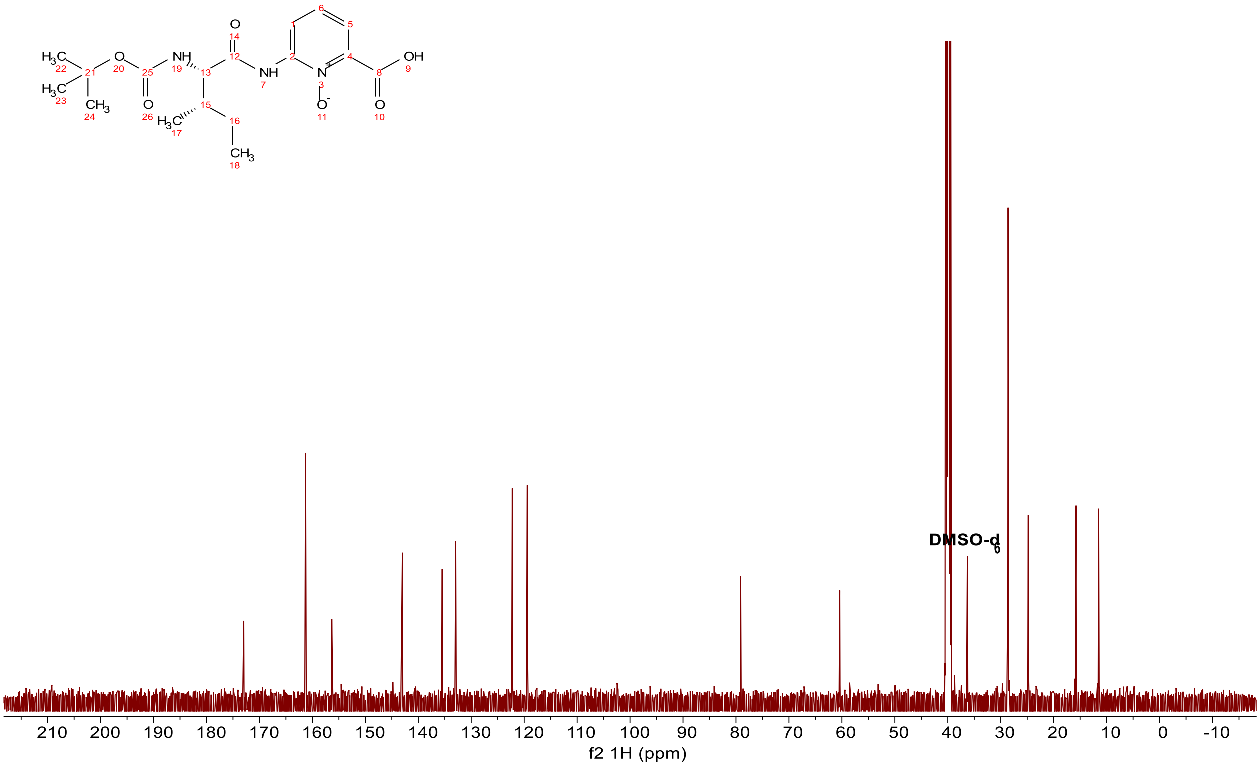

7 ^{1}H NMR

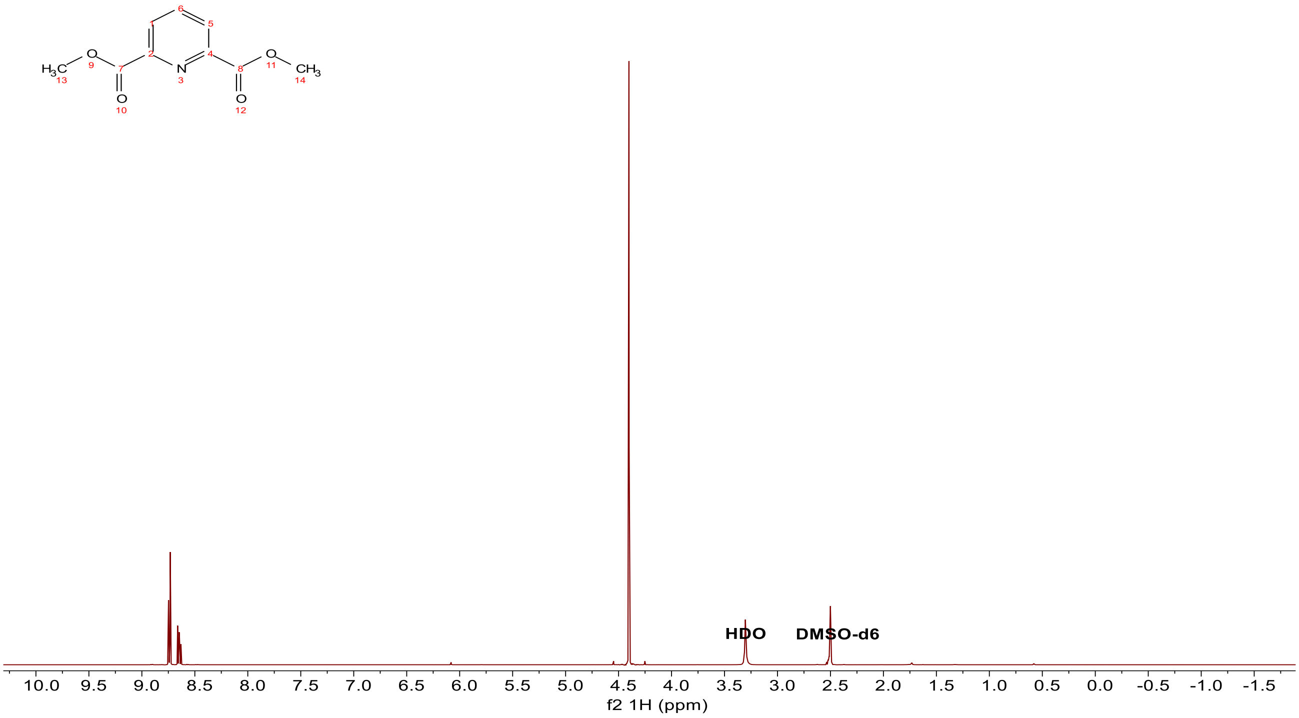

7 ^{13}C NMR

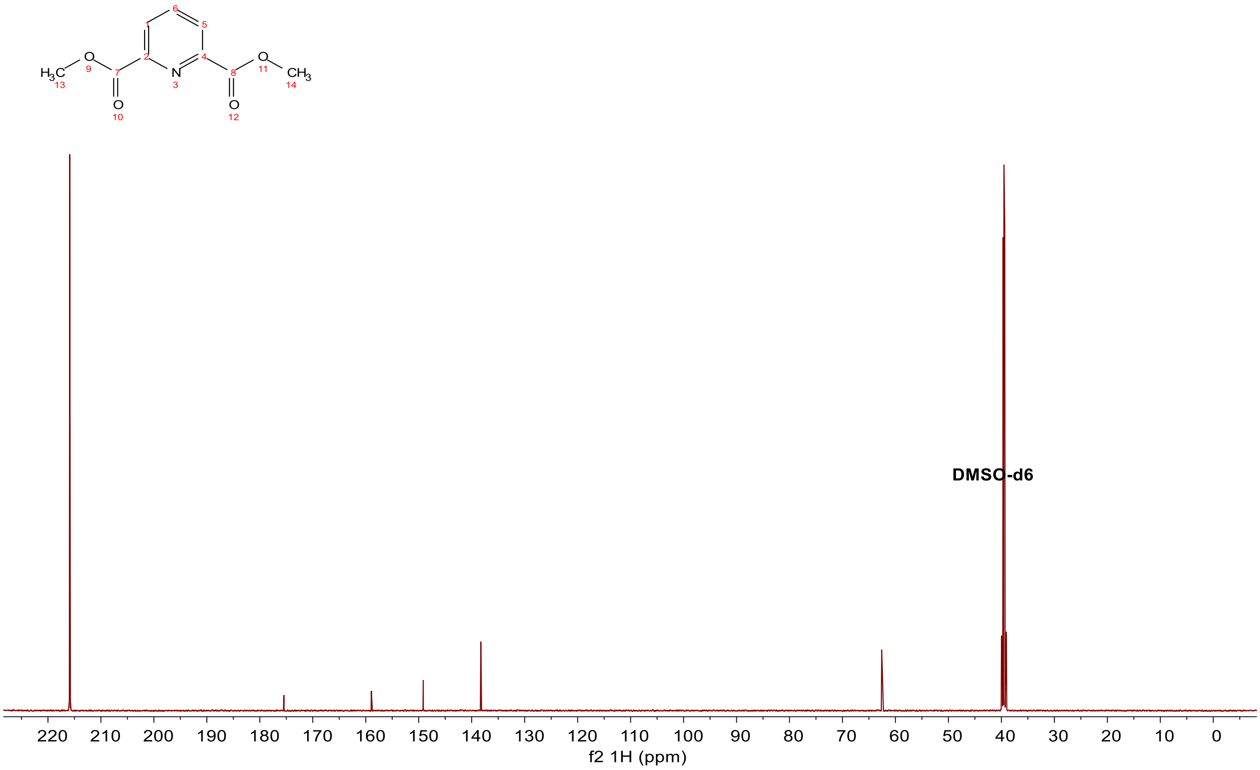

9 ^{1}H NMR

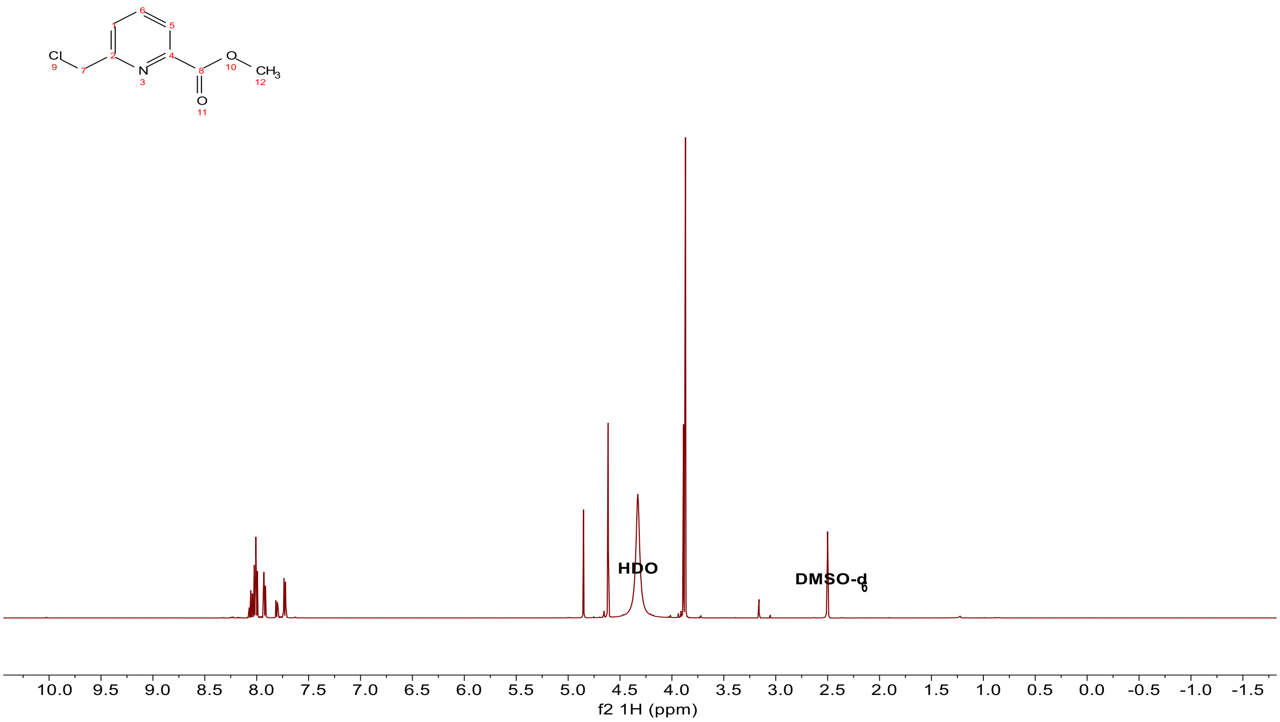

9 ^{13}C NMR

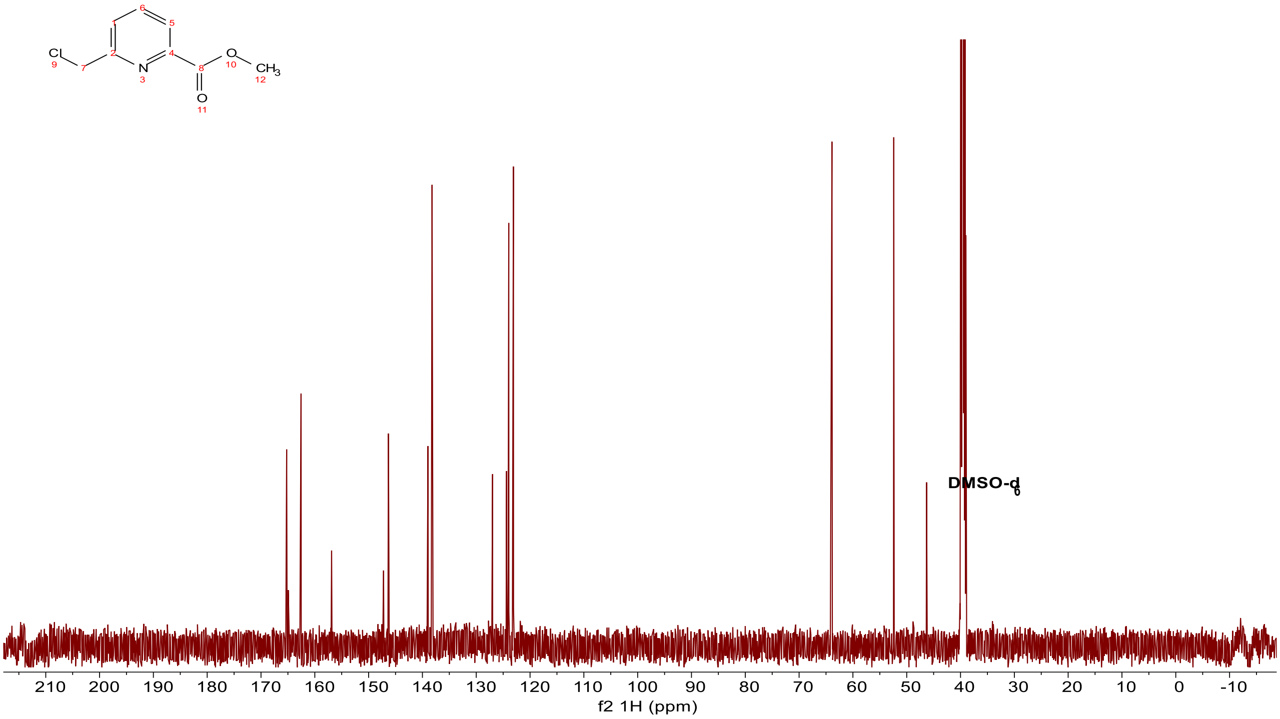

10 ^{1}H NMR

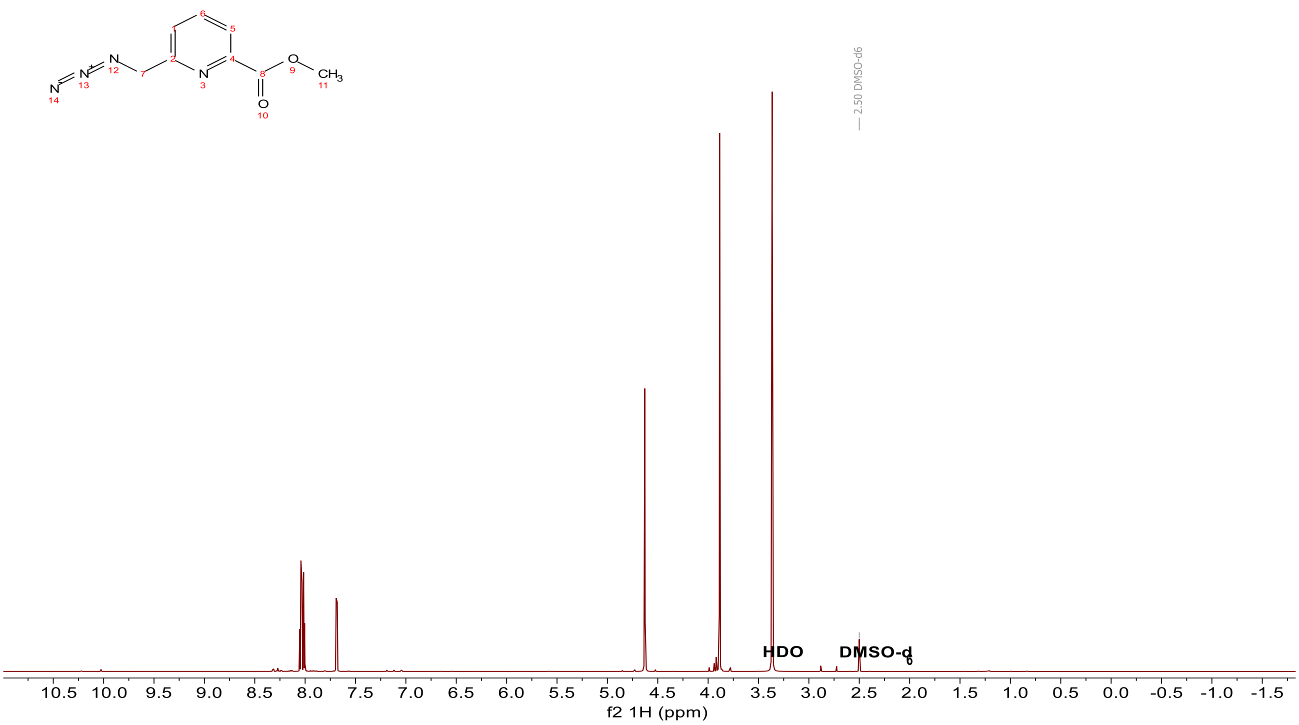

10 ^{13}C NMR

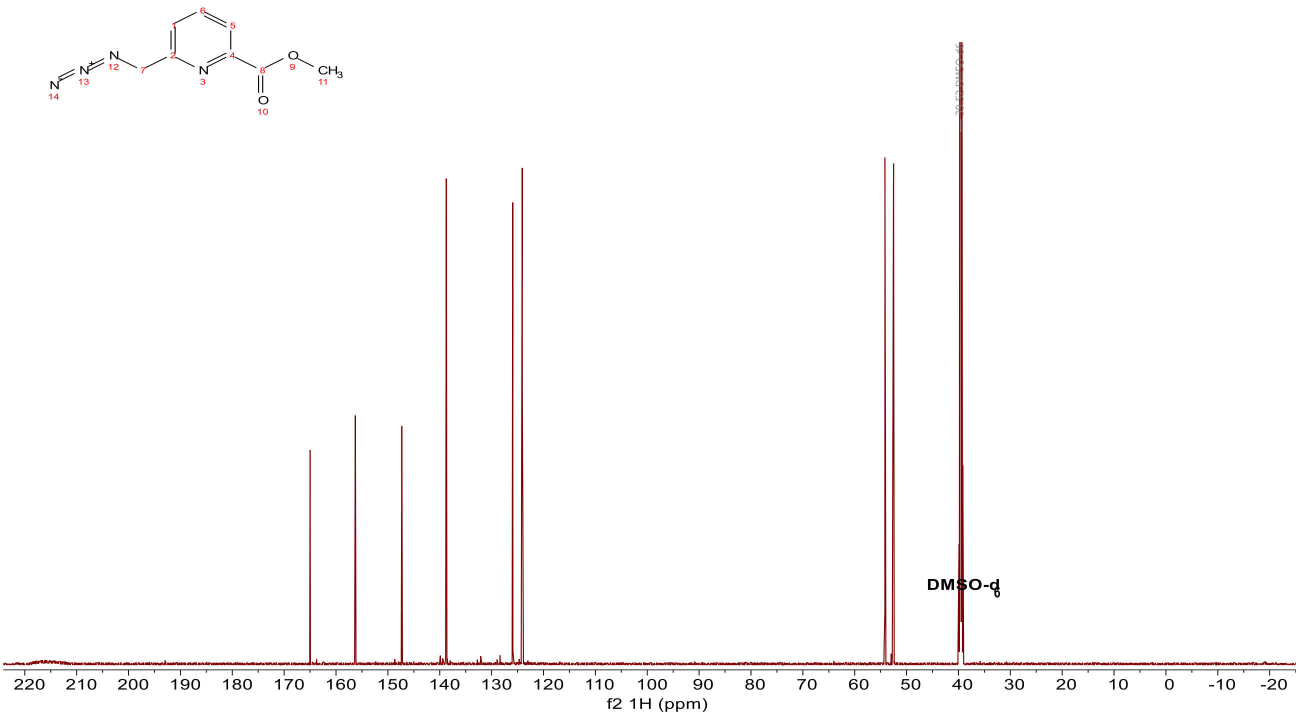

12 ^{1}H NMR

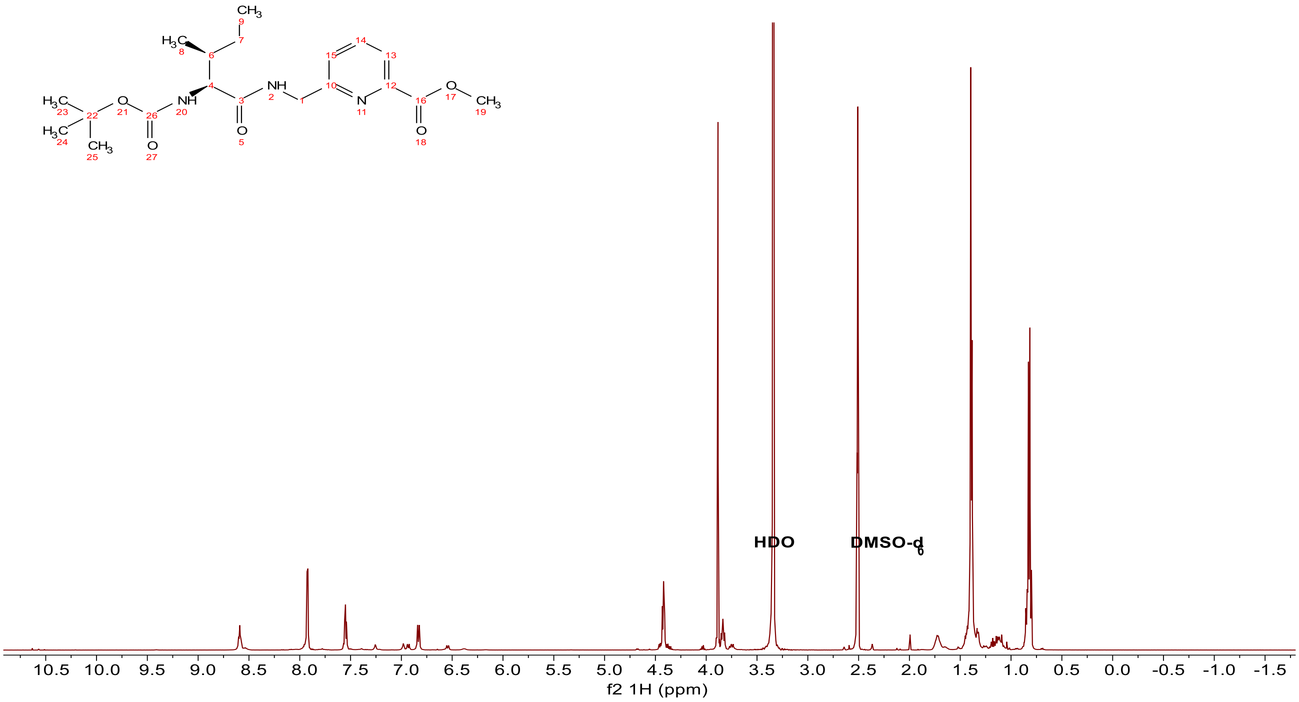

12 ^{13}C NMR

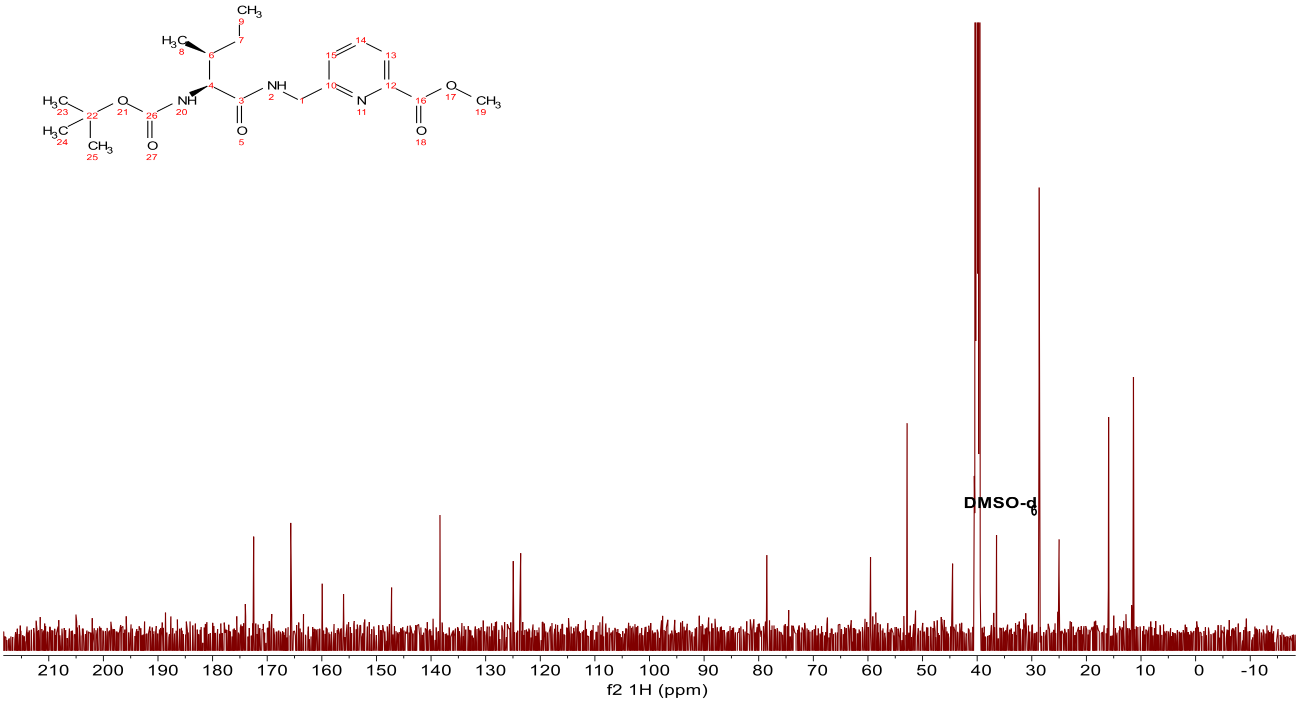

13 ^{1}H NMR

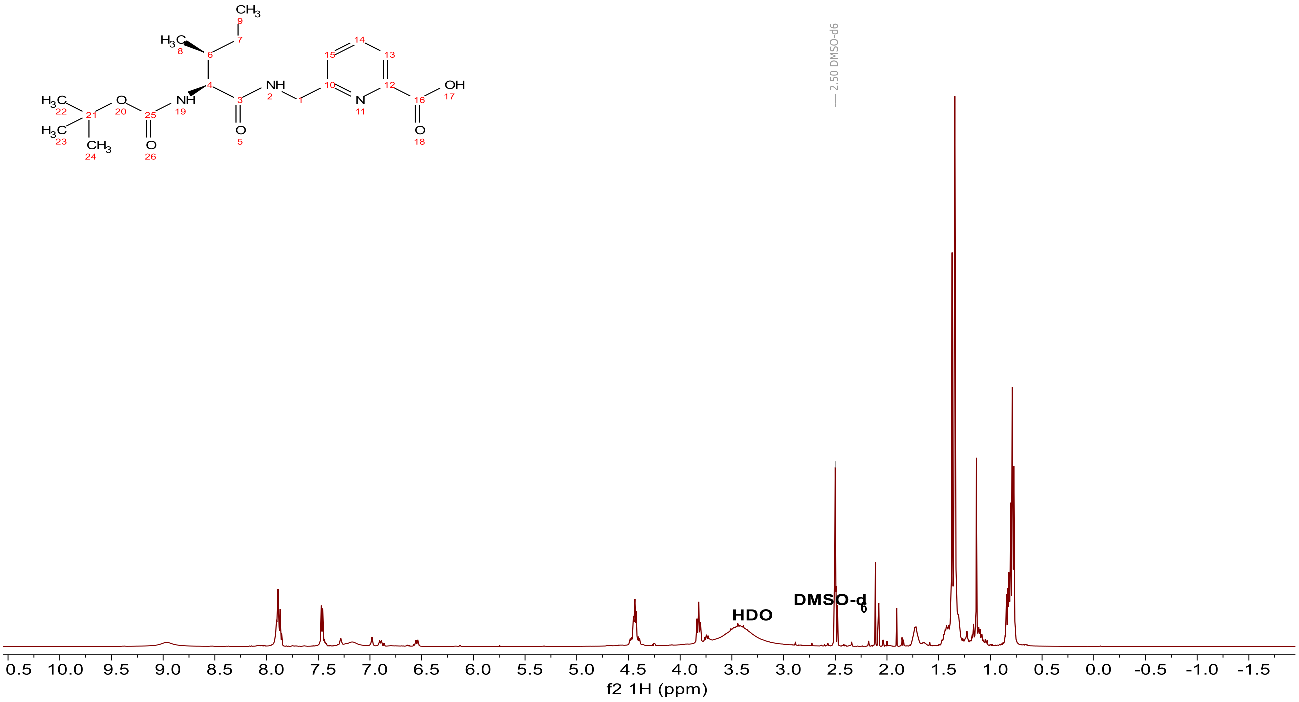

13 ^{13}C NMR

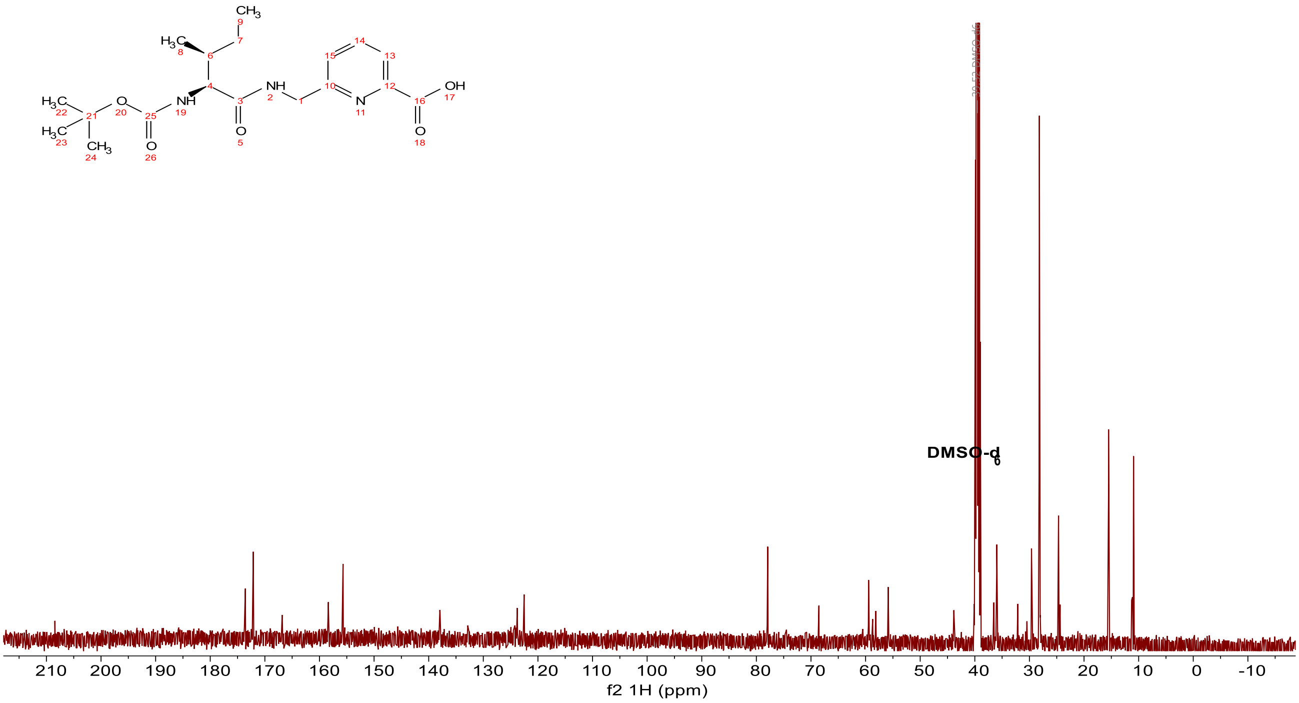

14 ^{1}H NMR

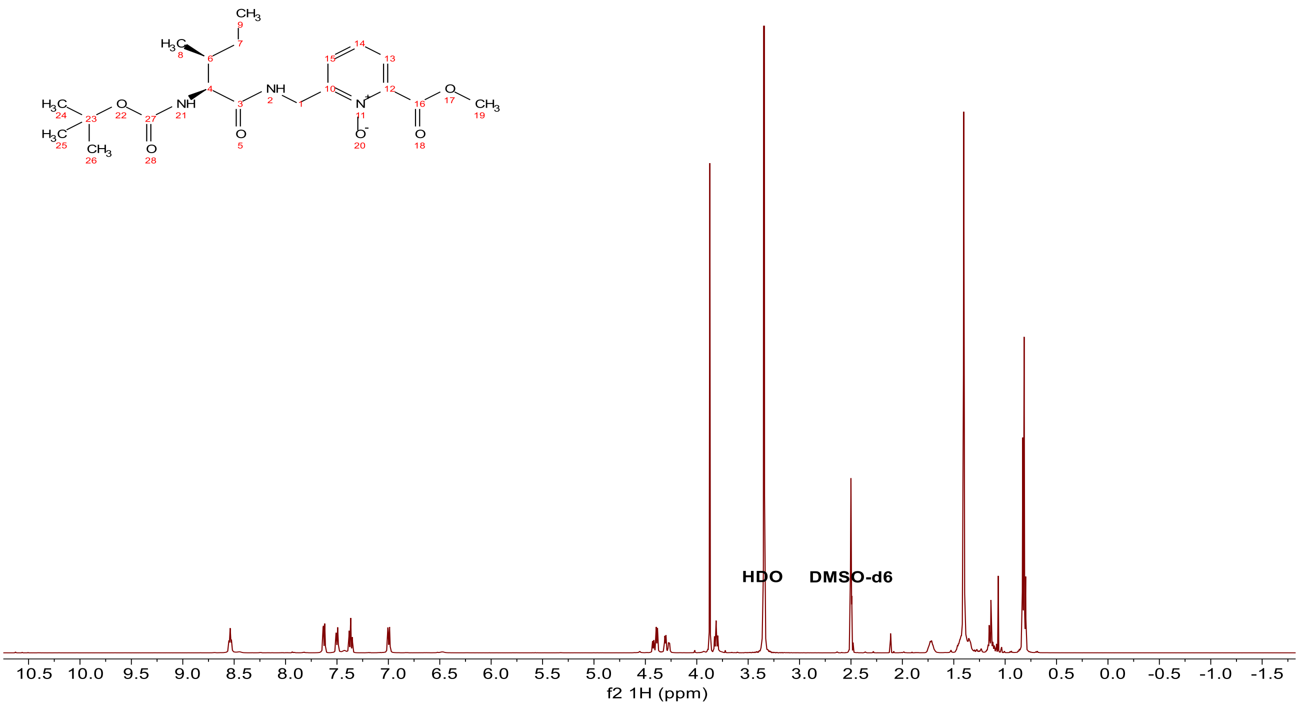

14 ^{13}C NMR

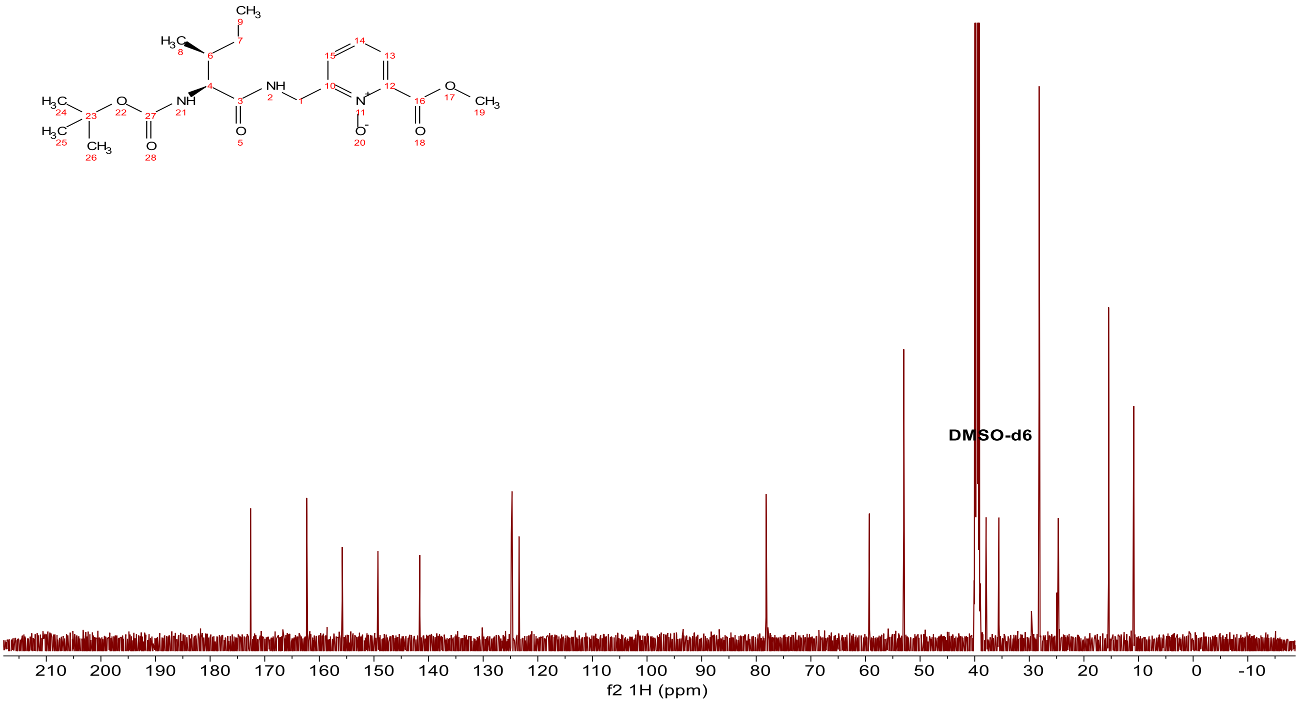

15 ^{1}H NMR

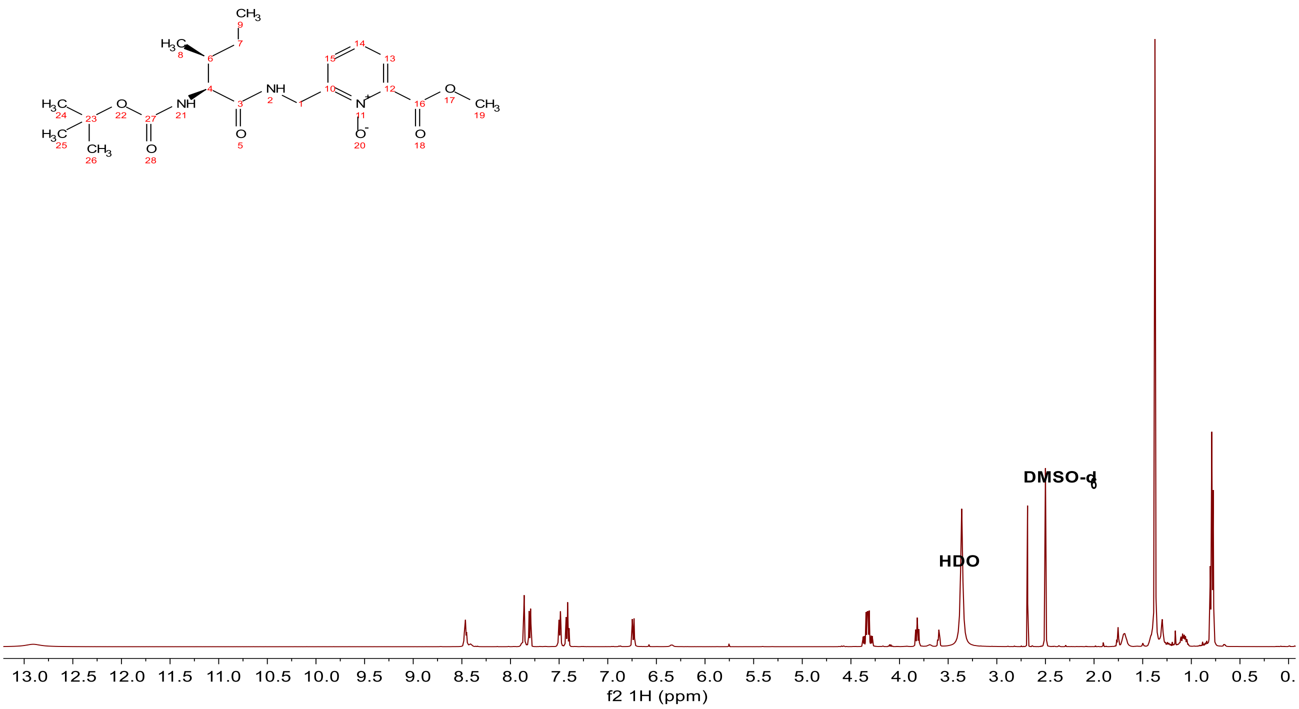

15 ^{13}C NMR

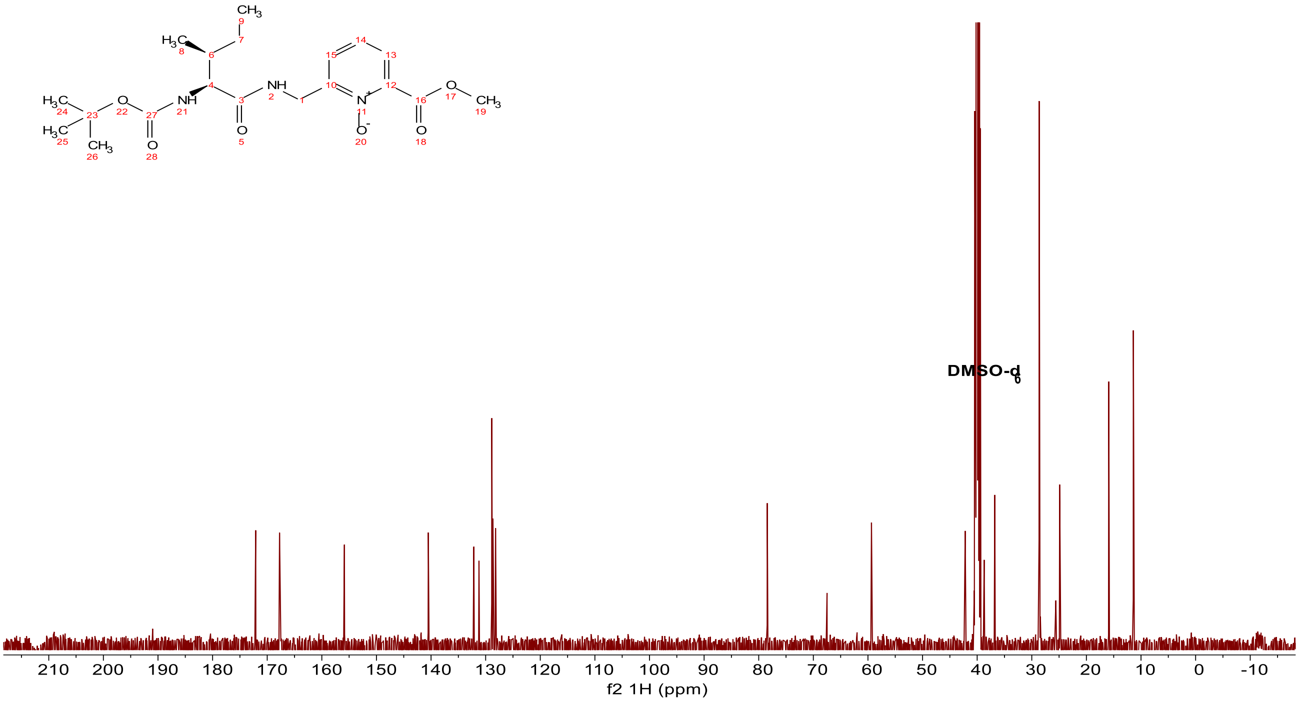

16 ^{1}H NMR

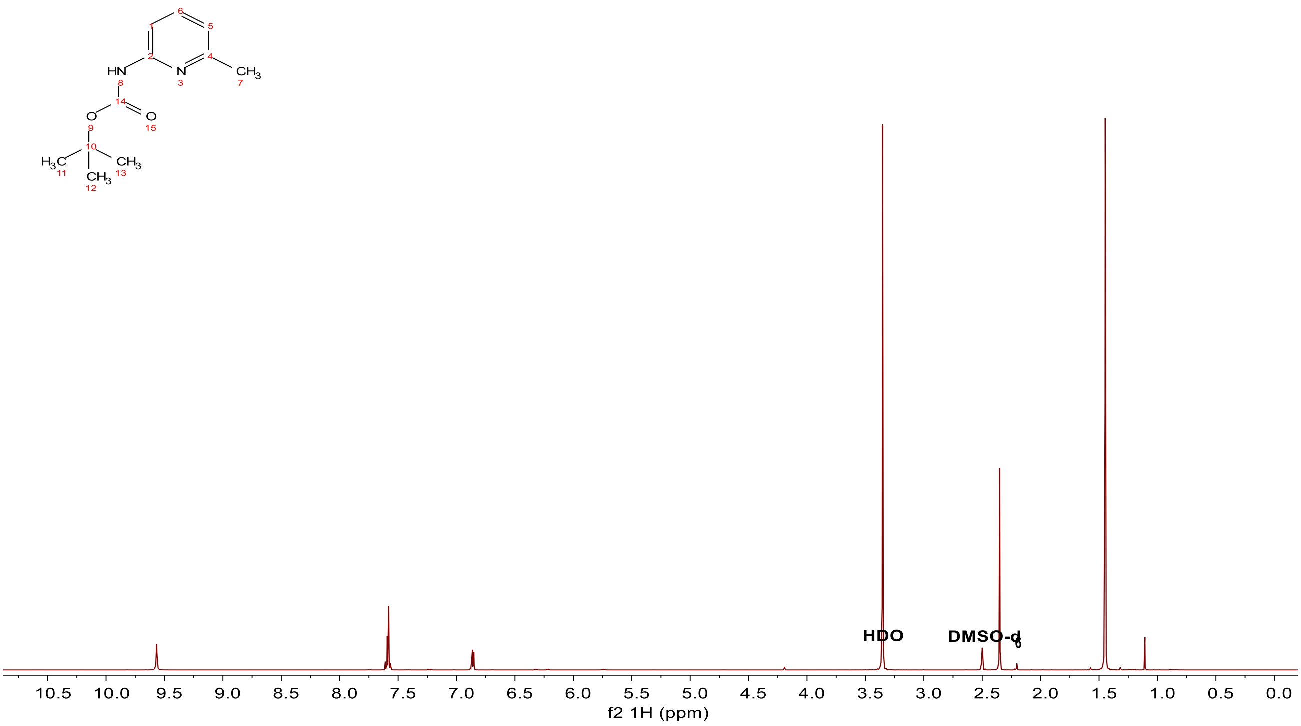

16 ^{13}C NMR

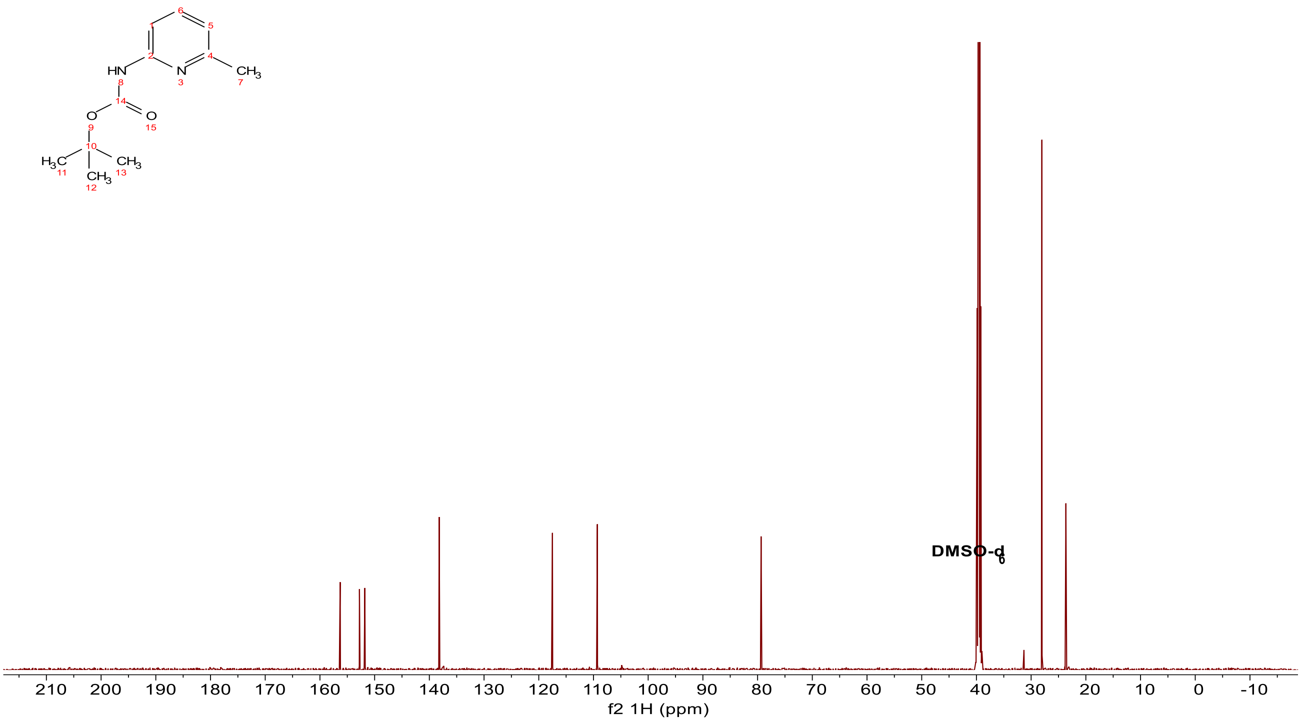

17 ^{1}H NMR

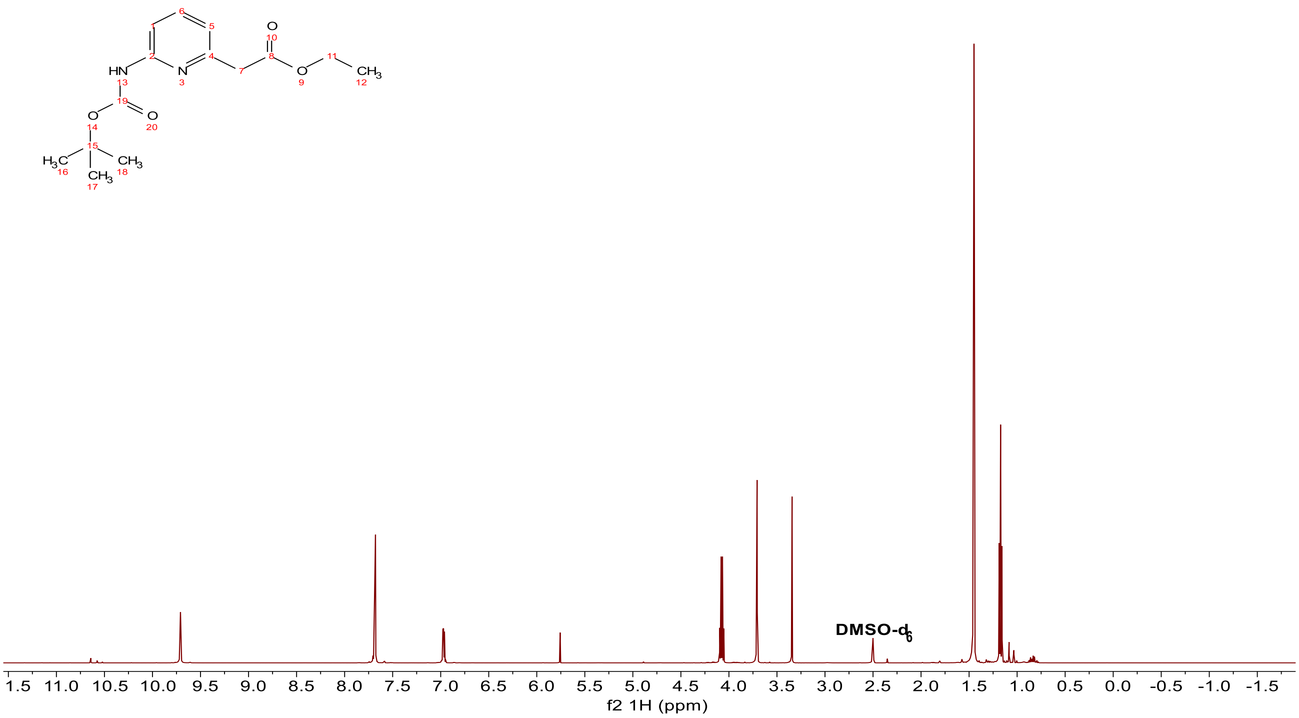

17 ^{13}C NMR

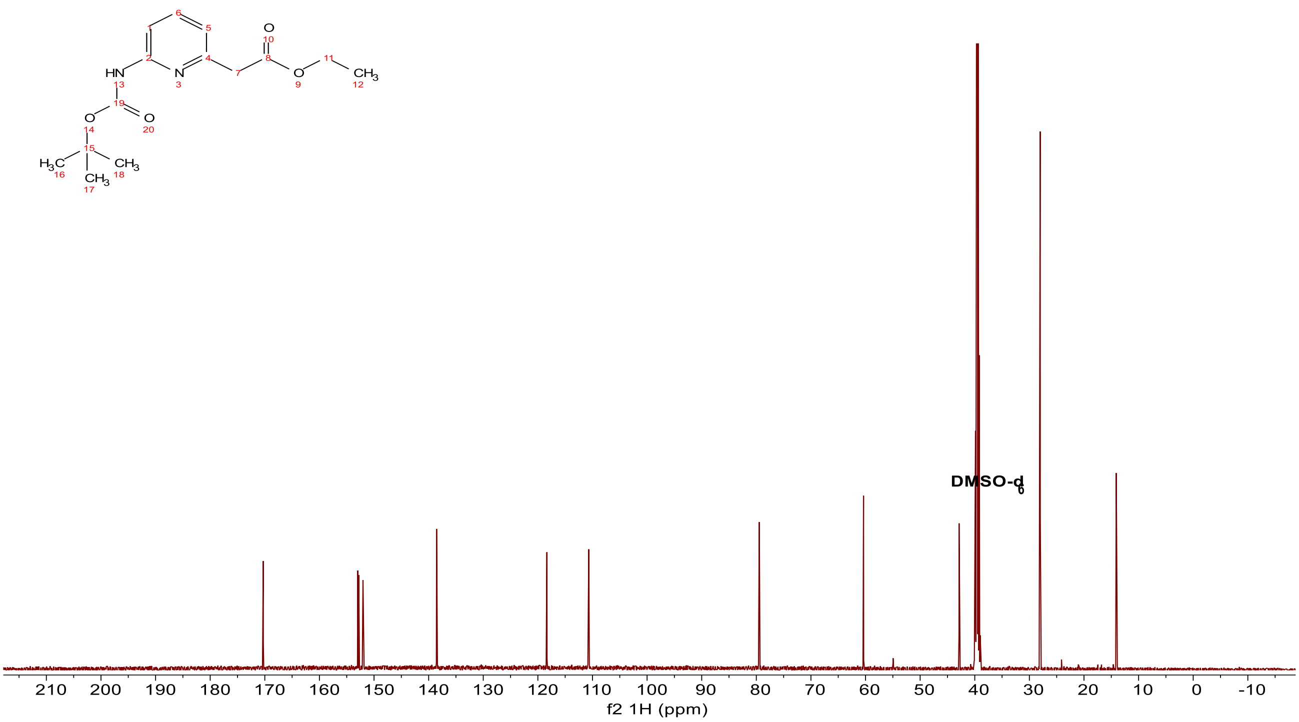

18 ^{1}H NMR

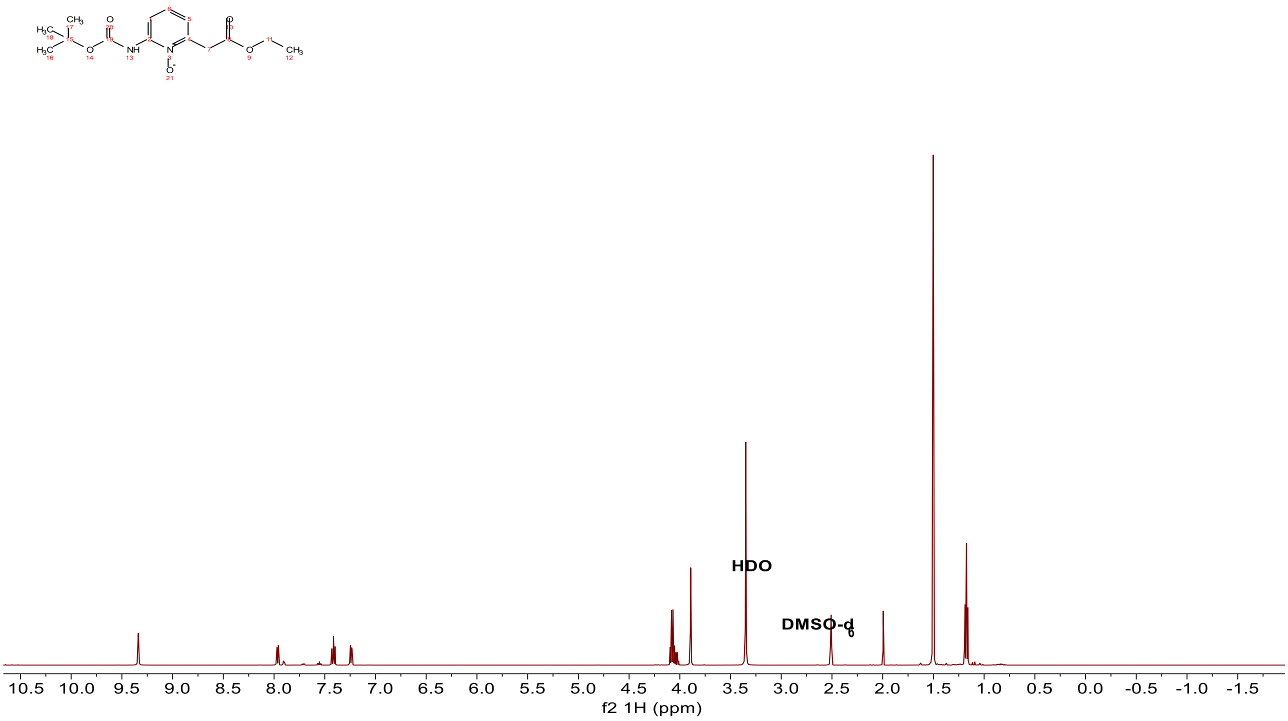

18 ^{13}C NMR

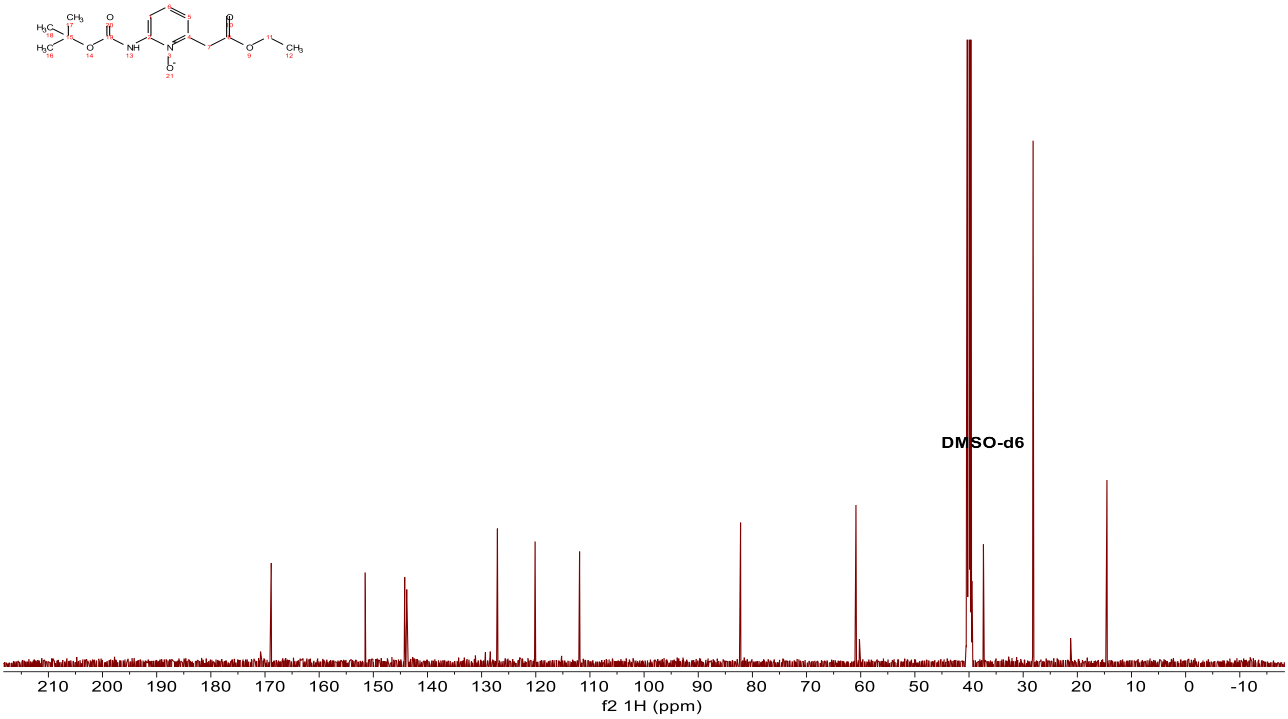

20 ^{1}H NMR

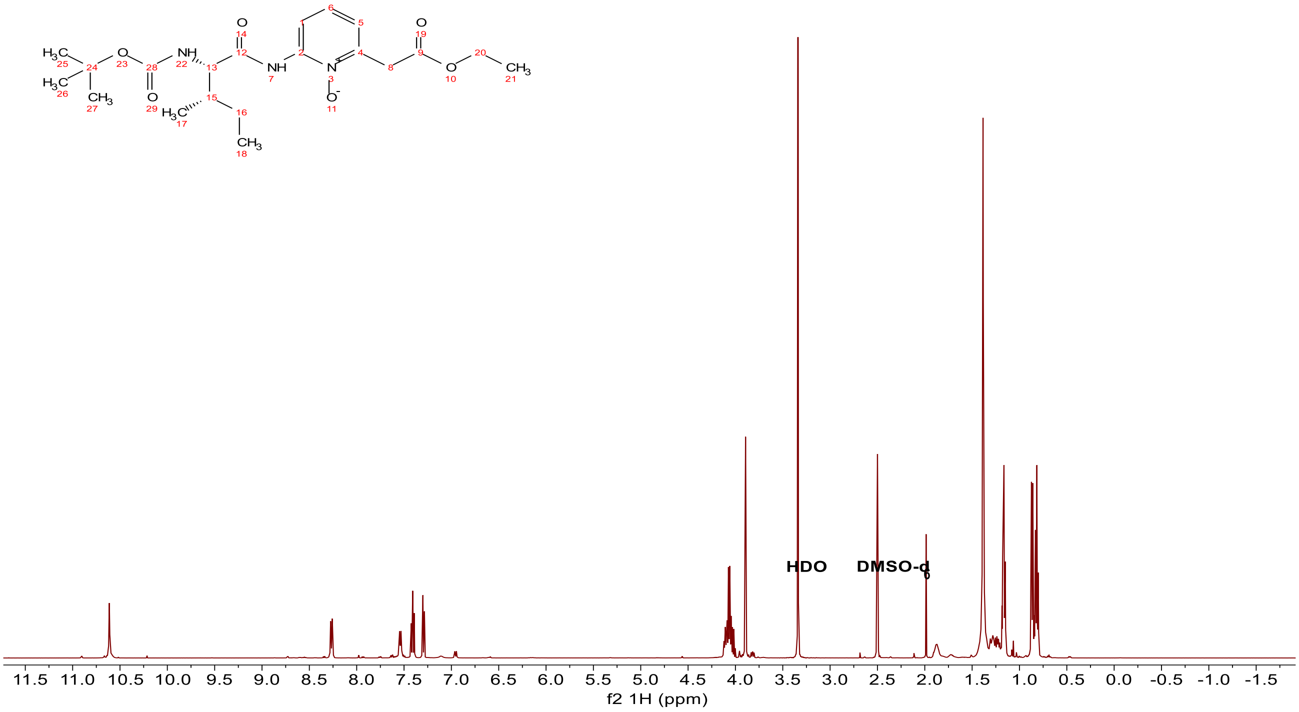

20 ^{13}C NMR

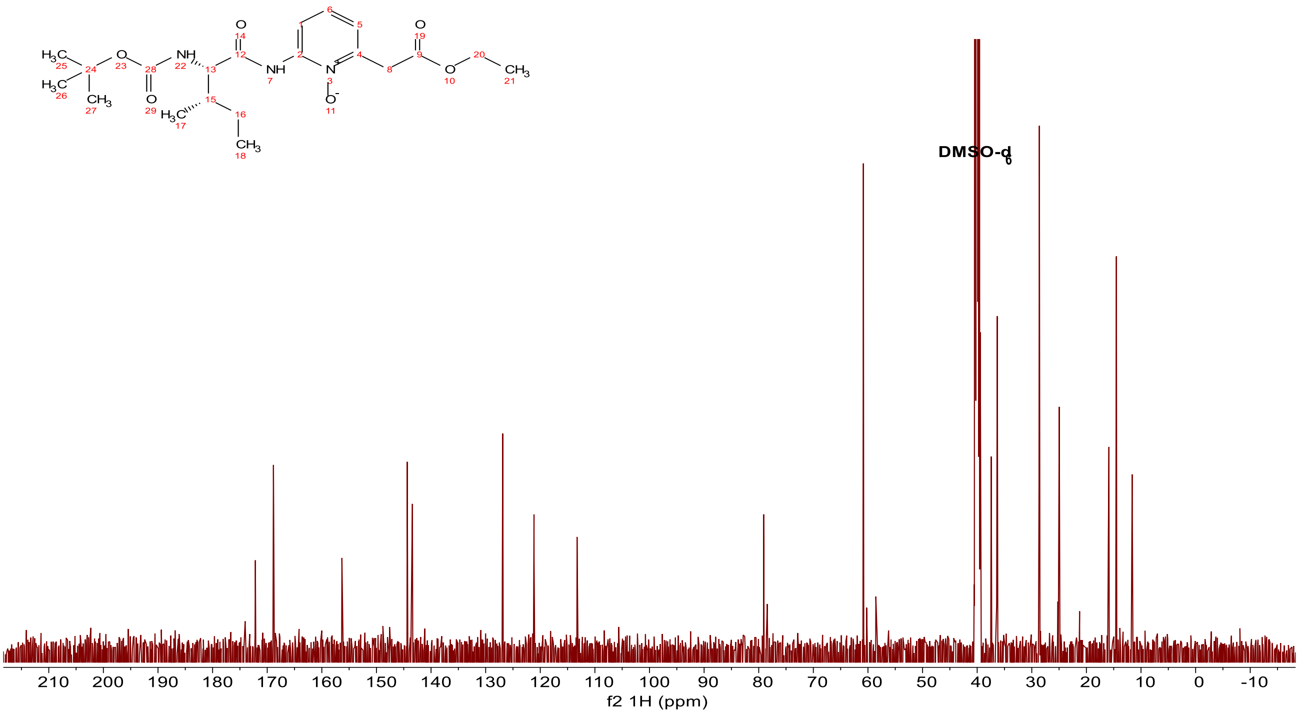

21 ^{1}H NMR

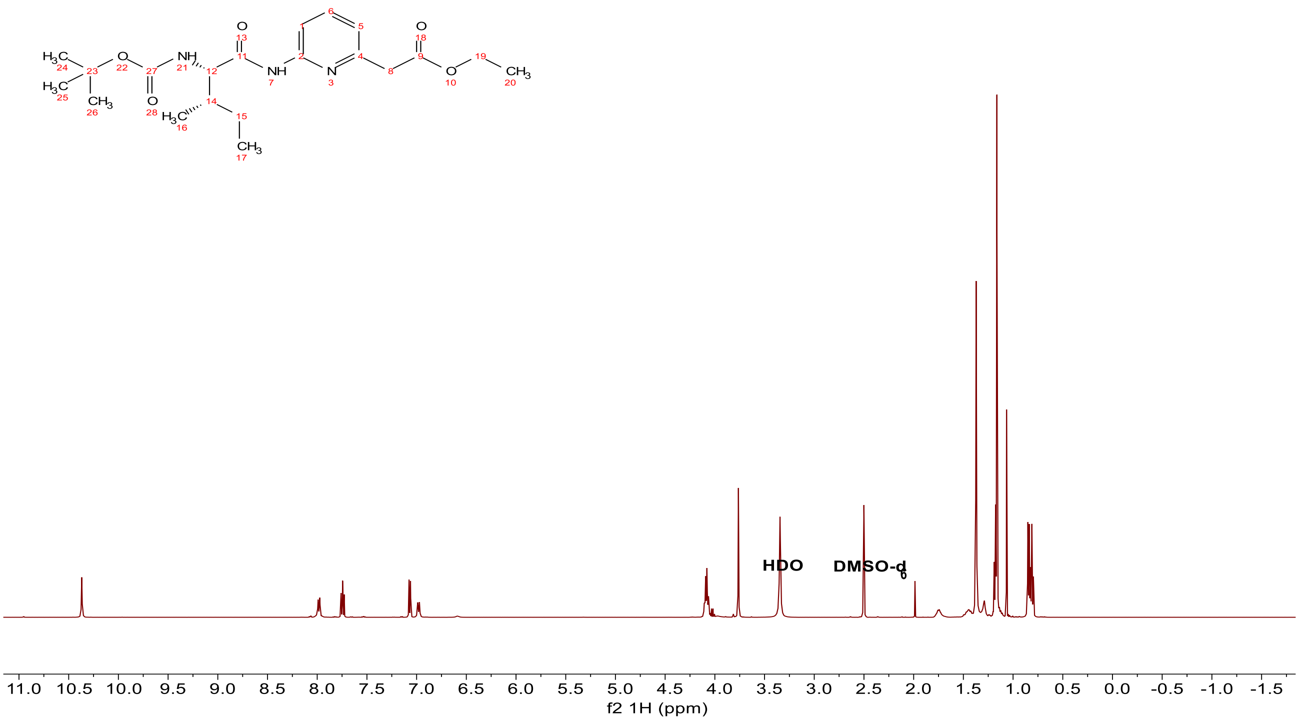

21 ^{13}C NMR

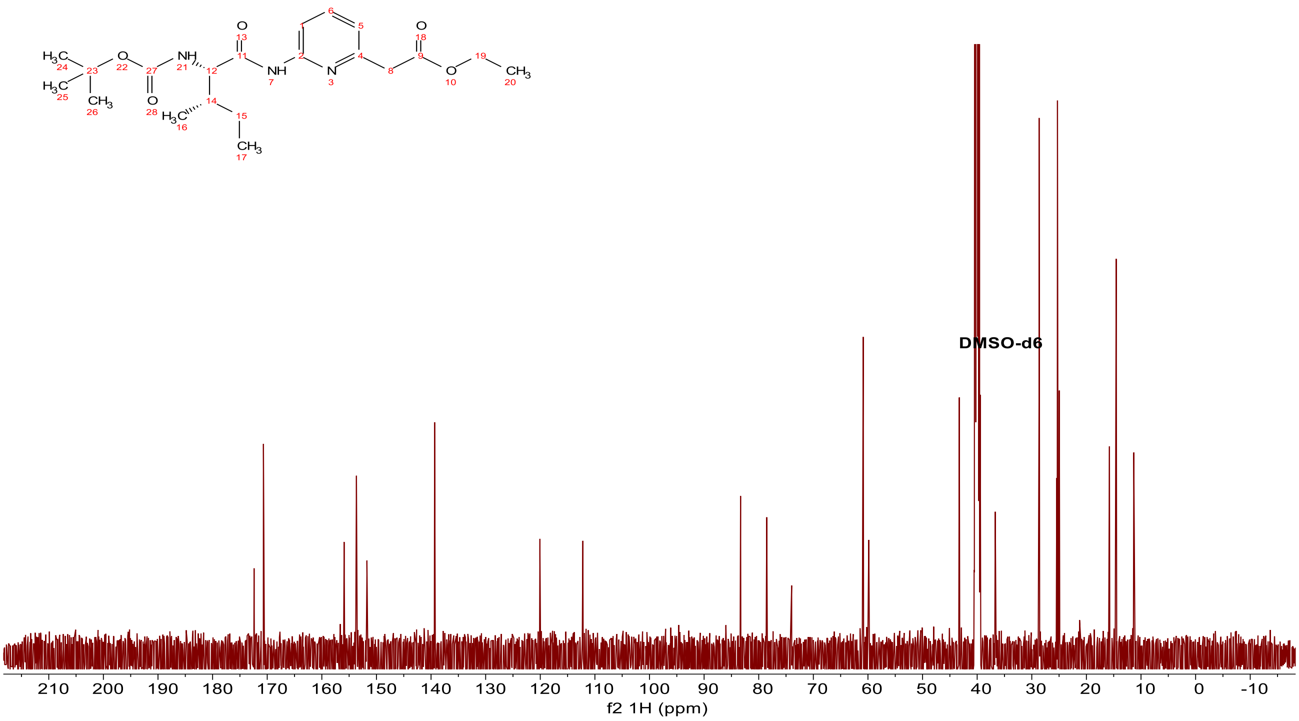

22 ^{1}H NMR

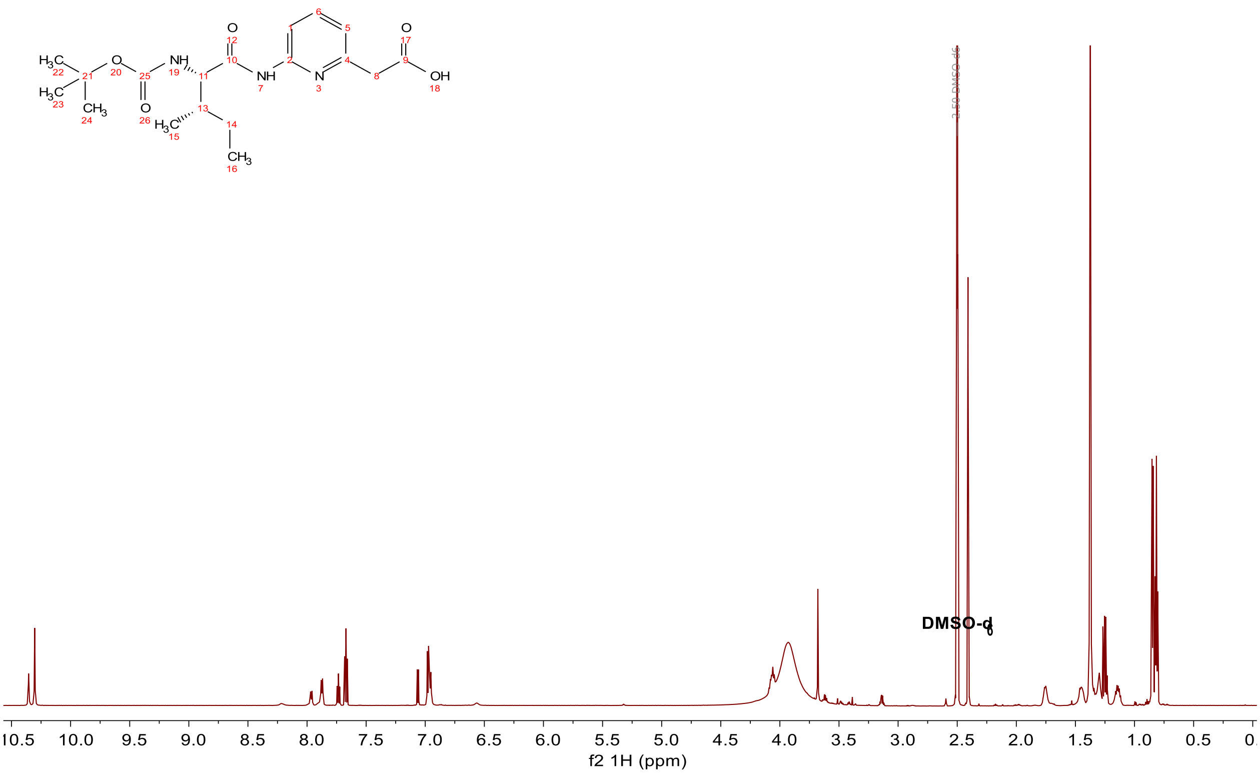

22 ^{13}C NMR

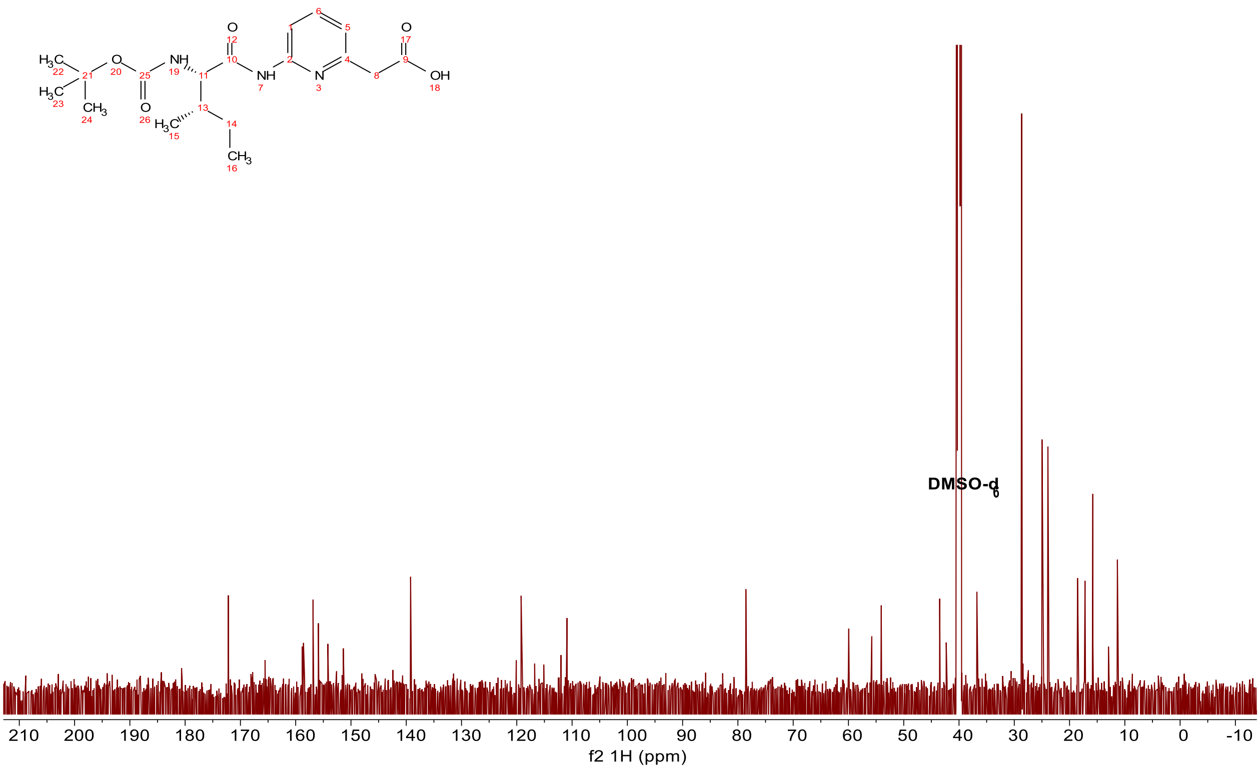

23 ^{1}H NMR

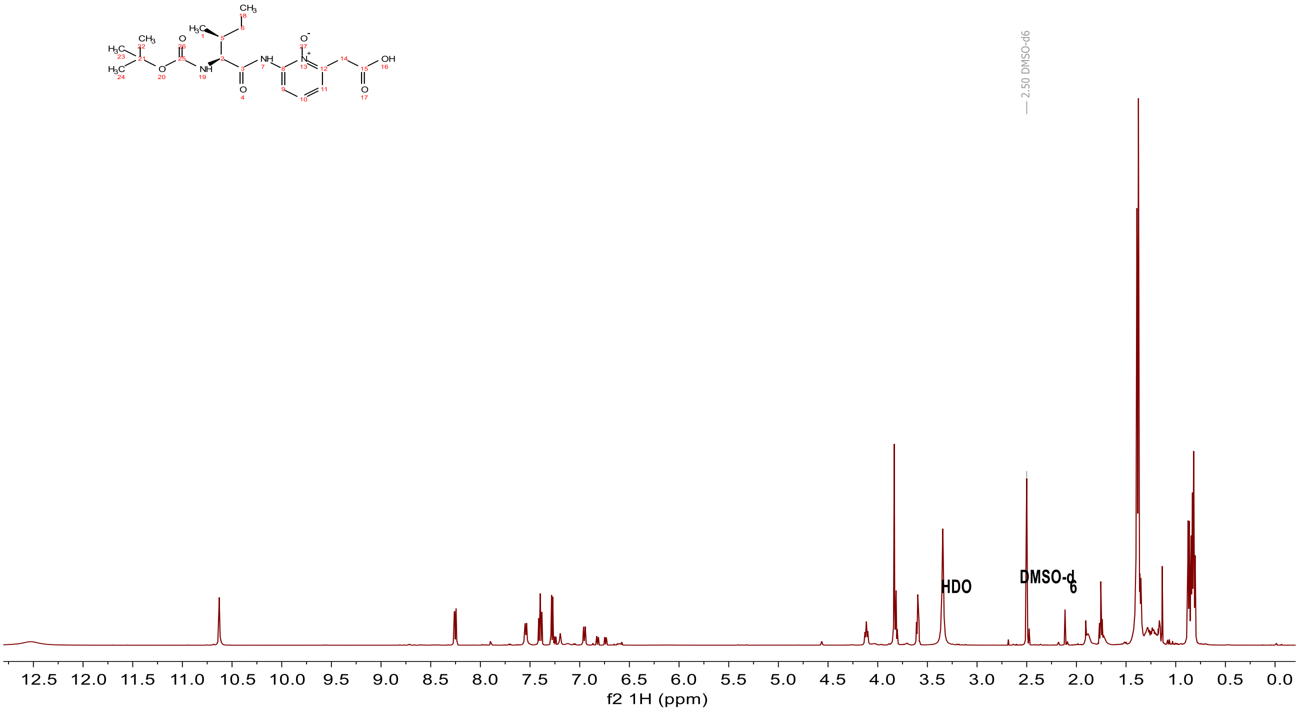

23 ^{13}C NMR

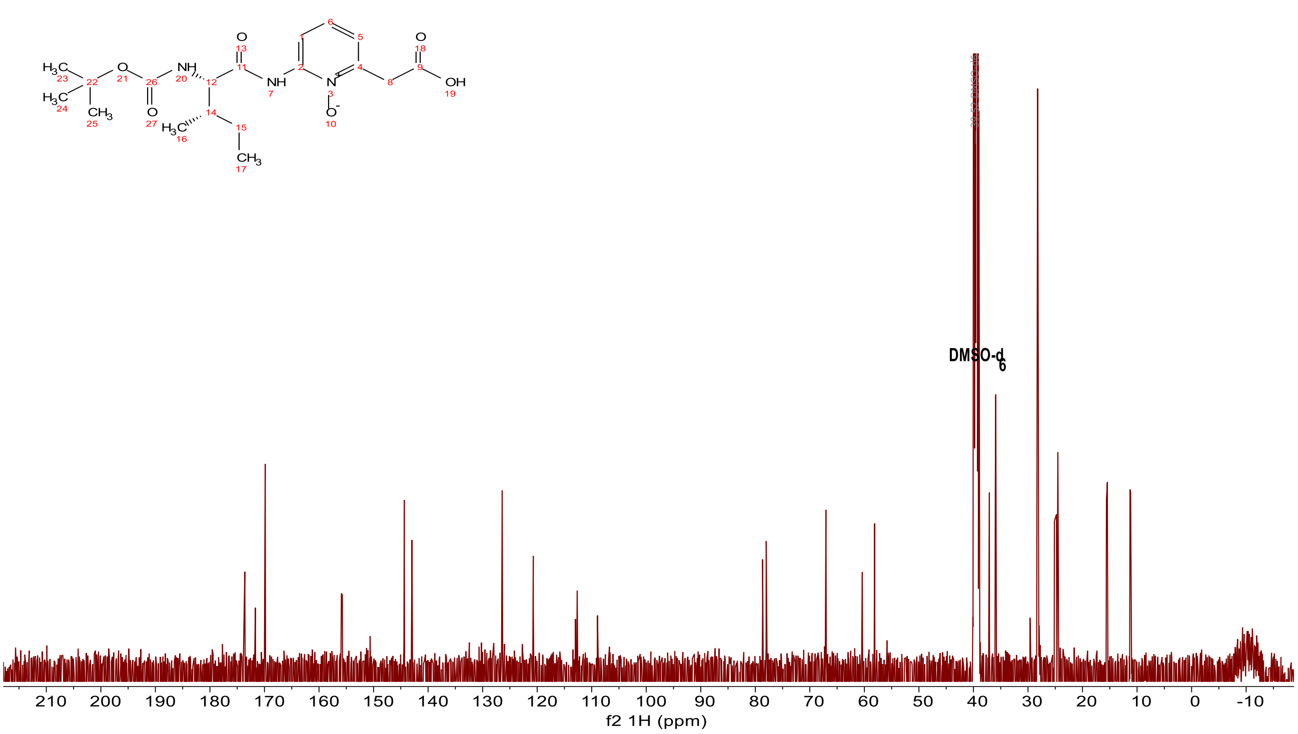

24 ^{1}H NMR

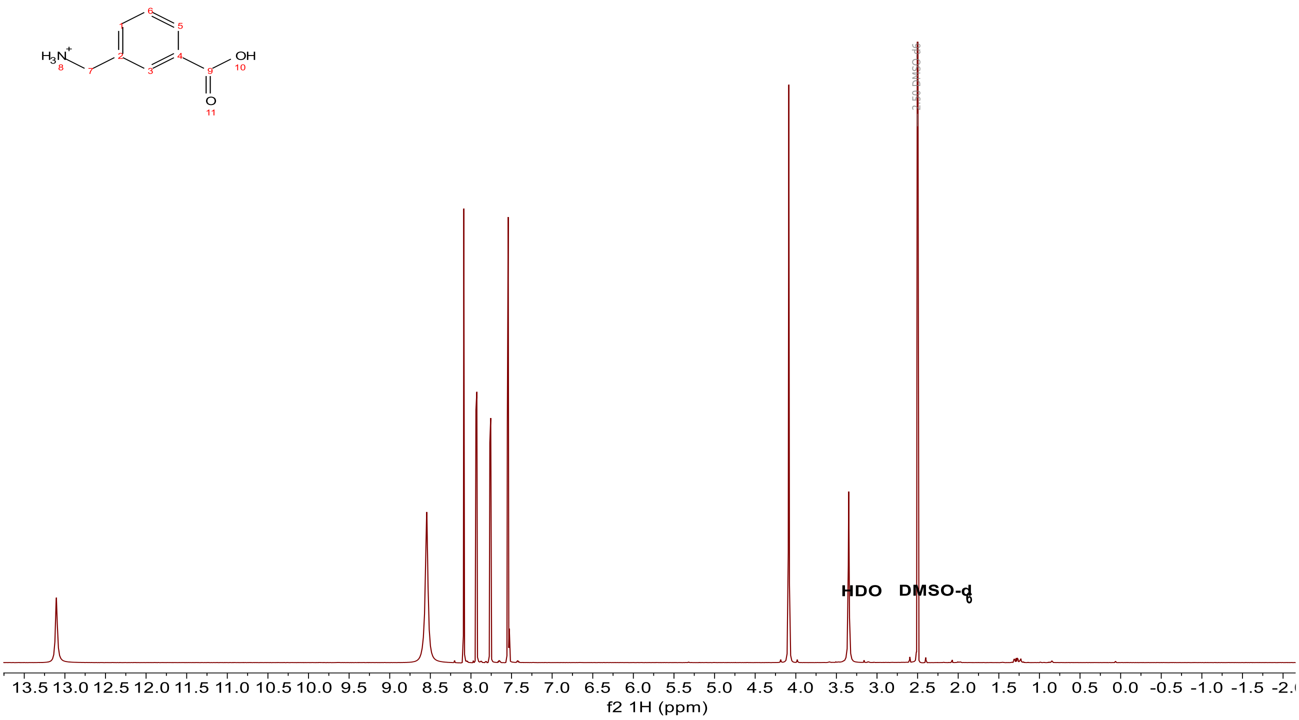

24 ^{13}C NMR

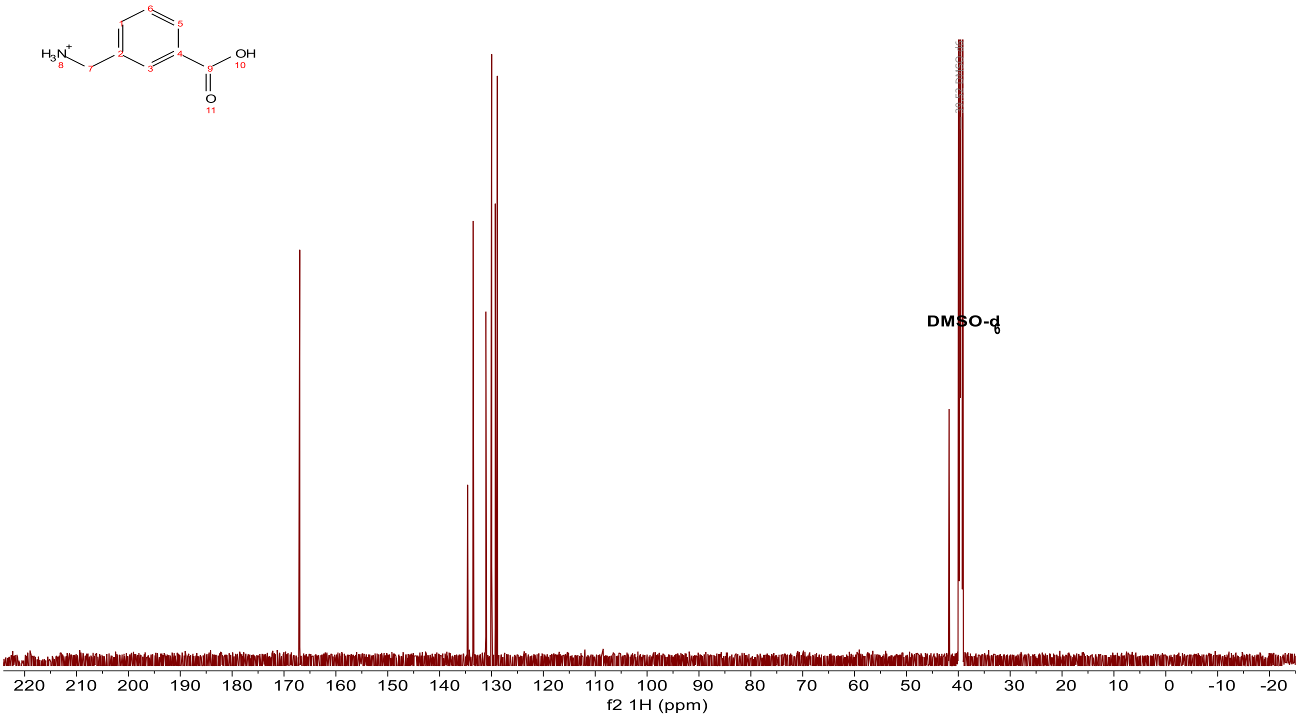

25 ^{1}H NMR

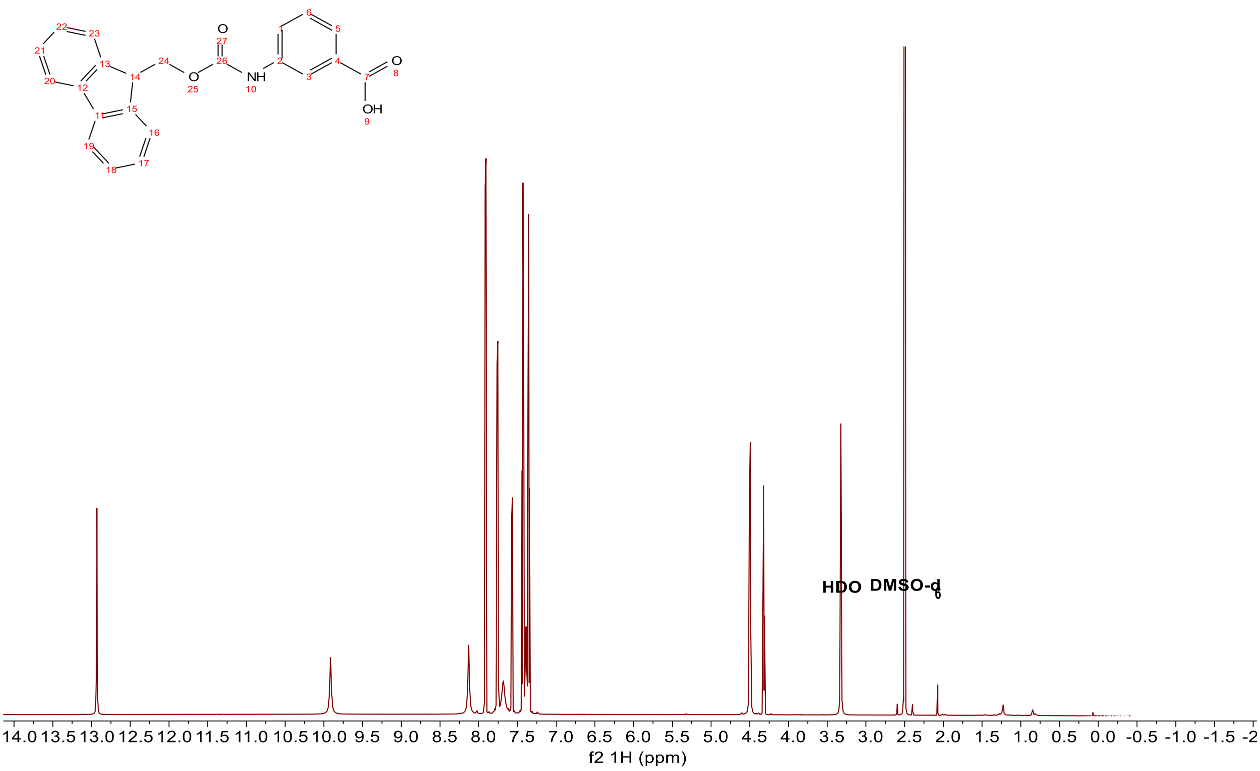

25 ^{13}C NMR

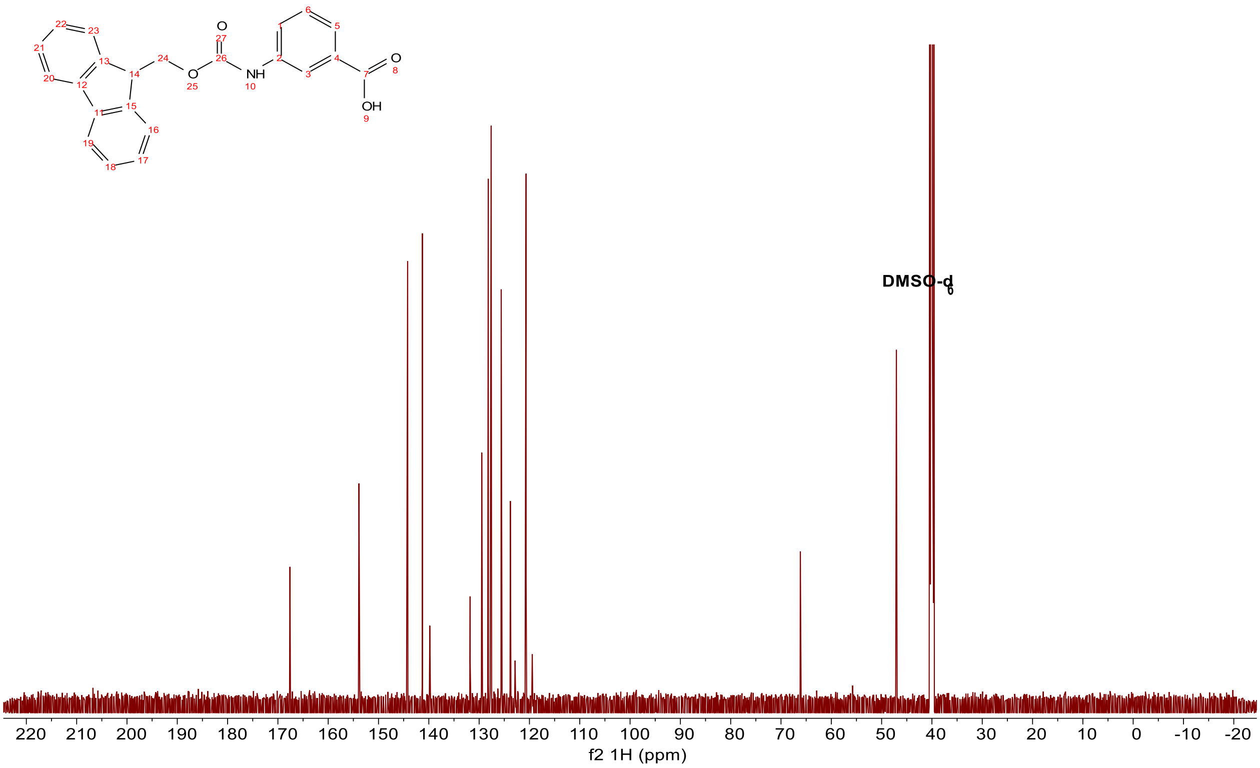

26 ^{1}H NMR

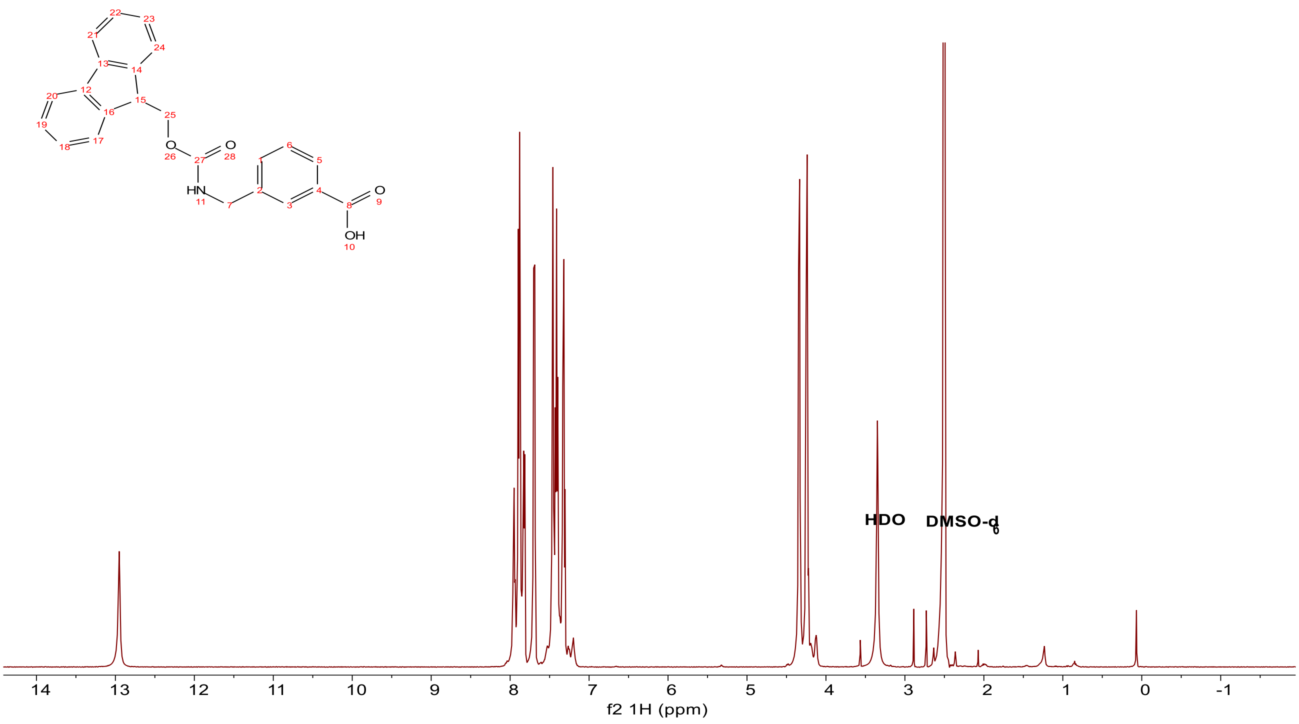

26 ^{13}C NMR

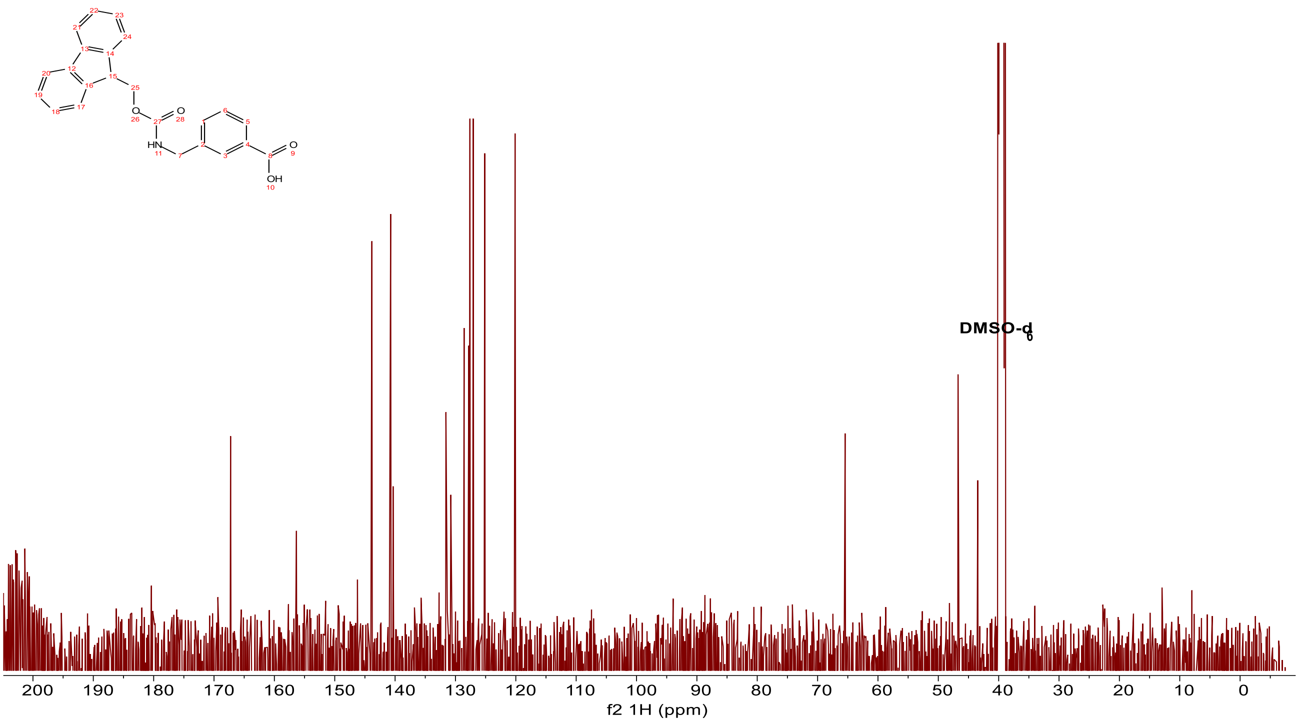

27 ^{1}H NMR

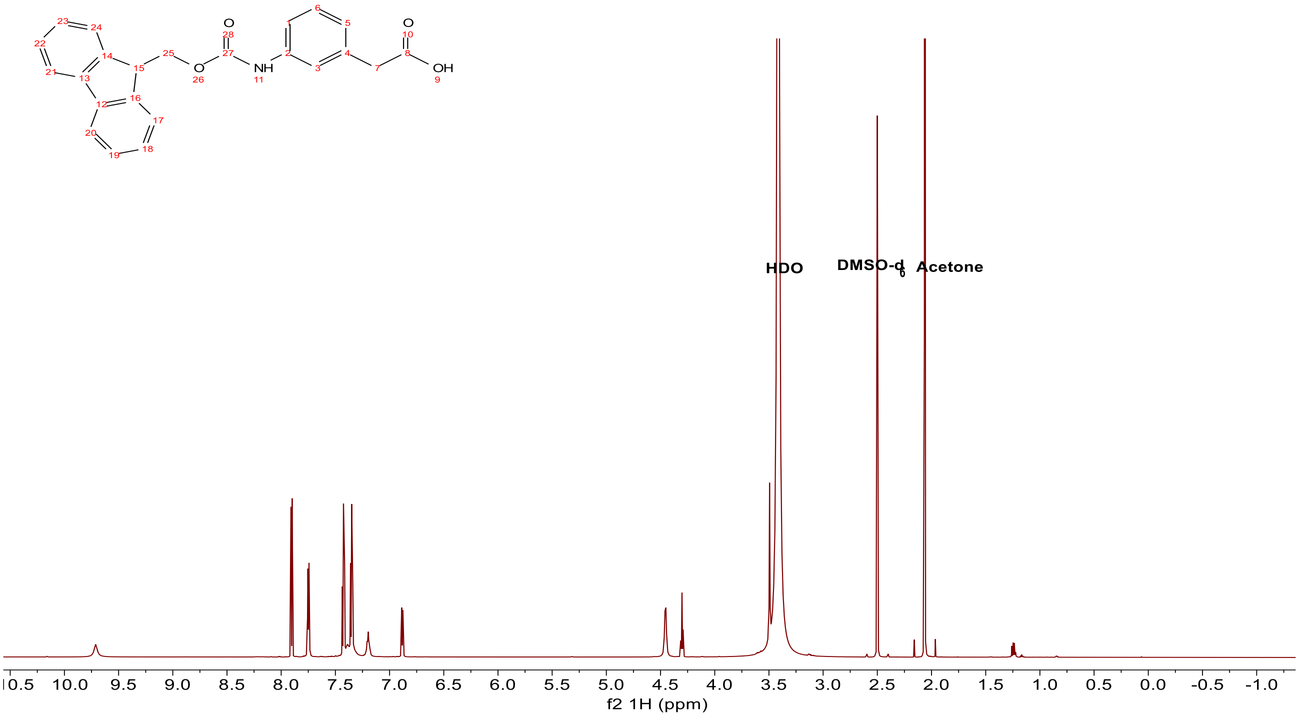

27 ^{13}C NMR

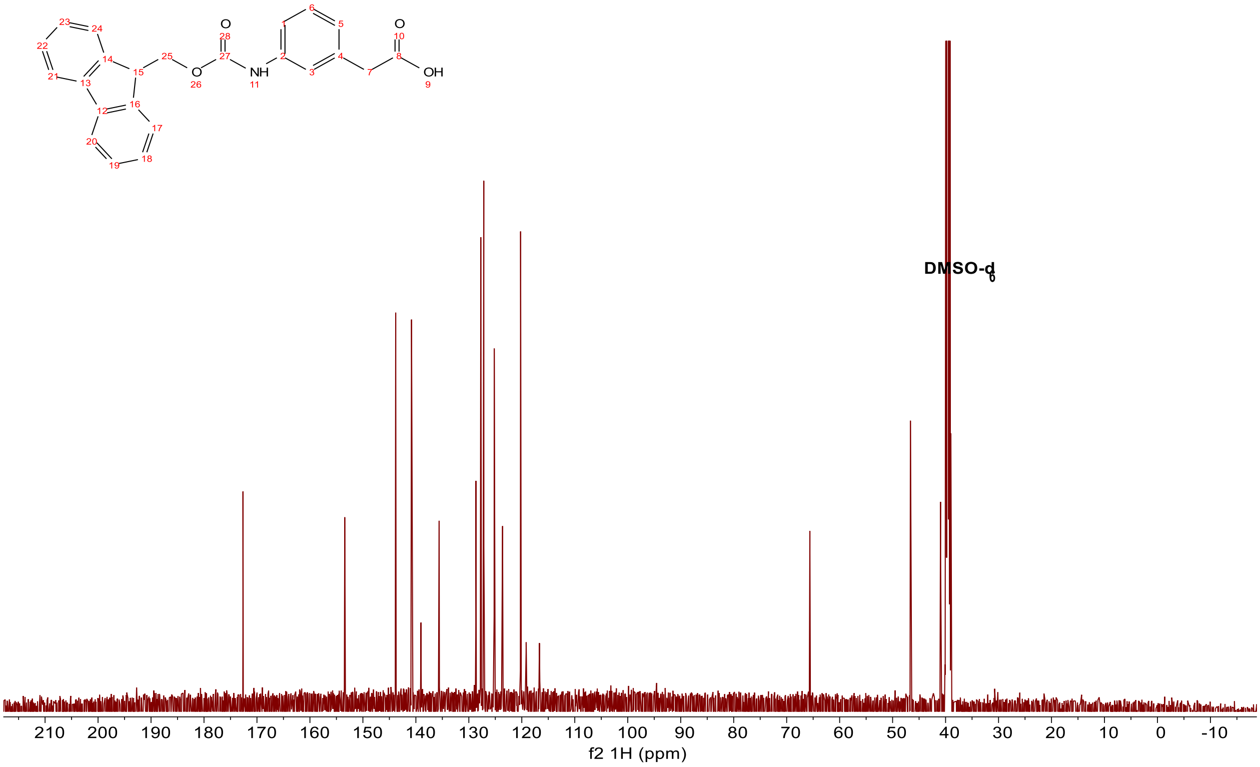

28 ^{1}H NMR

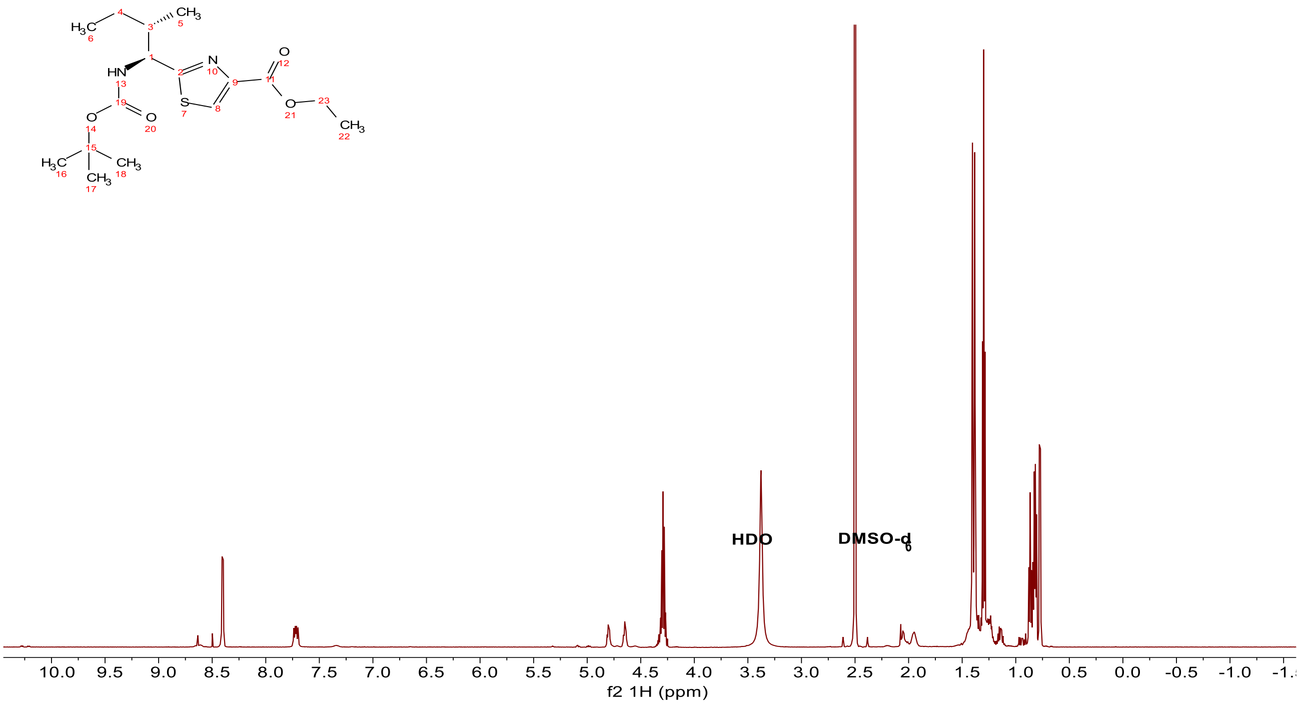

28 ^{13}C NMR

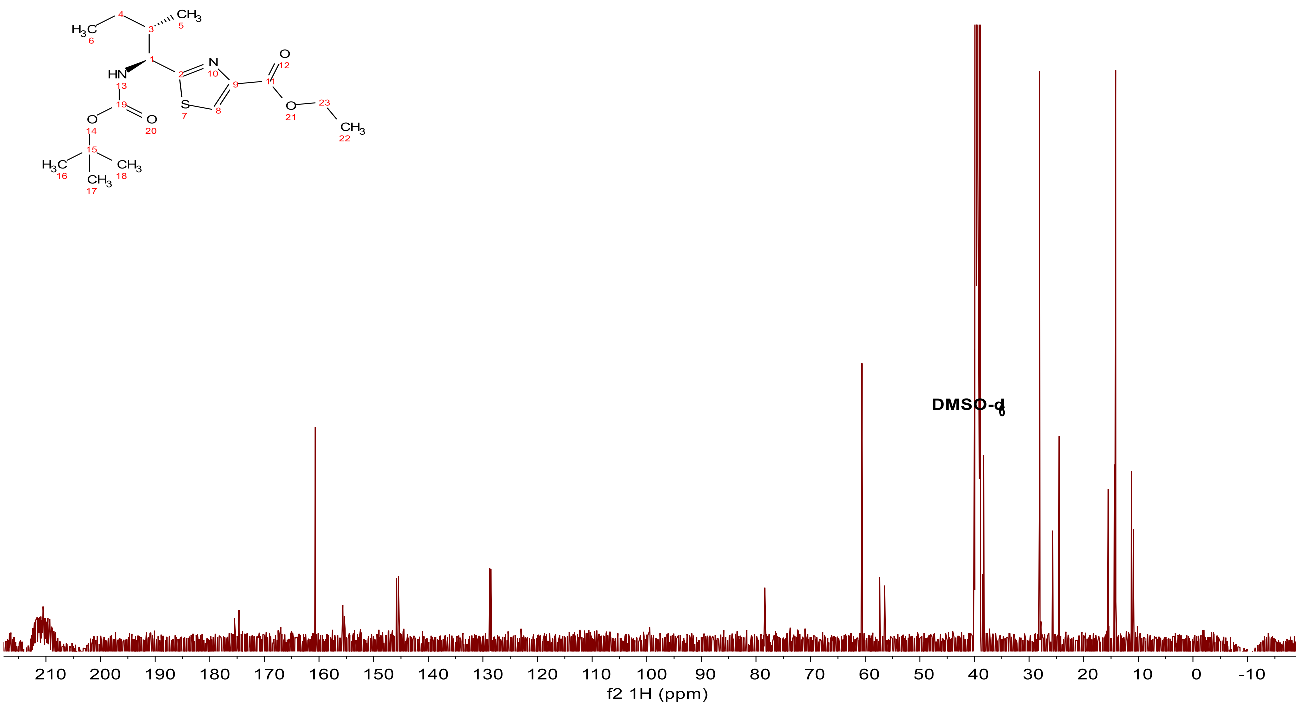

29 ^{1}H NMR

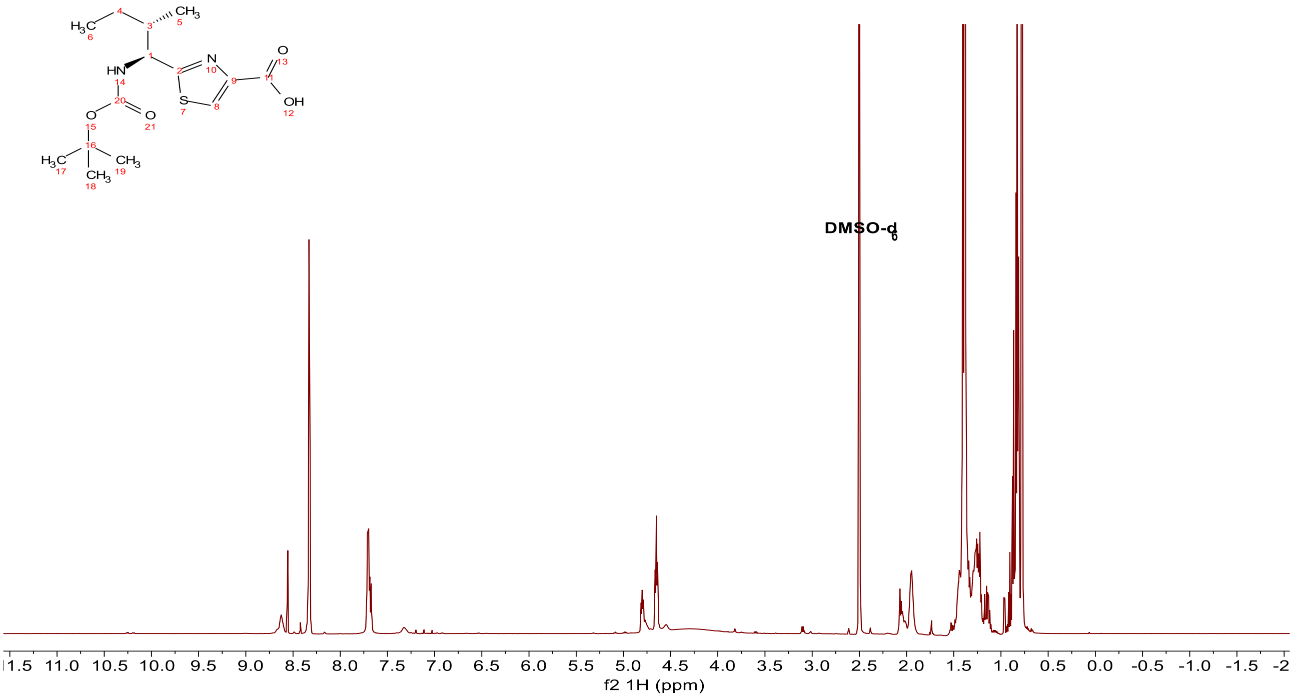

29 ^{13}C NMR

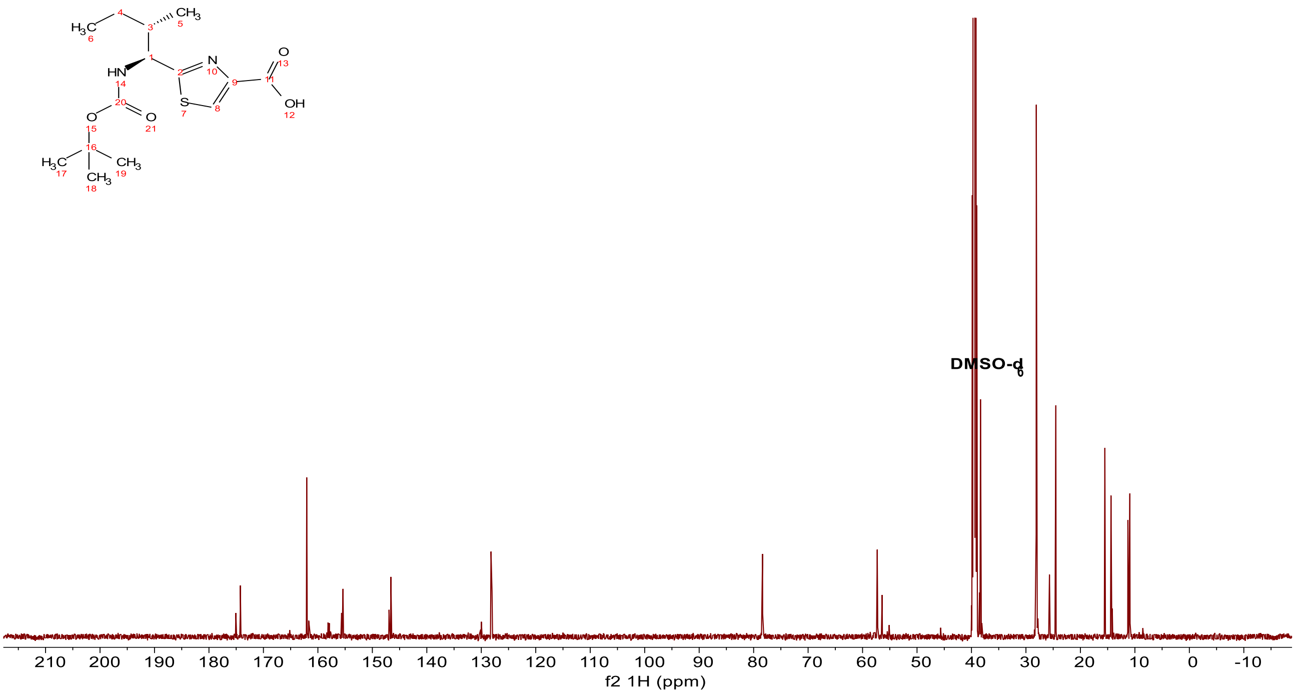

Supplement: Supplementary file 1 [file jm5c01901_si_001.pdf]
